# The Effects of Vitamin D Supplementation During Pregnancy on Maternal, Neonatal, and Infant Health: A Systematic Review and Meta-analysis

**DOI:** 10.1093/nutrit/nuae065

**Published:** 2024-07-01

**Authors:** Wen-Chien Yang, Ramaa Chitale, Karen M O’Callaghan, Christopher R Sudfeld, Emily R Smith

**Affiliations:** Department of Global Health, Milken Institute School of Public Health, The George Washington University, Washington, DC 20037, United States; Department of Global Health, Milken Institute School of Public Health, The George Washington University, Washington, DC 20037, United States; Department of Nutritional Sciences, King’s College London, London, United Kingdom; Department of Global Health and Population, Harvard T. H. Chan School of Public Health, Boston, MA 02115, United States; Department of Nutrition, Harvard T. H. Chan School of Public Health, Boston, MA 02115, United States; Department of Global Health, Milken Institute School of Public Health, The George Washington University, Washington, DC 20037, United States; Department of Exercise and Nutrition Sciences, Milken Institute School of Public Health, The George Washington University, Washington, DC 20037, United States

**Keywords:** meta-analysis, vitamin D, systematic review, pregnancy, micronutrient supplementation, maternal and child health

## Abstract

**Context:**

Previous research linked vitamin D deficiency in pregnancy to adverse pregnancy outcomes.

**Objective:**

Update a 2017 systematic review and meta-analysis of randomized controlled trials (RCTs) on the effect of vitamin D supplementation during pregnancy, identify sources of heterogeneity between trials, and describe evidence gaps precluding a clinical recommendation.

**Data Sources:**

The MEDLINE, PubMed, Europe PMC, Scopus, Cochrane Database of Systematic Reviews, Web of Science, and CINAHL databases were searched. Articles were included that reported on RCTs that included pregnant women given vitamin D supplements as compared with placebo, no intervention, or active control (≤600 IU d^–1^). Risk ratios (RRs) and mean differences were pooled for 38 maternal, birth, and infant outcomes, using random effects models. Subgroup analyses examined effect heterogeneity. The Cochrane risk of bias tool was used.

**Data Extraction:**

Included articles reported on a total of 66 trials (n = 17 276 participants).

**Data Analysis:**

The median vitamin D supplementation dose was 2000 IU d^–1^ (range: 400-60 000); 37 trials used placebo. Antenatal vitamin D supplementation had no effect on the risk of preeclampsia (RR, 0.81 [95% CI, 0.43-1.53]; n = 6 trials and 1483 participants), potentially protected against gestational diabetes mellitus (RR, 0.65 [95% CI, 0.49-0.86; n = 12 trials and 1992 participants), and increased infant birth weight by 53 g (95% CI, 16-90; n = 40 trials and 9954 participants). No effect of vitamin D on the risk of preterm birth, small-for-gestational age, or low birth weight infants was found. A total of 25 trials had at least 1 domain at high risk of bias.

**Conclusion:**

Additional studies among the general pregnant population are not needed, given the many existing trials. Instead, high-quality RCTs among populations with low vitamin D status or at greater risk of key outcomes are needed. Benefits of supplementation in pregnancy remain uncertain because current evidence has high heterogeneity, including variation in study context, baseline and achieved end-line 25-hydroxyvitamin D level, and studies with high risk of bias.

**Systematic Review Registration:**

PROSPERO registration no. CRD42022350057.

## INTRODUCTION

Researchers debate the role of vitamin D supplementation in pregnancy despite there being established benefits of the micronutrient during other times in the life course. Scientists have established that vitamin D modulates calcium metabolism and is essential for bone health. Additionally, researchers have identified immunomodulatory and other extraskeletal effects of vitamin D.^1^

Vitamin D supplementation in pregnancy remains controversial because findings from observational studies and clinical trials seemingly contradict each other. Observational studies and relevant meta-analyses have noted an association between low vitamin D status (as reflected by 25-hydroxyvitamin D [25(OH)D] concentration) in pregnancy and adverse pregnancy outcomes, although many of these studies may be prone to bias.^2–7^ Randomized trials of vitamin D supplementation have examined effects on both maternal and infant outcomes but reported conflicting results.^8^

Two large meta-analyses pooled evidence from randomized controlled trials (RCTs) on this topic and generally aligned in their findings despite methodological differences.^9^^,^^10^ Specifically, slight differences in results might be attributable to different study eligibility criteria. Both meta-analyses found null effects on gestational hypertension, cesarean section delivery, stillbirth, and preterm birth.^9^^,^^10^ In terms of differences, the 2017 Roth et al review^9^ found null effects of prenatal vitamin D supplementation on risks of preeclampsia and gestational diabetes mellitus (GDM). In contrast, the 2019 Cochrane review by Palacios et al^10^ showed supplementation reduces the risks of these 2 outcomes. These frequently cited reviews highlight a gap in knowledge that clinical trialists continue to investigate about vitamin D supplementation in pregnancy.

Evidence remains insufficient for global recommendation of vitamin D supplementation for pregnant women. Currently, the World Health Organization does not recommend routine vitamin D supplementation for pregnant women.^11^^,^^12^ Similarly, the American College of Obstetricians and Gynecologists does not recommend routine vitamin D supplementation beyond that contained in prenatal vitamins to improve pregnancy outcomes.^13^ Still, given the variability in the prevalence of vitamin D deficiency in pregnancy globally, a universal recommendation may benefit geographic areas with a high prevalence of vitamin D deficiency.^14^

Since the publication of the Roth et al review in 2017,^9^ additional trials have been completed, thus offering an opportunity to re-examine and synthesize the evidence. New evidence includes more than 30 ongoing vitamin D trials identified by Roth et al,^9^ of which more than half are now published, and other newly published trial results not anticipated in the 5 years since the 2017 Roth et al publication. The objective of our review was to update the 2017 meta-analysis by Roth et al and present the impact of prenatal vitamin D supplementation on maternal, neonatal, and infant outcomes. This way, we may better inform health care practices and recommendations for public health policy.

## METHODS

We updated the review published by Roth et al in 2017,^9^ drawing our methods from the previous meta-analysis. The protocol was registered in the International Prospective Register of Systematic Reviews (PROSPERO) database (CRD42022350057). We report results in accordance with the Preferred Reporting Items for Systematic Reviews and Meta-Analyses (PRISMA) guideline.^15^ We define our Population, Intervention, Comparator, and Outcome (PICO) criteria in Table 1.

**Table 1.** PICOS Criteria for the Inclusion of Studies

Parameter	Criterion
Population	Pregnant women at any trimester of pregnancy
Intervention	Vitamin D supplementation, including vitamin D alone, vitamin D with calcium, and vitamin D with other vitamin and/or minerals
Comparator	Placebo (or no intervention) or active control (vitamin D dose ≤600 IU d^–1^ or equivalent)
Outcomes	Three categories of outcomes: maternal, birth, and infant outcomes Maternal outcomes: preeclampsia, gestational hypertension, gestational diabetes mellitus, preterm labor, cesarean section delivery, maternal hospitalization, maternal hypercalcemia, maternal hypocalcemia, maternal hypercalciuria, maternal 25-hydroxyvitamin D concentration at or near delivery Birth outcomes: stillbirth, low birth weight, preterm birth, small for gestational age, congenital malformation, admission to neonatal intensive care unit, gestational age, infant birth weight, infant birth body length, infant birth head circumference, cord 25-hydroxyvitamin D concentration Infant outcomes: neonatal death; neonatal hypercalcemia; neonatal hypocalcemia; respiratory infections; upper respiratory tract infections; lower respiratory tract infections; asthma or recurrent or persistent wheeze by age 3 y; the following measurements at age 1 y: body weight, body height, head circumference, weight for age *z* score, height for age *z* score, and head circumference for age *z* score; neonatal bone mineral content, neonatal bone mineral density, infant bone mineral content, infant bone mineral density
Study design	Randomized controlled trials
Research question	What are the effects of vitamin D supplementation in pregnancy on maternal, birth, and infant health outcomes?

### Search strategy

We searched articles published from September 2017 through November 2023, following the latest search of the prior review.^9^ Databases searched include MEDLINE, PubMed, Europe PMC, Scopus, Cochrane Database of Systematic Reviews, Web of Science, and CINAHL. We developed the search strategy in consultation with a research librarian at The George Washington University Himmelfarb Health Sciences Library (Table S1). Finally, we manually checked the references and the ongoing trials listed in the prior review.^9^

### Inclusion criteria

We included full-text, peer-reviewed articles of RCTs only. We considered case reports, case study designs, or nonrandomized trials ineligible. We excluded preprints or data published only in abstract format. Finally, we excluded retracted publications and any subsequent studies from the same research group. We did not restrict the language of publication.

We included pregnant women enrolled within any trimester of pregnancy. Studies were eligible if one or more of the intervention arms included supplementing pregnant women with one of the following: vitamin D alone, vitamin D with calcium, or vitamin D with other vitamin and/or minerals. The intervention dosage needed to be greater than 600 IU d^–1^ if compared with active control but could be any dose if compared with placebo control. The US Institute of Medicine (now the National Academy of Medicine) has a recommended dietary allowance of 600 IU d^–1^ for children and adults.^16^ We converted nondaily doses to a daily dose (IU d^–1^) to assess eligibility and compare outcomes across studies. Either form of vitamin D was eligible for inclusion (ie, vitamin D_2_, ergocalciferol; vitamin D_3_, cholecalciferol). We included any administration route (ie, oral, intramuscular, subcutaneous, or intravenous). Frequency of supplementation could be regular (eg, daily, weekly, every 2 weeks, monthly) or bolus (eg, once or twice during pregnancy). The comparator group could be either placebo or an active control. Studies were eligible if at least one of the comparison arms included any of the following: placebo, no intervention, or an active control: vitamin D dose ≤600 IU d^–1^ or equivalent (eg, 4200 IU wk^–1^ is equivalent to 600 IU d^–1^). We considered a trial eligible if the intervention and control arms received the same dosage of cointervention with any other micronutrients.

### Study selection and data extraction 

Two reviewers (W.-C.Y. and R.C.) independently screened titles and abstracts to assess eligibility for a full-text review. A third reviewer (E.R.S.) resolved any conflicts. We used Covidence, a web-based software program, to manage the screening process.^17^ Two reviewers (W.-C.Y. and R.C.) extracted data independently using a standard data collection form and cross-checked that to ensure accuracy. We contacted authors to request data for any studies with unreported outcomes (Table S2). We combined the data set derived from our updated search with the previously generated database provided by Roth et al.^9^

### Outcomes

We assessed three categories of outcomes: maternal, birth, and infant outcomes. A list of outcomes and detailed definitions is given in Table S3. Maternal outcomes of interest included preeclampsia, gestational hypertension, GDM, preterm labor, cesarean section delivery, maternal hospitalization, hyper- or hypocalcemia, hypercalciuria, and maternal postintervention (at or near delivery; studies measuring status more than 1 month after delivery were excluded) 25(OH)D concentration (nmol/L). Dichotomous birth outcomes included stillbirth or intrauterine death, preterm birth, low birth weight infants, small-for-gestational age infants, congenital malformations, and admission to intensive care units. Continuous birth outcomes, such as gestational age (in weeks), birth weight (measured in grams), birth length (cm), birth head circumference (cm), and umbilical cord 25(OH)D concentration (nmol/L), were assessed. Infant outcomes included neonatal death, hyper- or hypocalcemia; respiratory infections, upper and lower respiratory tract infections, asthma, and persistent or recurrent wheeze by age 3 years; anthropometric measurements at age 1 year (weight, height, and head circumference) and corresponding *z* scores; and neonatal and infant body mineral content (in grams) and density (g/cm^2^).

### Risk of bias

Two reviewers (W.-C.Y. and R.C.) independently assessed risk of bias for each trial using the Cochrane Risk of Bias assessment tool for RCTs (RoB).^18^ A third reviewer (E.R.S) resolved any disagreements. We acknowledge the updated RoB2 tool; however, we used an older version of this tool to incorporate our findings with those reported by Roth et al in 2017.^9^^,^^18^^,^^19^ We used funnel plots and Egger’s test to assess publication bias for outcomes reported for at least 10 trials.

### Statistical analysis

The primary analysis included trials reporting outcomes consistent with our a priori definitions, and sensitivity analysis included all trials regardless of outcome definitions. Data were pooled by DerSimonian and Laird random-effects models and summarized by pooled risk ratio (RR) or mean difference (MD) with 95% CIs for dichotomous and continuous outcomes, respectively.^20^ We chose, a priori, to use a random effects model based on the assumed distribution of effect sizes expected across trials, given the heterogeneity of populations and intervention doses or regimens used in these studies. In the event of missing means or standard deviations (SD), we imputed values using the median and interquartile range (Table S4).^21^^,^^22^ In the event of trials with no events in either the control or intervention arm, we used a continuity correction by adding 0.5 to all cells.^23^ We omitted trials with zero events in both arms from meta-analysis.

For studies with multiple arms, we collapsed data from the intervention arms. We requested these collapsed data from the original authors. If this was not possible, we collapsed data using Cochrane’s formula for combining groups (Table S4).^24^ All analyses were completed using R statistical software (version 4.2.0; R Foundation for Statistical Computing, Vienna, Austria; packages: *Meta*, *metafor*).

### Subgroup analysis

We conducted subgroup analyses for outcomes reported by more than three trials using the following categories: intervention type, population type, vitamin D dose in intervention arm, administration frequency, trimester of intervention initiation, and maternal baseline 25(OH)D concentration. Intervention types were (1) type 1: vitamin D alone vs placebo or no intervention, (2) type 2: vitamin D and calcium and/or other vitamins and/or minerals vs no vitamin D and calcium and/or other vitamins and/or minerals, (3) type 3: vitamin D vs active control of up to 600 IU d^–1^, and (4) type 4: vitamin D and calcium and/or other vitamins and/or minerals vs active control of up to 600 IU d^–1^ and calcium and/or other vitamins and/or minerals. Population types were (1) general population and (2) populations with selected morbidities. The vitamin D doses in the intervention arm were (1) ≤600 IU d^–1^, (2) 600 to ≤2000 IU d^–1^, and (3) >2000 IU d^–1^. Administration frequency groups included: (1) regular (ie, daily, weekly, every 2 weeks, or monthly) or (2) bolus (eg, once, twice). The trimester of intervention initiation was first, second, or third trimester.

The subgroup of maternal baseline vitamin D status was determined by the population mean 25(OH)D concentration of the control group. Two subgroup analyses were performed using different thresholds: ≥50 nmol/L vs <50 nmol/L, and ≥30 nmol/L vs <30 nmol/L.

## RESULTS

### Included trials

**Figure 1.**
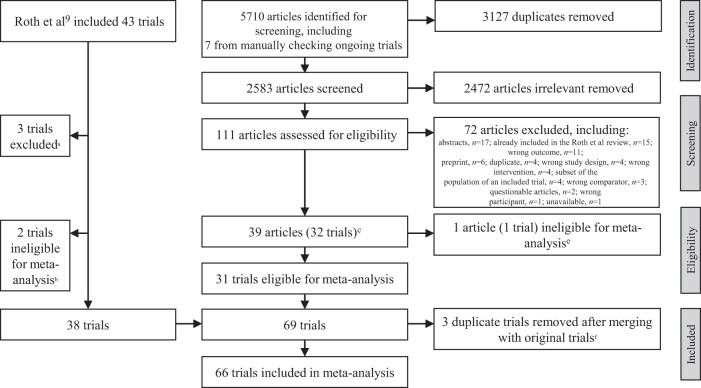
PRISMA diagram. ^a^Two articles were retracted by journals: Asemi et al (2013a) and Asemi et al (2013b); the article by Karamali et al (2015) was manually removed because Asemi was the corresponding author of Karamali et al (2015). ^b^Data from the Etemadifar et al (2015) and Zhang et al (2016) articles were ineligible for meta-analysis because they did not contribute to the analysis for any outcome of interest. ^c^(1) The Roth et al (2018) and Morris et al (2021) reports are from the same Maternal Vitamin D for Infant Growth trial (merged into Roth et al (2018)); (2) the Enkhmaa et al (2019) and Nasantogtokh et al (2023) report on the same trial (merged into Enkhmaa et al (2019)); (3) the Jefferson et al (2019), Powell et al (2019), and Khatiwada et al (2021) articles report data from the Kellogg trial (merged into Jefferson et al (2019)); (4) Brustad et al (2020), Sass et al (2020), and Brustad et al (2023) data are from the same trial (merged into Brustad et al (2020)); and (5) Corcoy et al (2020) and Harreiter et al (2022) data are from the same trial (merged into Corcoy et al (2020)). ^d^The Jamilian et al (2017) and Razavi et al (2017) articles were manually removed because they were from the research group that published articles that subsequently were retracted. ^e^The Bhowmik et al (2021) article was ineligible for meta-analysis because it did not contribute to the analysis for any outcome of interest. ^f^(1) The El-Heis et al (2022) and Moon et al (2023) data were merged with those of Cooper et al (2016) (MAVIDOS [Maternal Vitamin D Osteoporosis Study]), which were included in Roth et al review^9^; (2) the Brustad et al (2020) study data were merged with those of Chewas et al (2016) (COPSAC2010 [Copenhagen Prospective Studies on Asthma in Childhood] trial), which was also included in the Roth et al review.^9^

We screened 2583 articles and identified 32 trials, published since September 2017, eligible for systematic review (*n* = 31 were eligible for meta-analysis). Of the 43 articles Roth et al^9^ included in their review, we removed five. Two of those publications were formally retracted by the publishing journal, and we removed one publication with the same corresponding author as the retracted studies. In addition, two publications were ineligible for meta-analysis because their findings did not contribute to any of our outcomes of interest. We combined our trials with those reported by Roth et al and removed duplicates. In total, we analyzed 66 trials with 17 276 participants (Figure 1, Table S5).

### Characteristics of included trials

Two-thirds of the trials (*n* = 44 trials; 67%) were conducted in Asia, and one trial was conducted in Africa (1.5%). Most trials supplemented women regularly with vitamin D (*n* = 56 trials; 85%). Intervention doses ranged from 400 to 60 000 IU d^–1^ (median, 2000 IU d^–1^). Timing of supplementation varied, with almost one-third of trials (35%) initiating supplementation during the second trimester of pregnancy. More than half of the trials (*n* = 37 trials; 56%) used a placebo control. The number of participants randomized in a trial ranged from 16 to 2300 (median *n* = 140 participants). Most trials (*n* = 49; 74%) were conducted with generally healthy pregnant women; 17 trials (26%) were conducted among women with comorbidities. Ten trials (15.1%) were conducted with women with vitamin D deficiency (as defined by study investigators) or in combination with some other risk factors (eg, GDM, preeclampsia, hypocalcemia).^25^ Characteristics of the trials and participants are given in Table 2 and Table S6.

**Table 2.** Characteristics of Included Trials

Characteristic	**All trials** ^a^	**Placebo control trials** ^a^	**Active control trials** ^a^
No. of trials	66 (100)	37 (56.1)	29 (43.9)
No. of intervention arms			
2	51 (77.3)	31 (83.8)	20 (69.0)
3	14 (21.2)	5 (13.5)	9 (31.0)
4	0	0	0
5	1 (1.5)	1 (2.7)	0
No. of participants randomized			
Total no.	17 276	8857	8419
Median (range)	140 (16, 2300)	126 (34, 2300)	179 (16, 1720)
Meta-analyses to which each trial contributed			
No. of maternal outcomes, median (range)	2 (0 to 7)	1 (0 to 7)	3 (0 to 6)
No. of birth and infant outcomes, median (range)	2 (0 to 20)	2 (0 to 20)	3 (0 to 12)
No. of outcomes, median (range)	5 (1 to 26)	4 (1 to 26)	6 (1 to 17)
Geographic region			
Europe	10 (15.2)	7 (18.9)	3 (10.3)
Asia	44 (66.7)	26 (70.3)	18 (62.1)
North America	8 (12.1)	1 (2.7)	7 (24.1)
Africa	1 (1.5)	1 (2.7)	0
Australia/New Zealand/Oceania	3 (4.5)	2 (5.4)	1 (3.4)
Participant health status at enrollment			
Generally healthy	49 (74.2)	29 (78.4)	20 (69.0)
GDM/GDM risk factors^b^	6 (9.1)	4 (10.8)	2 (6.9)
Vitamin D deficiency^c^	6 (9.1)	1 (2.7)	5 (12.2)
Vitamin D deficiency + GDM/GDM risk factors	2 (3.0)	2 (5.4)	0
Vitamin D deficiency + preeclampsia risk factors	1 (1.5)	0	1 (3.4)
Vitamin D deficiency + hypocalcemia	1 (1.5)	0	1 (3.4)
HIV	1 (1.5)	1 (2.7)	0
Participants baseline 25(OH)D status^d^			
Mean 25(OH)D < 30 nmol/L	15 (28.3)	9 (33.3)	6 (23.1)
Mean 25(OH)D ≥ 30 nmol/L	37 (69.8)	18 (66.7)	19 (73.1)
Not reported	1 (1.9)	0	1 (3.8)
Type of 25(OH)D^e^			
D_2_	2 (3.0)	2 (5.4)	0
D_3_	57 (86.4)	33 (89.2)	24 (82.8)
D_2_ and D_3_	1 (1.5)	1 (2.7)	0
Not reported	6 (9.1)	1 (2.7)	5 (17.2)
Supplementation regularity			
Regularly	56 (84.8)	28 (75.7)	28 (96.6)
Bolus	6 (9.1)	5 (13.5)	1 (3.4)
Regular and bolus	3 (4.5)	3 (8.1)	0
Not reported	1 (1.5)	1 (2.7)	0
Supplementation frequency			
Daily	42 (63.6)	19 (51.4)	23 (79.3)
Weekly	6 (9.1)	4 (10.8)	2 (6.9)
Every 2 wk	5 (7.6)	4 (10.8)	1 (3.4)
Monthly	1 (1.5)	1 (2.7)	0
Every 2 mo	0	0	0
Once	1 (1.5)	1 (2.7)	0
Twice	3 (4.5)	2 (5.4)	1 (3.4)
Other^f^	7 (10.6)	5 (13.5)	2 (6.9)
Not reported	1 (1.5)	1 (2.7)	0
Intervention dose^g^			
Median (range) IU d^–1^^h^	2000 (400, 60 000)	2000 (400, 60 000)	2400 (600, 50 000)
Intervention dose ≤600 IU d^–1^, pairs	5 (7.0)	4 (11.8)	1 (2.7)
Intervention dose >600 IU d^–1^ and ≤2000 IU d^–1^, pairs	33 (46.5)	16 (47.1)	17 (45.9)
Intervention dose >2000 IU d^–1^, pairs	33 (46.5)	14 (41.2)	19 pairs (51.4)
Timing of initiation of supplementation^i^			
First trimester	14 (21.2)	5 (13.5)	9 (31.0)
Second trimester	23 (34.8)	16 (43.2)	7 (24.1)
Third trimester	4 (6.1)	3 (8.1)	1 (3.4)
Initiated in >1 trimester	24 (36.4)	12 (32.4)	12 (41.4)
Not reported	1 (1.5)	1 (2.7)	0

a Data are reported as no. (%) unless otherwise indicated.

b The Corcoy et al^28^ study was conducted with participants with prepregnancy body mass index ≥ 29, which was included in the group of GDM/GDM risk factors.

c Defined by trial investigators.

d Determined by the baseline maternal population mean 25(OH)D concentration of the control group.

e The Yu et al^39^ study had 3 arms: 1 intervention arm received vitamin D_2_, 1 received vitamin D_3_, and the other received no intervention.

f Valizadeh et al^40^ dosed daily and weekly; Rahbar et al^31^ dosed daily and every 2 wk; Mallet et al^41^ and Yu et al^39^ dosed daily and once; Sahoo et al^42^ dosed every 4 wk and every 8 wk; Sahu et al^43^ dosed once and twice; Sablok et al^44^ dosed once, twice, and 4 times.

g The number of intervention control pairs was used to report because 15 trials had multiple intervention arms. Only trials that administered vitamin D regularly are presented. Frequencies other than daily were converted to equivalent daily doses.

h Manasova et al^45^ gave participants vitamin D 4500 IU d^–1^ from gestational age (GA) 10–12 wk to GA 16 wk and then gave vitamin D 2500 IU d^–1^ from GA 16 wk to delivery. This study used 2500 IU d^–1^ to determine the dose of intervention.

i n = 24 trials initiated supplementation in >1 trimester.

*Abbreviations*: 25(OH)D, 25-hydroxyvitamin D; GDM, gestational diabetes mellitus.

### Risk of bias

The risk-of-bias assessment is detailed in Figure S1. A total of 25 trials (38%) had at least one domain at high risk of bias. Funnel plots and Egger’s test for outcomes reported by more than 10 trials are shown in Figure S2. We found relative symmetry in funnel plots for the following outcomes: GDM, cesarean section delivery, stillbirth (or intrauterine death), low-birth-weight infants, preterm birth, maternal 25(OH)D concentration near or at delivery, gestational age, birth weight, birth length, or cord 25(OH)D concentrations. The results of the Egger’s tests were not statistically significant at the α = 0.05 level.

### Main outcomes

We present primary analysis and subgroup analysis results in Table 3 and Table S7, respectively, and summarize both in the following paragraphs. Sensitivity analysis results are available in Table S8. Forest plots for all outcomes are provided in Figure S3. Figure S4 shows the trials that contributed to the meta-analysis for each outcome, stratified by primary and sensitivity analyses.

**Table 3.** Summary of Findings for the Primary Analysis.

Outcome	No. of trials	No. of participants	Risk ratio/mean difference (95% CI)	***I*** ^2^ **, % (*P* value)**
Maternal outcome				
Preeclampsia	6	1483	0.81 (0.43, 1.53)	41.7 (0.13)
Gestational hypertension	5	2262	1.23 (0.73, 2.07)	0.0 (0.92)
Gestational diabetes mellitus	12	1992	0.65 (0.49, 0.86)	22.6 (0.22)
Preterm labor	7	1276	0.70 (0.44, 1.11)	37.0 (0.15)
Cesarean section delivery^a^	26	7199	1.05 (0.99, 1.12)	0.0 (0.73)
Maternal hospitalization^b^	5	1776	0.74 (0.42, 1.32)	55.1 (0.06)
Maternal hypercalcemia^c^	1	175	3.10 (0.87, 11.08)	NA
Maternal hypocalcemia^d^	2	553	0.22 (0.01, 4.19)	93.9 (<0.01)
Maternal hypercalciuria^e^	3	1286	0.78 (0.15, 4.14)	0.0 (0.51)
Maternal 25(OH)D concentration at or near delivery (nmol/L)^f^	48	10 064	33.96 (28.16, 39.76)	97.6 (<0.01)
Birth outcome				
Stillbirth (or intrauterine death)	21	9186	0.82 (0.63, 1.07)	0.0 (0.60)
Low-birth-weight (<2500 g)^g^	13	5044	0.95 (0.76, 1.19)	27.5 (0.17)
Preterm birth (<37 wk)^h^	18	8446	1.01 (0.85, 1.19)	20.7 (0.21)
Small for gestational age (<10th percentile)	9	4211	0.92 (0.75, 1.13)	28.6 (0.19)
Congenital malformation^i^	6	4042	0.78 (0.49, 1.22)	32.6 (0.19)
Admission to neonatal intensive care unit	6	3236	1.02 (0.82, 1.27)	0.0 (0.83)
Gestational age (wk)	24	7305	–0.07 (–0.19, 0.05)	31.0 (0.08)
Birth weight (g)	40	9954	53.14 (16.48, 89.80)	65.1 (<0.01)
Birth body length (cm)	22	5261	0.24 (0.01, 0.47)	62.0 (<0.01)
Birth head circumference (cm)	20	5159	0.17 (0.02, 0.32)	63.2 (<0.01)
Cord 25(OH)D concentration (nmol/L)	26	4060	29.16 (21.87, 36.45)	97.6 (<0.01)
Neonatal and infant outcome				
Neonatal death^j^	5	4374	1.14 (0.70, 1.86)	4.3 (0.38)
Neonatal hypercalcemia	2	840	1.14 (0.39, 3.33)	0.0 (0.46)
Neonatal hypocalcemia^k^	1	126	0.10 (0.01, 1.83)	NA
Respiratory infections	2	1228	1.02 (0.93, 1.12)	0.0 (0.62)
Upper respiratory tract infections	2	389	0.93 (0.72, 1.21)	13.1 (0.28)
Lower respiratory tract infections	5	2080	0.98 (0.86, 1.11)	0.0 (0.78)
Asthma or recurrent or persistent wheeze by age 3 y	2	1387	0.81 (0.67, 0.98)	0.0 (0.96)
Body weight at age 1 y (g)	4	2707	61.06 (–174.11, 296.23)	80.8 (<0.01)
Body length at age 1 y (cm)	4	2923	0.42 (–0.40, 1.24)	89.5 (<0.01)
Head circumference at age 1 y (cm)	3	1171	–0.05 (–0.23, 0.13)	0.0 (0.59)
Weight for age *z* score at age 1 y	4	2642	–0.09 (–0.18, 0.00)	0.0 (0.42)
Length for age *z* score at age 1 y	4	2858	0.01 (–0.18, 0.17)	52.8 (0.10)
Head circumference for age *z* score at age 1 y	3	1106	–0.07 (–0.21, 0.07)	3.5 (0.35)
Neonatal bone mineral content (g)	2	690	1.09 (–0.64, 2.81)	0.0 (0.88)
Neonatal bone mineral density (g cm^–2^)	2	690	0.00 (–0.00, 0.00)	0.0 (0.90)
Infant bone mineral content (g)	1	52	–41.38 (–66.12, –16.64)	NA
Infant bone mineral density (g cm^–2^)	1	52	–0.05 (–0.06, –0.03)	NA

a n = 27 Trials reported cesarean section delivery; Delvin et al^46^ reported 0 events in both arms; data from 26 trials were used for meta-analysis.

b For the outcome of maternal admission, Pulido et al^47^ reported maternal admission due to glucose intolerance.

c n = 8 Trials reported the outcome maternal hypercalcemia; 7 trials, including Roth et al,^48^ Mutlu et al,^49^ Litonjua et al,^50^ Roth et al,^26^ Enkhmaa et al,^51^ Corcoy et al,^28^ and Sudfeld et al^27^ reported 0 events in both arms.

d n = 3 Trials reported the outcome maternal hypocalcemia; Mutlu et al^49^ reported 0 events in both arms.

e n = 4 Trials reported the outcome maternal hypercalciuria; Mutlu et al^49^ reported 0 events in both arms.

f n = 49 Trials reported this outcome. Valizadeh et al^40^ was excluded from primary analysis because its maternal post-intervention 25(OH)D was measured 6–12 wk after delivery. Data from 48 trials were used for meta-analysis.

g n = 14 Trials reported low-birth-weight infants; Hashemipour et al^52^ reported 0 events in both arms.

h n = 19 Trials reported preterm birth; Delvin et al^46^ reported 0 events in both arms.

i n = 7 Trials reported congenital malformation; Hashemipour et al^52^ reported 0 events in both arms.

j n = 7 Trials reported neonatal death; Hashemipour et al^52^ and Chawes et al^53^ reported 0 events in both arms.

k n = 2 Trials reported neonatal hypocalcemia; Roth et al^48^ reported 0 events in both arms.

*Abbreviations*: 25(OH)D, 25-hydroxyvitamin D; NA, not applicable.

#### Maternal outcomes

We found no effect of prenatal vitamin D supplementation on most maternal adverse outcomes, including gestational hypertension (RR, 1.23 [95% CI, 0.73-2.07]; *n* = 5 trials and 2262 participants), preterm labor (RR, 0.70 [95% CI, 0.44-1.11]; *n* = 7 trials and 1276 participants), cesarean section delivery (RR, 1.05 [95% CI, 0.99-1.12]; *n* = 26 trials and 7199 participants), and maternal hospitalization (RR, 0.74 [95% CI, 0.42-1.32]; *n* = 5 trials and 1776 participants).

##### Safety outcomes

We found no effect of prenatal vitamin D supplementation on maternal hypercalcemia (RR, 3.10 [95% CI, 0.87-11.08]; *n* = 1 trial and 175 participants), maternal hypocalcemia (RR, 0.22 [95% CI, 0.01-4.19]; *n* = 2 trials and 553 participants), and maternal hypercalciuria (RR, 0.78 [95% CI, 0.15-4.14; *n* = 3 trials and 1286 participants).

##### Gestational diabetes mellitus

We found that prenatal vitamin D supplementation decreased the risk of GDM by 35% (RR, 0.65 [95% CI, 0.49-0.86]; *n* = 12 trials and 1992 participants). In subgroup analysis, we found no effect on GDM when the analysis was limited to generally healthy pregnant women (RR, 0.74 [95% CI, 0.43-1.26]; *n* = 6 trials and 1283 participants).

##### Preeclampsia

In our primary analysis, we found no effect of vitamin D supplementation on the risk of preeclampsia (RR, 0.81 [95% CI, 0.43-1.53; *n* = 6 trials and 1483 participants). However, in our sensitivity analysis (Table S8), which included trials regardless of outcome definitions, we found a beneficial effect of supplementation on risk of preeclampsia (RR, 0.65 [95% CI, 0.49-0.86]; *n* = 22 trials and 4981 participants).

##### Maternal 25(OH)D concentration at or near delivery

Vitamin D supplementation in pregnancy increased postintervention maternal 25(OH)D concentration by an average of 34.0 nmol/L (95% CI, 28.16-39.76; *n* = 48 trials and 10 064 participants).

#### Birth outcomes

##### Umbilical cord 25(OH)D concentration

Antenatal supplementation with vitamin D increased the cord blood 25(OH)D concentration by an average of 29.2 nmol/L (95% CI, 21.9-36.5; *n* = 26 trials and 4060 participants).

##### Stillbirth or intrauterine death, preterm birth, and low-birth-weight infant

We found no effect of prenatal vitamin D supplementation on the risk of stillbirth or intrauterine death (RR, 0.82 [95% CI, 0.63-1.07; *n* = 21 trials and 9186 participants), preterm birth (RR, 1.01 [95% CI, 0.85-1.19; *n* = 18 trials and 8446 participants), and low-birth-weight infant (RR, 0.95 [95% CI, 0.76-1.19]; *n* = 13 trials and 5044 participants).

##### Anthropometric measurements at birth

On average, supplementing pregnant women with vitamin D increased infant birth weight by 53.14 g (95% CI, 16.48-89.80; *n* = 40 trials and 9954 participants), birth body length by 0.24 cm (95% CI, 0.01-0.47; *n* = 22 trials and 5261 participants), and birth head circumference by 0.17 cm (95% CI, 0.02-0.32; *n* = 20 trials and 5159 participants).

#### Infant outcomes

##### Anthropometric measurements in infancy (at 1 year of age)

Prenatal vitamin D supplementation did not affect infant body weight (MD, 61.06 g [95% CI, –174.11 to 296.23]; *n* = 4 trials and 2707 participants), body length (MD, 0.42 cm [95% CI, –0.40 to 1.24]; *n* = 4 trials and 2923 participants), or head circumference (MD, –0.05 cm, 95% CI, –0.23 to 0.13]; *n* = 3 trials and 1171 participants) at 1 year of age. There was no effect on weight-for-age *z* scores (MD, –0.09 [95% CI, –0.18 to 0.00]; *n* = 4 trials and 2642 participants), length-for-age *z* scores (MD, 0.01 [95% CI, –0.18 to 0.17]; *n* = 4 trials and 2858 participants), or head circumference-for-age *z* scores (MD, –0.07 [95% CI, –0.21 to 0.07]; *n* = 3 trials and 1106 participants) at 1 year of age. For findings on respiratory tract infections, asthma or wheeze, and bone mineral content and density for neonates and infants, see Table 3.

#### Subgroup analyses

Based on trial subgroups defined by intervention type, population type, intervention dose, administration frequency, vitamin D form, trimester at initiation, and maternal baseline 25(OH)D concentration, we found no evidence that the effect of vitamin D supplementation in pregnancy on maternal, birth, or infant outcomes differed (for subgroup heterogeneity, all *P* > 0.05) (Table S7, Figure S3).

## DISCUSSION

To our knowledge, this is the largest and most inclusive meta-analysis of vitamin D supplementation in pregnancy, with 66 trials and more than 17 000 participants. The potential benefits of vitamin D supplementation during pregnancy on maternal, infant, and child outcomes remain unclear, despite our inclusion of two new, adequately powered trials in Bangladesh and Tanzania.^26^^,^^27^ Most trials included in our meta-analysis had small study populations, were of low quality based on RoB assessment, and took place among generally healthy pregnant women. Studies were heterogeneous and included a variety of supplementation doses, administration frequency, and co-interventions. As a result, these study characteristics limited our conclusions, particularly regarding groups at high risk of vitamin D deficiency or those at risk of the outcome of interest.

We found vitamin D supplementation did not reduce the risk of any maternal morbidity outcomes except GDM. Our RR was similar to the magnitude found by Roth et al^9^ (RR, 0.65 [95% CI, 0.39-1.08; *n* = 5 trials and 1030 participants); however, Roth et al identified a statistically insignificant result. Our statistically significant result may be because we pooled data from 7 newly published trials along with the data reported by Roth et al^9^ and analyzed data from 12 trials.^28–34^ Of these 7 newly published trials, three were among women with GDM or GDM risk factors.^28^^,^^30^^,^^32^ Despite this, we are unable to draw conclusions that vitamin D supplementation benefits women, because most of the trials are small and among generally healthy women. In fact, those studies among women with GDM or GDM risk factors contributed only 400 participants, or just 20% of the data for that outcome.

We believe our primary analysis using a standardized outcome definition provided the highest quality summary estimate. Like Roth et al,^9^ we found a null effect of supplementation on preeclampsia in our primary analysis when we applied a strict outcome definition that required study-specific measurements of blood pressure, urine protein, and clinical assessment. In contrast, the 2019 Cochrane review by Palacios et al^10^ included 4 studies with any definition of preeclampsia and found a vitamin D supplementation led to a large reduction in preeclampsia. To investigate this, we performed sensitivity analysis using Cochrane’s methods and found a beneficial effect similar to what they reported. We caution readers against interpreting outcomes like preeclampsia or GDM in trials that did not use a standardized outcome definition, given the clinical complexity of diagnosing these conditions. Furthermore, these findings cannot be translated to populations with already established risk factors for such outcomes.

Consistent with this finding of a null effect on preeclampsia, we found no effect of supplementation on gestational hypertension. Therefore, the role of vitamin D supplementation in hypertensive disorders of pregnancy remains unclear. While in vitro studies provided evidence for a role for 1,25-dihydroxy vitamin D in regulation of placental activity, vitamin D supplementation may also affect hypertensive disorders of pregnancy by promoting calcium absorption.^3^ Relative to calcium as the comparator, few trials in our review were designed to test the combined effect of calcium and vitamin D on such outcomes; routine calcium supplementation is now the standard of care in many settings, thus more trials that co-administer both micronutrients may be warranted and may be particularly relevant for settings where vitamin D status and calcium intake are low.

We found that vitamin D supplementation increases mean birth weight. Our finding is consistent with those of prior reviews; however, our pooled effect is smaller compared with others (eg, Roth et al^9^ estimated an MD of 58 g; our estimate was 53 g). An explanation is the addition of data from eight new trials including more than 4000 participants. Although we found that supplementation was also associated with increased body length and head circumference at birth, the effect sizes were small relative to the SDs. Furthermore, beyond absolute increases in birth anthropometry, changes at the lower end of the distribution are generally more clinically meaningful. Given we found no benefits of supplementation on low birth weight infants, preterm birth, and small-for-gestational age, we cannot conclude that increases in birth size are particularly beneficial for infant health outcomes. Future analyses looking at more extreme adverse birth outcomes such as very low birth weight, moderate or severe preterm birth, and size for gestational age below the third percentile may provide more insight.

There are several limitations to our study, based on complexity of published studies and interpreting clinical trial quality and risk of bias. First, we included trials using active or placebo control for the comparator groups to maximize the amount of trial information included, whereas the 2019 Cochrane review included only trials with placebo control which provides a clearer comparison.^9^^,^^10^ Second, we found variation in the intervention between studies, including dose and frequency, limiting our ability to compare similar studies. To address this, we conducted subgroup analysis by comparator and intervention type, and the findings were similar. Careful interpretation is needed because some trials used intervention doses lower than 600 IU d^–1^, which is less than the current recommended daily allowance established by the Institute of Medicine, and some argue doses at these lower levels would not affect outcomes.^16^^,^^35^ However, we did not find evidence that the effect of vitamin D supplementation varied by dose in subgroup analyses. Third, the optimal 25(OH)D concentration in pregnancy remains unknown and may vary by baseline health status or outcome of interest. For example, some evidence suggests a higher 25(OH)D level might prevent prediabetic patients from becoming diabetic.^36^ Others have hypothesized that achieving higher 25(OH)D levels is needed to achieve extraskeletal benefits of vitamin D supplementation.^37^^,^^38^ Future meta-analyses might use individual patient data to investigate whether effects vary by the 25(OH)D concentrations achieved at or near delivery, particularly among women at high risk for certain outcomes. And future trials might test what supplemental vitamin D intake is needed to achieve these 25(OH)D concentrations. Furthermore, despite the large number of randomized trials on this topic, they vary in sample size and quality, which contributed to apparent inconsistency and heterogeneity. We included studies, regardless of their risk-of-bias assessment, and used an older version of the ROB tool to integrate our findings with that of Roth et al.^9^ Trials with a high or unclear risk of bias may limit our ability to provide clear interpretations of the evidence. We found only 21% (*n* = 14 trials) of the included trials had low risk of bias in all 7 domains. Lastly, multiple trials in this field have been retracted by journals due to potential research fraud. As a result, interpreting study quality and overall certainty of evidence is complex.

## CONCLUSION

Overall, there is no consistent evidence that vitamin D supplementation during pregnancy has clinically meaningful health benefits for pregnant women and infants in the general population. However, research in this field should focus on conducting adequately powered trials with high-quality assessment of maternal outcomes including GDM and hypertensive disorders of pregnancy. Our findings are consistent with the current World Health Organization guidelines, which do not recommend routine vitamin D supplementation in pregnancy.

## Supplementary Material

nuae065_Supplementary_Data

## Data Availability

The data set is available from the corresponding author upon request. Statistical code can be accessed at https://github.com/SmithLabGWSPH/VitaminDSystematicReview.
