# Supplementary material for: The Effects of Vitamin D Supplementation During Pregnancy on Maternal, Neonatal, and Infant Health: A Systematic Review and Meta-analysis
Source: Nutr Rev. 2024 Jul 1;83(3):e892–903. doi: 10.1093/nutrit/nuae065 (PMC11819489; doi:10.1093/nutrit/nuae065)
Supplement: nuae065_Supplementary_Data [file nuae065_supplementary_data.zip › nuae065_Supplementary_Data/S1 supplementary file 2024.06.28.docx]

**The effects of vitamin D supplementation during pregnancy on maternal, neonatal, and infant health: a systematic review and meta-analysis**

Wen-Chien Yang, Ramaa Chitale, Karen M. O’Callaghan, Christopher R. Sudfeld, Emily R. Smith

**Supplementary file**

Correspondence to: Emily R. Smith, ScD, MPH [emilysmith@gwu.edu](mailto:emilysmith@gwu.edu)

| **Contents** | | page |
| --- | --- | --- |
| **Table S1** | Search strategies for databases……………………………………………….………………………………………...... | 3 |
| **Table S2** | Outcome data requested and obtained by contacting authors of included trials………………………………………..... | 8 |
| **Table S3** | Outcome definitions……………………………………………………………………………………………………… | 11 |
| **Table S4** | Outcome data from imputation…………………………………………………………………………………………... | 13 |
| **Table S5** | References of included trials…………………………………………………………………………………………….. | 15 |
| **Table S6** | Description of included trials…………………………………………………………………………………………..… | 22 |
| **Table S7** | Subgroup analyses.……………………………………………………………………………………………………….. | 26 |
| **Figure S1** | Risk of bias assessment for included trials……………………………………………………………………………….. | 34 |
| **Figure S2** | Funnel plots and Egger's tests (for outcomes with at least 10 trials) |  |
|  | Gestational diabetes ………..…………………………………………………………………………………………….. | 35 |
|  | Cesarean delivery……………………………………………………………………………………… ………………... | 36 |
|  | Stillbirth or intrauterine death …………………………………………………………………………………………… | 37 |
|  | Low birthweight infant…………………………………………………………………………………………………… | 38 |
|  | Preterm birth……………………………………………………………………………………………………………... | 39 |
|  | Maternal 25(OH)D concentration at or near delivery (nmol/L) ………………………………………………………..... | 40 |
|  | Gestational age (weeks) ……………………………………………………………………………………………...….. | 41 |
|  | Birthweight (g).………………………………………………………………………………………….……………….. | 42 |
|  | Birth body length (cm).………………………………………………………………………………………………….. | 43 |
|  | Birth head circumference (cm)……….………………………………………………………………………………….. | 44 |
|  | Cord 25(OH)D concentration (nmol/L) …………………………………………………………………………………. | 45 |
| **Table S8** | Summary of findings for sensitivity analyses……………………………………………………………………………. | 46 |
| **Figure S3** | Forest plots for primary analyses, sensitivity analyses, and subgroup analyses………………………………………… | 47 |
|  | Preeclampsia……………………………………………………………………………………………………………… | 54 |
|  | Gestational hypertension…………………………………………………………………………………………………. | 64 |
|  | Gestational diabetes…………………………………………………………………………………………………..….. | 74 |
|  | Preterm labor……………………………………………………………………………………………………………... | 85 |
|  | Cesarean delivery………………………………………………………………………………………………………… | 94 |
|  | Maternal hospitalization………………………………………………………………………………………………...... | 103 |
|  | Maternal hypercalcemia……………………………………………………………………………………….…………. | 112 |
|  | Maternal hypocalcemia…………………………………………………………………………………………………... | 114 |
|  | Maternal hypercalciuria………………………………………………………………………………………………….. | 116 |
|  | Maternal 25(OH)D concentration at or near delivery (nmol/L) …………………………………………………………. | 118 |
|  | Stillbirth or intrauterine death……………………………………………………………………………………………. | 127 |
|  | Low birthweight infant (birthweight<2500g) ….………………………………………………………………………... | 136 |
|  | Preterm birth (<37 weeks) ………………...…………………………………………………………………………....... | 145 |
|  | Small for gestational age (birthweight<10th percentile) ……………………………………………………………........ | 154 |
|  | Congenital malformations………………………………………………………………………………………………... | 163 |
|  | Admission to neonatal intensive care unit (NICU)………………………………………………………………………. | 172 |
|  | Gestational age (weeks) ………………………………………………………………………………………………..... | 181 |
|  | Birthweight (g) ………………………………………………………………………………………………………….. | 190 |
|  | Birth body length (cm) ………………………………………………………………………………………………….. | 199 |
|  | Birth head circumference (cm) ………………………………………………………………………………………….. | 208 |
|  | Cord 25(OH)D concentration (nmol/L) …………………………………………………………………………………. | 217 |
|  | Neonatal death…………………………………………………………………………………………………………… | 226 |
|  | Neonatal hypercalcemia………………………………………………………………………………………………..... | 235 |
|  | Neonatal hypocalcemia………………………………………………………………………………………………….. | 237 |
|  | Respiratory infection…………………………………………………………………………………………………….. | 239 |
|  | Upper respiratory tract infection…………………………………………………………………………………………. | 240 |
|  | Lower respiratory tract infection………………………………………………………………………………………… | 241 |
|  | Asthma or recurrent/persistent wheeze by 3 y/o…………………………………………………………………………. | 250 |
|  | Infant body weight at 1 year old (g) ………….………………………………………………………………………….. | 252 |
|  | Infant body length at 1 year old (cm) ………..…………………………………………………………………………... | 261 |
|  | Infant head circumference at 1 year old (cm) ………………………………………………………………………….. | 270 |
|  | Weight for age z score at 1 year old.………………………………………………….………………………………….. | 271 |
|  | Length for age z score at 1 year old……………………………………………………………………………………… | 280 |
|  | Head circumference for age z score at 1 year old ……………………………………………………………………...... | 289 |
|  | Neonatal bone mineral content (g) ………………………………………………………………………………………. | 290 |
|  | Neonatal bone mineral density (g/cm^2^) …………………………………………………………………………………. | 291 |
|  | Infant bone mineral content (g) ………………………………………………………………………………………….. | 292 |
|  | Infant bone mineral density (g/cm^2^) ……………………………………………………………………………………... | 293 |
| **Figure S4** | The contribution of trials to primary and sensitivity analyses……………………………………………….................... | 294 |

**Table S1. Search strategies for databases**

| **MEDLINE** | |
| --- | --- |
| 1 | (Randomized Controlled Trial or Controlled Clinical Trial or Pragmatic Clinical Trial or Equivalence Trial or Clinical Trial, Phase III).pt. |
| 2 | Randomized Controlled Trial/ |
| 3 | exp Randomized Controlled Trials as Topic/ |
| 4 | "Randomized Controlled Trial (topic)"/ |
| 5 | Controlled Clinical Trial/ |
| 6 | exp Controlled Clinical Trials as Topic/ |
| 7 | "Controlled Clinical Trial (topic)"/ |
| 8 | Randomization/ |
| 9 | Random Allocation/ |
| 10 | Double-Blind Method/ |
| 11 | Double Blind Procedure/ |
| 12 | Double-Blind Studies/ |
| 13 | Single-Blind Method/ |
| 14 | Single Blind Procedure/ |
| 15 | Single-Blind Studies/ |
| 16 | Placebos/ |
| 17 | Placebo/ |
| 18 | Control Groups/ |
| 19 | Control Group/ |
| 20 | (random* or sham or placebo*).ti,ab,hw,kf. |
| 21 | ((singl* or doubl*) adj (blind* or dumm* or mask*)).ti,ab,hw,kf. |
| 22 | ((tripl* or trebl*) adj (blind* or dumm* or mask*)).ti,ab,hw,kf. |
| 23 | (control* adj3 (study or studies or trial* or group*)).ti,ab,kf. |
| 24 | (Nonrandom* or non random* or non-random* or quasi-random* or quasirandom*).ti,ab,hw,kf. |
| 25 | allocated.ti,ab,hw. |
| 26 | ((open label or open-label) adj5 (study or studies or trial*)).ti,ab,hw,kf. |
| 27 | ((equivalence or superiority or non-inferiority or noninferiority) adj3 (study or studies or trial*)).ti,ab,hw,kf. |
| 28 | (pragmatic study or pragmatic studies).ti,ab,hw,kf. |
| 29 | ((pragmatic or practical) adj3 trial*).ti,ab,hw,kf. |
| 30 | ((quasiexperimental or quasi-experimental) adj3 (study or studies or trial*)).ti,ab,hw,kf. |
| 31 | (phase adj3 (III or "3") adj3 (study or studies or trial*)).ti,hw,kf. |
| 32 | or/1-31 |
| 33 | (Maternal or gestation* or pregnan* or parous or primipar* or multipar* or primigravid* or multigravid* or gestation* or gravidit* or parturi* or puerper* or peri-conception* or periconception* or perinatal* or peri natal* or prenatal* or pre natal* or antenatal* or ante natal* or postnatal* or post natal* or peripartum or peri partum or prepartum or pre partum or antepartum or ante partum or intrapartum or intra partum or postpartum or post partum or (expectant* adj2 mother*) or mother-to-be or mothers-to-be or (child adj1 bear*) or childbear* or ((before or after or vaginal or abdominal) adj2 delivery) or caesarean* or cesarean* or c-section* or childbirth or (child* adj5 (birth or delivery))).mp. |
| 34 | Pregnant Women/ or Postpartum Period/ or Prenatal Care/ or Pregnancy/ or Gravidity/ or Parity/ or exp Parturition/ or Pregnancy in Adolescence/ or exp Pregnancy Outcome/ or Pregnancy, High-Risk/ or exp Pregnancy, Multiple/ or Peripartum Period/ or exp Pregnancy Trimesters/ or Pregnancy Complications/ or Pregnancy, Prolonged/ or Obstetric Labor Complications/ or Puerperal Disorders/ or exp Delivery, Obstetric/ or exp Labor, Obstetric/ |
| 35 | 33 or 34 |
| 36 | ("25(OH)D" or cholecalciferol or ergocalciferol or calciferol or "25(OH)D2" or "25(OH)D3" or "hydroxy25(OH)D" or vitamin-d or vitamin-d2 or vitamin-d3 or "25 hydroxy25(OH)D" or "25 hydroxy25(OH)D2" or "25 hydroxy25(OH)D3" or "25-hydroxy25(OH)D" or "25-hydroxy-25(OH)D" or "25-hydroxy-25(OH)D2" or "25-hydroxy-25(OH)D3" or 25OHD or "25-OH-25(OH)D" or 25-OHD or calcidiol or calcifediol).mp. |
| 37 | 32 and 35 and 36 |
| 38 | 37 not (exp animals/ not humans.sh.) |
| 39 | (2017* or 2018* or 2019* or 202*).dt,ez,da. |
| 40 | 38 and 39 |
| **PubMed** | |
| 1 | ("Randomized Controlled Trial"[pt] OR "Controlled Clinical Trial"[pt] OR "Pragmatic Clinical Trial"[pt] OR "Equivalence Trial"[pt] OR "Clinical Trial, Phase III"[pt]) |
| 2 | "Randomized Controlled Trials as Topic"[Mesh] |
| 3 | "Controlled Clinical Trials as Topic"[Mesh] |
| 4 | "Random Allocation"[Mesh:no exp] |
| 5 | "Double-Blind Method"[Mesh:no exp] |
| 6 | "Single-Blind Method"[Mesh:no exp] |
| 7 | Placebos[Mesh:no exp] |
| 8 | "Control Groups"[Mesh:no exp] |
| 9 | (random*[tw] OR sham[tw] OR placebo*[tw]) |
| 10 | ((singl*[tw] OR doubl*[tw]) AND (blind*[tw] OR dumm*[tw] OR mask*[tw])) |
| 11 | ((tripl*[tw] OR trebl*[tw]) AND (blind*[tw] OR dumm*[tw] OR mask*[tw])) |
| 12 | (control*[tiab] AND (study[tiab] OR studies[tiab] OR trial*[tiab] OR group*[tiab])) |
| 13 | (Nonrandom*[tw] OR "non random*"[tw] OR non-random*[tw] OR quasi-random*[tw] OR quasirandom*[tw]) |
| 14 | allocated[tw] |
| 15 | (("open label"[tw] OR open-label[tw]) AND (study[tw] OR studies[tw] OR trial*[tw])) |
| 16 | ((equivalence[tw] OR superiority[tw] OR non-inferiority[tw] OR noninferiority[tw]) AND (study[tw] OR studies[tw] OR trial*[tw])) |
| 17 | ("pragmatic study"[tw] OR "pragmatic studies"[tw]) |
| 18 | ((pragmatic[tw] OR practical[tw]) AND trial*[tw]) |
| 19 | ((quasiexperimental[tw] OR quasi-experimental[tw]) AND (study[tw] OR studies[tw] OR trial*[tw])) |
| 20 | (phase[tw] AND (III[tw] OR 3[tw]) AND (study[tw] OR studies[tw] OR trial*[tw])) |
| 21 | #1 OR #2 OR #3 OR #4 OR #5 OR #6 OR #7 OR #8 OR #9 OR #10 OR #11 OR #12 OR #13 OR #14 OR #15 OR #16 OR #17 OR #18 OR #19 OR #20 |
| 22 | (Maternal[tw] OR gestation*[tw] OR pregnan*[tw] OR parous[tw] OR primipar*[tw] OR multipar*[tw] OR primigravid*[tw] OR multigravid*[tw] OR gestation*[tw] OR gravidit*[tw] OR parturi*[tw] OR puerper*[tw] OR peri-ceonption*[tw] OR periconception*[tw] OR perinatal*[tw] OR "peri natal*"[tw] OR prenatal*[tw] OR "pre natal*"[tw] OR antenatal*[tw] OR "ante natal*"[tw] OR postnatal*[tw] OR "post natal*"[tw] OR peripartum[tw] OR "peri partum"[tw] OR prepartum[tw] OR "pre partum"[tw] OR antepartum[tw] OR "ante partum"[tw] OR intrapartum[tw] OR "intra partum"[tw] OR postpartum[tw] OR "post partum"[tw] OR (expectant*[tw] AND mother*[tw]) OR mother-to-be[tw] OR mothers-to-be[tw] OR (child[tw] AND bear*[tw]) OR childbear*[tw] OR ((before[tw] OR after[tw] OR vaginal[tw] OR abdominal[tw]) AND delivery[tw]) OR caesarean*[tw] OR cesarean*[tw] OR c-section*[tw] OR childbirth[tw] OR (child*[tw] AND (birth[tw] OR delivery[tw]))) |
| 23 | "Pregnant Women"[Mesh:no exp] OR "Postpartum Period"[Mesh:no exp] OR "Prenatal Care"[Mesh:no exp] OR Pregnancy[Mesh:no exp] OR Gravidity[Mesh:no exp] OR Parity[Mesh:no exp] OR Parturition[Mesh] OR "Pregnancy in Adolescence"[Mesh:no exp] OR "Pregnancy Outcome"[Mesh] OR "Pregnancy, High-Risk"[Mesh:no exp] OR "Pregnancy, Multiple"[Mesh] OR "Peripartum Period"[Mesh:no exp] OR "Pregnancy Trimesters"[Mesh] OR "Pregnancy Complications"[Mesh:no exp] OR "Pregnancy, Prolonged"[Mesh:no exp] OR "Obstetric Labor Complications"[Mesh:no exp] OR "Puerperal Disorders"[Mesh:no exp] OR "Delivery, Obstetric"[Mesh] OR "Labor, Obstetric"[Mesh] |
| 24 | #22 OR #23 |
| 25 | ("25(OH)D"[tw] OR cholecalciferol[tw] OR ergocalciferol[tw] OR calciferol[tw] OR "25(OH)D2"[tw] OR "25(OH)D3"[tw] OR "hydroxy25(OH)D"[tw] OR vitamin-d[tw] OR vitamin-d2[tw] OR vitamin-d3[tw] OR "25 hydroxy25(OH)D"[tw] OR "25 hydroxy25(OH)D2"[tw] OR "25 hydroxy25(OH)D3"[tw] OR "25-hydroxy25(OH)D"[tw] OR "25-hydroxy-25(OH)D"[tw] OR "25-hydroxy-25(OH)D2"[tw] OR "25-hydroxy-25(OH)D3"[tw] OR 25OHD[tw] OR "25-OH-25(OH)D"[tw] OR 25-OHD[tw] OR calcidiol[tw] OR calcifediol[tw]) |
| 26 | #21 AND #24 AND #25 |
| 27 | 2017/09/01:3000/12/31[Date - Completion] OR 2017/09/01:3000/12/31[Date - Create] OR 2017/09/01:3000/12/31[Date - Entry] OR 2017/09/01:3000/12/31[Date - MeSH] |
| 28 | #26 AND #27 |
| 29 | (pubstatusaheadofprint OR publisher[sb] OR pubmednotmedline[sb]) |
| 30 | #28 AND #29 |
| **Europe PMC** | |
| 1 | (((PUB_TYPE:"Randomized Controlled Trial" OR PUB_TYPE:"Controlled Clinical Trial" OR PUB_TYPE:"Pragmatic Clinical Trial" OR PUB_TYPE:"Equivalence Trial" OR PUB_TYPE:"Clinical Trial, Phase III")) or (KW:"Randomized Controlled Trials as Topic") or (KW:"Controlled Clinical Trials as Topic") or (KW:"Random Allocation") OR (KW:"Double-Blind Method") OR (KW:"Single-Blind Method") OR (KW:Placebos) OR (KW:"Control Groups") OR (TITLE:(random* OR sham OR placebo*) OR ABSTRACT:(random* OR sham OR placebo*)) OR (TITLE:((singl* OR doubl*) AND (blind* OR dumm* OR mask*)) OR ABSTRACT:((singl* OR doubl*) AND (blind* OR dumm* OR mask*))) or (TITLE:((tripl* OR trebl*) AND (blind* OR dumm* OR mask*)) OR ABSTRACT:((tripl* OR trebl*) AND (blind* OR dumm* OR mask*))) OR (TITLE:(control* AND (study OR studies OR trial* OR group*)) OR ABSTRACT:(control* AND (study OR studies OR trial* OR group*))) OR (TITLE:(Nonrandom* OR "non random*" OR non-random* OR quasi-random* OR quasirandom*) OR ABSTRACT:(Nonrandom* OR "non random*" OR non-random* OR quasi-random* OR quasirandom*)) OR (TITLE:allocated OR ABSTRACT:allocated) OR (TITLE:(("open label" OR open-label) AND (study OR studies OR trial*)) OR ABSTRACT:(("open label" OR open-label) AND (study OR studies OR trial*))) OR (TITLE:((equivalence OR superiority OR non-inferiority OR noninferiority) AND (study OR studies OR trial*)) OR ABSTRACT:((equivalence OR superiority OR non-inferiority OR noninferiority) AND (study OR studies OR trial*))) OR (TITLE:("pragmatic study" OR "pragmatic studies") or ABSTRACT:("pragmatic study" OR "pragmatic studies")) OR (TITLE:((pragmatic OR practical) AND trial*) OR ABSTRACT:((pragmatic OR practical) AND trial*)) OR (TITLE:((quasiexperimental OR quasi-experimental) AND (study OR studies OR trial*)) OR ABSTRACT:((quasiexperimental OR quasi-experimental) AND (study OR studies OR trial*))) OR (TITLE:(phase AND (III OR 3) AND (study OR studies OR trial*)) OR ABSTRACT:(phase AND (III OR 3) AND (study OR studies OR trial*)))) AND (TITLE:(Maternal OR gestation* OR pregnan* OR parous OR primipar* OR multipar* OR primigravid* OR multigravid* OR gestation* OR gravidit* OR parturi* OR puerper* OR peri-ceonption* OR periconception* OR perinatal* OR "peri natal*" OR prenatal* OR "pre natal*" OR antenatal* OR "ante natal*" OR postnatal* OR "post natal*" OR peripartum OR "peri partum" OR prepartum OR "pre partum" OR antepartum OR "ante partum" OR intrapartum OR "intra partum" OR postpartum OR "post partum" OR (expectant* AND mother*) OR mother-to-be OR mothers-to-be OR (child AND bear*) OR childbear* OR ((before OR after OR vaginal OR abdominal) AND delivery) OR caesarean* OR cesarean* OR c-section* OR childbirth OR (child* AND (birth OR delivery))) OR ABSTRACT:(Maternal OR gestation* OR pregnan* OR parous OR primipar* OR multipar* OR primigravid* OR multigravid* OR gestation* OR gravidit* OR parturi* OR puerper* OR peri-ceonption* OR periconception* OR perinatal* OR "peri natal*" OR prenatal* OR "pre natal*" OR antenatal* OR "ante natal*" OR postnatal* OR "post natal*" OR peripartum OR "peri partum" OR prepartum OR "pre partum" OR antepartum OR "ante partum" OR intrapartum OR "intra partum" OR postpartum OR "post partum" OR (expectant* AND mother*) OR mother-to-be OR mothers-to-be OR (child AND bear*) OR childbear* OR ((before OR after OR vaginal OR abdominal) AND delivery) OR caesarean* OR cesarean* OR c-section* OR childbirth OR (child* AND (birth OR delivery))) OR KW:("Pregnant Women" OR "Postpartum Period" OR "Prenatal Care" OR Pregnancy OR Gravidity OR Parity OR Parturition OR "Pregnancy in Adolescence" OR "Pregnancy Outcome" OR "Pregnancy, High-Risk" OR "Pregnancy, Multiple" OR "Peripartum Period" OR "Pregnancy Trimesters" OR "Pregnancy Complications" OR "Pregnancy, Prolonged" OR "Obstetric Labor Complications" OR "Puerperal Disorders" OR "Delivery, Obstetric" OR "Labor, Obstetric")) AND (TITLE:("25(OH)D" OR cholecalciferol OR ergocalciferol OR calciferol OR "25(OH)D2" OR "25(OH)D3" OR "hydroxy25(OH)D" OR vitamin-d OR vitamin-d2 OR vitamin-d3 OR "25 hydroxy25(OH)D" OR "25 hydroxy25(OH)D2" OR "25 hydroxy25(OH)D3" OR "25-hydroxy25(OH)D" OR "25-hydroxy-25(OH)D" OR "25-hydroxy-25(OH)D2" OR "25-hydroxy-25(OH)D3" OR 25OHD OR "25-OH-25(OH)D" OR 25-OHD OR calcidiol OR calcifediol) OR ABSTRACT:("25(OH)D" OR cholecalciferol OR ergocalciferol OR calciferol OR "25(OH)D2" OR "25(OH)D3" OR "hydroxy25(OH)D" OR vitamin-d OR vitamin-d2 OR vitamin-d3 OR "25 hydroxy25(OH)D" OR "25 hydroxy25(OH)D2" OR "25 hydroxy25(OH)D3" OR "25-hydroxy25(OH)D" OR "25-hydroxy-25(OH)D" OR "25-hydroxy-25(OH)D2" OR "25-hydroxy-25(OH)D3" OR 25OHD OR "25-OH-25(OH)D" OR 25-OHD OR calcidiol OR calcifediol)) NOT SRC:MED AND (FIRST_PDATE:[2017 TO 2023]) |
| **Scopus** | |
|  | ( ( ( ( TITLE-ABS ( "Randomized Controlled Trial" OR "Controlled Clinical Trial" OR "Pragmatic Clinical Trial" OR "Equivalence Trial" OR "Clinical Trial, Phase III" ) ) OR ( KEY ( "Randomized Controlled Trial" ) OR KEY ( "Randomized Controlled Trials" ) OR KEY ( "Controlled Clinical Trial" ) OR KEY ( "Controlled Clinical Trials" ) OR KEY ( randomization ) OR KEY ( "Random Allocation" ) OR KEY ( "Double-Blind" ) OR KEY ( "Single-Blind" ) OR KEY ( placebo* ) OR KEY ( "Control Groups" ) OR KEY ( "Control Group" ) ) OR ( TITLE-ABS ( random* OR sham OR placebo* ) ) OR ( TITLE-ABS ( ( singl* OR doubl* ) W/1 ( blind* OR dumm* OR mask* ) ) ) OR ( TITLE-ABS ( ( tripl* OR trebl* ) W/1 ( blind* OR dumm* OR mask* ) ) ) OR ( TITLE-ABS ( control* W/3 ( study OR studies OR trial* OR group* ) ) ) OR ( TITLE-ABS ( nonrandom* OR "non random*" OR non-random* OR quasi-random* OR quasirandom* ) ) OR ( TITLE-ABS ( allocated ) ) OR ( TITLE-ABS ( ( "open label" OR open-label ) W/5 ( study OR studies OR trial* ) ) ) OR ( TITLE-ABS ( ( equivalence OR superiority OR non-inferiority OR noninferiority ) W/3 ( study OR studies OR trial* ) ) ) OR ( TITLE-ABS ( "pragmatic study" OR "pragmatic studies" ) ) OR ( TITLE-ABS ( ( pragmatic OR practical ) W/3 trial* ) ) OR ( TITLE-ABS ( ( quasiexperimental OR quasi-experimental ) W/3 ( study OR studies OR trial* ) ) ) OR ( TITLE ( phase W/3 ( iii OR 3 ) W/3 ( study OR studies OR trial* ) ) OR KEY ( phase W/3 ( iii OR 3 ) W/3 ( study OR studies OR trial* ) ) ) ) ) AND ( ( ( TITLE-ABS ( Maternal OR gestation* OR pregnan* OR parous OR primipar* OR multipar* OR primigravid* OR multigravid* OR gestation* OR gravidit* OR parturi* OR puerper* OR peri-ceonption* OR periconception* OR perinatal* OR "peri natal*" OR prenatal* OR "pre natal*" OR antenatal* OR "ante natal*" OR postnatal* OR "post natal*" OR peripartum OR "peri partum" OR prepartum OR "pre partum" OR antepartum OR "ante partum" OR intrapartum OR "intra partum" OR postpartum OR "post partum" OR ( expectant* W/2 mother* ) OR mother-to-be OR mothers-to-be OR ( child W/1 bear* ) OR childbear* OR ( ( before OR after OR vaginal OR abdominal ) W/2 delivery ) OR caesarean* OR cesarean* OR c-section* OR childbirth OR ( child* W/5 ( birth OR delivery ) ) ) ) OR ( KEY ( "Pregnant Women" ) OR KEY ( "Postpartum Period" ) OR KEY ( "Prenatal Care" ) OR KEY ( pregnancy ) OR KEY ( gravidity ) OR KEY ( parity ) OR KEY ( parturition ) OR KEY ( "Pregnancy in Adolescence" ) OR KEY ( "Pregnancy Outcome" ) OR KEY ( "Pregnancy, High-Risk" ) OR KEY ( "Pregnancy, Multiple" ) OR KEY ( "Peripartum Period" ) OR KEY ( "Pregnancy Trimesters" ) OR KEY ( "Pregnancy Complications" ) OR KEY ( "Pregnancy, Prolonged" ) OR KEY ( "Obstetric Labor Complications" ) OR KEY ( "Puerperal Disorders" ) OR KEY ( "Delivery, Obstetric" ) OR KEY ( "Labor, Obstetric" ) ) ) ) AND ( ( TITLE-ABS ( "25(OH)D" OR cholecalciferol OR ergocalciferol OR calciferol OR "25(OH)D2" OR "25(OH)D3" OR "hydroxy25(OH)D" OR vitamin-d OR vitamin-d2 OR vitamin-d3 OR "25 hydroxy25(OH)D" OR "25 hydroxy25(OH)D2" OR "25 hydroxy25(OH)D3" OR "25-hydroxy25(OH)D" OR "25-hydroxy-25(OH)D" OR "25-hydroxy-25(OH)D2" OR "25-hydroxy-25(OH)D3" OR 25ohd OR "25-OH-25(OH)D" OR 25-ohd OR calcidiol OR calcifediol ) ) ) AND ( ( PUBYEAR > 2016 ) ) ) AND NOT ( TITLE ( ( animal OR animals OR canine* OR dog OR dogs OR feline OR hamster* OR lamb OR lambs OR mice OR monkey OR monkeys OR mouse OR murine OR pig OR pigs OR piglet* OR porcine OR primate* OR rabbit* OR rats OR rat OR rodent* OR sheep* ) AND NOT ( human* OR patient* ) ) OR KEY ( ( animals OR ( animal W/3 experiment* ) OR ( model* W/3 animal* ) OR nonhuman OR vertebrate* ) AND NOT ( humans OR ( human W/3 experiment* ) ) ) ) |
| **Cochrane** | |
| 1 | MeSH descriptor: [Pregnant Women] explode all trees |
| 2 | MeSH descriptor: [Postpartum Period] explode all trees |
| 3 | MeSH descriptor: [Prenatal Care] explode all trees |
| 4 | MeSH descriptor: [Pregnancy] explode all trees |
| 5 | MeSH descriptor: [Peripartum Period] explode all trees |
| 6 | MeSH descriptor: [Pregnancy Trimesters] explode all trees |
| 7 | MeSH descriptor: [Pregnancy Complications] explode all trees |
| 8 | MeSH descriptor: [Delivery, Obstetric] explode all trees |
| 9 | (Maternal OR gestation* OR pregnan* OR parous OR primipar* OR multipar* OR primigravid* OR multigravid* OR gestation* OR gravidit* OR parturi* OR puerper* OR peri-ceonption* OR periconception* OR perinatal* OR "peri natal*" OR prenatal* OR "pre natal*" OR antenatal* OR "ante natal*" OR postnatal* OR "post natal*" OR peripartum OR "peri partum" OR prepartum OR "pre partum" OR antepartum OR "ante partum" OR intrapartum OR "intra partum" OR postpartum OR "post partum" OR ( expectant* NEAR/2 mother* ) OR mother-to-be OR mothers-to-be OR ( child NEAR/1 bear* ) OR childbear* OR ( ( before OR after OR vaginal OR abdominal ) NEAR/2 delivery ) OR caesarean* OR cesarean* OR c-section* OR childbirth OR ( child* NEAR/5 ( birth OR delivery ) )):kw |
| 10 | (Maternal OR gestation* OR pregnan* OR parous OR primipar* OR multipar* OR primigravid* OR multigravid* OR gestation* OR gravidit* OR parturi* OR puerper* OR peri-ceonption* OR periconception* OR perinatal* OR "peri natal*" OR prenatal* OR "pre natal*" OR antenatal* OR "ante natal*" OR postnatal* OR "post natal*" OR peripartum OR "peri partum" OR prepartum OR "pre partum" OR antepartum OR "ante partum" OR intrapartum OR "intra partum" OR postpartum OR "post partum" OR ( expectant* NEAR/2 mother* ) OR mother-to-be OR mothers-to-be OR ( child NEAR/1 bear* ) OR childbear* OR ( ( before OR after OR vaginal OR abdominal ) NEAR/2 delivery ) OR caesarean* OR cesarean* OR c-section* OR childbirth OR ( child* NEAR/5 ( birth OR delivery ) )):ti |
| 11 | #1 OR #2 OR #3 OR #4 OR #5 OR #6 OR #7 OR #8 OR #9 OR #10 |
| 12 | (25(OH)D OR cholecalciferol OR ergocalciferol OR calciferol OR 25(OH)D2 OR 25(OH)D3 OR hydroxy25(OH)D OR 25 hydroxy25(OH)D OR 25 hydroxy25(OH)D2 OR 25 hydroxy25(OH)D3 OR 25ohd OR 25 OH 25(OH)D OR calcidiol OR calcifediol):ti,ab,kw |
| 13 | #11 AND #12 |
| 14 | with Publication Year from 2017 to 2022, in Trials |
| **Web of Science** | |
| 1 | TI=("Randomized Controlled Trial" OR "Controlled Clinical Trial" OR "Pragmatic Clinical Trial" OR "Equivalence Trial" OR "Clinical Trial, Phase III") OR AB=("Randomized Controlled Trial" OR "Controlled Clinical Trial" OR "Pragmatic Clinical Trial" OR "Equivalence Trial" OR "Clinical Trial, Phase III" ) OR TS=( "Randomized Controlled Trial" OR "Randomized Controlled Trial" OR "Randomized Controlled Trials" OR "Controlled Clinical Trial" OR "Controlled Clinical Trials" OR randomization OR "Random Allocation" OR "Double-Blind" OR "Single-Blind" OR placebo* OR "Control Groups" OR "Control Group") OR TI=( random* OR sham OR placebo* ) OR AB=( random* OR sham OR placebo* ) OR TI=( ( singl* OR doubl* ) NEAR/1 ( blind* OR dumm* OR mask* ) ) OR AB=( ( singl* OR doubl* ) NEAR/1 ( blind* OR dumm* OR mask* ) ) OR TI=( ( tripl* OR trebl* ) NEAR/1 ( blind* OR dumm* OR mask* ) ) OR AB=( ( tripl* OR trebl* ) NEAR/1 ( blind* OR dumm* OR mask* ) ) OR TI=( control* NEAR/3 ( study OR studies OR trial* OR group* ) ) OR AB=( control* NEAR/3 ( study OR studies OR trial* OR group* ) ) OR TI=( nonrandom* OR "non random*" OR non-random* OR quasi-random* OR quasirandom* ) OR AB=( nonrandom* OR "non random*" OR non-random* OR quasi-random* OR quasirandom* ) OR TI=(allocated ) OR AB=(allocated ) OR TI=(( "open label" OR open-label ) NEAR/5 ( study OR studies OR trial* )) OR AB=(( "open label" OR open-label ) NEAR/5 ( study OR studies OR trial* )) OR TI= ( ( equivalence OR superiority OR non-inferiority OR noninferiority ) NEAR/3 ( study OR studies OR trial* ) ) OR AB=(( equivalence OR superiority OR non-inferiority OR noninferiority ) NEAR/3 ( study OR studies OR trial* ) ) OR TI=( "pragmatic study" OR "pragmatic studies" ) OR AB=( "pragmatic study" OR "pragmatic studies" ) OR TI=( ( pragmatic OR practical ) NEAR/3 trial* ) OR AB=( ( pragmatic OR practical ) NEAR/3 trial* ) OR TI=( ( quasiexperimental OR quasi-experimental ) NEAR/3 ( study OR studies OR trial* )) OR AB=( ( quasiexperimental OR quasi-experimental ) NEAR/3 ( study OR studies OR trial* )) OR TI=( phase NEAR/3 ( iii OR 3 ) NEAR/3 ( study OR studies OR trial* ) ) OR TS=( phase NEAR/3 ( iii OR 3 ) NEAR/3 ( study OR studies OR trial* ) ) |
| 2 | TI=( Maternal OR gestation* OR pregnan* OR parous OR primipar* OR multipar* OR primigravid* OR multigravid* OR gestation* OR gravidit* OR parturi* OR puerper* OR peri-ceonption* OR periconception* OR perinatal* OR "peri natal*" OR prenatal* OR "pre natal*" OR antenatal* OR "ante natal*" OR postnatal* OR "post natal*" OR peripartum OR "peri partum" OR prepartum OR "pre partum" OR antepartum OR "ante partum" OR intrapartum OR "intra partum" OR postpartum OR "post partum" OR ( expectant* NEAR/2 mother* ) OR mother-to-be OR mothers-to-be OR ( child NEAR/1 bear* ) OR childbear* OR ( ( before OR after OR vaginal OR abdominal ) NEAR/2 delivery ) OR caesarean* OR cesarean* OR c-section* OR childbirth OR ( child* NEAR/5 ( birth OR delivery ) ) ) OR AB=( Maternal OR gestation* OR pregnan* OR parous OR primipar* OR multipar* OR primigravid* OR multigravid* OR gestation* OR gravidit* OR parturi* OR puerper* OR peri-ceonption* OR periconception* OR perinatal* OR "peri natal*" OR prenatal* OR "pre natal*" OR antenatal* OR "ante natal*" OR postnatal* OR "post natal*" OR peripartum OR "peri partum" OR prepartum OR "pre partum" OR antepartum OR "ante partum" OR intrapartum OR "intra partum" OR postpartum OR "post partum" OR ( expectant* NEAR/2 mother* ) OR mother-to-be OR mothers-to-be OR ( child NEAR/1 bear* ) OR childbear* OR ( ( before OR after OR vaginal OR abdominal ) NEAR/2 delivery ) OR caesarean* OR cesarean* OR c-section* OR childbirth OR ( child* NEAR/5 ( birth OR delivery ) ) ) OR TS= ("Pregnant Women" OR "Postpartum Period" OR "Prenatal Care" OR pregnancy OR gravidity OR parity OR parturition OR "Pregnancy in Adolescence" OR "Pregnancy Outcome" OR "Pregnancy, High-Risk" OR "Pregnancy, Multiple" OR "Peripartum Period" OR "Pregnancy Trimesters" OR "Pregnancy Complications" OR "Pregnancy, Prolonged" OR "Obstetric Labor Complications" OR "Puerperal Disorders" OR "Delivery, Obstetric" OR "Labor, Obstetric" ) |
| 3 | TS= ("25(OH)D" OR cholecalciferol OR ergocalciferol OR calciferol OR "25(OH)D2" OR "25(OH)D3" OR "hydroxy25(OH)D" OR vitamin-d OR vitamin-d2 OR vitamin-d3 OR "25 hydroxy25(OH)D" OR "25 hydroxy25(OH)D2" OR "25 hydroxy25(OH)D3" OR "25-hydroxy25(OH)D" OR "25-hydroxy-25(OH)D" OR "25-hydroxy-25(OH)D2" OR "25-hydroxy-25(OH)D3" OR 25ohd OR "25-OH-25(OH)D" OR 25-ohd OR calcidiol OR calcifediol) |
| 4 | LD=(2017-08-31/2022-08-04) |
| 5 | #4 AND #3 AND #2 AND #1 |
| **CINAHL** | |
| 1 | (((MH "Experimental Studies+") OR (MH "Multicenter Studies") OR (MH "Random Sample+") OR (MH "Placebos") OR (MH "Control (Research)+") OR (MH "Crossover Design") OR ((TI random* OR AB random*) OR (TI sham OR AB sham) OR (TI placebo* OR AB placebo*)) OR (((TI singl* OR AB singl*) OR (TI doubl* OR AB doubl*)) W1 ((TI blind* OR AB blind*) OR (TI dumm* OR AB dumm*) OR (TI mask* OR AB mask*))) OR (((TI tripl* OR AB tripl*) OR (TI trebl* OR AB trebl*)) W1 ((TI blind* OR AB blind*) OR (TI dumm* OR AB dumm*) OR (TI mask* OR AB mask*))) OR ((TI control* OR AB control*) N3 ((TI study OR AB study) OR (TI studies OR AB studies) OR (TI trial* OR AB trial*) OR (TI group* OR AB group*))) OR ((TI clinical OR AB clinical) N3 ((TI study OR AB study) OR (TI studies OR AB studies) OR (TI trial* OR AB trial*))) OR ((TI Nonrandom* OR AB Nonrandom*) OR (TI "non random*" OR AB "non random*") OR (TI "non-random*" OR AB "non-random*") OR (TI "quasi-random*" OR AB "quasi-random*") OR (TI quasirandom* OR AB quasirandom*)) OR ((TI phase OR AB phase) N3 ((TI study OR AB study) OR (TI studies OR AB studies) OR (TI trial* OR AB trial*))) OR (((TI crossover OR AB crossover) OR (TI "cross-over" OR AB "cross-over")) N3 ((TI study OR AB study) OR (TI studies OR AB studies) OR (TI trial* OR AB trial*))) OR (((TI multicent* OR AB multicent*) OR (TI "multi-cent*" OR AB "multi-cent*")) N3 ((TI study OR AB study) OR (TI studies OR AB studies) OR (TI trial* OR AB trial*))) OR (TI allocated OR AB allocated) OR (((TI "open label" OR AB "open label") OR (TI "open-label" OR AB "open-label")) N5 ((TI study OR AB study) OR (TI studies OR AB studies) OR (TI trial* OR AB trial*))) OR (((TI equivalence OR AB equivalence) OR (TI superiority OR AB superiority) OR (TI "non-inferiority" OR AB "non-inferiority") OR (TI noninferiority OR AB noninferiority)) N3 ((TI study OR AB study) OR (TI studies OR AB studies) OR (TI trial* OR AB trial*))) OR ((TI "pragmatic study" OR AB "pragmatic study") OR (TI "pragmatic studies" OR AB "pragmatic studies")) OR (((TI pragmatic OR AB pragmatic) OR (TI practical OR AB practical)) N3 (TI trial* OR AB trial*)) OR (((TI quasiexperimental OR AB quasiexperimental) OR (TI "quasi-experimental" OR AB "quasi-experimental")) N3 ((TI study OR AB study) OR (TI studies OR AB studies) OR (TI trial* OR AB trial*))) OR TI trial) |
| 2 | (TI (pregnan* or parous or primipar* or multipar* or primigravid* or multigravid* or gestation* or gravidit* or parturi* or puerper* or perinatal* or “peri natal*” or prenatal* or “pre natal*” or antenatal* or “ante natal*” or postnatal* or “post natal*” or peripartum or “peri partum” or prepartum or “pre partum” or antepartum or “ante partum” or intrapartum or “intra partum” or postpartum or “post partum” or (expectant* N1 mother*) or “mother-to-be” or “mothers-to-be” or (child N0 bear*) or childbear* or ((before or after or vaginal or abdominal) N1 delivery) or caesarean* or cesarean* or “c-section*” or childbirth or (child* N4 (birth or delivery))) OR AB (pregnan* or parous or primipar* or multipar* or primigravid* or multigravid* or gestation* or gravidit* or parturi* or puerper* or perinatal* or “peri natal*” or prenatal* or “pre natal*” or antenatal* or “ante natal*” or postnatal* or “post natal*” or peripartum or “peri partum” or prepartum or “pre partum” or antepartum or “ante partum” or intrapartum or “intra partum” or postpartum or “post partum” or (expectant* N1 mother*) or “mother-to-be” or “mothers-to-be” or (child N0 bear*) or childbear* or ((before or after or vaginal or abdominal) N1 delivery) or caesarean* or cesarean* or “c-section*” or childbirth or (child* N4 (birth or delivery)))) OR MH (Expectant Mothers OR Multiparas OR Primiparas OR Pregnancy+ OR Pregnancy, Multiple+ OR Pregnancy Trimesters+ OR Pregnancy, Unplanned OR Pregnancy, Unwanted OR Parity OR Prenatal Care OR Postnatal Care OR Perinatal Care OR Intrapartum Care+ OR Postnatal Period OR Puerperium OR Delivery, Obstetric OR Pregnancy Complications OR Labor Complications OR Pregnancy, Prolonged OR Puerperal Disorders) |
| 3 | TX ("25(OH)D" OR cholecalciferol OR ergocalciferol OR calciferol OR "25(OH)D2" OR "25(OH)D3" OR "hydroxy25(OH)D" OR vitamin-d OR vitamin-d2 OR vitamin-d3 OR "25 hydroxy25(OH)D" OR "25 hydroxy25(OH)D2" OR "25 hydroxy25(OH)D3" OR "25-hydroxy25(OH)D" OR "25-hydroxy-25(OH)D" OR "25-hydroxy-25(OH)D2" OR "25-hydroxy-25(OH)D3" OR 25OHD OR "25-OH-25(OH)D" OR 25-OHD OR calcidiol OR calcifediol)) |
| 4 | S1 AND S2 AND S3 |
| 5 | (MH "Vertebrates+") NOT MH Human) |
| 6 | S4 NOT S5 |
|  | ZD(201708* OR 201709* OR 201710* OR 201711* OR 201712* OR 2018* OR 2019* OR 2020* OR 2021* OR 2022* OR "in process") |
| 8 | S6 AND S7 |

**Table S2. Outcome data requested and obtained by contacting authors of included trials**

| **Trial** | **Outcome** | **Requested data** | |
| --- | --- | --- | --- |
|  |  | **Intervention group**  **N. of events/participants** | **Comparison group**  **N. of events/participants** |
| Best 2018 | Preeclampsia | 1/39 | 3/39 |
|  | Gestational hypertension | 1/39 | 1/39 |
| Roth 2018 | Neonatal death | 9/758 (collapsed group 2, 3, 4) | 4/247 (data of group 1) |
| **Trial** | **Outcome** | **Requested data** | |
|  |  | **Intervention group**  **mean (sd)**^a^  (collapsed intervention arms if multiple intervention arms) | **Comparison group**  **mean (sd)**^a^ |
| Hollis 2011 | Maternal baseline 25(OH)D concentration (nmol/L) | 57.4 (22.1), n=239 | available in the paper |
|  | Maternal 25(OH)D concentration at delivery (nmol/L) | 104.9 (37.4), n=239 | available in the paper |
|  | Cord 25(OH)D concentration (nmol/L) | 61.6 (25.5), n=239 | available in the paper |
|  | Birthweight (g) | 3323 (591), n=239 | available in the paper |
|  | Gestational age (weeks) | 38.9 (1.8), n=239 | available in the paper |
| Grant 2014 | Maternal baseline 25(OH)D concentration (nmol/L) | 66.4 (33.3), n=172 | 60.1 (38.5), n=87 |
|  | Maternal 25(OH)D concentration at delivery (nmol/L) | 100.1 (35.8), n=150 | 59.3 (42.2), n=78 |
|  | Cord 25(OH)D concentration (nmol/L) | 63.2 (23.4), n=137 | 36.5 (21.1), n=63 |
|  | Birthweight (g) | 3543.0 (584.4), n=162 | 3402.9 (684.4), n=82 |
|  | Gestational age (weeks) | 39.4 (1.3), n=162 | 38.9 (2.4), n=82 |
| March 2015 | Maternal 25(OH)D concentration at GA 24 weeks (nmol/L) | 87.7 (25.4) n=84 | available in the paper |
|  | Maternal 25(OH)D concentration at GA 36 weeks (nmol/L) | 98.4 (26.1) n=81 | available in the paper |
|  | Cord 25(OH)D concentration (nmol/L) | 47.3 (15.0) n=65 | available in the paper |
| O’Callaghan 2018 | Maternal baseline 25(OH)D concentration (nmol/L) | 53.7 (21.6) n=94 | available in the paper |
|  | Maternal 25(OH)D concentration at GA 24 weeks (nmol/L) | 87.7 (25.4) n=84 | available in the paper |
|  | Maternal 25(OH)D concentration at GA 36 weeks (nmol/L) | 98.4 (26.1) n=81 | available in the paper |
|  | Cord 25(OH)D concentration (nmol/L) | 47.3 (15.0) n=65 | available in the paper |
| Nausheen 2021 | Maternal baseline 25(OH)D concentration (ng/ml)^b^ | 9.1 (8.2) n=234 | available in the paper |
|  | Maternal 25(OH)D concentration at delivery (ng/ml)^b^ | 13.0 (8.7) n=165 | available in the paper |
|  | Cord 25(OH)D concentration (ng/ml) | 16.0 (12.7) n=147 | available in the paper |
| Sudfeld 2022 | Infant length at 1 year old (cm) | 70.7 (3.7) n=867 | 71.0 (3.7) n=872 |
|  | Infant weight at 1 year old (kg) | 9.0 (1.3) n=767 | 9.1 (1.3) n=758 |
| **Trial** | **Outcome** | **Requested data** | |
|  |  | **Intervention groups**  **mean (sd)**^a^ | **Comparison group**  **mean (sd)**^a^ |
| Roth 2018 | *Collapsed data of group 2, 3, 4, 5^c^* | | |
|  | Maternal baseline 25(OH)D concentration (nmol/L) | 27.5 (14.2) n=926 | available in the paper |
|  | Maternal 25(OH)D concentration at delivery (nmol/L) | 99.0 (29.8) n=507 | available in the paper |
|  | Birthweight (g) | 2713 (359) n=669 | available in the paper |
|  | Birth body length (cm) | 47.4 (2.0) n=660 | available in the paper |
|  | Birth head circumference (cm) | 33.0 (1.2) n=663 | available in the paper |
|  | Cord 25(OH)D concentration (nmol/L) | 59.4 (19.6) n=401 | available in the paper |
|  | Gestation age (weeks) | 39.0 (1.6) n=1007 | 38.9 (1.6) n=247 |
|  | *Collapsed data of group 2, group3, and group 4^d^* |  |  |
|  | Body length at 1 year old (cm) | 72.4 (2.7) n=704 | 72.6 (2.8) n=229 |
|  | Body length for age z score at 1 year old | -1.1 (1.1) n=704 | -0.9 (1.1) n=229 |
|  | Body weight at 1 year old (g) | 8446 (1139) n=702 | 8549 (1184) n=228 |
|  | Body weight for age z score at 1 year old | -0.9 (1.1) n=702 | -0.8 (1.1) n=228 |
|  | Head circumference at 1 year old (cm) | 43.9 (1.4) n=698 | 44 (1.4) n=225 |
|  | Head circumference for age z score at 1 year old | -1.2 (1.0) n=698 | -1.1 (1.0) n=225 |
|  | *Collapsed data of group 4 and group 5^e^* |  |  |
|  | Maternal baseline 25(OH)D concentration (nmol/L) | 26.8 (14.2), n=455 | available in the paper |
|  | Maternal 25(OH)D concentration at delivery (nmol/L) | 112.2 (26.8), n=250 | available in the paper |
|  | Birthweight (g) | 2713 (250), n=330 | available in the paper |
|  | Birth body length (cm) | 47.4 (2.0), n=325 | available in the paper |
|  | Birth head circumference (cm) | 32.9 (1.2), n=326 | available in the paper |
|  | Cord 25(OH)D concentration(nmol/L) | 70.8 (16.3), n=190 | available in the paper |
|  | Gestational age (weeks) | 39.0 (1.6), n=501 | 38.9 (1.7), n=123.5 |
|  | Body weight at 1 year old (g) | 8425 (1139), n=229 | available in the paper |
|  | Head circumference at 1 year old (cm) | 43.9 (1.4), n=229 | available in the paper |
|  | *Individual group data* |  |  |
|  | Gestational age (weeks): group 3 versus group 1 | 38.8 (1.8), n=252 | 38.9 (1.7), n=61.75 |
|  | Gestational age (weeks): group 2 versus group 1 | 39.1 (1.5), n=254 | 38.9 (1.7), n=61.75 |
|  | Body weight at 1 year old (g): group 3 versus group 1 | 8502 (1138), n=237 | available in the paper |
|  | Body weight at 1 year old (g): group 2 versus group 1 | 8411 (1142), n=236 | available in the paper |
|  | Head circumference at 1 year old (cm): group 3 versus group 1 | 43.9 (1.5), n=234 | available in the paper |
|  | Head circumference at 1 year old (cm): group 2 versus group 1 | 43.9 (1.4), n=235 | available in the paper |

^a^ sd, standard deviation

^b^ The requested data was provided in ng/ml; we converted the value to nmol/L for analysis.

^c^ Data of group 2 to group 5 were collapsed for outcomes that would not be affected by the postpartum supplementation in group 5.

^d^ Data of group 2 to group 4 were collapsed for outcomes that might be affected by the postpartum supplementation in group 5.

^e^ Data of group 4 and group 5 were collected to develop the intervention comparison pair for outcomes that would not be affected by the postpartum supplementation in group 5.

**Table S3. Outcome definitions (adopted from Roth et al. BMJ. 2017 Appendix 3)**

| **Maternal outcomes** | **Definition** *(used to guide development of criteria for content and method of ascertainment)* | **Criteria for content for case definition** | **Criteria for method of ascertainment of the outcome as reported in the description of the trial methods** | **Alternative definitions** |
| --- | --- | --- | --- | --- |
| Preeclampsia | Presence of high blood pressure and at least one other clinical complication such as proteinuria or low platelet count after 20 weeks gestation | Abnormal findings from: • Blood pressure readings after 20 weeks gestation • Urinary protein testing (e.g., urine dipstick) • +/- Clinical signs upon which diagnosis was made (e.g., maternal organ dysfunction) | All participants were scheduled to undergo routine blood pressure readings and urinary protein testing (at least in women with hypertension), as well as routine measurements of any other specified clinical and laboratory criteria upon which the diagnosis was based. | • Ad-hoc physician diagnosis • Passive surveillance (e.g., review of medical records) • Participant enrolment was limited to women with preeclampsia. |
| Gestational hypertension | Pregnancy induced hypertension characterized by high blood pressure (systolic ≥ 140 mmHg and/or diastolic ≥ 90 mmHg) after 20 weeks gestation without the presence of protein in the urine | Abnormal findings from: • Blood pressure readings • Urinary protein testing to verify the presence/absence of protein in the urine • +/- Weight gain monitoring | All participants were scheduled to undergo routine blood pressure readings and urinary protein testing if a positive BP reading was obtained. | • Ad-hoc physician diagnosis • Passive surveillance (e.g., review of medical records)  • Participant enrolment was limited to women with gestational hypertension |
| Gestational diabetes | Glucose intolerance that either begins or is first diagnosed during pregnancy. | Abnormal values from at least one of the following: • Glucose challenge test • Oral glucose tolerance test • Glycated hemoglobin (A1C) test | All participants were scheduled to undergo routine glucose challenge testing, oral glucose tolerance test, and/or HbA1c testing on at least one occasion after initiation of the study intervention. | • Ad-hoc physician diagnosis • Passive surveillance (e.g., review of medical records) • Diagnosis was made at the time of, or before, randomization. • Participant enrolment was limited to women with gestational diabetes. |
| Preterm labor | Regular contractions of the uterus that start before 37 weeks gestation that result in changes in the cervix. | No specified criteria | No specified criteria | - |
| Cesarean delivery | Delivery of a baby involving the surgical incision of the mother’s abdomen and uterus. Can be either elective or emergency. | No specified criteria | No specified criteria | - |
| Maternal hospitalization | Any hospitalization other than for an uncomplicated delivery. | No specified criteria | Outcome reporting based on prospective surveillance and/or hospitalization records | - |
| Maternal hypercalcemia (anytime) | Biochemical values outside of the normal range e.g., hypercalcemia, hypercalciuria, hypophosphatemia | Abnormal findings from laboratory testing e.g., serum calcium, phosphate etc. We did not apply universal criteria for cut-offs values, as these may differ across labs. | All participants underwent routine blood work screening for biochemical abnormalities at least once after first dose of supplement. | Passive surveillance (e.g., review of medical records) |
| Maternal Hypocalcemia (anytime) |  |  |  |  |
| Maternal hypercalciuria (anytime) |  |  |  |  |
| Maternal 25(OH)D concentration at or near delivery (nmol/L) | Post-intervention biochemical value in the third trimester or at delivery | No specified criteria | No specified criteria | - |
| **Birth outcomes** |  |  |  |  |
| Stillbirth or intrauterine death | Intrauterine fetal death refers to no signs of life in utero, while stillbirth refers to a baby with no signs of life upon delivery after 24 weeks gestation. | • No signs of life in utero • Delivery of baby with no signs of life | No specified criteria | - |
| Low birthweight (<2500g) | birthweight<2500g | The term “low birthweight” or LBW is used to label the outcome, or an outcome defined as birthweight<2500g (regardless of the label). | No specified criteria | - |
| Preterm birth (<37 weeks) | Birth that occurs between 20 weeks and 37 weeks gestation | No specified criteria | No specified criteria | - |
| Small for gestational age (SGA) | Baby born with weight <10th percentile for babies of the same (or similar) gestational age | The term “small-for-gestational age” or “SGA” is used to label the outcome. | No specified criteria | - |
| Congenital malformations | Major congenital anomalies/defects that are present at birth and have medical, surgical, or cosmetic significance. Can be identified prenatally or postnatally | No specified criteria | Diagnosis was ascertained by any of the following: • Parental/caregiver report • Medical records review • Physical examination by physician | - |
| Admission to intensive or special care unit within neonatal period | Admission to a neonatal intensive care unit (NICU) at a concentration 2 or concentration 3 hospital | No specified criteria | Outcome reporting based on hospitalization records and/or prospective surveillance | - |
| Gestational age (weeks) | - | No specified criteria | No specified criteria | - |
| Birthweight (g) | Anthropometry of an infant at birth  (e.g., length, weight, head  circumference) | Anthropometric measurements  collected at birth | No specified criteria | - |
| Birth body length (cm) |  |  |  |  |
| Birth head circumference (cm) |  |  |  |  |
| Cord 25(OH)D concentration (nmol/L) | - | Analysis of 25(OH)D from either maternal serum or plasma samples taken at or near delivery | Specified laboratory method of  determining circulating 25(OH)D  concentrations in serum or plasma samples | Unclear methods of determining  circulating 25(OH)D |
| **Neonatal and infant outcomes** |  |  |  |  |
| Neonatal death | Death within the neonatal period (i.e., first 28 days of life) | No specified criteria | No specified criteria | - |
| Neonatal hypercalcemia | Biochemical values outside of the normal range e.g., hypercalcemia, hypophosphatemia | Abnormal findings from laboratory testing e.g., serum calcium. We did not apply universal criteria for cut-offs values, as these may differ across labs | Trial authors stated that all participants  underwent routine blood work screening for biochemical abnormalities. | Passive surveillance (e.g., review of medical records) |
| Neonatal hypocalcemia |  |  |  |  |
| Respiratory infections by 3 y/o | Respiratory tract infections in general, upper or lower respiratory tract infection. | Any outcome based on clinical,  microbiological, and/or radiological features that suggest acute respiratory infection | Diagnosis was ascertained by ant of the following:  • Parental/caregiver report  • Study personnel case identification  based on established criteria  • Physician diagnosis  • Medical records revie | - |
| Upper respiratory tract infections by 3 y/o |  |  |  |  |
| Lower respiratory tract infections by 3 y/o |  |  |  |  |
| Asthma or recurrent/persistent wheeze by 3 y/o | Physician diagnosis of “asthma” and/or “recurrent wheeze” or “persistent wheeze” that reflects a respiratory condition involving  bronchospasm and/or airway edema  that is not solely attributable to an acute infection | Any outcome that is labeled as “asthma,” “reactive airway disease”, “wheeze” or other similar related terms | Prospective surveillance at least annually with scheduled Follow-up until at least 3 years of age | Asthma or wheeze outcomes based on  retrospective medical record review or cross-sectional interview |
| Weight at 1 year old (g) | Anthropometry of an infant at 1 year  (e.g., length, weight, head circumference) | Anthropometric measurements  collected at 1 year of age | No specified criteria | - |
| Length at 1 year old (cm) |  |  |  |  |
| Head circumference at 1 year old (cm) |  |  |  |  |
| Weight for age z score at 1 year old | The corresponding z scores of anthropometry of an infant at 1 year  (e.g., length, weight, head circumference) | The corresponding z scores of anthropometric measurements  collected at 1 year of age | No specified criteria | - |
| Length for age z score at 1 year old |  |  |  |  |
| Head circumference for age z score at 1 year old |  |  |  |  |
| Neonatal bone mineral content (g) | Bone densitometry as assessed by a  conventional method (e.g., dual energy  x-ray absorptiometry,  computed tomography, ultrasound). | Bone densitometry as assessed by a  conventional method (e.g., dual energy  x-ray absorptiometry,  computed tomography, ultrasound) | All (or a defined random sub-set of)  participants underwent bone densitometry. | - |
| Neonatal bone mineral density (g/cm^2^) |  |  |  |  |
| Infant bone mineral content (g) |  |  |  |  |
| Infant bone mineral density (g/cm^2^) |  |  |  |  |

**Table S4. Outcome data from imputation**

| **Trial** | **Outcome** | **Imputed outcome data** | | **Original outcome data** | |
| --- | --- | --- | --- | --- | --- |
|  |  | **Intervention mean (sd)**^a^ | **Control mean (sd)**^a^ | **Intervention median (IQR)**^b^ | **Control median (IQR)**^b^ |
| Aggarwal 2022 | Maternal baseline 25(OH)D concentration (nmol/L) | 20.8 (10.8) | 22.6 (13.3) | 19.47 (14.23-28.70) | 21.47 (14.23-31.95) |
| Pulido 2022 | Maternal baseline 25(OH)D concentration (nmol/L) | 61.6 (15.6) | 61.57 (15.6) | 59.90 (52.42-72.38) | 59.90 (52.42-72.38) |
|  | Maternal 25(OH)D concentration at delivery (nmol/L) | 78.2 (23.4) | 59.9 (23.4) | 79.87 (62.40-92.35) | 64.90 (42.43-72.38) |
| **Trial** | **Outcome** | **Imputed outcome data** | | **Original outcome data** | |
|  |  | **collapsed high and low dose groups mean (sd)**^a^ | | **high dose group  mean (sd)**^a^ | **low dose group mean (sd)**^a^ |
| Mallet 1986 | Birthweight (g) | 3280 (430), n=48 | | 3210(468), n=27 | 3370 (367), n=21 |
|  | Maternal 25(OH)D concentration at delivery (nmol/L) | 25.7 (6.9), n=48 | | 26.0(6.4), n=27 | 25.3 (7.7), n=21 |
|  | Cord 25(OH)D concentration (nmol/L) | 17.1 (5.3), n=48 | | 18.2 (5.2), n=27 | 15.7 (5.1), n=21 |
| Sahu 2009 | Maternal baseline 25(OH)D concentration (nmol/L) | 38.2 (21.2), n=70 | | 41.8 (23.3), n=35 | 34.6 (18.6), n=35 |
|  | Maternal 25(OH)D concentration at delivery (nmol/L) | 47.7 (30.2), n=70 | | 60.9 (34.7), n=35 | 34.6 (17.3), n=35 |
| Yu 2009 | Birthweight (g) | 3306 (496), n=108 | | 3290 (467), n=52 | 3321 (525), n=56 |
|  | Maternal baseline 25(OH)D concentration (nmol/L) | 28.5 (13.7), n=120 | | 29.3 (14.8), n=60 | 27.7 (12.6), n=60 |
|  | Maternal 25(OH)D concentration at delivery (nmol/L) | 43.2 (25.8), n=120 | | 36.7 (11.9), n=60 | 49.7 (33.3), n=60 |
|  | Cord 25(OH)D concentration (nmol/L) | 27.5 (16.9), n=120 | | 25.7(11.9), n=60 | 29.3 (20.7), n-60 |
|  | Gestational age (weeks) | 39.5 (1.7), n=108 | | 40.0 (1.0), n=52 | 39.0 (2.0), n=56 |
| Dawodu 2013 | Birthweight (g) | 3145 (571), n=107 | | 3102 (639), n=55 | 3191 (492), n=52 |
|  | Birth body length (cm) | 51.2 (3.3), n=106 | | 50.9 (3.7), n=54 | 51.60 (2.8), n=52 |
|  | Birth head circumference (cm) | 34.2 (2.1), n=107 | | 33.9 (2.2), n=55 | 34.5 (2.0) n=52 |
|  | Maternal baseline 25(OH)D concentration (nmol/L) | 20.1 (10.0), n=128 | | 19.6 (7.7), n=63 | 20.5 (11.9), n=65 |
|  | Maternal 25(OH)D concentration at delivery (nmol/L) | 77.5 (30.6), n=84 | | 89.7 (30.3), n=43 | 64.8 (25.5), n=41 |
|  | Cord 25(OH)D concentration (nmol/L) | 57.4 (22.9), n=81 | | 66.0 (20.8), n=42 | 48.2 (21.7), n=39 |
|  | Gestational age (weeks) | 38.8 (2.1), n=107 | | 38.6 (2.5), n=55 | 38.9 (1.7), n=52 |
| Soheilykhah 2013 | Maternal baseline 25(OH)D concentration (nmol/L) | 18.2 (13.9), n=78 | | 18.2 (14.7), n=40 | 18.2 (13.2), n=38 |
|  | Maternal 25(OH)D concentration at delivery (nmol/L) | 76.7 (28.9), n=78 | | 85.1 (28.7), n=40 | 67.9 (26.7), n=38 |
| Multu 2014 | Birthweight (g) | 3368 (425), n=32 | | 3315 (351), n=12 | 3399 (479), n=20 |
|  | Maternal baseline 25(OH)D concentration (nmol/L) | 26.6 (8.9), n=63 | | 25.0 (7.2), n=32 | 28.2 (10.2), n=31 |
|  | Maternal 25(OH)D concentration at delivery (nmol/L) | 53.7 (21.0), n=63 | | 61.7 (13.4), n=32 | 45.4 (24.2), n=31 |
|  | Cord 25(OH)D concentration (nmol/L) | 68.6 (36.8), n=32 | | 84.9 (47.7), n=12 | 58.9 (25.0), n=20 |
| Sahoo 2016 | Birthweight (g) | 2710 (450), n=36 | | 2670 (450), n=23 | 2780 (460), n=13 |
|  | Birth body length (cm) | 47.2 (2.1), n=36 | | 47.2 (2.3), n=23 | 47.1 (1.9), n=13 |
|  | Maternal baseline 25(OH)D concentration (nmol/L) | 27.1 (17.6), n=36 | | 29.2 (18.1), n=23 | 23.3 (16.7), n=13 |
|  | Maternal 25(OH)D concentration at delivery (nmol/L) | 55.3 (21.1), n=36 | | 59.8 (22.9), n=23 | 47.3 (15.3), n=13 |
|  | Cord 25(OH)D concentration (nmol/L) | 41.7 (15.9), n=36 | | 47.8 (13.8), n=23 | 31.0 (14.0), n=13 |
|  | Weight for age z score at 1 year old | -0.9 (1.1), n=36 | | -0.9 (1.2), n=23 | -1.1 (0.9), n=13 |
|  | Length for age z score at 1 year old | -0.7 (1.0), n=36 | | -0.5 (1.0), n=23 | -0.9 (1.0), n=13 |
|  | Head circumference for age z score at 1 year old | -1 (0.9), n=36 | | -1.0 (0.7), n=23 | -1.0 (1.3), n=13 |
|  | Infant bone mineral content (g) | 209.4 (40.9), n=36 | | 213.1 (46.2), n=23 | 202.9 (29.9), n=13 |
|  | Infant bone mineral density | 0.3 (0.0), n=36 | | 0.3 (0.0), n=23 | 0.3 (0.0), n=13 |
| Enkhmaa 2019 | Birthweight (g) | 3396 (432), n=233 | | 23316 (458), n=117 | 3476 (391), n=116 |
|  | Maternal baseline 25(OH)D concentration (nmol/L) | 20.0(23.0), n=241 | | 20.0 (22.0), n=120 | 20.0 (24.0), n=121 |
|  | Maternal 25(OH)D concentration at delivery (nmol/L) | 75.6 (26.7), n=236 | | 81.0 (29.0), n=120 | 70.0 (23.0), n=116 |
| Xiaomang 2021 | Birthweight (g) | 2930 (470), n=272 | | 2959 (490), n=138 | 2910 (450), n=134 |

^a^ sd: standard deviation

^b^ IQR: inter-quartile range

**Table S5. Reference of included trials (ID 1 to 38 were adopted and modified from Roth et al. BMJ. 2017 Appendix 5)**

| ID | Trial | Citations and follow-up/sub-studies |
| --- | --- | --- |
| 1 | Brooke 1980 | Brooke OG, Brown IR, Bone CD, Carter ND, Cleeve HJ, Maxwell JD, Robinson VP, Winder SM. Vitamin D supplements in pregnant Asian women: effects on calcium status and fetal growth. Br Med J. 1980 Mar 15;280(6216):751-4.  **Follow-up/sub-studies:** 1. Brooke OG, Butters F, Wood C. Intrauterine vitamin D nutrition and postnatal growth in Asian infants. British medical journal (Clinical research ed.). 1981 Oct 17;283(6298):1024.  2. Brown IR, Brooke OG, Cleeve HJ. Changes in mineral metabolism in the human foetus and newborns associated with maternal vitamin D supplements. Biochemical Society Transactions. 1980;8(1):136-137. 3. Maxwell JD, Ang L, Brooke OG, Brown IR. Vitamin D supplements enhance weight gain and nutritional status in pregnant Asians. BJOG: An International Journal of Obstetrics & Gynaecology. 1981 Oct 1;88(10):987-91 |
| 2 | Delvin 1986 | Delvin EE, Salle BL, Glorieux FH, Adeleine P, David LS. Vitamin D supplementation during pregnancy: effect on neonatal calcium homeostasis. The Journal of pediatrics. 1986 Aug 1;109(2):328-34. |
| 3 | Mallet 1986 | Mallet E, Gügi B, Brunelle P, Henocq A, Basuyau JP, Lemeur H. Vitamin D supplementation in pregnancy: a controlled trial of two methods. Obstetrics & Gynecology. 1986 Sep 1;68(3):300-4.  **Follow-up/sub-studies:**  1. de Ménibus CH, Mallet E, Henocq A, Lemeur H. Should vitamin D supplements be given to pregnant women? Bulletin de l'Academie nationale de medecine. 1983 Dec;168(7-8):909-16. |
| 4 | Marya 1988 | Marya RK, Rathee S, Dua V, Sangwan K. Effect of vitamin D supplementation during pregnancy on foetal growth. Indian journal of medical research. 1988;88:488-92. |
| 5 | Kaur 1991 | Kaur J, Marya RK, Rathee S, Lal H, Singh GP. Effect of pharmacological doses of vitamin D during pregnancy on placental protein status and birth weight. Nutrition Research. 1991 Sep 1;11(9):1077-81. |
| 6 | Sahu 2009 | Sahu M, Das V, Aggarwal A, Rawat V, Saxena P, Bhatia V. Vitamin D replacement in pregnant women in rural north India: a pilot study. European journal of clinical nutrition. 2009 Sep 1;63(9):1157-9.  **Follow-up/sub-studies:** 1. Das V, Agarwal A, Bhatia V, Pandey A, Agarwal S, Saxena P, Sahu M. O205 Evaluation of Vit D status and need for supplementation in pregnant women of a rural area of North India. International Journal of Gynecology & Obstetrics. 2009 Oct 1;107:S151. |
| 7 | Yu 2009 | Yu CK, Sykes L, Sethi M, Teoh TG, Robinson S. Vitamin D deficiency and supplementation during pregnancy. Clinical endocrinology. 2009 May 1;70(5):685-90.  **Follow-up/sub-studies:** 1. Goldring ST, Griffiths CJ, Martineau AR, Robinson S, Yu C, Poulton S, Kirkby JC, Stocks J, Hooper R, Shaheen SO, Warner JO. Prenatal vitamin D supplementation and child respiratory health: a randomised controlled trial. PloS one. 2013 Jun 24;8(6):e66627. 2. Griffiths M, Goldring S, Griffiths C, Shaheen SO, Martineau A, Cross L, Robinson S, Warner JO, Devine A, Boyle RJ. Effects of Pre-Natal Vitamin D Supplementation with Partial Correction of Vitamin D Deficiency on Early Life Healthcare Utilisation: A Randomised Controlled Trial. PloS one. 2015 Dec 23;10(12):e0145303. |
| 8 | Hollis 2011 | Hollis BW, Johnson D, Hulsey TC, Ebeling M, Wagner CL. Vitamin D supplementation during pregnancy: Double‐blind, randomized clinical trial of safety and effectiveness. Journal of bone and mineral research. 2011 Oct 1;26(10):2341-57.  **Follow-up/sub-studies:** 1. Appelgren KE, Nietert PJ, Hulsey TC, Hollis BW, Wagner CL. Analyzing adherence to prenatal supplement: does pill count measure up? International journal of endocrinology. 2010 Feb 4;2010.  2. Hollis BW, Wagner CL. Vitamin D and pregnancy: skeletal effects, nonskeletal effects, and birth outcomes. Calcified tissue international. 2013 Feb 1;92(2):128-39.  3. Rodgers MD, Mead MJ, McWhorter CA, Ebeling MD, Shary JR, Newton DA, Baatz JE, Gregoski MJ, Hollis BW, Wagner CL. Vitamin D and Child Neurodevelopment-A Post Hoc Analysis. Nutrients. 2023 Oct 3;15(19):4250. |
| 9 | Sabet 2012 | Sabet Z, Ghazi AA, Tohidi M, Oladi B. Vitamin D supplementation in pregnant Iranian women: effects on maternal and neonatal vitamin D and parathyroid hormone status. Acta Endocrinologica (1841-0987). 2012 Jan 1;8(1). |
| 10 | Dawodu 2013 | Dawodu A, Saadi HF, Bekdache G, Javed Y, Altaye M, Hollis BW. Randomized controlled trial (RCT) of vitamin D supplementation in pregnancy in a population with endemic vitamin D deficiency. The Journal of Clinical Endocrinology & Metabolism. 2013 Apr 4;98(6):2337-46. |
| 11 | Hashemipour 2013 | Hashemipour S, Lalooha F, Mirdamadi SZ, Ziaee A, Ghaleh TD. Effect of vitamin D administration in vitamin D-deficient pregnant women on maternal and neonatal serum calcium and vitamin D concentrations: a randomised clinical trial. British Journal of Nutrition. 2013 Nov 14;110(09):1611-6.  **Follow-up/sub-studies:** 1. Hashemipour S, Ziaee A, Javadi A, Movahed F, Elmizadeh K, Javadi EH, Lalooha F. Effect of treatment of vitamin D deficiency and insufficiency during pregnancy on fetal growth indices and maternal weight gain: a randomized clinical trial. European Journal of Obstetrics & Gynecology and Reproductive Biology. 2014 Jan 31;172:15-9. |
| 12 | Roth 2013 | Roth DE, Al Mahmud A, Raqib R, Akhtar E, Perumal N, Pezzack B, Baqui AH. Randomized placebo-controlled trial of high-dose prenatal third-trimester vitamin D3 supplementation in Bangladesh: the AViDD trial. Nutrition journal. 2013 Apr 12;12(1):47.  **Follow-up/sub-studies**:  1. Roth DE, Perumal N, Al Mahmud A, Baqui AH. Maternal vitamin D3 supplementation during the third trimester of pregnancy: effects on infant growth in a longitudinal Follow-up study in Bangladesh. The Journal of pediatrics. 2013 Dec 31;163(6):1605-11.  2. Harrington J, Perumal N, Al Mahmud A, Baqui A, Roth DE. Vitamin D and fetal-neonatal calcium homeostasis: findings from a randomized controlled trial of high-dose antenatal vitamin D supplementation. Pediatric research. 2014 Jun 17;76(3):302-9.  3. Perumal N, Al Mahmud A, Baqui AH, Roth DE. Prenatal vitamin D supplementation and infant vitamin D status in Bangladesh. The FASEB Journal. 2014;28(no.1 supplement 256.4)  4. Perumal N, Al Mahmud A, Baqui AH, Roth DE. Prenatal vitamin D supplementation and infant vitamin D status in Bangladesh. Public health nutrition. 2015 Nov 6:1-9.  5. Akhtar E, Mily A, Haq A, Al-Mahmud A, El-Arifeen S, Baqui AH, Roth DE, Raqib R. Prenatal high-dose vitamin D3 supplementation has balanced effects on cord blood Th1 and Th2 responses. Nutrition Journal. 2016 Aug 9;15(1):75.  6. Dimitris MC, Perumal N, Craig-Barnes HA, Leadley M, Mahmud AA, Baqui AH, Roth DE. Effect of weekly high-dose vitamin D3 supplementation on serum cholecalciferol concentrations in pregnant women. The Journal of steroid biochemistry and molecular biology. 2016 Apr 30;158:76-81. |
| 13 | Soheilykhah 2013 | Soheilykhah S, Mojibian M, Moghadam MJ, Shojaoddiny-Ardekani A. The effect of different doses of vitamin D supplementation on insulin resistance during pregnancy. Gynecological Endocrinology. 2013 Apr 1;29(4):396-9. |
| 14 | Grant 2014 | Grant CC, Stewart AW, Scragg R, Milne T, Rowden J, Ekeroma A, Wall C, Mitchell EA, Crengle S, Trenholme A, Crane J. Vitamin D during pregnancy and infancy and infant serum 25-hydroxyvitamin D concentration. Pediatrics. 2013 Dec 1:peds-2013.  **Follow-up/sub-studies:** 1. Grant CC, Kaur S, Waymouth E, Mitchell EA, Scragg R, Ekeroma A, Stewart A, Crane J, Trenholme A, Camargo CA. Reduced Primary care respiratory infection visits following pregnancy and infancy vitamin D supplementation: a randomised controlled trial. Acta paediatrica. 2015 Apr 1;104(4):396-404. 2. Grant CC, Crane J, Mitchell EA, Sinclair J, Stewart A, Milne T, Knight J, Gilchrist C, Camargo CA. Vitamin D supplementation during pregnancy and infancy reduces aeroallergen sensitization: a randomized controlled trial. Allergy. 2016 Sep 1;71(9):1325-34. 3. Wall CR, Stewart AW, Camargo CA, Scragg R, Mitchell EA, Ekeroma A, Crane J, Milne T, Rowden J, Horst R, Grant CC. Vitamin D activity of breast milk in women randomly assigned to vitamin D3 supplementation during pregnancy. The American journal of clinical nutrition. 2016 Feb 1;103(2):382-8. |
| 15 | Hossain 2014 | Hossain N, Kanani FH, Ramzan S, Kausar R, Ayaz S, Khanani R, Pal L. Obstetric and neonatal outcomes of maternal vitamin D supplementation: results of an open-label, randomized controlled trial of antenatal vitamin D supplementation in Pakistani women. The Journal of Clinical Endocrinology & Metabolism. 2014 Mar 19;99(7):2448-55.  **Follow-up/sub-studies:** 1. Hossain N, Kanani F, Khanani R, Ayaz S, Pal L. Effect of maternal supplementation with vitamin D during pregnancy on neonatal serum vitamin D concentrations and anthropometric measurements. International Journal of Gynecology & Obstetrics. 2012 Oct 1;119:S372. |
| 16 | Mutlu 2014 | Yesiltepe Mutlu G, Ozsu E, Kalaca S, Yuksel A, Pehlevan Y, Cizmecioglu F, Hatun S. Evaluation of vitamin D supplementation doses during pregnancy in a population at high risk for deficiency. Hormone Research in Paediatrics. 2014 Apr 3;81(6):402-8. |
| 17 | Yap 2014 | Yap C, Cheung NW, Gunton JE, Athayde N, Munns CF, Duke A, McLean M. Vitamin D supplementation and the effects on glucose metabolism during pregnancy: a randomized controlled trial. Diabetes Care. 2014 Jul 1;37(7):1837-44. |
| 18 | March 2015 | March KM, Chen NN, Karakochuk CD, Shand AW, Innis SM, von Dadelszen P, Barr SI, Lyon MR, Whiting SJ, Weiler HA, Green TJ. Maternal vitamin D3 supplementation at 50 μg/d protects against low serum 25-hydroxyvitamin D in infants at 8 wk of age: a randomized controlled trial of 3 doses of vitamin D beginning in gestation and continued in lactation. The American journal of clinical nutrition. 2015 Aug 1;102(2):402-10.  **Follow-up/sub-studies:** 1. Chen NN, March K, Innis SM, Shand A, von Dadelszen P, Lyon M, Weiler H, Green TJ. The effect of vitamin D supplementation during pregnancy and lactation on maternal and infant 25-hydroxyvitamin D (25OHD) concentration. The FASEB journal. 2013;27(no 1. Supplement lb259) |
| 19 | Charandabi 2015 | Mohammad-Alizadeh-Charandabi S, Mirghafourvand M, Mansouri A, Najafi M, Khodabande F. The effect of vitamin D and calcium plus vitamin D during pregnancy on pregnancy and birth outcomes: a randomized controlled trial. Journal of caring sciences. 2015 Mar;4(1):35.  **Follow-up/sub-studies:** 1. Mirghafourvand M, Mohammad AC, Mansouri A, Najafi M, Khodabande F. The effect of vitamin D and calcium plus vitamin D on sleep quality in pregnant women with leg cramps: a controlled randomized clinical trial. Journal of Isfahan Medical School. 2015; 320.  2. Mansouri A, Mirghafourvand M, Charandabi SM, Najafi M. The effect of Vitamin D and calcium plus Vitamin D on leg cramps in pregnant women: A randomized controlled trial. Journal of research in medical sciences: the official journal of Isfahan University of Medical Sciences. 2017;22. |
| 20 | Mojibian 2015 | Mojibian M, Soheilykhah S, Zadeh MA, Moghadam MJ. The effects of vitamin D supplementation on maternal and neonatal outcome: A randomized clinical trial. Iranian journal of reproductive medicine. 2015 Nov;13(11):687. |
| 21 | Rodda 2015 | Rodda CP, Benson JE, Vincent AJ, Whitehead CL, Polykov A, Vollenhoven B. Maternal vitamin D supplementation during pregnancy prevents vitamin D deficiency in the newborn: an open‐label randomized controlled trial. Clinical endocrinology. 2015 Sep 1;83(3):363-8. |
| 22 | Sablok 2015 | Sablok A, Batra A, Thariani K, Batra A, Bharti R, Aggarwal AR, Kabi BC, Chellani H. Supplementation of vitamin D in pregnancy and its correlation with feto‐maternal outcome. Clinical endocrinology. 2015 Oct 1;83(4):536-41. |
| 23 | Chawes 2016 | Chawes BL, Bønnelykke K, Stokholm J, Vissing NH, Bjarnadóttir E, Schoos AM, Wolsk HM, Pedersen TM, Vinding RK, Thorsteinsdóttir S, Arianto L. Effect of vitamin D3 supplementation during pregnancy on risk of persistent wheeze in the offspring: a randomized clinical trial. Jama. 2016 Jan 26;315(4):353-61.  **Follow-up/sub-studies:** 1. Brustad N, Garland J, Thorsen J, Sevelsted A, Krakauer M, Vinding RK, Stokholm J, Bønnelykke K, Bisgaard H, Chawes BL. Effect of High-Dose vs Standard-Dose Vitamin D Supplementation in Pregnancy on Bone Mineralization in Offspring Until Age 6 Years: A Prespecified Secondary Analysis of a Double-Blinded, Randomized Clinical Trial. JAMA Pediatr. 2020 May 1;174(5):419-427.  2. Sass L, Vinding RK, Stokholm J, Bjarnadóttir E, Noergaard S, Thorsen J, Sunde RB, McGrath J, Bønnelykke K, Chawes B, Bisgaard H. High-Dose Vitamin D Supplementation in Pregnancy and Neurodevelopment in Childhood: A Prespecified Secondary Analysis of a Randomized Clinical Trial. JAMA Netw Open. 2020 Dec 1;3(12):e2026018.  3. Brustad N, Chawes BL, Thorsen J, Krakauer M, Lasky-Su J, Weiss ST, Stokholm J, Bønnelykke K, Bisgaard H. High-dose vitamin D supplementation in pregnancy and vitamin D sufficiency in childhood reduce the risk of fractures and improve bone mineralization in childhood: Follow-up of a randomized clinical trial. EClinicalMedicine. 2021 Dec 24;43:101254.  4. Brustad N, Yang L, Chawes BL, Stokholm J, Gürdeniz G, Bønnelykke K, Bisgaard H. Fish Oil and Vitamin D Supplementations in Pregnancy Protect Against Childhood Croup. J Allergy Clin Immunol Pract. 2023 Jan;11(1):315-321. |
| 24 | Cooper 2016 | Cooper C, Harvey NC, Bishop NJ, Kennedy S, Papageorghiou AT, Schoenmakers I, Fraser R, Gandhi SV, Carr A, D'Angelo S, Crozier SR. Maternal gestational vitamin D supplementation and offspring bone health (MAVIDOS): a multicentre, double-blind, randomised placebo-controlled trial. The lancet Diabetes & endocrinology. 2016 May 31;4(5):393-402.  **Follow-up/sub-studies:**  1. Cooper C, Harvey NC, Javaid MK, Bishop NJ, Kennedy S, Papageorghiou AT, Fraser R, Gandhi SV, D'angelo S, Crozier SR, Moon RJ. Effectiveness of maternal vitamin D supplementation: a multicenter randomised, double-blind, placebo controlled trial (MAVIDOS). Osteoporosis International. 2015; 26(no. 1 supplement S40).  2. Moon RJ, Harvey NC, Cooper C, D'Angelo S, Crozier SR, Inskip HM, Schoenmakers I, Prentice A, Arden NK, Bishop NJ, Carr A. Determinants of the maternal 25-hydroxyvitamin D response to vitamin D supplementation during pregnancy. The Journal of Clinical Endocrinology & Metabolism. 2016 Oct 28;101(12):5012-20.  3. Curtis E, Cook E, Krstic N, D’Angelo S, Crozier S, Moon R, Murray R, Garratt E, Costello P, Bishop NJ, Kennedy S, Papageorghiou A, Schoenmakers I, Fraser R, Gandhi S, Prentice A, Javaid K, Inskip H, Godfrey K, Bell C, Cooper C, Lillycrop K, Harvey N. DNA methylation at the RXRA promoter at birth is associated with gestational vitamin D supplementation. Results from the MAVIDOS trial. Osteoporosis International. 2017; 28(no. 1 Supplement): S72-S73.  4. El-Heis S, D'Angelo S, Curtis EM, Healy E, Moon RJ, Crozier SR, Inskip H, Cooper C, Harvey NC, Godfrey KM; MAVIDOS Trial Group. Maternal antenatal vitamin D supplementation and offspring risk of atopic eczema in the first 4 years of life: evidence from a randomized controlled trial. Br J Dermatol. 2022 Nov;187(5):659-666.  5. Curtis, E., Moon, R., D'Angelo, S., Crozier, S., Bishop, N., Gopal-Kothandapani, J., Harvey, N. (2021). Pregnancy vitamin D supplementation leads to greater offspring bone mineral density at age 4 years: Findings from the MAVIDOS trial. Proceedings of the Nutrition Society, 80(OCE5), E182.  6. Moon RJ, D'Angelo S, Crozier SR, Curtis EM, Fernandes M, Kermack AJ, Davies JH, Godfrey KM, Bishop NJ, Kennedy SH, Prentice A, Schoenmakers I, Fraser R, Gandhi SV, Inskip HM, Javaid MK, Papageorghiou AT, Cooper C, Harvey NC. Does antenatal cholecalciferol supplementation affect the mode or timing of delivery? Post hoc analyses of the MAVIDOS randomized controlled trial. J Public Health (Oxf). 2023 Aug 28;45(3):738-747. |
| 25 | Khan 2016 | Khan FR, Ahmad T, Hussain R, Bhutta ZA. A Randomized Controlled Trial of Oral Vitamin D Supplementation in Pregnancy to Improve Maternal Periodontal Health and Birth Weight. Journal of International Oral Health. 2016 Jun 1;8(6):657. |
| 26 | Litonjua 2016 | Litonjua AA, Carey VJ, Laranjo N, Harshfield BJ, McElrath TF, O’Connor GT, Sandel M, Iverson RE, Lee-Paritz A, Strunk RC, Bacharier LB. Effect of prenatal supplementation with vitamin D on asthma or recurrent wheezing in offspring by age 3 years: the VDAART randomized clinical trial. Jama. 2016 Jan 26;315(4):362-70.  **Follow-up/sub-studies:** 1. Al-Garawi A, Carey VJ, Chhabra D, Mirzakhani H, Morrow J, Lasky-Su J, Qiu W, Laranjo N, Litonjua AA, Weiss ST. The Role of Vitamin D in the Transcriptional Program of Human Pregnancy. PloS one. 2016 Oct 6;11(10):e0163832.Mirzakhani H, Litonjua AA, McElrath TF, O’Connor G, Lee-Parritz A, Iverson R, Macones G, Strunk RC, Bacharier LB, Zeiger R, Hollis BW. Early pregnancy vitamin D status and risk of preeclampsia. The Journal of Clinical Investigation. 2016 Nov 14;126(12).  2. Mirzakhani H, Litonjua AA, McElrath TF, O’Connor G, Lee-Parritz A, Iverson R, Macones G, Strunk RC, Bacharier LB, Zeiger R, Hollis BW. Early pregnancy vitamin D status and risk of preeclampsia. The Journal of Clinical Investigation. 2016 Nov 14;126(12).  3. Hornsby E, Pfeffer PE, Laranjo N, Cruikshank W, Tuzova M, Litonjua AA, Weiss ST, Carey VJ, O'Connor G, Hawrylowicz C. Vitamin D supplementation during pregnancy: Effect on the neonatal immune system in a randomized controlled trial. Journal of Allergy and Clinical Immunology. 2017 May 26. 4. Wolsk HM, Harshfield BJ, Laranjo N, Carey VJ, O'Connor G, Sandel M, Strunk RC, Bacharier LB, Zeiger RS, Schatz M, Hollis BW. Vitamin D supplementation in pregnancy, prenatal 25 (OH) D concentrations, race, and subsequent asthma or recurrent wheeze in offspring: Secondary analyses from the Vitamin D Antenatal Asthma Reduction Trial. Journal of Allergy and Clinical Immunology. 2017 Mar 9. 5. Knihtilä HM, Huang M, Prince N, Stubbs BJ, Carey VJ, Laranjo N, Mirzakhani H, Zeiger RS, Bacharier LB, O'Connor GT, Litonjua AA, Weiss ST, Lasky-Su J. Maternal vitamin D status modifies the effects of early life tobacco exposure on child lung function. J Allergy Clin Immunol. 2023 Feb;151(2):556-564. 6. Kelly RS, Lee-Sarwar K, Chen YC, Laranjo N, Fichorova R, Chu SH, Prince N, Lasky-Su J, Weiss ST, Litonjua AA. Maternal Inflammatory Biomarkers during Pregnancy and Early Life Neurodevelopment in Offspring: Results from the VDAART Study. Int J Mol Sci. 2022 Dec 3;23(23):15249.  7. Shadid IL, Brustad N, Lu M, Chawes BL, Bisgaard H, Zeiger RS, O'Connor GT, Bacharier LB, Guchelaar HJ, Litonjua AA, Weiss ST, Mirzakhani H. The Impact of Baseline 25-Hydroxyvitamin D Level and Gestational Age on Prenatal Vitamin D Supplementation to Prevent Offspring Asthma or Recurrent Wheezing. Am J Clin Nutr. 2023 Jun;117(6):1342-1352.  8. Weiss ST, Mirzakhani H, Carey VJ, O'Connor GT, Zeiger RS, Bacharier LB, Stokes J, Litonjua AA. Prenatal vitamin D supplementation to prevent childhood asthma: 15-year results from the Vitamin D Antenatal Asthma Reduction Trial (VDAART). J Allergy Clin Immunol. 2023 Oct 16:S0091-6749(23)01254-X. |
| 27 | Naghshineh 2016 | Naghshineh E, Sheikhaliyan S. Effect of vitamin D supplementation in the reduce risk of preeclampsia in nulliparous women. Advanced biomedical research. 2016;5. |
| 28 | Sahoo 2016 | Sahoo SK, Katam KK, Das V, Agarwal A, Bhatia V. Maternal vitamin D supplementation in pregnancy and offspring outcomes: a double-blind randomized placebo-controlled trial. Journal of Bone and Mineral Metabolism. 2016 Sep 14:1-8. |
| 29 | Shahgheibi 2016 | Shahgheibi S, Farhadifar F, Pouya B. The effect of vitamin D supplementation on gestational diabetes in high-risk women: Results from a randomized placebo-controlled trial. Journal of Research in Medical Sciences: The Official Journal of Isfahan University of Medical Sciences. 2016;21. |
| 30 | Thiele 2016 | Thiele DK, Ralph J, El-Masri M, Anderson CM. Vitamin D3 Supplementation During Pregnancy and Lactation Improves Vitamin D Status of the Mother–Infant Dyad. Journal of Obstetric, Gynecologic & Neonatal Nursing. 2017 Feb 28;46(1):135-47.  **Follow-up/sub-studies:** 1. Anderson CM, Thiele DK, Ralph JL, Perley D, Ohm JE. Vitamin D Supplementation and DNA Methylation Patterns during Pregnancy and Lactation in Mothers and Infants. The FASEB Journal. 2016 Apr 1;30(1 Supplement):1028-3. |
| 31 | Valizadeh 2016 | Valizadeh M, Piri Z, Mohammadian F, Kamali K, Moghadami HR. The Impact of Vitamin D Supplementation on Post-Partum Glucose Tolerance and Insulin Resistance in Gestational Diabetes: A Randomized Controlled Trial. International Journal of Endocrinology and Metabolism. 2016 Apr;14(2). |
| 32 | Vaziri 2016 | Vaziri F, Dabbaghmanesh MH, Samsami A, Nasiri S, Shirazi PT. Vitamin D supplementation during pregnancy on infant anthropometric measurements and bone mass of mother-infant pairs: A randomized placebo clinical trial. Early Human Development. 2016 Dec 31; 103:61-8.  **Follow-up/sub-studies:** 1. Vaziri F, Nasiri S, Tavana Z, Dabbaghmanesh MH, Sharif F, Jafari P. A randomized controlled trial of vitamin D supplementation on perinatal depression: in Iranian pregnant mothers. BMC Pregnancy and Childbirth. 2016 Aug 20;16(1):239. |
| 33 | Yazdchi 2016 | Yazdchi R, Gargari BP, Asghari-Jafarabadi M, Sahhaf F. Effects of vitamin D supplementation on metabolic indices and hs-CRP concentrations in gestational diabetes mellitus patients: a randomized, double-blinded, placebo-controlled clinical trial. Nutrition research and practice. 2016 Jun 1;10(3):328-35. |
| 34 | Zerofsky 2016 | Zerofsky MS, Jacoby BN, Pedersen TL, Stephensen CB. Daily cholecalciferol supplementation during pregnancy alters markers of regulatory immunity, inflammation, and clinical outcomes in a randomized controlled trial. The Journal of nutrition. 2016 Nov 1;146(11):2388-97.  **Follow-up/sub-studies:** 1. Zerofsky M, Jacoby B, Stephensen CB. A randomized controlled trial of vitamin D supplementation in pregnancy: effects on vitamin D status and clinical outcomes. The FASEB journal; 28 (no.1 supplement 1041.5) 2. Zerofsky M, Jacoby B, Pedersen TL, Stephensen CB. Effects of a randomized, controlled trial of daily vitamin D3 supplementation during pregnancy on regulatory immunity and inflammation. The FASEB journal; 30 (no. 1 supplement 296.7) |
| 35 | Abotorabi 2017 | Abotorabi S, Hashemi Poor S, Esmailzadehha N, Ziaee A, Khoeiniha MH. Effect of Treatment with Vitamin D on Maternal and Neonatal Indices in Pregnant Women with Hypocalcemia: A Randomized Controlled Trial. International Journal of Pediatrics. 2017;5(9):5733-9. |
| 36 | Samimi 2017 | Samimi M, Foroozanfard F, Amini F, Sehat M. Effect of vitamin D supplementation on unexplained recurrent spontaneous abortion: a double-blind randomized controlled trial. Global Journal of Health Science. 2017 Mar; 9(3):95. |
| 37 | Tehrani 2017 | Tehrani HG, Mostajeran F, Banihashemi B. Effect of Vitamin D Supplementation on the Incidence of Gestational Diabetes. Advanced Biomedical Research. 2017;6:79 |
| 38 | Sasan 2017 | Behjat Sasan S, Zandvakili F, Soufizadeh N, Baybordi E. The Effects of Vitamin D Supplement on Prevention of Recurrence of Preeclampsia in Pregnant Women with a History of Preeclampsia. Obstetrics and Gynecology International. 2017;2017. Article ID 8249264. |
| 39 | Azadehrah 2017 | Azadehrah M, Mobasheri E, Behnampour N, Azadehrah . Effect of Maternal Supplementation with 200 and 50,000 IU Vitamin D on Serum vitamin D concentrations of Pregnant Women. Pharmacophore, 8(6S) 2017, e-1173685, Pages. |
| 40 | Keshavarzi 2017 | Keshavarzi F, Nankali A, Azizi F, Hematti M. Low-dose versus high-dose vitamin D supplementation and pregnancy outcome in gestational diabetes. World Family Medicine. 2017 Dec; 5(10):50-5. |
| 41 | Best 2018 | Best CM, Pressman EK, Queenan RA, Cooper E, Vermeylen F, O'Brien KO. Gestational Age and Maternal Serum 25-hydroxyvitamin D Concentration Interact to Affect the 24,25-dihydroxyvitamin D Concentration in Pregnant Adolescents. J Nutr. 2018 Jun 1;148(6):868-875. |
| 42 | O’Callaghan 2018 | O'Callaghan KM, Hennessy Á, Hull GLJ, Healy K, Ritz C, Kenny LC, Cashman KD, Kiely ME. Estimation of the maternal vitamin D intake that maintains circulating 25-hydroxyvitamin D in late gestation at a concentration sufficient to keep umbilical cord sera ≥25-30 nmol/L: a dose-response, double-blind, randomized placebo-controlled trial in pregnant women at northern latitude. Am J Clin Nutr. 2018 Jul 1;108(1):77-91. |
| 43 | Roth 2018 | Roth DE, Morris SK, Zlotkin S, Gernand AD, Ahmed T, Shanta SS, Papp E, Korsiak J, Shi J, Islam MM, Jahan I, Keya FK, Willan AR, Weksberg R, Mohsin M, Rahman QS, Shah PS, Murphy KE, Stimec J, Pell LG, Qamar H, Al Mahmud A. Vitamin D Supplementation in Pregnancy and Lactation and Infant Growth. N Engl J Med. 2018 Aug 9;379(6):535-546. doi: 10.1056/NEJMoa1800927. Erratum in: N Engl J Med. 2021 Oct 28;385(18):1728.   **Follow-up/sub-studies:** 1. Morris SK, Pell LG, Rahman MZ, Mahmud AA, Shi J, Ahmed T, Dimitris MC, Gubbay JB, Islam MM, Kashem T, Keya FK, Mohsin M, Pullenayegum E, Science M, Shanta SS, Sumiya MK, Zlotkin S, Roth DE. Effects of Maternal Vitamin D Supplementation During Pregnancy and Lactation on Infant Acute Respiratory Infections: Follow-up of a Randomized Trial in Bangladesh. J Pediatric Infect Dis Soc. 2021 Oct 27;10(9):901-909.  2. O'Callaghan KM, Shanta SS, Fariha F, Harrington J, Mahmud AA, Emdin AL, Gernand AD, Ahmed T, Abrams SA, Moore DR, Roth DE. Effect of maternal prenatal and postpartum vitamin D supplementation on offspring bone mass and muscle strength in early childhood: follow-up of a randomized controlled trial. Am J Clin Nutr. 2022 Mar 4;115(3):770-780. Erratum in: Am J Clin Nutr. 2023 May;117(5):1047. |
| 44 | Ali 2018 | Ali AM, Alobaid A, Malhis TN, Khattab AF. Effect of vitamin D3 supplementation in pregnancy on risk of pre-eclampsia - Randomized controlled trial. Clin Nutr. 2019 Apr;38(2):557-563. |
| 45 | Enkhmaa 2019 | Enkhmaa D, Tanz L, Ganmaa D, Enkhtur S, Oyun-Erdene B, Stuart J, Chen G, Carr A, Seely EW, Fitzmaurice G, Buyandelger Y, Sarantsetseg B, Gantsetseg G, Rich-Edwards J. Randomized trial of three doses of vitamin D to reduce deficiency in pregnant Mongolian women. EBioMedicine. 2019 Jan;39:510-519.  **Follow-up/sub-studies:**  1. Nasantogtokh E, Ganmaa D, Altantuya S, Amgalan B, Enkhmaa D. Maternal vitamin D intakes during pregnancy and child health outcome. J Steroid Biochem Mol Biol. 2023 Oct 21;235:106411. |
| 46 | Jefferson 2019 | Jefferson KK, Parikh HI, Garcia EM, Edwards DJ, Serrano MG, Hewison M, Shary JR, Powell AM, Hollis BW, Fettweis JM, Strauss Iii JF, Buck GA, Wagner CL. Relationship between vitamin D status and the vaginal microbiome during pregnancy. J Perinatol. 2019 Jun;39(6):824-836.  **Follow-up/sub-studies:** 1. Powell AM, Shary JR, Louden C, Ramakrishnan V, Eckard AR, Wagner CL. Association of Bacterial Vaginosis with Vitamin D in Pregnancy: Secondary Analysis from the Kellogg Pregnancy Study. AJP Rep. 2019 Jul;9(3):e226-e234. 2. Khatiwada A, Wolf BJ, Mulligan JK, Shary JR, Hewison M, Baatz JE, Newton DA, Hawrylowicz C, Hollis BW, Wagner CL. Effects of vitamin D supplementation on circulating concentrations of growth factors and immune-mediators in healthy women during pregnancy. Pediatr Res. 2021 Feb;89(3):554-562.  3. Mead MJ, McWhorter CA, Rodgers MD, Ebeling MD, Shary JR, Gregoski MJ, Hollis BW, Hewison M, Johnson D, Caplan MJ, Wagner CL. Does maternal vitamin D status influence placental weight or vascular and inflammatory pathology? Secondary analysis from the Kellogg Pregnancy Study. J Steroid Biochem Mol Biol. 2023 Oct;233:106358.  4. McWhorter CA, Mead MJ, Rodgers MD, Ebeling MD, Shary JR, Gregoski MJ, Newton DA, Baatz JE, Hollis BW, Hewison M, Wagner CL. Predicting comorbidities of pregnancy: A comparison between total and free vitamin D and their associations with parathyroid hormone. J Steroid Biochem Mol Biol. 2023 Oct 31;235:106420. |
| 47 | Zhao 2019 | Zhao Y, Teng Y, Wang J, Yang Z, Dong S, Hu J, Zhang Y, Lai J. Effects of Vitamin D Supplementation in Early Pregnancy on High-Risk Groups of Gestational Diabetes Mellitus. Journal of hygiene research. 2019;48(2):226-231. |
| 48 | Corcoy 2020 | Corcoy R, Mendoza LC, Simmons D, Desoye G, Adelantado JM, Chico A, Devlieger R, van Assche A, Galjaard S, Timmerman D, Lapolla A, Dalfra MG, Bertolotto A, Harreiter J, Wender-Ozegowska E, Zawiejska A, Kautzky-Willer A, Dunne FP, Damm P, Mathiesen ER, Jensen DM, Andersen LLT, Tanvig M, Hill DJ, Jelsma JG, Snoek FJ, Köfeler H, Trötzmüller M, Lips P, van Poppel MNM. The DALI vitamin D randomized controlled trial for gestational diabetes mellitus prevention: No major benefit shown besides vitamin D sufficiency. Clin Nutr. 2020 Mar;39(3):976-984.  **Follow-up/sub-studies:** 1. Harreiter J, Mendoza LC, Simmons D, Desoye G, Devlieger R, Galjaard S, Damm P, Mathiesen ER, Jensen DM, Andersen LLT, Dunne F, Lapolla A, Dalfra MG, Bertolotto A, Wender-Ozegowska E, Zawiejska A, Hill D, Jelsma JGM, Snoek FJ, Worda C, Bancher-Todesca D, van Poppel MNM, Corcoy R, Kautzky-Willer A, On Behalf Of The Dali Core Investigator Group. Vitamin D3 Supplementation in Overweight/Obese Pregnant Women: No Effects on the Maternal or Fetal Lipid Profile and Body Fat Distribution-A Secondary Analysis of the Multicentric, Randomized, Controlled Vitamin D and Lifestyle for Gestational Diabetes Prevention Trial (DALI). Nutrients. 2022 Sep 14;14(18):3781.  2. Dieberger AM, Obermayer-Pietsch B, Harreiter J, Desoye G, van Poppel MNM; DALI Core Investigator group. Physical activity and sedentary time across pregnancy and associations with neonatal weight, adiposity and cord blood parameters: a secondary analysis of the DALI study. Int J Obes (Lond). 2023 Sep;47(9):873-881. |
| 49 | Rahbar 2020 | Rahbar N, Rajabi M, Ghods S, Mirmohammadkhani M. Effect of High-Dose Vitamin D Supplementation on Compensation for Vitamin D Deficiency in Pregnancy and Incidence of Gestational Diabetes Mellitus: A Randomized Clinical Trial. Middle East J Rehabil Health Stud.7(2):e99723. |
| 50 | Sircar 2020 | Ajmani SN, Sircar S. Role of Vitamin D Supplementation in Preventing Development of Gestational Diabetes Mellitus. Indian Obstetrics and Gynaecology. 2020; 10(4). |
| 51 | Alhomaid 2021 | Alhomaid RM, Mulhern MS, Strain J, Laird E, Healy M, Parker MJ, McCann MT. Maternal obesity and baseline vitamin D insufficiency alter the response to vitamin D supplementation: a double-blind, randomized trial in pregnant women. Am J Clin Nutr. 2021 Sep 1;114(3):1208-1218. |
| 52 | Manasova 2021 | Manasova GS, Kuzmin NV, Didenkul NV, Derishov SV, Badiuk NS. Supplementation of Vitamin D in Pregnant Women with vitamin D Deficiency and Risk of Preeclampsia Development Improves Perinatal Outcomes. PharmacologyOnline. 2021;3:1107-1116. |
| 53 | Mohammadi 2021 | Mohammadi F, Bahadori F, Khalkhali HR, Ghavamzadeh S. Vitamin D Effects on GH, IGF-1, Glycemic Control Indicators, and Lipid Profile in Gestational Diabetes Mellitus. Arch Pharm Pract. 2021;12(1):118-25. |
| 54 | Nausheen 2021 | Nausheen S, Habib A, Bhura M, Rizvi A, Shaheen F, Begum K, Iqbal J, Ariff S, Shaikh L, Raza SS, Soofi SB. Impact evaluation of the efficacy of different doses of vitamin D supplementation during pregnancy on pregnancy and birth outcomes: a randomised, controlled, dose comparison trial in Pakistan. BMJ Nutr Prev Health. 2021 Sep 27;4(2):425-434. |
| 55 | Srilekha 2021 | Srilekha V, Vijayalakshmi B, Reddy IY, Fathima N. Effect of Vitamin D Supplementation on Fetal Growth and Development in Pregnant Women. Biomedicine. 2021;41(4):821-824. |
| 56 | Xiaomang 2021 | Jiang X, Wei Y. Effect of Vitamin D3 Supplementation during Pregnancy on High Rsk Factors-a Randomized Controlled Trial. Journal of perinatal medicine. 2021;49(4):480-484 |
| 57 | Aggarwal 2022 | Aggarwal N, Singla R, Dutta U, Bhadada SK, Dutta S, Dhaliwal L, Garg S. Prevalence of Vitamin D Deficiency among Pregnant Women and Effect of Vitamin D Supplementation on Maternal and Fetal outcomes: A Double-Blind Randomized Placebo Controlled Trial. Asian Journal of Medical Sciences. 2022:13(2):95-101. |
| 58 | Memon 2022 | Memon HA, Rafique M, Khalid S, Perveen R, Imtiaz M, Naqvi N. Role of Vitamin D for the Prevention of Pre-Eclampsia in Pregnant Women: A Randomized Controlled Trial. Pakistan Journal of Medical & Health Sciences. 2022;16(1):1086-1088 |
| 59 | Pulido 2022 | Camarena Pulido EE, Mora González S, Corona Gutiérrez AA, Robledo Aceves M, Basso Barba P, Salgado Leyva Y. Effect of supplementation with 5,000 IU of vitamin D on the glycemic profile of women with gestational diabetes mellitus. J Perinat Med. 2022 Jul 4;50(9):1225-1229. |
| 60 | Sudfeld 2022 | Sudfeld CR, Manji KP, Muhihi A, Duggan CP, Aboud S, Alwy Al-Beity FM, Wang M, Zhang N, Ulenga N, Fawzi WW. Vitamin D3 supplementation during pregnancy and lactation for women living with HIV in Tanzania: A randomized controlled trial. PLoS Med. 2022 Apr 15;19(4): e1003973.  **Follow-up/sub-studies:**  1. Regan M, Muhihi A, Saleh A, Duggan CP, Ulenga N, Alwy Al-Beity FM, Aboud S, Fawzi WW, Manji KP, Sudfeld CR. Antenatal depression and adverse birth outcomes among pregnant women living with HIV in Dar es Salaam, Tanzania. J Affect Disord. 2023 Oct 15;339:82-88.  2. Lauer JM, Kirby MA, Muhihi A, Ulenga N, Aboud S, Liu E, Choy RKM, Arndt MB, Kou J, Fawzi W, Gewirtz A, Sudfeld CR, Manji KP, Duggan CP. Assessing environmental enteric dysfunction via multiplex assay and its relation to growth and development among HIV-exposed uninfected Tanzanian infants. PLoS Negl Trop Dis. 2023 Mar 21;17(3):e0011181. |
| 61 | Mirzaei-Azandaryani 2022 | Mirzaei-Azandaryani Z, Mohammad-Alizadeh-Charandabi S, Shaseb E, Abbasalizadeh S, Mirghafourvand M. Effects of vitamin D on insulin resistance and fasting blood glucose in pregnant women with insufficient or deficient vitamin D: a randomized, placebo-controlled trial. BMC Endocr Disord. 2022 Oct 20;22(1):254.  **Follow-up/sub-studies:**  1. Mirzaei-Azandaryani, Z., Javadzadeh, Y., Shaseb, E. and Mirghafourvand, M. "The effects of vitamin D on sleep quality and pregnancy symptoms in pregnant women: a randomized, tripled-blinded and placebo-controlled clinical trial". Nutrition & Food Science. 2023;53(8):1360-1372. |
| 62 | Vanda 2022 | Vanda R, Hassanzadeh S, Masnavi E. Comparing the Effect of Different Doses of Vitamin D Supplementation During Pregnancy on Prevention of Adverse Pregnancy Outcomes; a Randomized Clinical Trial. J Clinic Care Skill 2022; 3 (4) :191-196. |
| 63 | Cagiran 2023 | Cagiran FT, Kali Z. Role of vitamin D on gestational hypertension, diabetes mellitus, timing and mode of delivery. Eur Rev Med Pharmacol Sci. 2023 Jan;27(2):511-516. |
| 64 | Ma 2023 | Ma S, Yin W, Wang P, Wang H, Zhang L, Tao R, Hu H, Jiang X, Zhang Y, Tao F, Zhu P. Effect of vitamin D supplementation on glucose control in mid-late gestation: A randomized controlled trial. Clin Nutr. 2023 Jun;42(6):929-936. |
| 65 | Nadeem 2023 | Nadeem A, Saeed M, Sadiqa A, Moin H, Khan QU. The Effect of Vitamin D3 Intervention on the Association Among Vitamin D3, Adiponectin, and Body Mass Index in Pregnant Women With Gestational Diabetes. Cureus. 2023 Aug 15;15(8):e43506. |
| 66 | Sunarno 2023 | Sunarno RD, Kartasurya MI, Suwondo A, Rahfiludin MZ. Vitamin D Supplementation and Sun Exposure Maintain Blood Pressures of Pregnant Women and Increase Birth Weight in a Randomized Controlled Trial. Iran J Public Health. 2023 Oct;52(10):2148-2156. |

**Table S6. Description of included trials**

|  | **Author, publication year** | **Region** | **Country** | **Number of participants randomized** | **Health status at enrolment** | **Baseline 25(OH)D concentration (nmol/L)**^a^ | | **Micronutrient co-intervention** | **Vitamin D type** | **Number of arms** | **Regularity (regular or**  **bolus)** | **Supplement frequency** | **Intervention dose**^b^ | **Comparator dose** | **Initiation and duration of supplementation** |
| --- | --- | --- | --- | --- | --- | --- | --- | --- | --- | --- | --- | --- | --- | --- | --- |
| 1 | Brooke 1980 | Europe | United Kingdom | 126 | Healthy | 20.0 | - | | Vitamin D2 | 2 arms | Regular | Daily | 1,000 IU/day | 0 | GA 28 until delivery |
| 2 | Delvin 1986 | Europe | France | 40 | Healthy |  | - | | Vitamin D3 | 2 arms | Regular | Daily | 1,000 IU/day | 0 | 6 months gestation until delivery |
| 3 | Mallet 1986 | Europe | France | 77 | Healthy | - | - | | Vitamin D2 | 3 arms | Regular | Daily | 1,000 IU/day | 0 | 7th months gestation till delivery |
|  |  |  |  |  |  |  |  | |  |  | Bolus | Once | 200,000 IU |  | 7th month gestation |
| 4 | Marya 1988 | Asia | India | 200 | Healthy |  | - | | Vitamin D3 | 2 arms | Bolus | Twice | 600,000 IU | 0 | each at 7 months and 8 months gestation |
| 5 | Kaur 1991 | Asia | India | 50 | Healthy | - | - | | Vitamin D3 | 2 arms | Bolus | Twice | 120,000 IU | 0 | at 6 months and 7 months gestation |
| 6 | Sahu 2009 | Asia | India | 139 | Healthy | 25.1 | Calcium, iron | | Vitamin D3 | 3 arms | Bolus | Once | 60,000 IU | 0 | 5 months gestation |
|  |  |  |  |  |  |  |  | |  |  | Bolus | Twice | 120,000 IU |  | 5 months and 7 months gestation |
| 7 | Yu 2009 | Europe | United Kingdom | 180 | Healthy | 28.0 | - | | Vitamin D2 and D3 | 3 arms | Regular | Daily | 800 IU/day | 0 | GA 27 until delivery |
|  |  |  |  |  |  |  |  | |  |  | Bolus | Once | 200,000 IU |  | GA 27 until delivery |
| 8 | Hollis 2011 | North America | United States | 502 | Healthy | 61.6 | - | | Vitamin D3 | 3 arms | Regular | Daily | 2,000 IU/day | 400 IU/day | GA 12-16 until delivery |
|  |  |  |  |  |  |  |  | |  |  | Regular | Daily | 4,000 IU/day |  | GA 12-16 until delivery |
| 9 | Sabet 2012 | Asia | Iran | 50 | Healthy | 95.6 | - | | Vitamin D3 | 2 arms | Regular | Monthly | 100,000 IU/month (=3289 IU/day) | 0 | GA 27-28 until delivery |
| 10 | Dawodu 2013 | Asia | United Arab Emirates | 192 | Healthy | 20.5 | - | | Vitamin D3 | 3 arms | Regular | Daily | 2,000 IU/day | 400 IU/day | GA 12-14 until delivery |
|  |  |  |  |  |  |  |  | |  |  | Regular | Daily | 4,000 IU/day |  | GA 12-14 until delivery |
| 11 | Hashemipour 2013 | Asia | Iran | 130 | Healthy | 43.7 | Calcium | | Vitamin D3 | 2 arms | Regular | Weekly | 50,000 IU/week  (=7,143 IU/day) | 400 IU/day | GA 26-28 until delivery |
| 12 | Roth 2013 | Asia | Bangladesh | 160 | Healthy |  | - | | Vitamin D3 | 2 arms | Regular | Weekly | 35,000 IU/week  (=5,000 IU/day) | 0 | GA 26-29 until delivery |
| 13 | Soheilykhah 2013 | Asia | Iran | 120 | Healthy | 20.7 | - | | Unreported | 3 arms | Regular | Daily | 2,000 IU/day | 200 IU/day | GA 12 until delivery |
|  |  |  |  |  |  |  |  | |  |  | Regular | Daily | 4,000 IU/day |  | GA 12 until delivery |
| 14 | Grant 2014 | Australia New Zealand | New Zealand | 260 | Healthy | 55.7 | - | | Vitamin D3 | 3 arms | Regular | Daily | 1,000 IU/day | 0 | GA 27 until delivery |
|  |  |  |  |  |  |  |  | |  |  | Regular | Daily | 2,000 IU/day |  | GA 27 until delivery |
| 15 | Hossain 2014 | Asia | Pakistan | 200 | Healthy | 13.7 | Iron, calcium | | Vitamin D3 | 2 arms | Regular | Daily | 4,000 IU/day | 0 | GA 20 until delivery |
| 16 | Mutlu 2014 | Asia | Turkey | 91 | Healthy | 24.7 | - | | Vitamin D3 | 3 arms | Regular | Daily | 1,200 IU/day | 600 IU/day | GA 13-32 until delivery |
|  |  |  |  |  |  |  |  | |  |  | Regular | Daily | 2,000 IU/day |  | GA 13-32 until delivery |
| 17 | Yap 2014 | Australia New Zealand | Australia | 179 | Healthy | 44.9 | - | | Vitamin D3 | 2 arms | Regular | Daily | 5,000 IU/day | 400 IU/day | GA 20 until delivery |
| 18 | March 2015 | North America | Canada | 226 | Healthy | 68.0 | Calcium, folic acid, iron, multivitamin | | Vitamin D3 | 3 arms | Regular | Daily | 1,000 IU/day | 400 IU/day | GA 13-24 until 8 weeks postpartum |
|  |  |  |  |  |  |  |  | |  |  | Regular | Daily | 2,000 IU/day |  | GA 13-24 until 8 weeks postpartum |
| 19 | Charandabi 2015 | Asia | Iran | 124 | Healthy | - | - | | Vitamin D3 | 2 arms analyzed^c^ | Regular | Daily | 1,000 IU/day | 0 | GA 25-30for 60 days |
|  | **Author, publication year** | **Region** | **Country** | **Number of participants randomized** | **Health status at enrolment** | **Baseline 25(OH)D concentration (nmol/L)**^a^ | **Micronutrient co-intervention** | | **Vitamin D type** | **Number of arms** | **Regularity (regular or bolus)** | **Supplement frequency** | **Intervention dose**^b^ | **Comparator dose** | **Initiation and duration of supplementation** |
| 20 | Mojibian 2015 | Asia | Iran | 500 | Healthy | 38.2 | - | | Vitamin D3 | 2 arms | Regular | Every 2 weeks | 50,000/2 weeks  (=3,571 IU/day) | 400 | GA 12-16 until delivery |
| 21 | Rodda 2015 | Australia New Zealand | Australia | 78 | Healthy | 33.0 | - | | Vitamin D3 | 2 arms | Regular | Daily | 2,000 IU/day | 0 | GA 12-16 until GA 28 |
| 22 | Sablok 2015 | Asia | India | 180 | Healthy | - | - | | Vitamin D3 | 2 arms^d^ | Bolus | Once | >50 nmol/L: 60,000 IU x1 | 0 | at GA 20 if >50 nmol/L |
|  |  |  |  |  |  |  |  | |  |  | Bolus | Twice | 25-50 nmol/L: 120,000 IU x2 |  | at GA 20 and 24 if 25-50 nmol/L |
|  |  |  |  |  |  |  |  | |  |  | Bolus | Four times | <25 nmol/L: 120,000 IU x4 |  | at GA 20, 24, 28 and 32 if <25 nmol/L |
| 23 | Chawes 2016 | Europe | Denmark | 623 | Healthy | 77.4 | - | | Vitamin D3 | 2 arms | Regular | Daily | 2,800 IU/day | 400 IU/day | GA 20 until 1 week postpartum |
| 24 | Cooper 2016 | Europe | UK | 1134 | Healthy | 45.9 | - | | Vitamin D3 | 2 arms | Regular | Daily | 1,000 IU/day | 0 | GA 14 until delivery |
| 25 | Khan 2016 | Asia | Pakistan | 85 | Healthy | 31.8 | - | | Vitamin D3 | 2 arms | Regular | Daily | 4,000 IU/day | 0 | GA 12 until delivery |
| 26 | Litonjua 2016 | North America | United States | 881 | Healthy | 56.2 | Multivitamin | | Vitamin D3 | 2 arms | Regular | Daily | 4,400 IU/day | 400 IU/day | GA 10-18 until delivery |
| 27 | Naghshineh 2016 | Asia | Iran | 140 | Healthy | - | - | | Vitamin D3 | 2 arms | Regular | Daily | 600 IU/day | 0 | GA 16 until delivery |
| 28 | Sahoo 2016 | Asia | India | 300 | Healthy | 33.0 | Calcium | | Vitamin D3 | 3 arms | Regular | Every 4 weeks | 60,000 IU/  4 weeks  (=2,143 IU/day) | 400 IU/day | GA 14-20 every 4 weeks until delivery |
|  |  |  |  |  |  |  |  | |  |  | Regular | Every 8 weeks | 60,000 IU/  8 weeks  (=1,071 IU/day) |  | GA 14-20 every 8 weeks until delivery |
| 29 | Shahgheibi 2016 | Asia | Iran | 100 | Healthy | 17.4 | - | | unreported | 2 arms | Regular | Weekly | 5,000 IU/week  (-714 IU/day) | 0 | GA 12 until GA 26 |
| 30 | Thiele 2016 | North America | United States | 16 | Healthy | - | - | | Vitamin D3 | 2 arms | Regular | Daily | 3,800 IU/day | 400 IU/day | GA 28 until 4 weeks postpartum |
| 31 | Valizadeh 2016 | Asia | Iran | 96 | GDM | 44.2 | - | | Vitamin D3 | 2 arms | Bolus and regular | Daily and weekly | if <28 weeks gestation; 200,000 IU/day x2 + 50,000 IU/week x6  (a total of 700,000 IU) | 0 | GA 12-32 |
|  |  |  |  |  |  |  |  | |  |  | Bolus and regular | Daily and weekly | If ≥28 weeks gestation: 200,000 IU/day x2 + 100,000 IU/week x3  (a total of 700,000 IU) |  | GA 12-32 |
| 32 | Vaziri 2016 | Asia | Iran | 153 | Healthy | 31.8 | - | | Vitamin D3 | 2 arms | Regular | Daily | 2,000 IU/day | 0 | GA 26-28 until delivery |
| 33 | Yazdchi 2016 | Asia | Iran | 76 | Healthy | 25.8 | Folic acid, iron | | Vitamin D3 | 2 arms | Regular | Every 2 weeks | 50,000 IU/  2 weeks  (=3,571 IU/day) | 0 | GA 24-28 for 2 months |
| 34 | Zerofsky 2014 | North America | United States | 57 | Healthy | 69.6 | - | | Vitamin D3 | 2 arms | Regular | Daily | 2,000 IU/day | 400 IU/day | GA 20 until delivery |
| 35 | Abotorabi 2017 | Asia | Iran | 110 | Vitamin D deficiency+  hypocalcemia | 45.0 | Prenatal capsule containing calcium | | Vitamin D3 | 2 arms | Regular | Weekly | 50,000 IU/week | 400 IU/day | GA 22-26 for 8 weeks |
|  | **Author, publication year** | **Region** | **Country** | **Number of participants randomized** | **Health status at enrolment** | **Baseline 25(OH)D concentration (nmol/L)**^a^ | **Micronutrient co-intervention** | | **Vitamin D type** | **Number of arms** | **Regularity (regular or bolus)** | **Supplement frequency** | **Intervention dose**^b^ | **Comparator dose** | **Initiation and duration of supplementation** |
| 36 | Samimi 2017 | Asia | Iran | 80 | Healthy | 28.8 | Vaginal progesterone | | Vitamin D3 | 2 arms | Regular | Daily | 400 IU/day | 0 | < GA 10 until GA 20 |
| 37 | Tehrani 2017 | Asia | Iran | 140 | Healthy | - | - | | Vitamin D3 | 2 arms analyzed | Regular | Every 2 weeks | 50,000 IU/  2 weeks  (=3,571 IU/day) | 0 | GA 14-16 for 10 weeks |
| 38 | Sasan 2017 | Asia | Iran | 142 | Healthy | - | - | | Vitamin D3 | 2 arms | Regular | Every 2 weeks | 50,000 IU/  2 weeks  (=3,571 IU/day) | 0 | until GA 36 |
| 39 | Azadehrah 2017 | Asia | Iran | 90 | Vitamin D deficiency | 31.3 | - | | Unreported | 2 arms | Regular | Daily | 50,000 IU/day | 200 IU/day | GA 12-16 for eight weeks |
| 40 | Keshavarzi 2017 | Asia | Iran | 128 | GDM | 50.7 | - | | unreported | 2 arms | Bolus | Twice | 50,000 IU | 400 IU/day | The first at GA 24-34, the second 3 weeks later |
| 41 | Best 2018 | North America | United States | 83 | Healthy | 57.7 | - | | Vitamin D3 | 2 arms | Regular | Daily | 2,400 IU/day | 600 IU/day | GA 12-29 for 5 months |
| 42 | O’Callaghan 2018 | Europe | Ireland | 144 | Healthy | 57.2 | - | | Vitamin D3 | 3 arms | Regular | Daily | 400 IU/day | 0 | GA 8-18 until GA 36 |
|  |  |  |  |  |  |  |  | |  |  | Regular | Daily | 800 IU/day |  | GA 8-18 until GA 36 |
| 43 | Roth 2018 | Asia | Bangladesh | 1300 | Healthy | 27.6 | - | | Vitamin D3 | 5 arms | Regular | Weekly | 4,200 IU/week  (=600 IU/day) | 0 | GA 17-24 until delivery |
|  |  |  |  |  |  |  |  | |  |  | Regular | Weekly | 16,800 IU/week  (=2,400 IU/day) |  | GA 17-24 until delivery |
|  |  |  |  |  |  |  |  | |  |  | Regular | Weekly | 28,000 IU/week  (= 2,400 IU/day) |  | GA 17-24 until delivery |
|  |  |  |  |  |  |  |  | |  |  | Regular | Weekly | 28,000 IU/week  (= 4,000 IU/day) |  | GA 17-24 until 26 weeks postpartum |
| 44 | Ali 2019 | Asia | Saudi Arabia | 179 | Healthy | 17.5 | - | | Vitamin D3 | 2 arms | Regular | Daily | 4,000 IU/day | 400 IU/day | GA 13 until delivery |
| 45 | Enkhmaa 2019 | Asia | Mongolia | 360 | Healthy | 18.0 | Prenatal vitamins containing calcium | | Vitamin D3 | 3 arms | Regular | Daily | 2,000 IU/day | 600 IU/day | GA 12-16 until delivery |
|  |  |  |  |  |  |  |  | |  |  |  | Daily | 4,000 IU/day |  | GA 12-16 until delivery |
| 46 | Jefferson 2019 | North America | United States | 387 | Healthy | - | Prenatal vitamins | | Vitamin D3 | 2 arms | Regular | Daily | 4,400 IU/day | 400 IU/day | GA 14 until delivery |
| 47 | Zhao 2019 | Asia | China | 101 | GDM risk factors | 58.2 | Calcium | | Unreported | 2 arms | Regular | Daily | 700 IU/day | 100 IU/day | enrollment until delivery |
| 48 | Corcoy 2020 | Europe | UK Ireland Austria  Poland  Italy  Spain  Belgium | 154 | pre-pregnancy BMI ≥29 | 69.6 | - | | Vitamin D3 | 2 arms | Regular | Daily | 1,600 IU/day | 0 | ≤ GA 19 weeks and 6 days until delivery |
| 49 | Rahbar 2020 | Asia | Iran | 180 | Vitamin D deficiency | 38.2 | - | | Vitamin D3 | 2 arms | Regular | Daily and every 2 weeks | 400 IU daily + 50,000 IU every 2 weeks | 400 IU/day | GA 14-16 for 6 weeks |
| 50 | Sircar 2020 | Asia | India | 178 | Vitamin D deficiency + GDM risk factors | - | Iron, calcium, vitamin B, and vitamin C | | Vitamin D3 | 2 arms | Regular | Weekly | 60,000 IU/week (=8571 IU/day) | 0 | GA 13-24 until GA 26 |
| 51 | Alhomaid 2021 | Europe | Northern Ireland | 240 | Healthy | 52.2 | - | | Vitamin D3 | 2 arms | Regular | Daily | 800 IU/day | 400 IU/day | GA 12 until delivery |
|  | **Author, publication year** | **Region** | **Country** | **Number of participants randomized** | **Health status at enrolment** | **Baseline 25(OH)D concentration (nmol/L)**^a^ | **Micronutrient co-intervention** | | **Vitamin D type** | **Number of arms** | **Regularity (regular or bolus)** | **Supplement frequency** | **Intervention dose**^b^ | **Comparator dose** | **Initiation and duration of supplementation** |
| 52 | Manasova 2021 | Europe | Ukraine | 54 | Vitamin D deficiency + preeclampsia risk factors | 65.1 | Multivitamin | | Vitamin D3 | 2 arms | Regular | Daily | 4,500 IU/day from 10-12 weeks, changed to 2,500 IU/day till delivery | 500 IU/day | GA 10-12 until delivery |
| 53 | Mohammadi 2021 | Asia | Iran | 36 | GDM | 47.7 | Folic acid, ferrous sulfate | | Vitamin D3 | 2 arms | Regular | Daily | 2,000 IU/day | 0 | From enrollment to 6 weeks |
| 54 | Nausheen 2021 | Asia | Pakistan | 350 | Healthy | 19.7 | - | | Vitamin D3 | 3 arms | Regular | Daily | 2,000 IU/day | 400 IU/day | GA 12-16 until delivery |
|  |  |  |  |  |  |  |  | |  |  | Regular | Daily | 4,000 IU/day |  | GA 12-16 until delivery |
| 55 | Srilekha 2021 | Asia | India | 100 | Healthy | 60.4 | - | | intravenous vitamin D3 | 2 arms | Unreported | Unreported | 6,000 IU/day | 0 | for 3 months |
| 56 | Xiaomang 2021 | Asia | China | 450 | Vitamin D deficiency | - | - | | Vitamin D3 | 3 arms | Regular | Daily | 1,500 IU/day | 400 IU/day | GA 13 |
|  |  |  |  |  |  |  |  | |  |  | Regular | Daily | 4000 IU/day |  | GA 13 |
| 57 | Aggarwal 2022 | Asia | India | 297 | Healthy | 22.6 | - | | Vitamin D3 | 2 arms | Regular | Daily | 60000 IU/day | 0 | GA 12-16 for 5 days |
| 58 | Memon 2022 | Asia | Pakistan | 90 | Healthy | - | - | | Vitamin D3 | 2 arms | Regular | Every 2 weeks | 25000 IU/  2 weeks  (=1,786 IU/day) | 0 | GA 24 until GA 36 |
| 59 | Pulido 2022 | North America | Mexico | 54 | GDM | 61.6 | - | | Vitamin D3 | 2 arms | Regular | Daily | 5,000 IU/day | 0 | GA 24 for 8 weeks |
| 60 | Sudfeld 2022 | Africa | Tanzania | 2300 | HIV | 81.4 | - | | Vitamin D3 | 2 arms | Regular | Daily | 3,000 IU/day | 0 | GA 12-27 until 1 year after delivery |
| 61 | Mirzaei-Azandaryani 2022 | Asia | Iran | 88 | Vitamin D deficiency | 43.7 | - | | Vitamin D3 | 2 arms | Regular | Daily | 4,000 IU/day | 0 | GA 8-10 until the end of GA 26 |
| 62 | Vanda 2022 | Asia | Iran | 130 | Healthy | - | - | | Unreported | 2 arms | Regular | Daily | 2,000 IU/day | 500 IU/day | GA 12 until delivery |
| 63 | Cagiran 2023 | Asia | Turkey | 40 | Vitamin D deficiency | 34.7 | - | | Vitamin D3 | 2 arms analyzed^e^ | Regular | Daily | 600 IU/day | 400 IU/day | GA 14 until delivery |
| 64 | Ma 2023 | Asia | China | 1720 | Vitamin D deficiency | 38.0 | - | | Vitamin D3 | 2 arms | Regular | Daily | 1,600 IU/day | 400 IU/day | GA 24-28 for 2 months |
| 65 | Nadeem 2023 | Asia | Pakistan | 34 | Vitamin D deficiency + GDM | 40.0 | - | | Vitamin D3 | 2 arms | Bolus | Once | 200,000 IU | 0 | once |
| 66 | Sunarno 2023 | Asia | Indonesia | 72 | Healthy | 30.2 | Iron, calcium, and vitamin C | | Vitamin D3 | 2 arms | Regular | Daily | 1,000 IU/day | 0 | GA 17 |

^a^ Maternal mean baseline 25(OH)D concentration was determined by the value of the control group.

^b^ For regular doses other than daily, the dosage was converted to daily (IU/day).

^c^ Charandabi et al. (2015) has 3 arms: one received calcium and vitamin D supplementation; one received vitamin D supplementation; one received placebo. The latter two arms were presented and used for analysis.

^d^ Sablok et al. (2015) has 2 arms: intervention and control groups. The intervention group received supplementation depending upon 25(OH)D concentration.

^e^ Cagriran et al. (2023) has 3 arms: participants with vitamin D level below 20 ng/ml receiving either 600 IU/day or 400 IU/day, and participants with vitamin D level above 20 ng/ml receiving no supplementation. The first two arms were used for analysis.

**Table S7. Subgroup analyses**

| **Subgroup** | **Preeclampsia** | | | | **Gestational hypertension** | | | | **Gestational diabetes** | | | |
| --- | --- | --- | --- | --- | --- | --- | --- | --- | --- | --- | --- | --- |
|  | **Number of trials** | **Number of participants** | **Risk Ratio   (95% CI)** | **I^2^ %  (p-value)** | **Number of trials** | **Number of participants** | **Risk Ratio**  **(95% CI)** | **I^2^ %  (p-value)** | **Number of trials** | **Number of participants** | **Risk Ratio  (95% CI)** | **I^2^ %  (p-value)** |
| Primary analysis | 6 | 1483 | 0.81 (0.43, 1.53) | 41.7% (0.13) | 5 | 2262 | 1.23 (0.73, 2.07) | 0.0% (0.92) | 12 | 1992 | 0.65 (0.49, 0.86) | 22.6% (0.22) |
| **Intervention type** |  |  |  |  |  |  |  |  |  |  |  |  |
| Vitamin D alone *versus* placebo (or no intervention) | 1 | 142 | 0.51 (0.27, 0.98) | NA | 1 | 1298 | 0.93 (0.31, 2.79) | NA | 4 | 442 | 0.60 (0.35, 1.00) | 20.3% (0.29) |
| Vitamin D + calcium and/or other vitamins and/or minerals *versus* calcium and/or other vitamins and/or minerals | 1 | 175 | 1.72 (0.66, 4.54) | NA | 1 | 175 | 1.63 (0.66, 4.00) | NA | 2 | 353 | 0.81 (0.21, 3.18) | 82.6% (0.02) |
| Vitamin D *versus* vitamin D of a lower dose (upto 600 IU/day)^a^ | 3 | 806 | 0.68 (0.16, 2.89) | 53.5% (0.12) | 2 | 429 | 1.34 (0.31, 5.82) | 0.0% (0.61) | 5 | 1096 | 0.67 (0.42, 1.06) | 14.6% (0.32) |
| Vitamin D + calcium and/or other vitamins and/or minerals *versus* vitamin D of a lower dose (upto 600IU/day)^a^ + calcium and/or other vitamins and/or minerals | 1 | 360 | 0.74 (0.21, 2.57) | NA | 1 | 360 | 1.07 (0.42, 2.74) | NA | 1 | 101 | 0.64 (0.31, 1.32) | NA |
| **Population type** |  |  |  |  |  |  |  |  |  |  |  |  |
| General population | 6 | 1483 | 0.81 (0.43, 1.53) | 41.7% (0.13) | 4 | 2222 | 1.25 (0.73, 2.16) | 0.0% (0.82) | 6 | 1283 | 0.74 (0.43, 1.26) | 50.9% (0.07) |
| Population with morbidities | - | - | - | - | 1 | 40 | 1.00 (0.16, 6.42) | NA | 6 | 709 | 0.60 (0.43, 0.83) | 0.0% (0.58) |
| **Intervention dose versus placebo pair^b^** |  |  |  |  |  |  |  |  |  |  |  |  |
| Intervention dose ≤600 IU/day | - | - | - | - | 2 pairs | 365 | 0.65 (0.14, 3.03) | 0.0% (0.41) | 1 pair | 40 | 3.00 (0.13, 69.4) | NA |
| Intervention dose >600 to ≤2000 IU/day | 2 pairs | 310 | 0.67 (0.15, 2.94) | 0.0% (0.84) | 1 pair | 181 | 0.98 (0.25, 3.80) | NA | 4 pairs | 459 | 0.65 (0.37, 1.12) | 35.9% (0.20) |
| Intervention dose >2000 IU/day | 6 pairs | 1173 | 0.83 (0.42, 1.65) | 41.1% (0.13) | 5 pairs | 1717 | 1.42 (0.76, 2.62) | 0.0% (0.98) | 8 pairs | 1493 | 0.65 (0.46, 0.93) | 21.0% (0.27) |
| **Administration frequency** |  |  |  |  |  |  |  |  |  |  |  |  |
| Regular | 6 | 1483 | 0.81 (0.43, 1.53) | 41.7% (0.13) | 5 | 2262 | 1.23 (0.73, 2.07) | 0.0% (0.92) | 12 | 1992 | 0.65 (0.49, 0.86) | 22.6% (0.22) |
| Bolus | - | - | - | - | - | - | - | - | - | - | - | - |
| **Vitamin D supplementation form** |  |  |  |  |  |  |  |  |  |  |  |  |
| Vitamin D3 | 6 | 1483 | 0.81 (0.43, 1.53) | 41.7% (0.13) | 5 | 2262 | 1.23 (0.73, 2.07) | 0.0% (0.92) | 10 | 1804 | 0.70 (0.50, 0.97) | 25.4% (0.21) |
| Vitamin D2 | - | - | - | - | - | - | - | - | - | - | - | - |
| **Trimester at supplementation initiation** |  |  |  |  |  |  |  |  |  |  |  |  |
| 1^st^ trimester | 1 | 164 | 0.16 (0.02, 1.32) | NA | - | - | - | - | 4 | 437 | 0.52 (0.33, 0.83) | 0.0% (0.52) |
| 2^nd^ trimester | 1 | 175 | 1.72 (0.66, 4.54) | NA | 3 | 1513 | 1.26 (0.66, 2.42) | 0.0% (0.72) | 5 | 654 | 0.88 (0.59, 1.31) | 0.0% (0.50) |
| 3^rd^ trimester | - | - | - | - | - | - | - | - | - | - | - | - |
| **Maternal baseline 25(OH)D concentration <30 nmol/L**^c^ | |  |  |  |  |  |  |  |  |  |  |  |
| 25(OH)D <30 nmol/L | 4 | 952 | 0.78 (0.32, 1.93) | 35.5% (0.20) | 3 | 1833 | 1.21 (0.69, 2.12) | 0.0% (0.71) | 3 | 515 | 1.03 (0.29, 3.72) | 75.3% (0.02) |
| 25(OH)D ≥30 nmol/L | 1 | 389 | 1.91 (0.57, 6.42) | NA | 2 | 428 | 1.34 (0.31, 5.82) | 0.0% (0.61) | 7 | 1159 | 0.62 (0.46, 0.85) | 0.0% (0.82) |
| **Maternal baseline 25(OH)D concentration <50 nmol/L**^c^ | |  |  |  |  |  |  |  |  |  |  |  |
| 25(OH)D <50 nmol/L | 5 | 1341 | 0.99 (0.48, 2.07) | 30.1% (0.22) | 5 | 2262 | 1.23 (0.73, 2.07) | 0.0% (0.92) | 8 | 1432 | 0.69 (0.43, 1.12) | 40.2% (0.11) |
| 25(OH)D ≥50 nmol/L | - | - | - | - | - | - | - | - | 2 | 242 | 0.72 (0.45, 1.14) | 0.0% (0.68) |

^a^ The comparator group was vitamin D of a lower dose (upto 600 IU/day, or its equivalence, for example, 4200 IU/week).

^b^ The subgroup analysis by intervention dose was performed based on intervention versus control comparison pairs because some trials have multiple intervention arms.

^c^ The subgroup was determined by the 25(OH)D concentration of the control group.

**Table S7. Subgroup analyses (continued)**

| **Subgroup** | **Preterm labor** | | | | **Cesarean delivery** | | | | **Maternal hospitalization** | | | |
| --- | --- | --- | --- | --- | --- | --- | --- | --- | --- | --- | --- | --- |
|  | **Number of trials** | **Number of participants** | **Risk Ratio   (95% CI)** | **I^2^ %  (p-value)** | **Number of trials** | **Number of participants** | **Risk Ratio**  **(95% CI)** | **I^2^ %  (p-value)** | **Number of trials** | **Number of participants** | **Risk Ratio  (95% CI)** | **I^2^ %  (p-value)** |
| Primary analysis | 7 | 1276 | 0.70 (0.44, 1.11) | 37.0% (0.15) | 26 | 7199 | 1.05 (0.99, 1.12) | 0.0% (0.73) | 5 | 1776 | 0.74 (0.42, 1.32) | 55.1% (0.06) |
| **Intervention type** |  |  |  |  |  |  |  |  |  |  |  |  |
| Vitamin D alone *versus* placebo (or no intervention) | 4 | 666 | 0.50 (0.25, 0.99) | 42.3% (0.16) | 12 | 3641 | 1.06 (0.97, 1.15) | 1.4% (0.43) | 2 | 201 | 0.38 (0.12, 1.26) | 37% (0.21) |
| Vitamin D + calcium and/or other vitamins and/or minerals *versus* calcium and/or other vitamins and/or minerals | - | - | - | - | 1 | 175 | 1.10 (0.84, 1.45) | NA | 1 | 76 | 3.00 (0.13, 71.37) | NA |
| Vitamin D *versus* vitamin D of a lower dose (upto 600 IU/day)^a^ | 3 | 610 | 1.03 (0.61, 1.74) | 0.0% (0.88) | 11 | 2232 | 1.00 (0.88, 1.13) | 0.0% (0.57) | 1 | 623 | 1.06 (0.62, 1.80) | NA |
| Vitamin D + calcium and/or other vitamins and/or minerals *versus* vitamin D of a lower dose (upto 600IU/day)^a^ + calcium and/or other vitamins and/or minerals | - | - | - | - | 2 | 1151 | 1.07 (0.88, 1.30) | 0.0% (0.70) | 1 | 876 | 0.88 (0.56, 1.39) | NA |
| **Population type** |  |  |  |  |  |  |  |  |  |  |  |  |
| General population | 5 | 1102 | 0.68 (0.36, 1.28) | 56.7% (0.06) | 20 | 6347 | 1.06 (0.99, 1.13) | 0.0% (0.65) | 4 | 1722 | 0.97 (0.69, 1.35) | 0.0% (0.86) |
| Population with morbidities | 2 | 174 | 0.78 (0.36, 1.67) | 0.0% (0.65) | 6 | 852 | 1.03 (0.88, 1.19) | 0.0% (0.53) | 1 | 54 | 0.26 (0.11, 0.60) | NA |
| **Intervention dose versus placebo pair^b^** |  |  |  |  |  |  |  |  |  |  |  |  |
| Intervention dose ≤600 IU/day | 1 pair | 138 | 0.24 (0.09, 0.68) | NA | 3 pairs | 494 | 1.09 (0.90, 1.31) | 0.0% (0.71) | - | - | - | - |
| Intervention dose >600 to ≤2000 IU/day | 1 pair | 131 | 1.02 (0.41, 2.54) | NA | 11 pairs | 2283 | 1.04 (0.92, 1.17) | 0.0% (0.95) | - | - | - | - |
| Intervention dose >2000 IU/day | 3 pairs | 758 | 1.09 (0.63, 1.87) | 0.0% (0.85) | 14 pairs | 4001 | 1.06 (0.97, 1.17) | 8.6% (0.36) | 4 pairs | 1722 | 0.97 (0.69, 1.35) | 0% (0.86) |
| **Administration frequency** |  |  |  |  |  |  |  |  |  |  |  |  |
| Regular | 5 | 1027 | 0.82 (0.47, 1.43) | 42.0% (0.14) | 22 | 6664 | 1.06 (0.99, 1.13) | 0.0% (0.68) | 5 | 1776 | 0.74 (0.42, 1.32) | 55.1% (0.06) |
| Bolus | 1 | 165 | 0.40 (0.18, 0.88) | NA | 2 | 293 | 0.97 (0.63, 1.48) | 26.6% (0.24) | - | - | - | - |
| **Vitamin D supplementation form** |  |  |  |  |  |  |  |  |  |  |  |  |
| Vitamin D3 | 5 | 1055 | 0.61 (0.32, 1.17) | 51.8% (0.08) | 22 | 6690 | 1.05 (0.98, 1.12) | 0.0% (0.53) | 5 | 1776 | 0.74 (0.42, 1.32) | 55.1% (0.06) |
| Vitamin D2 | - | - | - | - | - | - | - | - | - | - | - | - |
| **Trimester at supplementation initiation** |  |  |  |  |  |  |  |  |  |  |  |  |
| 1^st^ trimester | 1 | 131 | 1.02 (0.41, 2.54) | NA | 6 | 1197 | 0.95 (0.79, 1.14) | 19.5% (0.29) | - | - | - | - |
| 2^nd^ trimester | 2 | 303 | 0.33 (0.17, 0.62) | 0.0% (0.46) | 12 | 3970 | 1.05 (0.96, 1.14) | 0.0% (0.96) | 2 | 770 | 1.06 (0.63, 1.76) | 0.0% (0.97) |
| 3^rd^ trimester | - | - | - | - | - | - | - | - | - | - | - | - |
| **Maternal baseline 25(OH)D concentration <30 nmol/L**^c^ | |  |  |  |  |  |  |  |  |  |  |  |
| 25(OH)D <30 nmol/L | 1 | 279 | 1.23 (0.44, 3.46) | NA | 7 | 2467 | 1.10 (0.99, 1.22) | 0% (0.66) | 1 | 76 | 3.00 (0.13, 71.37)) | NA |
| 25(OH)D ≥30 nmol/L | 4 | 728 | 0.71 (0.42, 1.20) | 19.2% (0.29) | 14 | 3830 | 0.99 (0.91, 1.09) | 0% (0.64) | 4 | 1700 | 0.71 (0.39, 1.29) | 63.6% (0.04) |
| **Maternal baseline 25(OH)D concentration <50 nmol/L**^c^ | |  |  |  |  |  |  |  |  |  |  |  |
| 25(OH)D <50 nmol/L | 5 | 1007 | 0.78 (0.49,1.24) | 14.3% (0.32) | 13 | 4027 | 1.05 (0.96, 1.14) | 0% (0.72) | 2 | 223 | 1.36 (0.26, 7.09) | 0.0% (0.57) |
| 25(OH)D ≥50 nmol/L | - | - | - | - | 78 | 1370 | 1.01 (0.89, 1.15) | 0% (0.34) | 3 | 1553 | 0.68 (0.34, 1.34) | 75.5% (0.02) |

**Table S7. Subgroup analyses (continued)**

| **Subgroup** | **Maternal 25(OH)D concentration at or near delivery (nmol/L)** | | | | **Stillbirth or intrauterine death** | | | | **Low birthweight infant** | | | |
| --- | --- | --- | --- | --- | --- | --- | --- | --- | --- | --- | --- | --- |
|  | **Number of trials** | **Number of participants** | **Mean difference (95%CI)** | **I^2^ %  (p-value)** | **Number of trials** | **Number of participants** | **Risk Ratio**  **(95% CI)** | **I^2^ %  (p-value)** | **Number of trials** | **Number of participants** | **Risk Ratio  (95% CI)** | **I^2^ %  (p-value)** |
| Primary analysis | 48 | 10064 | 33.96 (28.16, 39.76) | 97.6% (<0.01) | 21 | 9186 | 0.82 (0.63, 1.07) | 0.0% (0.60) | 13 | 5044 | 0.95 (0.76, 1.19) | 27.5% (0.17) |
| **Intervention type** |  |  |  |  |  |  |  |  |  |  |  |  |
| Vitamin D alone *versus* placebo (or no intervention) | 20 | 3682 | 38.66 (27.24, 50.07) | 98.4% (<0.01) | 11 | 5966 | 0.79 (0.56, 1.11) | 0.0% (0.79) | 7 | 3665 | 0.82 (0.60, 1.14) | 52.2% (0.05) |
| Vitamin D + calcium and/or other vitamins and/or minerals *versus* calcium and/or other vitamins and/or minerals | 4 | 258 | 34.49 (21.31, 47.67) | 89.2% (<0.01) | 2 | 252 | 0.61 (0.11, 3.48) | 35.8% (0.21) | 1 | 175 | 1.09 (0.62, 1.94) | NA (NA) |
| Vitamin D *versus* vitamin D of a lower dose (upto 600 IU/day)^a^ | 17 | 4500 | 28.70 (20.54, 36.86) | 96.4% (<0.01) | 6 | 1732 | 1.01 (0.45, 2.27) | 26.2% (0.24) | 4 | 866 | 1.28 (0.82, 2.00) | 0.0% (0.80) |
| Vitamin D + calcium and/or other vitamins and/or minerals *versus* vitamin D of a lower dose (upto 600IU/day)^a^ + calcium and/or other vitamins and/or minerals | 7 | 1624 | 35.73 (21.05, 50.40) | 96.6% (<0.01) | 2 | 1236 | 1.14 (0.55, 2.32) | 0.0% (0.86) | 1 | 338 | 0.32 (0.05, 1.87) | NA (NA) |
| **Population type** |  |  |  |  |  |  |  |  |  |  |  |  |
| General population | 36 | 7319 | 34.34 (27.28, 41.40) | 97.8% (<0.01) | 18 | 6753 | 0.77 (0.54, 1.10) | 0.0% (0.53) | 11 | 2746 | 0.90 (0.67, 1.20) | 36.1% (0.11) |
| Population with morbidities | 12 | 2745 | 33.15 (20.74, 45.57) | 96.8% (<0.01) | 3 | 2433 | 0.89 (0.61, 1.31) | 0.0% (0.47) | 2 | 2298 | 1.06 (0.82, 1.36) | 0.0% (0.42) |
| **Intervention dose versus placebo pair^b^** |  |  |  |  |  |  |  |  |  |  |  |  |
| Intervention dose ≤600 IU/day | 2 pairs | 211 | 36.27 (15.49, 57.05) | 86.4% (<0.01) | 2 pairs | 402 | 0.41 (0.18, 0.98) | 0.0% (0.58) | 1 pair | 213 | 1.23 (0.69, 2.16) | NA |
| Intervention dose >600 to ≤2000 IU/day | 28 pairs | 4878 | 24.83 (20.09, 29.57) | 92.8% (<0.01) | 10 pairs | 2307 | 0.91 (0.46, 1.80) | 0.0% (0.45) | 5 pairs | 726 | 0.82 (0.49, 1.37) | 0.0% (0.46) |
| Intervention dose >2000 IU/day | 26 pairs | 4515 | 42.63 (30.96, 54.29) | 98.2% (<0.01) | 14 pairs | 6303 | 0.90 (0.67, 1.22) | 0.0% (0.94) | 10 pairs | 3905 | 1.06 (0.89, 1.25) | 0.0% (0.95) |
| **Administration frequency** |  |  |  |  |  |  |  |  |  |  |  |  |
| Regular | 42 | 9425 | 35.36 (28.83, 41.89) | 97.7% (<0.01) | 19 | 8923 | 0.83 (0.64, 1.08) | 0.0% (0.54) | 12 | 4844 | 1.04 (0.89, 1.22) | 0.0% (0.74) |
| Bolus | 3 | 283 | 32.95 (18.98, 46.92) | 75.1% (0.02) | - | - | - | - | 1 | 200 | 0.21 (0.07, 0.60) | NA |
| **Vitamin D supplementation form** |  |  |  |  |  |  |  |  |  |  |  |  |
| Vitamin D3 | 42 | 9391 | 33.85 (27.60, 40.11) | 97.5% (<0.01) | 19 | 8876 | 0.79 (0.61, 1.04) | 0.0% (0.82) | 12 | 4918 | 1.00 (0.80, 1.23) | 22.1% (0.23) |
| Vitamin D2 | 2 | 203 | 83.45 (-49.34, 216.24) | 99.1% (<0.01) | - | - | - | - | 1 | 126 | 0.53 (0.23, 1.21) | NA |
| **Trimester at supplementation initiation** |  |  |  |  |  |  |  |  |  |  |  |  |
| 1^st^ trimester | 9 | 1134 | 35.45 (15.56, 55.33) | 97.4 % (<0.01) | 4 | 797 | 0.77 (0.27, 2.17) | 62.5% (0.05) | 1 | 62 | 0.82 (0.37, 1.84) | NA |
| 2^nd^ trimester | 17 | 3709 | 43.48 (31.84, 55.11) | 98.2% (<0.01) | 9 | 4037 | 0.64 (0.34, 1.22) | 0.0% (0.83) | 4 | 1291 | 1.07 (0.84, 1.36) | 0.0% (0.77) |
| 3^rd^ trimester | 3 | 216 | 59.14 (24.56, 93.72) | 98.2% (<0.01) | - | - | - | - | 2 | 326 | 0.35 (0.14, 0.87) | 46.0% (0.17) |
| **Maternal baseline 25(OH)D concentration <30 nmol/L**^c^ | |  |  |  |  |  |  |  |  |  |  |  |
| 25(OH)D <30 nmol/L | 11 | 844 | 40.59 (22.49, 58.69) | 98.8% (<0.01) | 7 | 2639 | 0.50 (0.28, 0.89) | 0.0% (0.68) | 5 | 1727 | 1.02 (0.78, 1.33) | 15.7% (0.31) |
| 25(OH)D ≥30 nmol/L | 34 | 7745 | 33.05 (27.02, 39.09) | 96.5% (<0.01) | 12 | 6274 | 0.90 (0.67, 1.22) | 0.0% (0.90) | 7 | 3117 | 1.05 (0.84, 1.30) | 0.0% (0.82) |
| **Maternal baseline 25(OH)D concentration <50 nmol/L**^c^ | |  |  |  |  |  |  |  |  |  |  |  |
| 25(OH)D <50 nmol/L | 29 | 6572 | 39.41 (30.80, 48.01) | 98.3% (<0.01) | 13 | 4335 | 0.55 (0.33, 0.90) | 0.0% (0.86) | 8 | 2322 | 1.03 (0.84, 1.28) | 0.0% (0.54) |
| 25(OH)D ≥50 nmol/L | 16 | 3368 | 25.13 (19.95 30.47) | 85.4% (<0.01) | 6 | 4578 | 0.92 (0.67, 1.26) | 0.0% (0.73) | 4 | 2522 | 1.07 (0.83, 1.37) | 0.0% (0.66) |

**Table S7. Subgroup analyses (continued)**

| **Subgroup** | **Preterm birth** | | | | **Small for gestational age infant** | | | | **Congenital malformations** | | | |
| --- | --- | --- | --- | --- | --- | --- | --- | --- | --- | --- | --- | --- |
|  | **Number of trials** | **Number of participants** | **Risk Ratio   (95% CI)** | **I^2^ %  (p-value)** | **Number of trials** | **Number of participants** | **Risk Ratio**  **(95% CI)** | **I^2^ %  (p-value)** | **Number of trials** | **Number of participants** | **Risk Ratio  (95% CI)** | **I^2^ %  (p-value)** |
| Primary analysis | 18 | 8446 | 1.01 (0.85, 1.19) | 20.7% (0.21) | 9 | 4211 | 0.92 (0.75, 1.13) | 28.6% (0.19) | 6 | 4042 | 0.78 (0.49, 1.22) | 32.6% (0.19) |
| **Intervention type** |  |  |  |  |  |  |  |  |  |  |  |  |
| Vitamin D alone *versus* placebo (or no intervention) | 7 | 5002 | 1.07 (0.83, 1.38) | 22.0% (0.26) | 6 | 3602 | 0.90 (0.71, 1.14) | 49.7% (0.08) | 4 | 2582 | 0.82 (0.33, 2.00) | 48.5% (0.12) |
| Vitamin D + calcium and/or other vitamins and/or minerals *versus* calcium and/or other vitamins and/or minerals | 2 | 250 | 1.16 (0.54, 2.49) | 0.0% (0.43) | - | - | - | - | - | - | - | - |
| Vitamin D *versus* vitamin D of a lower dose (upto 600 IU/day)^a^ | 7 | 2050 | 0.88 (0.65, 1.19) | 22.5% (0.26) | 1 | 162 | 0.64 (0.18, 2.30) | NA | 1 | 584 | 0.71 (0.39, 1.31) | NA |
| Vitamin D + calcium and/or other vitamins and/or minerals *versus* vitamin D of a lower dose (upto 600IU/day)^a^ + calcium and/or other vitamins and/or minerals | 2 | 1144 | 1.03 (0.58, 1.84) | 40.9% (0.19) | 2 | 447 | 1.08 (0.18, 6.54) | 0.0% (0.37) | 1 | 876 | 1.06 (0.52, 2.17) | NA |
| **Population type** |  |  |  |  |  |  |  |  |  |  |  |  |
| General population | 14 | 5679 | 0.96 (0.77, 1.19) | 16.4% (0.27) | 7 | 1914 | 0.78 (0.53, 1.15) | 38.9% (0.13) | 5 | 3958 | 0.78 (0.48, 1.29) | 45.7% (0.12) |
| Population with morbidities | 4 | 2767 | 1.10 (0.87, 1.40) | 15.9% (0.31) | 2 | 2297 | 0.77 (0.23, 2.61) | 26.9% (0.24) | 1 | 84 | 1.00 (0.06, 15.47) | NA |
| **Intervention dose versus placebo pair^b^** |  |  |  |  |  |  |  |  |  |  |  |  |
| Intervention dose ≤600 IU/day | 1 pair | 316 | 0.85 (0.36, 2.02) | NA | 1 pair | 213 | 1.19 (0.81, 1.73) | NA | 1 pair | 316 | 0.36 (0.11, 1.25) | NA |
| Intervention dose >600 to ≤2000 IU/day | 9 pairs | 2114 | 0.84 (0.61, 1.17) | 0.0% (0.44) | 5 pairs | 601 | 0.59 (0.34, 1.03) | 0.0% (0.53) | 1 pair | 965 | 1.62 (0.53, 4.93) | NA |
| Intervention dose >2000 IU/day | 15 pairs | 6016 | 1.12 (0.99, 1.26) | 0.0% (0.61) | 6 pairs | 3144 | 1.03 (0.90, 1.17) | 0.0% (0.93) | 5 pairs | 2677 | 0.69 (0.46, 1.04) | 9.3 % (0.35) |
| **Administration frequency** |  |  |  |  |  |  |  |  |  |  |  |  |
| Regular | 18 | 8446 | 1.01 (0.85, 1.19) | 20.7% (0.21) | 7 | 3867 | 1.00 (0.85, 1.17) | 13.0% (0.33) | 5 | 3958 | 0.78 (0.48, 1.29) | 45.7% (0.12) |
| Bolus | - | - | - | - | 1 | 165 | 0.43 (0.19, 0.98) | NA | 1 | 84 | 1.00 (0.06, 15.47) | NA |
| **Vitamin D supplementation form** |  |  |  |  |  |  |  |  |  |  |  |  |
| Vitamin D3 | 18 | 8446 | 1.01 (0.85, 1.19) | 20.7% (0.21) | 7 | 3906 | 0.98 (0.80, 1.20) | 25.1% (0.24) | 6 | 4042 | 0.78 (0.49, 1.22) | 32.6% (0.19) |
| Vitamin D2 | - | - | - | - | 1 | 126 | 0.54 (0.26, 1.10) | NA | - | - | - | - |
| **Trimester at supplementation initiation** |  |  |  |  |  |  |  |  |  |  |  |  |
| 1^st^ trimester | 4 | 1047 | 0.93 (0.52, 1.65) | 52.8% (0.10) | - | - | - | - | - | - | - | - |
| 2^nd^ trimester | 8 | 3675 | 1.03 (0.78, 1.35) | 0.0% (0.47) | 5 | 1424 | 0.78 (0.47, 1.29) | 45.3% (0.12) | 3 | 2803 | 0.69 (0.35, 1.35) | 61.0% (0.08) |
| 3^rd^ trimester | - | - | - | - | 1 | 126 | 0.54 (0.26, 1.10) | NA | - | - | - | - |
| **Maternal baseline 25(OH)D concentration <30 nmol/L**^c^ | |  |  |  |  |  |  |  |  |  |  |  |
| 25(OH)D <30 nmol/L | 5 | 2095 | 0.87 (0.68, 1.12) | 0.0% (0.62) | 4 | 1478 | 0.93 (0.64, 1.35) | 33.4% (0.21) | 2 | 1533 | 0.56 (0.19, 1.64) | 41.1% (0.19) |
| 25(OH)D ≥30 nmol/L | 8 | 5026 | 1.16 (1.01, 1.32) | 0.0% (0.47) | 4 | 2571 | 0.66 (0.33, 1.31) | 45.8% (0.14) | 4 | 2509 | 0.93 (0.61, 1.42) | 0.0% (0.61) |
| **Maternal baseline 25(OH)D concentration <50 nmol/L**^c^ | |  |  |  |  |  |  |  |  |  |  |  |
| 25(OH)D <50 nmol/L | 8 | 3371 | 0.90 (0.72, 1.12) | 0.0% (0.61) | 6 | 1752 | 0.78 (0.51, 1.20) | 46.5% (0.10) | 4 | 2582 | 0.82 (0.33, 2.00) | 48.5% (0.12) |
| 25(OH)D ≥50 nmol/L | 5 | 3750 | 1.17 (1.02, 1.34) | 0.0% (0.46) | 2 | 2297 | 0.77 (0.23, 2.61) | 26.9% (0.24) | 2 | 1460 | 0.84 (0.53, 1.34) | 0.0% (0.41) |

**Table S7. Subgroup analyses (continued)**

| **Subgroup** | **Admission to neonatal intensive care unit** | | | | **Gestational age (weeks)** | | | | **Birthweight (g)** | | | |
| --- | --- | --- | --- | --- | --- | --- | --- | --- | --- | --- | --- | --- |
|  | **Number of trials** | **Number of participants** | **Risk Ratio   (95% CI)** | **I^2^ %  (p-value)** | **Number of trials** | **Number of participants** | **Mean difference (95%CI)** | **I^2^ %  (p-value)** | **Number of trials** | **Number of participants** | **Mean difference (95%CI)** | **I^2^ %  (p-value)** |
| Primary analysis | 6 | 3236 | 1.02 (0.82, 1.27) | 0.0% (0.83) | 24 | 7305 | -0.07 (-0.19, 0.05) | 31.0% (0.08) | 40 | 9954 | 53.14 (16.48, 89.80) | 65.1% (<0.01) |
| **Intervention type** |  |  |  |  |  |  |  |  |  |  |  |  |
| Vitamin D alone *versus* placebo (or no intervention) | 3 | 1429 | 0.93 (0.68, 1.27) | 0.0% (0.64) | 13 | 5107 | -0.08 (-0.25, 0.09) | 47.0% (0.03) | 20 | 6180 | 42.93 (-5.50, 91.37) | 64.2% (<0.01) |
| Vitamin D + calcium and/or other vitamins and/or minerals *versus* calcium and/or other vitamins and/or minerals | - | - | - | - | 1 | 175 | -0.10 (-0.68, 0.48) | NA | 2 | 247 | 191.87 (-53.78, 437.53) | 88.2% (<0.01) |
| Vitamin D *versus* vitamin D of a lower dose (upto 600 IU/day)^a^ | 2 | 931 | 1.01 (0.68, 1.51) | 0.0% (0.86) | 8 | 1098 | -0.02 (-0.34, 0.30) | 33.1% (0.16) | 13 | 2136 | 29.44 (-20.06, 78.94) | 15.6% (0.29) |
| Vitamin D + calcium and/or other vitamins and/or minerals *versus* vitamin D of a lower dose (upto 600IU/day)^a^ + calcium and/or other vitamins and/or minerals | 1 | 876 | 1.27 (0.79, 2.05) | NA | 2 | 925 | -0.06 (-0.28, 0.16) | 0.0% (0.75) | 5 | 1391 | 30.90 (-45.52, 107.32) | 42.3% (0.14) |
| **Population type** |  |  |  |  |  |  |  |  |  |  |  |  |
| General population | 6 | 3236 | 1.02 (0.82, 1.27) | 0.0% (0.83) | 21 | 4965 | -0.05 (-0.18, 0.08) | 33.1% (0.07) | 33 | 6910 | 59.89 (15.42, 104.36) | 68.5% (<0.01) |
| Population with morbidities | - | - | - | - | 3 | 2340 | -0.26 (-0.50, -0.02) | 0.0% (0.66) | 7 | 3044 | 7.65 (-33.65, 48.96) | 6.8% (0.38) |
| **Intervention dose versus placebo pair^b^** |  |  |  |  |  |  |  |  |  |  |  |  |
| Intervention dose ≤600 IU/day | 1 pair | 336 | 0.95 (0.53, 1.69) | NA | 3 pairs | 494 | 0.26 (-0.15, 0.68) | 0.0% (0.82) | 3 pairs | 391 | 64.33 (-69.43, 198.09) | 49.9% (0.14) |
| Intervention dose >600 to ≤2000 IU/day | 1 pair | 178 | 1.06 (0.43, 2.62) | NA | 12 pairs | 1286 | -0.02 (-0.33, 0.30) | 68.2% (<0.01) | 21 pairs | 2896 | 47.43 (-19.83, 114.70) | 65.2% (<0.01) |
| Intervention dose >2000 IU/day | 7 pairs | 2722 | 1.03 (0.81, 1.32) | 0.0% (0.89) | 12 pairs | 5203 | -0.09 (-0.21, -0.03) | 0.0% (0.48) | 20 pairs | 5923 | 5.16 (-20.60, 20.93) | 0.0% (0.76) |
| **Administration frequency** |  |  |  |  |  |  |  |  |  |  |  |  |
| Regular | 6 | 3236 | 1.02 (0.82, 1.27) | 0.0% (0.83) | 21 | 6897 | -0.06 (-0.18, 0.06) | 20.7% (0.19) | 33 | 9092 | 36.35 (0.02, 72.68) | 58.5% (<0.01) |
| Bolus | - | - | - | - | 2 | 250 | 0.12 (-0.58, 0.81) | 68.9% (0.07) | 4 | 543 | 194.30 (128.82, 259.78) | 0.0% (0.40) |
| **Vitamin D supplementation form** |  |  |  |  |  |  |  |  |  |  |  |  |
| Vitamin D3 | 6 | 3236 | 1.02 (0.82, 1.27) | 0.0% (0.83) | 22 | 7021 | -0.04 (-0.16, 0.08) | 26.3% (0.13) | 35 | 9374 | 59.02 (20.30, 97.74) | 67.0% (<0.01) |
| Vitamin D2 | - | - | - | - | 1 | 126 | -0.20 (-0.75, 0.35) | NA | 2 | 203 | -26.97 (-323.89, 269.95) | 82.0% (0.02) |
| **Trimester at supplementation initiation** |  |  |  |  |  |  |  |  |  |  |  |  |
| 1^st^ trimester | 1 | 350 | 0.97 (0.50, 1.85) | NA | 2 | 514 | 0.10 (-0.38, 0.58) | 34.0% (0.22) | 5 | 1075 | -31.58 (-112.84, 49.67) | 21.5% (0.28) |
| 2^nd^ trimester | 3 | 1731 | 0.95 (0.73, 1.25) | 0.0% (0.62) | 12 | 2659 | 0.05 (-0.14, 0.24) | 31.4% (0.14) | 18 | 3629 | 100.44 (36.50, 164.38) | 74.0% (<0.01) |
| 3^rd^ trimester | - | - | - | - | 3 | 339 | -0.26 (-0.59, 0.07) | 0.0% (0.80) | 4 | 416 | 39.11 (-155.66, 233.89) | 76.2% (<0.01) |
| **Maternal baseline 25(OH)D concentration <30 nmol/L**^c^ | |  |  |  |  |  |  |  |  |  |  |  |
| 25(OH)D <30 nmol/L | 2 | 1284 | 0.96 (0.70, 1.33) | 0.0% (0.78) | 7 | 2318 | -0.17 (-0.38, 0.05) | 43.6% (0.10) | 10 | 2383 | 3.83 (-34.95, 42.62) | 0.0% (0.82) |
| 25(OH)D ≥30 nmol/L | 3 | 1602 | 1.10 (0.79, 1.54) | 0.0% (0.43) | 12 | 4502 | -0.04 (-0.21, -0.14) | 32.1% (0.13) | 23 | 6602 | 71.09 (20.47, 121.70) | 70.0% (<0.01) |
| **Maternal baseline 25(OH)D concentration <50 nmol/L**^c^ | |  |  |  |  |  |  |  |  |  |  |  |
| 25(OH)D <50 nmol/L | 3 | 1429 | 0.93 (0.68, 1.27) | 0.0% (0.64) | 12 | 2900 | -0.15 (-0.30, 0.00) | 25.2% (0.20) | 23 | 4837 | 44.44 (-5.96, 94.84) | 68.1% (<0.01) |
| 25(OH)D ≥50 nmol/L | 2 | 1457 | 1.16 (0.82, 1.63) | 0.0% (0.57) | 7 | 3920 | 0.06 (-0.29, 0.32) | 48.8% (0.07) | 10 | 4148 | 46.46 (-3.43, 96.34) | 28.6% (0.18) |

**Table S7. Subgroup analyses (continued)**

| **Subgroup** | **Birth body length (cm)** | | | | **Birth head circumference (cm)** | | | | **Cord 25(OH)D concentration (nmol/L)** | | | |
| --- | --- | --- | --- | --- | --- | --- | --- | --- | --- | --- | --- | --- |
|  | **Number of trials** | **Number of participants** | **Mean difference (95%CI)** | **I^2^ %  (p-value)** | **Number of trials** | **Number of participants** | **Mean difference (95%CI)** | **I^2^ %  (p-value)** | **Number of trials** | **Number of participants** | **Mean difference (95%CI)** | **I^2^ %  (p-value)** |
| Primary analysis | 22 | 5261 | 0.24 (0.01, 0.47) | 62.0% (<0.01) | 20 | 5159 | 0.17 (0.02, 0.32) | 63.2% (<0.01) | 26 | 4060 | 29.16 (21.87, 36.45) | 97.6% (<0.01) |
| **Intervention type** |  |  |  |  |  |  |  |  |  |  |  |  |
| Vitamin D alone *versus* placebo (or no intervention) | 10 | 2957 | 0.36 (-0.01, 0.74) | 77.4% (<0.01) | 9 | 2908 | 0.13 (0.10, 0.36) | 76.5% (<0.01) | 13 | 1820 | 38.30 (25.93, 50.68) | 98.7% (<0.01) |
| Vitamin D + calcium and/or other vitamins and/or minerals *versus* calcium and/or other vitamins and/or minerals | 22 | 247 | 0.16 (-0.29, 0.62) | 0.0% (0.84) | 2 | 247 | 0.00 (-0.37, 0.37) | 0.0% (1.00) | - | - | - | - |
| Vitamin D *versus* vitamin D of a lower dose (upto 600 IU/day)^a^ | 6 | 1011 | 0.11 (-0.37, 0.60) | 34.5% (0.18) | 6 | 1014 | 0.27 (-0.03, 0.57) | 22.3% (0.27) | 8 | 1311 | 18.62 (12.59, 24.65) | 77.8% (<0.01) |
| Vitamin D + calcium and/or other vitamins and/or minerals *versus* vitamin D of a lower dose (upto 600IU/day)^a^ + calcium and/or other vitamins and/or minerals | 4 | 1046 | 0.10 (-0.46, 0.65) | 58.4% (0.07) | 3 | 990 | 0.24 (-0.18, 0.66) | 74.1% (0.02) | 3 | 929 | 23.32 (11.00, 35.64) | 93.8% (<0.01) |
| **Population type** |  |  |  |  |  |  |  |  |  |  |  |  |
| General population | 20 | 5048 | 0.25 (0.00, 0.49) | 62.5% (<0.01) | 18 | 4946 | 0.16 (-0.00, 0.33) | 66.2% (<0.01) | 23 | 3794 | 28.50 (20.83, 36.17) | 97.7% (<0.01) |
| Population with morbidities | 2 | 213 | 0.19 (-0.94, 1.32) | 78.1% (0.03) | 2 | 213 | 0.26 (-0.16, 0.69) | 0.0% (0.37) | 3 | 266 | 33.67 (8.18, 59.15) | 96.4% (<0.01) |
| **Intervention dose versus placebo pair^b^** |  |  |  |  |  |  |  |  |  |  |  |  |
| Intervention dose ≤600 IU/day | 1 pair | 210 | 0.10 (-0.60, 0.80) | NA | 1 pair | 210 | 0.00 (-0.44, 0.44) | NA | 2 pairs | 174 | 15.58 (-4.38, 35.55) | 93.7% (<0.01) |
| Intervention dose >600 to ≤2000 IU/day | 10 pairs | 1789 | -0.05 (-0.26, 0.16) | 0.0% (0.90) | 9 pairs | 1769 | 0.02 (-0.20, 0.25) | 42.0% (0.09) | 20 pairs | 1361 | 21.10 (15.09, 27.12) | 90.2% (<0.01) |
| Intervention dose >2000 IU/day | 13 pairs | 2908 | 0.12 (-0.09, 0.33) | 15.7% (0.29) | 11 pairs | 2826 | 0.07 (-0.07, 0.21) | 22.3% (0.23) | 12 pairs | 2145 | 37.02 (27.11, 46.94) | 96.4% (<0.01) |
| **Administration frequency** |  |  |  |  |  |  |  |  |  |  |  |  |
| Regular | 19 | 4768 | 0.06 (-0.09, 0.21) | 7.7% (0.36) | 17 | 4666 | 0.06 (-0.06, 0.19) | 35.6% (0.05) | 22 | 3555 | 30.13 (22.43, 37.83) | 96.3% (<0.01) |
| Bolus | 3 | 493 | 1.02 (0.36, 1.68) | 70.2% (0.03) | 3 | 493 | 0.67 (0.43, 0.91) | 3.6% (0.35) | 1 | 165 | 13.69 (-9.24, 36.62) | NA |
| **Vitamin D supplementation form** |  |  |  |  |  |  |  |  |  |  |  |  |
| Vitamin D3 | 20 | 5007 | 0.22 (-0.03, 0.46) | 63.9% (<0.01) | 18 | 4905 | 0.16 (-0.01, 0.32) | 66.1% (<0.01) | 22 | 3635 | 27.71 (20.31, 35.11) | 96.2% (<0.01) |
| Vitamin D2 | 1 | 126 | 0.20 (-0.78, 1.18) | NA | 1 | 126 | 0.20 (-0.24, 0.64) | NA | 2 | 203 | 69.24 (-44.33, 182.80) | 99.1% (<0.01) |
| **Trimester at supplementation initiation** |  |  |  |  |  |  |  |  |  |  |  |  |
| 1^st^ trimester | - | - | - | - | - | - | - | - | 3 | 420 | 20.16 (13.16, 27.27) | 39.0% (0.19) |
| 2^nd^ trimester | 11 | 2793 | 0.15 (-0.09, 0.38) | 41.6% (0.07) | 9 | 2692 | 0.17 (-0.06, 0.41) | 72.3% (<0.01) | 10 | 1617 | 34.63 (22.15, 47.12) | 97.7% (<0.01) |
| 3^rd^ trimester | 3 | 339 | 0.82 (-0.39, 2.03) | 71.9% (0.03) | 3 | 339 | 0.45 (0.18, 0.71) | 6.5% (0.34) | 3 | 216 | 49.90 (21.57, 78.23) | 98.3% (<0.01) |
| **Maternal baseline 25(OH)D concentration <30 nmol/L**^c^ | |  |  |  |  |  |  |  |  |  |  |  |
| 25(OH)D <30 nmol/L | 5 | 1562 | 0.04 (-0.22, 0.29) | 0.0% (0.47) | 5 | 1571 | 0.03 (-0.12, 0.18) | 0.0% (0.95) | 4 | 1036 | 47.10 (20.35, 73.85) | 99.3% (<0.01) |
| 25(OH)D ≥30 nmol/L | 14 | 3402 | 0.19 (-0.04, 0.42) | 40.4% (0.06) | 12 | 3291 | 0.20 (-0.03, 0.44) | 72.5% (<0.01) | 13 | 2027 | 31.09 (20.22, 41.97) | 95.7% (<0.01) |
| **Maternal baseline 25(OH)D concentration <50 nmol/L**^c^ | |  |  |  |  |  |  |  |  |  |  |  |
| 25(OH)D <50 nmol/L | 15 | 3822 | 0.07 (-0.11, 0.25) | 19.1% (0.24) | 14 | 3774 | 0.11 (-0.06, 0.28) | 60.5% (<0.01) | 11 | 2052 | 42.49 (29.25, 55.73) | 98.1% (<0.01) |
| 25(OH)D ≥50 nmol/L | 4 | 1142 | 0.42 (-0.08, 0.91) | 50.2% (0.11) | 3 | 1088 | 0.45 (-0.19, 1.10) | 74.3% (0.02) | 6 | 1011 | 16.95 (8.39, 25.51) | 84.6% (<0.01) |

**Table S7. Subgroup analyses (continued)**

| **Subgroup** | **Neonatal death** | | | | **Lower respiratory tract infection** | | | | **Body weight at 1 year old (cm)** | | | |
| --- | --- | --- | --- | --- | --- | --- | --- | --- | --- | --- | --- | --- |
|  | **Number of trials** | **Number of participants** | **Risk ratio (95%CI)** | **I^2^ %  (p-value)** | **Number of trials** | **Number of participants** | **Risk ratio (95%CI)** | **I^2^ %  (p-value)** | **Number of trials** | **Number of participants** | **Mean difference (95%CI)** | **I^2^ %  (p-value)** |
| Primary analysis | 5 | 4374 | 1.14 (0.70, 1.86) | 4.3% (0.38) | 5 | 2080 | 0.98 (0.86, 1.11) | 0.0% (0.78) | 4 | 2707 | 61.06 (-174.11, 296.23) | 80.8% (<0.01) |
| **Intervention type** |  |  |  |  |  |  |  |  |  |  |  |  |
| Vitamin D alone *versus* placebo (or no intervention) | 4 | 3498 | 0.96 (0.46, 2.01) | 27.3% (0.25) | 2 | 387 | 1.14 (0.78, 1.66) | 0.0% (0.39) | 4 | 2707 | 61.06 (-174.11, 296.23) | 80.8% (<0.01) |
| Vitamin D + calcium and/or other vitamins and/or minerals *versus* calcium and/or other vitamins and/or minerals | - | - | - | - | - | - | - | - | - | - | - | - |
| Vitamin D *versus* vitamin D of a lower dose (upto 600 IU/day)^a^ | - | - | - | - | 1 | 576 | 0.96 (0.76, 1.22) | NA | - | - | - | - |
| Vitamin D + calcium and/or other vitamins and/or minerals *versus* vitamin D of a lower dose (upto 600IU/day)^a^ + calcium and/or other vitamins and/or minerals | 1 | 876 | 0.99 (0.20, 4.88) | 0.0% (NA) | 2 | 1117 | 0.96 (0.81, 1.14) | 0.0% (0.55) | - | - | - | - |
| **Population type** |  |  |  |  |  |  |  |  |  |  |  |  |
| General population | 4 | 2207 | 0.64 (0.28, 1.49) | 0.0% (0.72) | 5 | 2080 | 0.98 (0.86, 1.11) | 0.0% (0.78) | 3 | 1182 | 132.03 (-217.77, 481.83) | 83.1% (<0.01) |
| Population with morbidities | 1 | 2167 | 1.50 (0.89. 2.54) | NA | - | - | - | - | 1 | 1525 | -100.00 (-231.00, 31.00) | NA |
| **Intervention dose versus placebo pair^b^** |  |  |  |  |  |  |  |  |  |  |  |  |
| Intervention dose ≤600 IU/day | 1 pair | 336 | 0.49 (0.06, 4.31) | NA | - | - | - | - | 1 pair | 312 | -138.00 (-440.87, 164.87) | NA |
| Intervention dose >600 to ≤2000 IU/day | 1 pair | 90 | 0.17 (0.01, 3.94) | NA | 4 pairs | 466 | 1.11 (0.82, 1.51) | 0.0% (0.92) | 1 pair | 117 | 410.00 (176.24, 643.76) | NA |
| Intervention dose >2000 IU/day | 5 pairs | 3859 | 1.28 (0.81, 2.03) | 0.0% (0.63) | 3 pairs | 1541 | 0.94 (0.81, 1.09) | 0.0% (0.96) | 4 pairs | 2278 | -79.90 (-186.98, 27.18) | 0.0% (0.77) |
| **Administration frequency** |  |  |  |  |  |  |  |  |  |  |  |  |
| Regular | 4 | 4195 | 1.23 (0.79, 1.93) | 0.0% (0.45) | 4 | 1929 | 0.96 (0.85, 1.10) | 0.0% (0.94) | 4 | 2707 | 61.06 (-174.11, 296.23) | 80.8% (<0.01) |
| Bolus | - | - | - | - | - | - | - | - | - | - | - | - |
| **Vitamin D supplementation form** |  |  |  |  |  |  |  |  |  |  |  |  |
| Vitamin D3 | 4 | 4195 | 1.23 (0.79, 1.93) | 0.0% (0.45) | 4 | 1929 | 0.96 (0.85, 1.10) | 0.0% (0.94) | 3 | 2590 | -86.36 (-187.38, 14.65) | 0.0% (0.60) |
| Vitamin D2 | - | - | - | - | - | - | - | - | 1 | 117 | 410.00 (176.24, 643.76) | NA |
| **Trimester at supplementation initiation** |  |  |  |  |  |  |  |  |  |  |  |  |
| 1^st^ trimester | - | - | - | - | - | - | - | - | - | - | - | - |
| 2^nd^ trimester | 3 | 1331 | 0.55 (0.20, 1.47) | 0.0% (0.62) | 3 | 963 | 1.01 (0.83, 1.23) | 0.0% (0.53) | 2 | 1065 | -66.36 (-225.03, 92.30) | 0.0% (0.33) |
| 3^rd^ trimester | - | - | - | - | - | - | - | - | 1 | 117 | 410.00 (176.24, 643.76) | NA |
| **Maternal baseline 25(OH)D concentration <30 nmol/L**^c^ | |  |  |  |  |  |  |  |  |  |  |  |
| 25(OH)D <30 nmol/L | 2 | 1184 | 0.61 (0.20, 1.84) | 0.0% (0.39) | 2 | 462 | 1.11 (0.84, 1.48) | 0.0% (0.40) | 2 | 1047 | 147.43 (-355.16, 650.02) | 91.6% (<0.01) |
| 25(OH)D ≥30 nmol/L | 3 | 3190 | 1.35 (0.83, 2.19) | 0.0% (0.41) | 3 | 1618 | 0.95 (0.82, 1.10) | 0.0% (0.96) | 2 | 1660 | -78.09 (-201.71, 45.53) | 0.0% (0.32) |
| **Maternal baseline 25(OH)D concentration <50 nmol/L**^c^ | |  |  |  |  |  |  |  |  |  |  |  |
| 25(OH)D <50 nmol/L | 3 | 1331 | 0.55 (0.20, 1.47) | 0.0% (0.62) | 2 | 462 | 1.11 (0.84, 1.48) | 0.0% (0.40) | 3 | 1182 | 132.03 (-217.77, 481.83) | 83.1% (<0.01) |
| 25(OH)D ≥50 nmol/L | 2 | 3043 | 1.44 (0.88, 2.37) | 0.0% (0.63) | 3 | 1618 | 0.95 (0.82, 1.10) | 0.0% (0.96) | 1 | 1525 | -100.00 (-231.00, 31.00) | NA |

**Table S7. Subgroup analyses (continued)**

| **Subgroup** | **Body length at 1 year old** | | | | **Weight for age z score at 1 year old** | | | | **Length for age z score at 1 year old** | | | |
| --- | --- | --- | --- | --- | --- | --- | --- | --- | --- | --- | --- | --- |
|  | **Number of trials** | **Number of participants** | **Mean difference (95%CI)** | **I^2^ %  (p-value)** | **Number of trials** | **Number of participants** | **Mean difference (95%CI)** | **I^2^ %  (p-value)** | **Number of trials** | **Number of participants** | **Mean difference (95%CI)** | **I^2^ %  (p-value)** |
| Primary analysis | 4 | 2923 | 0.42 (-0.40, 1.24) | 89.5% (<0.01) | 4 | 2642 | -0.09 (-0.18, 0.00) | 0.0% (0.42) | 4 | 2858 | 0.01 (-0.18, 0.17) | 52.8% (0.10) |
| **Intervention type** |  |  |  |  |  |  |  |  |  |  |  |  |
| Vitamin D alone *versus* placebo (or no intervention) | 4 | 2923 | 0.42 (-0.40, 1.24) | 89.5% (<0.01) | 3 | 2590 | -0.08 (-0.20, 0.04) | 28.8% (0.25) | 3 | 2806 | 0.01 (-0.20, 0.21) | 68.5% (0.04) |
| Vitamin D + calcium and/or other vitamins and/or minerals *versus* calcium and/or other vitamins and/or minerals | - | - | - | - | - | - | - | - | - | - | - | - |
| Vitamin D *versus* vitamin D of a lower dose (upto 600 IU/day)^a^ | - | - | - | - | - | - | - | - | - | - | - | - |
| Vitamin D + calcium and/or other vitamins and/or minerals *versus* vitamin D of a lower dose (upto 600IU/day)^a^ + calcium and/or other vitamins and/or minerals | - | - | - | - | 1 | 52 | -0.15 (-0.89, 0.59) | NA | 1 | 52 | -0.04 (-0.73, 0.65) | NA |
| **Population type** |  |  |  |  |  |  |  |  |  |  |  |  |
| General population | 3 | 1184 | 0.71 (-0.53, 1.96) | 90.6% (<0.01) | 3 | 1117 | -0.04 (-0.26, 0.18) | 27.5% (0.25) | 3 | 1119 | 0.08 (-0.31, 0.48) | 68.5% (0.04) |
| Population with morbidities | 1 | 1739 | -0.30 (-0.65, 0.05) | NA | 1 | 1525 | -0.10 (-0.22, 0.02) | NA | 1 | 1739 | -0.05 (-0.19, 0.09) | NA |
| **Intervention dose versus placebo pair^b^** |  |  |  |  |  |  |  |  |  |  |  |  |
| Intervention dose ≤600 IU/day | 1 pair | 313 | -0.31 (-1.03, 0.41) | NA | 1 pair | 312 | -0.19 (-0.48, 0.10) | NA | 1 pair | 313 | -0.18 (-0.45, 0.09) | NA |
| Intervention dose >600 to ≤2000 IU/day | 1 pair | 117 | 1.60 (0.94, 2.26) | NA | 1 pair | 31 | -0.07 (-1.12, 0.98) | NA | 1 pair | 31 | 0.10 (-0.85, 1.05) | NA |
| Intervention dose >2000 IU/day | 4 pairs | 2493 | -0.11 (-0.48, 0.25) | 27.8% (0.25) | 5 pairs | 2299 | -0.08 (-0.18, 0.02) | 0.0% (0.58) | 5 pairs | 2514 | -0.01 (-0.17, 0.14) | 33.2% (0.20) |
| **Administration frequency** |  |  |  |  |  |  |  |  |  |  |  |  |
| Regular | 4 | 2923 | 0.42 (-0.40, 1.24) | 89.5% (<0.01) | 4 | 2642 | -0.09 (-0.18, 0.00) | 0.0% (0.42) | 4 | 2858 | 0.01 (-0.18, 0.17) | 52.8% (0.10) |
| Bolus | - | - | - | - | - | - | - | - | - | - | - | - |
| **Vitamin D supplementation form** |  |  |  |  |  |  |  |  |  |  |  |  |
| Vitamin D3 | 3 | 2806 | -0.11 (-0.53, 0.30) | 50.4% (0.13) | 4 | 2642 | -0.09 (-0.18, 0.00) | 0.0% (0.42) | 4 | 2858 | 0.01 (-0.18, 0.17) | 52.8% (0.10) |
| Vitamin D2 | 1 | 117 | 1.60 (0.94, 2.26) | NA | - | - | - | - | - | - | - | - |
| **Trimester at supplementation initiation** |  |  |  |  |  |  |  |  |  |  |  |  |
| 1^st^ trimester | - | - | - | - | - | - | - | - | - | - | - | - |
| 2^nd^ trimester | 2 | 1067 | 0.19 (-0.77, 1.14) | 68.7% (0.07) | 3 | 1117 | -0.04 (-0.26, 0.18) | 27.5% (0.25) | 3 | 1119 | 0.08 (-0.31, 0.48) | 68.5% (0.04) |
| 3^rd^ trimester | 1 | 117 | 1.60 (0.94, 2.26) | NA | - | - | - | - | - | - | - | - |
| **Maternal baseline 25(OH)D concentration <30 nmol/L**^c^ | |  |  |  |  |  |  |  |  |  |  |  |
| 25(OH)D <30 nmol/L | 2 | 1050 | 0.68 (-1.08, 2.44) | 95.1% (<0.01) | 1 | 930 | -0.13 (-0.30, 0.04) | NA | 1 | 933 | -0.12 (-0.28, 0.04) | NA |
| 25(OH)D ≥30 nmol/L | 2 | 1873 | 0.14 (-0.91, 1.20) | 75.2% (0.04) | 2 | 1660 | 0.00 (-0.28, 0.28) | 59.5% (0.12) | 2 | 1873 | 0.16 (-0.32, 0.63) | 80.1% (0.03) |
| **Maternal baseline 25(OH)D concentration <50 nmol/L**^c^ | |  |  |  |  |  |  |  |  |  |  |  |
| 25(OH)D <50 nmol/L | 3 | 1184 | 0.71 (-0.53, 1.96) | 90.6 (<0.01) | 2 | 1065 | -0.00 (-0.32, 0.31) | 63.2% (0.10) | 2 | 1067 | 0.13 (-0.42, 0.67) | 84.3% (0.01) |
| 25(OH)D ≥50 nmol/L | 1 | 1739 | -0.30 (-0.65, 0.05) | NA | 1 | 1525 | -0.10 (-0.22, 0.02) | NA | 1 | 1739 | -0.05 (-0.19, 0.09) | NA |

**Figure S1. Risk of bias assessment for included trials**


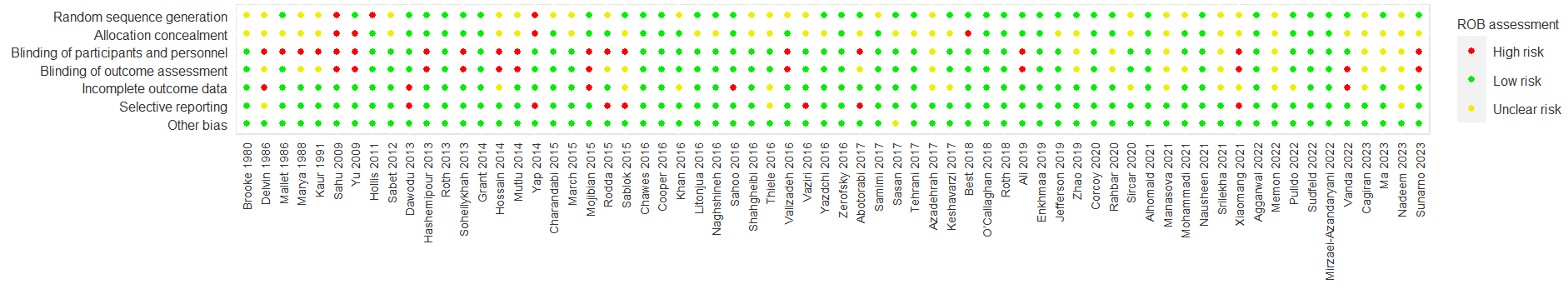


**Figure S2. Funnel plots and Egger’s test (for outcomes with at least 10 trials)**

**Figure S2.1 Funnel plot for gestational diabetes**

**
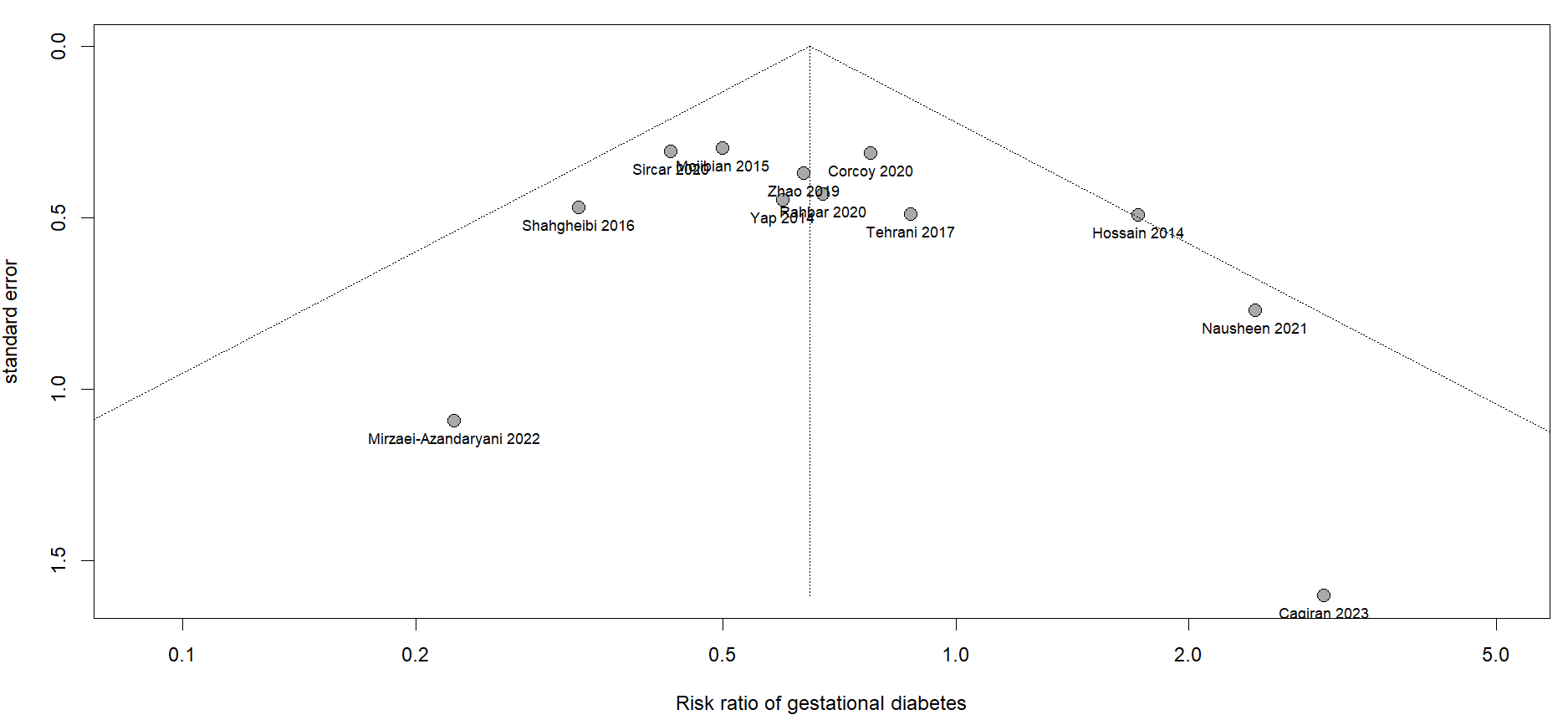
**

Egger’s test p-value = 0.245

**Figure S2.2 Funnel plot for Cesarean delivery**

**
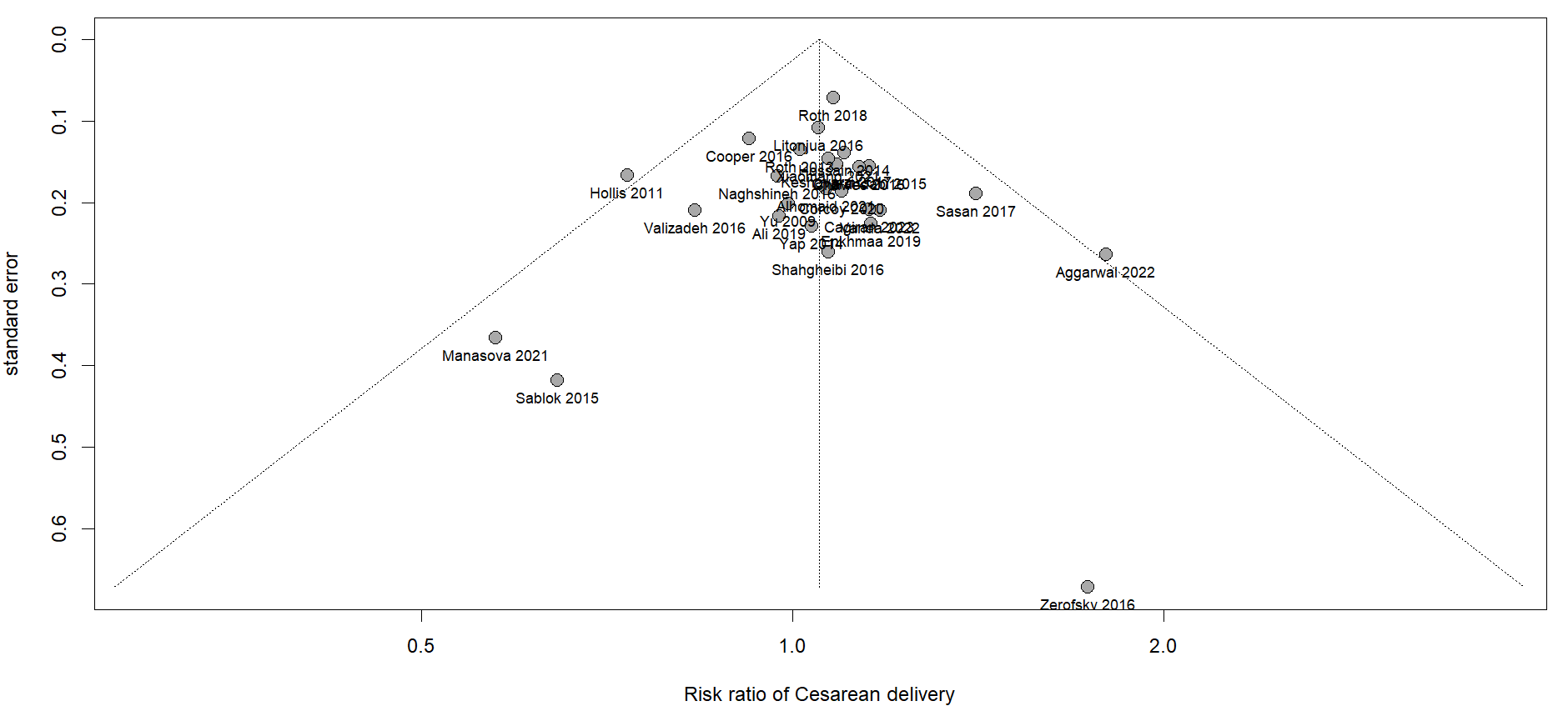
**

Egger’s test p-value = 0.855

**Figure S2.3 Funnel plot for stillbirth or intrauterine death**

**
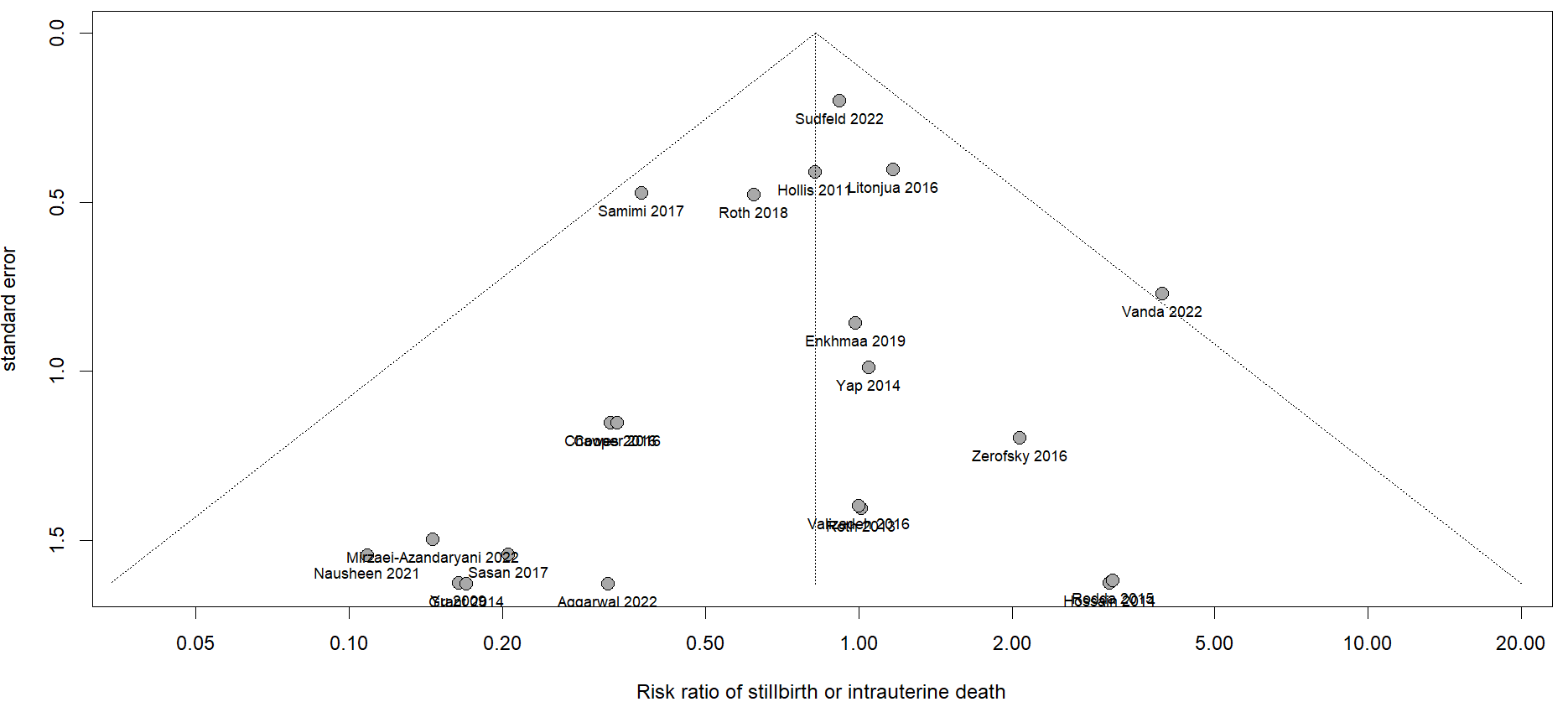
**

Egger’s test p-value = 0.262

**Figure S2.4 Funnel plot for low birthweight infant**

**
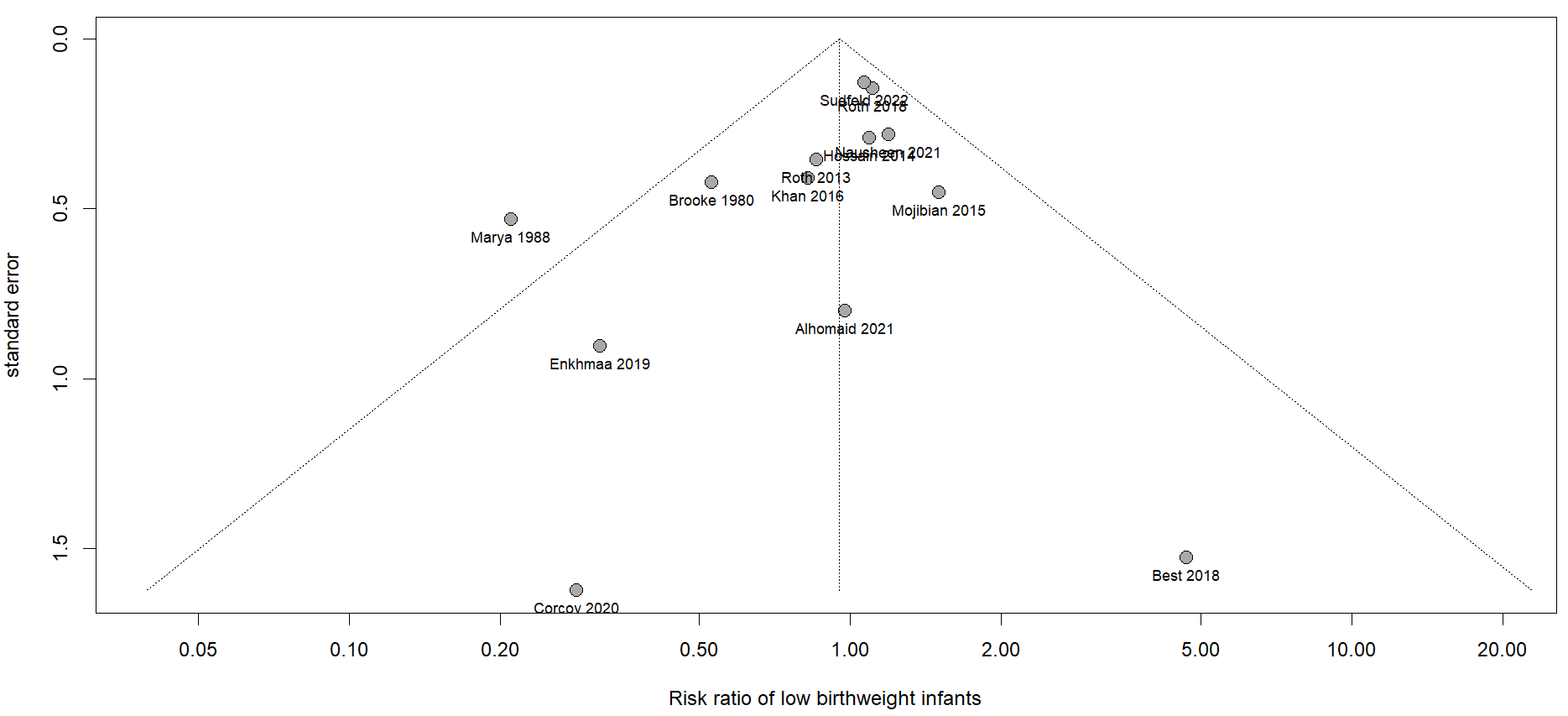
**

Egger’s test p-value = 0.155

**Figure S2.5 Funnel plot for preterm birth**


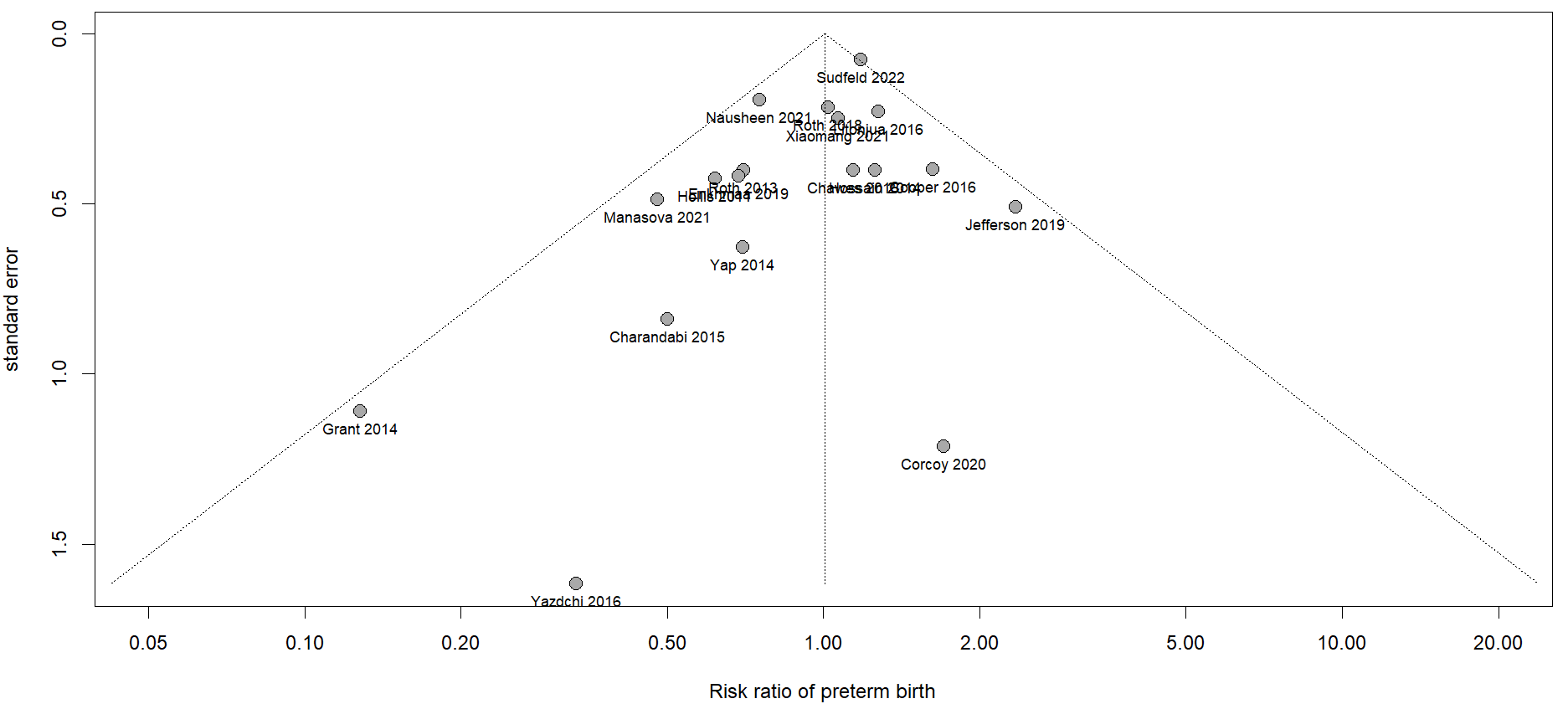


Egger’s test p-value = 0.058

**Figure S2.6 Funnel plot for maternal 25(OH)D concentration at or near delivery (nmol/L)**

**
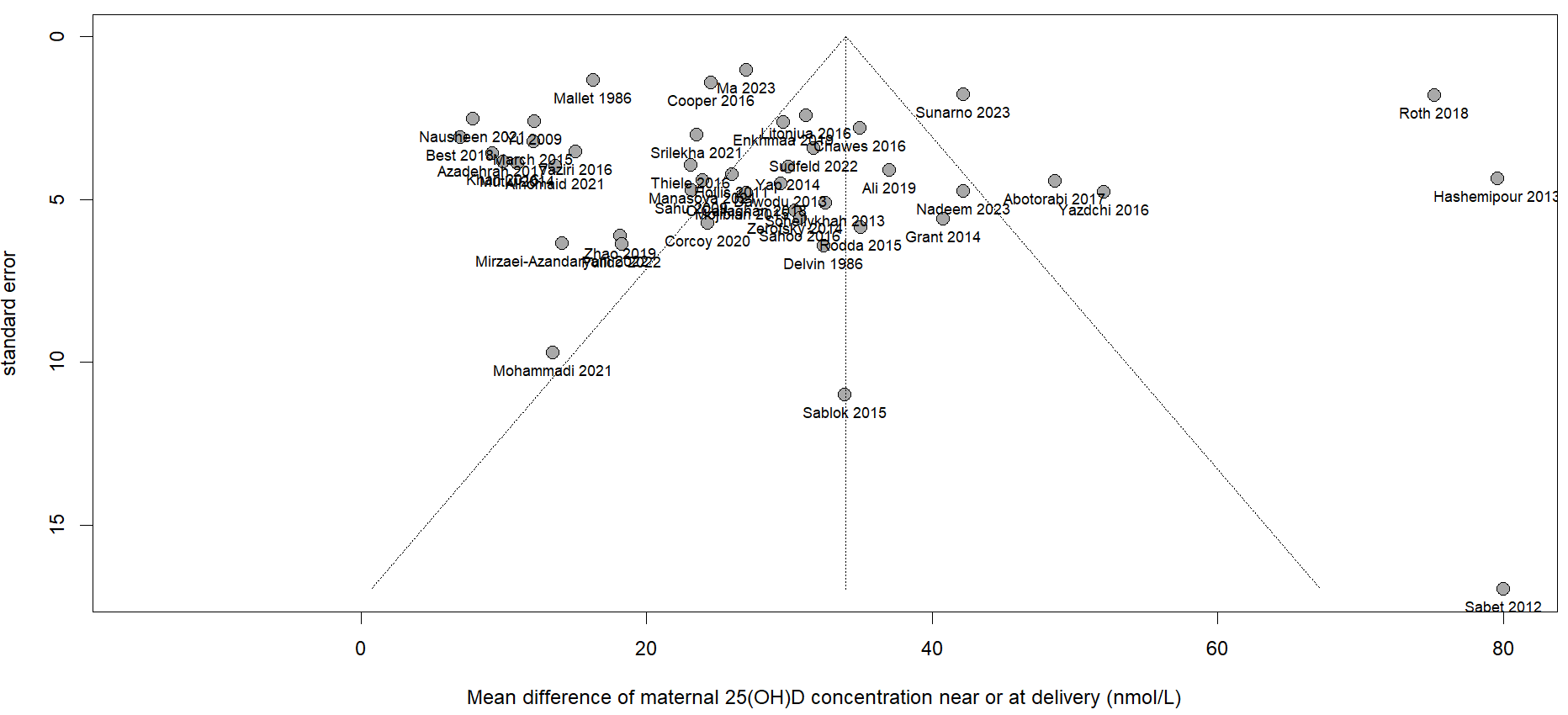
**

Egger’s test p-value = 0.337

**Figure S2.7 Funnel plot for gestational age (weeks)**

**
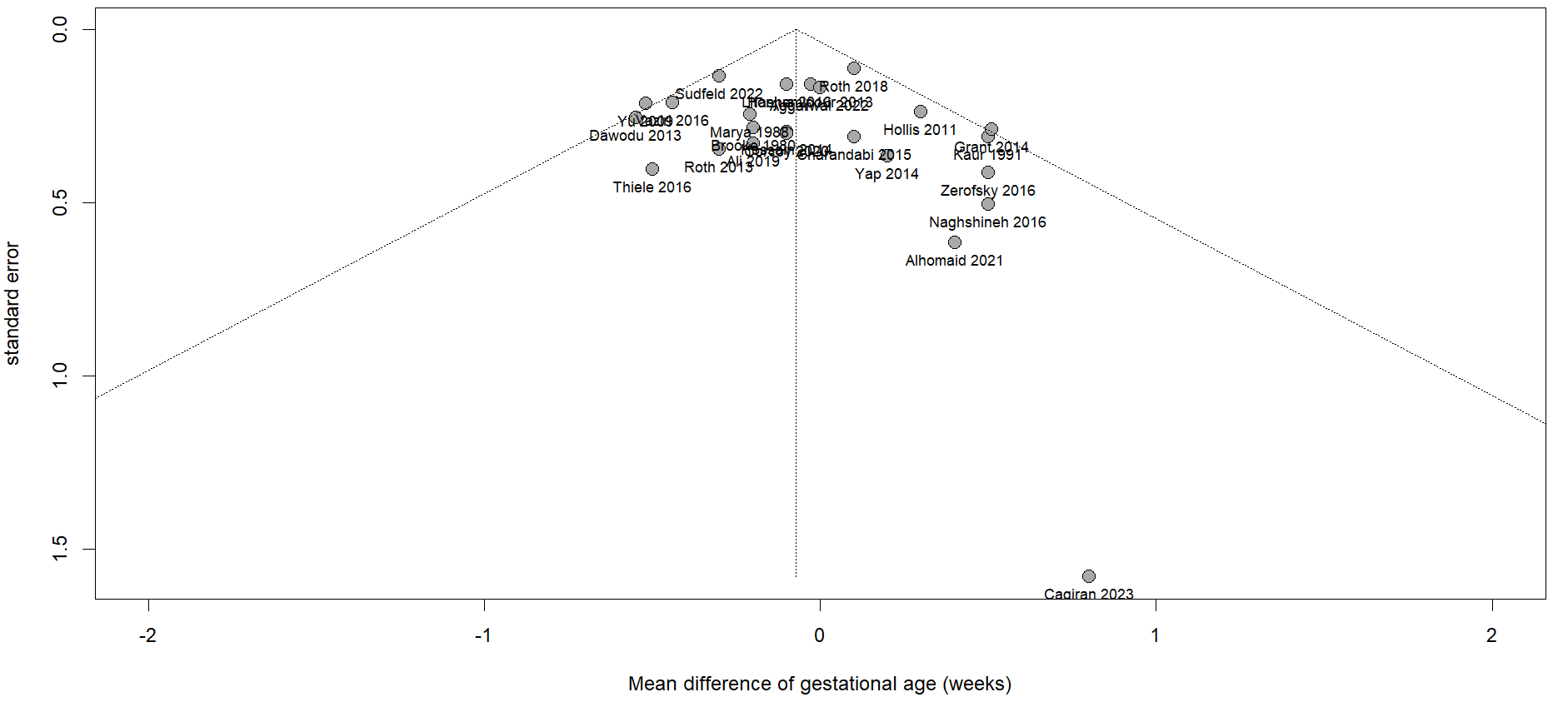
**

Egger’s test p-value = 0.417

**Figure S2.8 Funnel plot for birthweight (g)**

**
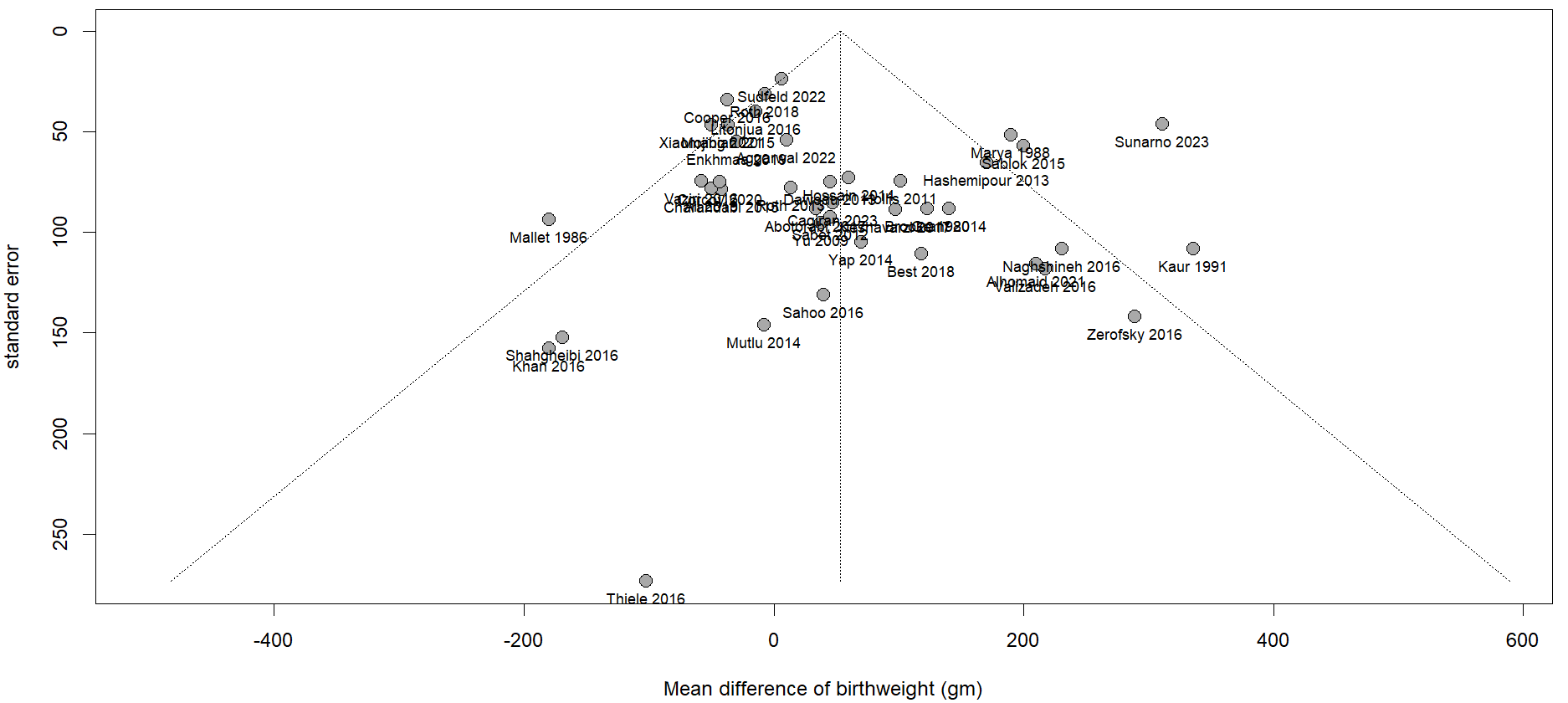
**

Egger’s test p-value = 0.197

**Figure S2.9 Funnel plot for birth body length (cm)**

**
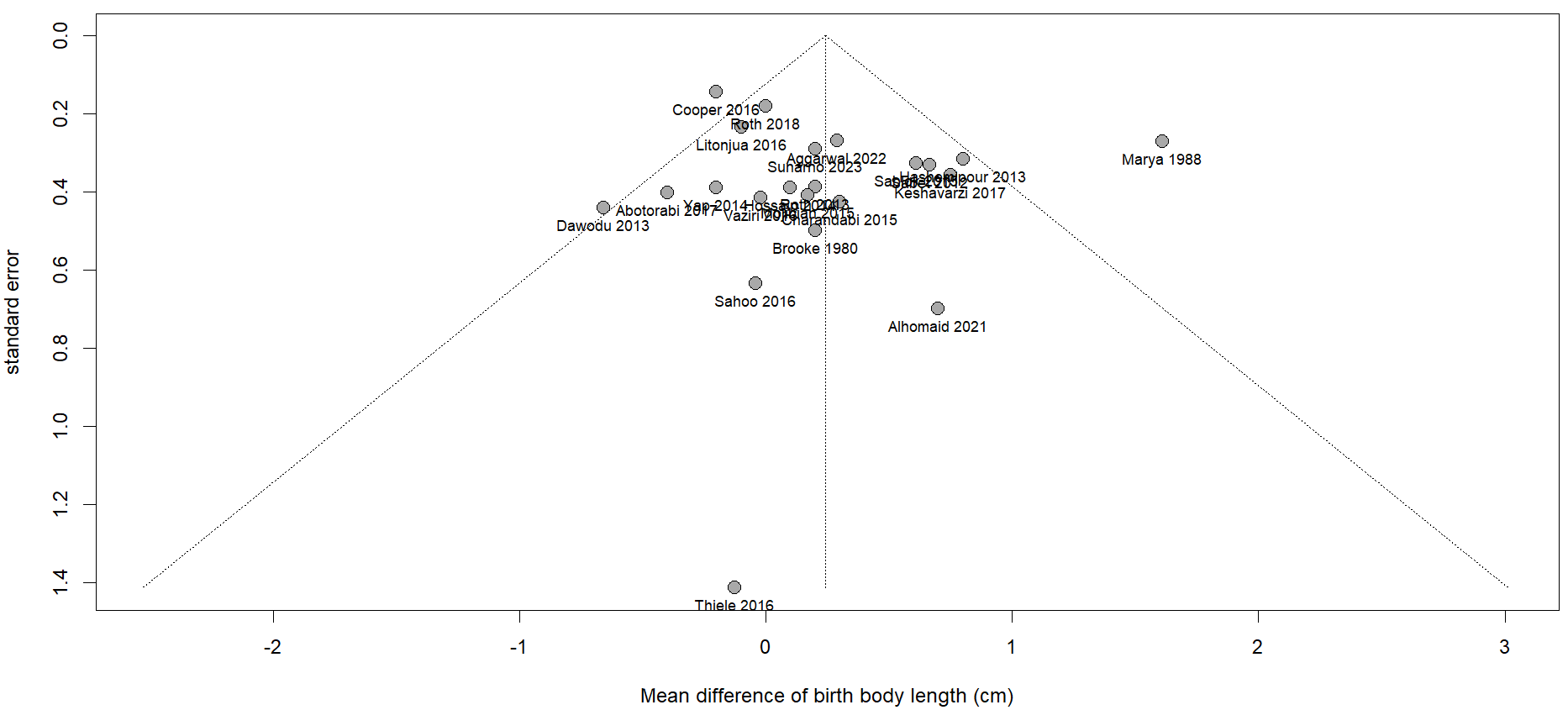
**

Egger’s test p-value = 0.409

**Figure S2.10 Funnel plot for birth head circumference (cm)**

**
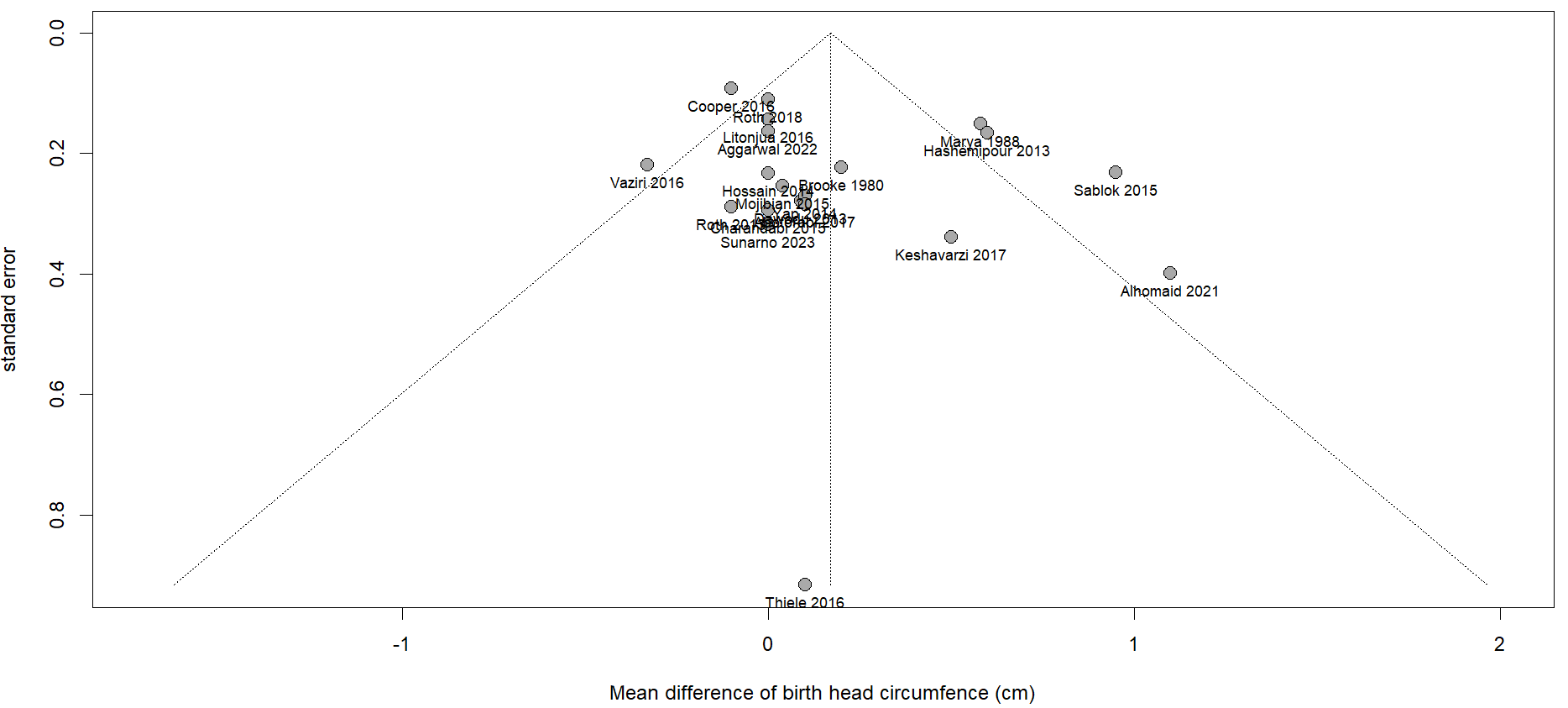
**

Egger’s test p-value = 0.233

**Figure S2.11 Funnel plot for cord 25(OH)D concentration** **(nmol/L)**

**
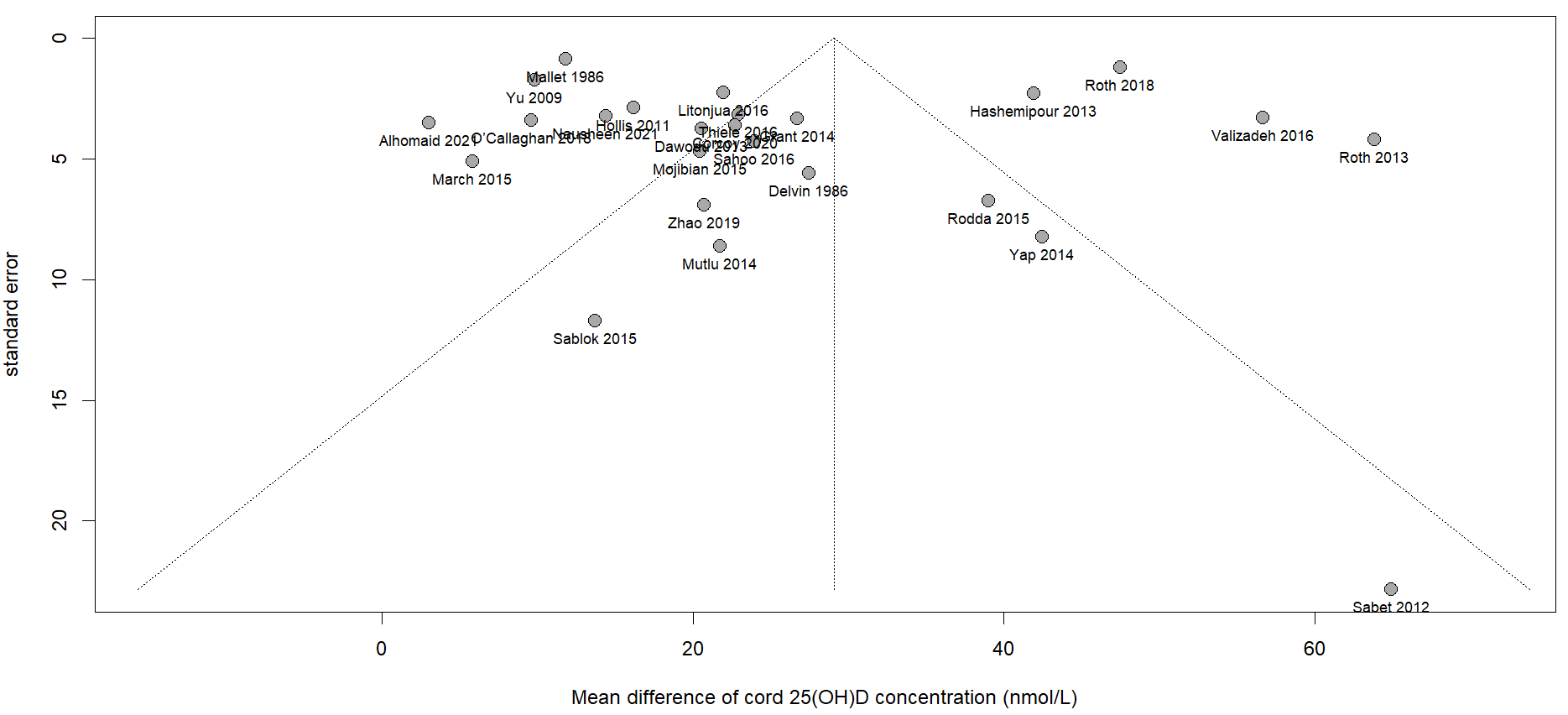
**

Egger’s test p-value = 0.356

**Table S8. Summary of findings for sensitivity analyses**

| **Outcome** | **Number of trials** | **Number of participants** | **Risk Ratio (95% CI)** | **I^2^ (p-value)** |
| --- | --- | --- | --- | --- |
| **Maternal outcomes** | | | | |
| Preeclampsia | 22 | 4981 | 0.65 (0.49, 0.86) | 14.8% (0.26) |
| Gestational hypertension | 12 | 4330 | 0.96 (0.67, 1.36) | 0.0% (0.62) |
| Gestational diabetes | 24 | 5938 | 0.66 (0.54, 0.80) | 3.5% (0.41) |
| Maternal hypercalcemia^a^ | 2 | 347 | 1.85 (0.84, 4.06) | 1.7% (0.31) |
| Maternal hypocalcemia^b^ | 3 | 662 | 0.13 (0.01, 1.61) | 90.8% (<0.01) |
| Maternal hypercalciuria^c^ | 4 | 1458 | 1.05 (0.60, 1.81) | 0.0% (0.69) |
| **Neonatal and infant outcomes** | | | | |
| Neonatal hypercalcemia^d^ | 3 | 892 | 1.23 (0.45, 3.38) | 0.0% (0.70) |
| Neonatal hypocalcemia^e^ | 2 | 235 | 0.14 (0.02, 1.12) | 0.0% (0.76) |
| Asthma or recurrent/persistent wheeze by 3 year old | 5 | 1833 | 0.77 (0.57, 1.05) | 36.3% (0.18) |

^a^ 13 trials reported maternal hypercalcemia; 11 trials, including Roth et al. (2013), Grant et al. (2014), Mutlu et al. (2014), Cooper et al. (2016), Litonjua et al. (2016), Sahoo et al. (2016), Valizadeh et al. (2016), Roth et al. (2018), Enkhmaa et al. (2019), Corcoy et al. (2020), and Sudfeld et al. (2022) have zero events in both arms.

^b^ 4 trials reported maternal hypocalcemia; Mutlu et al. (2014) has zero events in both arms.

^c^ 5 trials reported maternal hypercalciuria; Mutlu et al. (2014) has zero events in both arms.

^d^ 4 trials reported neonatal hypercalcemia; Grant et al. (2014) has zero events in both arms.

^e^ 4 trials reported neonatal hypocalcemia; Roth et al. (2013) and Sablok et al. (2015) have zero events in both arms.

**Figure S3. Forest plots of primary analyses, sensitivity analyses, and subgroup analyses**

***Maternal outcomes***

**1. Preeclampsia**

Figure S3.1.1 Primary analysis

Figure S3.1.2 Sensitivity analysis

Figure S3.1.3 Subgroup analysis by intervention type

Figure S3.1.4 Subgroup analysis by population type

Figure S3.1.5 Subgroup analysis by intervention dose

Figure S3.1.6 Subgroup analysis by administration frequency

Figure S3.1.7 Subgroup analysis by supplement form

Figure S3.1.8 Subgroup analysis by trimester of supplementation initiation

Figure S3.1.9 Subgroup analysis by maternal population mean 25(OH)D concentration (30 nmol/L)

Figure S3.1.10 Subgroup analysis by maternal population mean 25(OH)D concentration (50 nmol/L)

**2. Gestational hypertension**

Figure S3.2.1 Primary analysis

Figure S3.2.2 Sensitivity analysis

Figure S3.2.3 Subgroup analysis by intervention type

Figure S3.2.4 Subgroup analysis by population type

Figure S3.2.5 Subgroup analysis by intervention dose

Figure S3.2.6 Subgroup analysis by administration frequency

Figure S3.2.7 Subgroup analysis by supplement form

Figure S3.2.8 Subgroup analysis by trimester of supplementation initiation

Figure S3.2.9 Subgroup analysis by maternal population mean 25(OH)D concentration (30 nmol/L)

Figure S3.2.10 Subgroup analysis by maternal population mean 25(OH)D concentration (50 nmol/L)

**3. Gestational diabetes**

Figure S3.3.1 Primary analysis

Figure S3.3.2 Sensitivity analysis

Figure S3.3.3 Subgroup analysis by intervention type

Figure S3.3.4 Subgroup analysis by population type

Figure S3.3.5 Subgroup analysis by population health condition

Figure S3.3.6 Subgroup analysis by intervention dose

Figure S3.3.7 Subgroup analysis by administration frequency

Figure S3.3.8 Subgroup analysis by supplement form

Figure S3.3.9 Subgroup analysis by trimester of supplementation initiation

Figure S3.3.10 Subgroup analysis by maternal population mean 25(OH)D concentration (30 nmol/L)

Figure S3.3.11 Subgroup analysis by maternal population mean 25(OH)D concentration (50 nmol/L)

**4. Preterm labor**

Figure S3.4.1 Primary analysis

Figure S3.4.2 Subgroup analysis by intervention type

Figure S3.4.3 Subgroup analysis by population type

Figure S3.4.4 Subgroup analysis by intervention dose

Figure S3.4.5 Subgroup analysis by administration frequency

Figure S3.4.6 Subgroup analysis by supplement form

Figure S3.4.7 Subgroup analysis by trimester of supplementation initiation

Figure S3.4.8 Subgroup analysis by maternal population mean 25(OH)D concentration (30 nmol/L)

Figure S3.4.9 Subgroup analysis by maternal population mean 25(OH)D concentration (50 nmol/L)

**5. Cesarean delivery**

Figure S3.5.1 Primary analysis

Figure S3.5.2 Subgroup analysis by intervention type

Figure S3.5.3 Subgroup analysis by population type

Figure S3.5.4 Subgroup analysis by intervention dose

Figure S3.5.5 Subgroup analysis by administration frequency

Figure S3.5.6 Subgroup analysis by supplement form

Figure S3.5.7 Subgroup analysis by trimester of supplementation initiation

Figure S3.5.8 Subgroup analysis by maternal population mean 25(OH)D concentration (30 nmol/L)

Figure S3.5.9 Subgroup analysis by maternal population mean 25(OH)D concentration (50 nmol/L)

**6. Maternal hospitalization**

Figure S3.6.1 Primary analysis

Figure S3.6.2 Subgroup analysis by intervention type

Figure S3.6.3 Subgroup analysis by population type

Figure S3.6.4 Subgroup analysis by intervention dose

Figure S3.6.5 Subgroup analysis by administration frequency

Figure S3.6.6 Subgroup analysis by supplement form

Figure S3.6.7 Subgroup analysis by trimester of supplementation initiation

Figure S3.6.8 Subgroup analysis by maternal population mean 25(OH)D concentration (30 nmol/L)

Figure S3.6.9 Subgroup analysis by maternal population mean 25(OH)D concentration (50 nmol/L)

**7. Maternal hypercalcemia**

Figure S3.7.1 Primary analysis

Figure S3.7.2 Sensitivity analysis

**8. Maternal Hypocalcemia**

Figure S3.8.1 Primary analysis

Figure S3.8.2 Sensitivity analysis

**9. Maternal hypercalciuria**

Figure S3.9.1 Primary analysis

Figure S3.9.2 Sensitivity analysis

**10. Maternal 25(OH)D concentration at or near delivery (nmol/L)**

Figure S3.10.1 Primary analysis

Figure S3.10.2 Subgroup analysis by intervention type

Figure S3.10.3 Subgroup analysis by population type

Figure S3.10.4 Subgroup analysis by intervention dose

Figure S3.10.5 Subgroup analysis by administration frequency

Figure S3.10.6 Subgroup analysis by supplement form

Figure S3.10.7 Subgroup analysis by trimester of supplementation initiation

Figure S3.10.8 Subgroup analysis by maternal population mean 25(OH)D concentration (30 nmol/L)

Figure S3.10.9 Subgroup analysis by maternal population mean 25(OH)D concentration (50 nmol/L)

***Birth outcomes***

**11. Stillbirth or intrauterine death**

Figure S3.11.1 Primary analysis

Figure S3.11.2 Subgroup analysis by intervention type

Figure S3.11.3 Subgroup analysis by population type

Figure S3.11.4 Subgroup analysis by intervention dose

Figure S3.11.5 Subgroup analysis by administration frequency

Figure S3.11.6 Subgroup analysis by supplement form

Figure S3.11.7 Subgroup analysis by trimester of supplementation initiation

Figure S3.11.8 Subgroup analysis by maternal population mean 25(OH)D concentration (30 nmol/L)

Figure S3.11.9 Subgroup analysis by maternal population mean 25(OH)D concentration (50 nmol/L)

**12. Low birthweight infant (birthweight < 2500g)**

Figure S3.12.1 Primary analysis

Figure S3.12.2 Subgroup analysis by intervention type

Figure S3.12.3 Subgroup analysis by population type

Figure S3.12.4 Subgroup analysis by intervention dose

Figure S3.12.5 Subgroup analysis by administration frequency

Figure S3.12.6 Subgroup analysis by supplement form

Figure S3.12.7 Subgroup analysis by trimester of supplementation initiation

Figure S3.12.8 Subgroup analysis by maternal population mean 25(OH)D concentration (30 nmol/L)

Figure S3.12.9 Subgroup analysis by maternal population mean 25(OH)D concentration (50 nmol/L)

**13. Preterm birth (<37 weeks)**

Figure S3.13.1 Primary analysis

Figure S3.13.2 Subgroup analysis by intervention type

Figure S3.13.3 Subgroup analysis by population type

Figure S3.13.4 Subgroup analysis by intervention dose

Figure S3.13.5 Subgroup analysis by administration frequency

Figure S3.13.6 Subgroup analysis by supplement form

Figure S3.13.7 Subgroup analysis by trimester of supplementation initiation

Figure S3.13.8 Subgroup analysis by maternal population mean 25(OH)D concentration (30 nmol/L)

Figure S3.13.9 Subgroup analysis by maternal population mean 25(OH)D concentration (50 nmol/L)

**14. Small for gestational age (birthweight <10th percentile)**

Figure S3.14.1 Primary analysis

Figure S3.14.2 Subgroup analysis by intervention type

Figure S3.14.3 Subgroup analysis by population type

Figure S3.14.4 Subgroup analysis by intervention dose

Figure S3.14.5 Subgroup analysis by administration frequency

Figure S3.14.6 Subgroup analysis by supplement form

Figure S3.14.7 Subgroup analysis by trimester of supplementation initiation

Figure S3.14.8 Subgroup analysis by maternal population mean 25(OH)D concentration (30 nmol/L)

Figure S3.14.9 Subgroup analysis by maternal population mean 25(OH)D concentration (50 nmol/L)

**15. Congenital malformations**

Figure S3.15.1 Primary analysis

Figure S3.15.2 Subgroup analysis by intervention type

Figure S3.15.3 Subgroup analysis by population type

Figure S3.15.4 Subgroup analysis by intervention dose

Figure S3.15.5 Subgroup analysis by administration frequency

Figure S3.15.6 Subgroup analysis by supplement form

Figure S3.15.7 Subgroup analysis by trimester of supplementation initiation

Figure S3.15.8 Subgroup analysis by maternal population mean 25(OH)D concentration (30 nmol/L)

Figure S3.15.9 Subgroup analysis by maternal population mean 25(OH)D concentration (50 nmol/L)

**16. Admission to neonatal intensive care unit (NICU)**

Figure S3.16.1 Primary analysis

Figure S3.16.2 Subgroup analysis by intervention type

Figure S3.16.3 Subgroup analysis by population type

Figure S3.16.4 Subgroup analysis by intervention dose

Figure S3.16.5 Subgroup analysis by administration frequency

Figure S3.16.6 Subgroup analysis by supplement form

Figure S3.16.7 Subgroup analysis by trimester of supplementation initiation

Figure S3.16.8 Subgroup analysis by maternal population mean 25(OH)D concentration (30 nmol/L)

Figure S3.16.9 Subgroup analysis by maternal population mean 25(OH)D concentration (50 nmol/L)

**17. Gestational age (weeks)**

Figure S3.17.1 Primary analysis

Figure S3.17.2 Subgroup analysis by intervention type

Figure S3.17.3 Subgroup analysis by population type

Figure S3.17.4 Subgroup analysis by intervention dose

Figure S3.17.5 Subgroup analysis by administration frequency

Figure S3.17.6 Subgroup analysis by supplement form

Figure S3.17.7 Subgroup analysis by trimester of supplementation initiation

Figure S3.17.8 Subgroup analysis by maternal population mean 25(OH)D concentration (30 nmol/L)

Figure S3.17.9 Subgroup analysis by maternal population mean 25(OH)D concentration (50 nmol/L)

**18. Birthweight (g)**

Figure S3.18.1 Primary analysis

Figure S3.18.2 Subgroup analysis by intervention type

Figure S3.18.3 Subgroup analysis by population type

Figure S3.18.4 Subgroup analysis by intervention dose

Figure S3.18.5 Subgroup analysis by administration frequency

Figure S3.18.6 Subgroup analysis by supplement form

Figure S3.18.7 Subgroup analysis by trimester of supplementation initiation

Figure S3.18.8 Subgroup analysis by maternal population mean 25(OH)D concentration (30 nmol/L)

Figure S3.18.9 Subgroup analysis by maternal population mean 25(OH)D concentration (50 nmol/L)

**19. Birth body length (cm)**

Figure S3.19.1 Primary analysis

Figure S3.19.2 Subgroup analysis by intervention type

Figure S3.19.3 Subgroup analysis by population type

Figure S3.19.4 Subgroup analysis by intervention dose

Figure S3.19.5 Subgroup analysis by administration frequency

Figure S3.19.6 Subgroup analysis by supplement form

Figure S3.19.7 Subgroup analysis by trimester of supplementation initiation

Figure S3.19.8 Subgroup analysis by maternal population mean 25(OH)D concentration (30 nmol/L)

Figure S3.19.9 Subgroup analysis by maternal population mean 25(OH)D concentration (50 nmol/L)

**20. Birth head circumference (cm)**

Figure S3.20.1 Primary analysis

Figure S3.20.2 Subgroup analysis by intervention type

Figure S3.20.3 Subgroup analysis by population type

Figure S3.20.4 Subgroup analysis by intervention dose

Figure S3.20.5 Subgroup analysis by administration frequency

Figure S3.20.6 Subgroup analysis by supplement form

Figure S3.20.7 Subgroup analysis by trimester of supplementation initiation

Figure S3.20.8 Subgroup analysis by maternal population mean 25(OH)D concentration (30 nmol/L)

Figure S3.20.9 Subgroup analysis by maternal population mean 25(OH)D concentration (50 nmol/L)

**21. Cord 25(OH)D concentration (nmol/L)**

Figure S3.21.1 Primary analysis

Figure S3.21.2 Subgroup analysis by intervention type

Figure S3.21.3 Subgroup analysis by population type

Figure S3.21.4 Subgroup analysis by intervention dose

Figure S3.21.5 Subgroup analysis by administration frequency

Figure S3.21.6 Subgroup analysis by supplement form

Figure S3.21.7 Subgroup analysis by trimester of supplementation initiation

Figure S3.21.8 Subgroup analysis by maternal population mean 25(OH)D concentration (30 nmol/L)

Figure S3.21.9 Subgroup analysis by maternal population mean 25(OH)D concentration (50 nmol/L)

***Neonatal and infant outcomes***

**22. Neonatal death**

Figure S3.22.1 Primary analysis

Figure S3.22.2 Subgroup analysis by intervention type

Figure S3.22.3 Subgroup analysis by population type

Figure S3.22.4 Subgroup analysis by intervention dose

Figure S3.22.5 Subgroup analysis by administration frequency

Figure S3.22.6 Subgroup analysis by supplement form

Figure S3.22.7 Subgroup analysis by trimester of supplementation initiation

Figure S3.22.8 Subgroup analysis by maternal population mean 25(OH)D concentration (30 nmol/L)

Figure S3.22.9 Subgroup analysis by maternal population mean 25(OH)D concentration (50 nmol/L)

**23 Neonatal hypercalcemia**

Figure S3.23.1 Primary analysis

Figure S3.23.2 Sensitivity analysis

**24. Neonatal hypocalcemia**

Figure S3.24.1 Primary analysis

Figure S3.24.2 Sensitivity analysis

**25. Respiratory infection**

Figure S3.25.1 Primary analysis

**26. Upper respiratory tract infection**

Figure S3.26.1 Primary analysis

**27. Lower respiratory tract infection**

Figure S3.27.1 Primary analysis

Figure S3.27.2 Subgroup analysis by intervention type

Figure S3.27.3 Subgroup analysis by population type

Figure S3.27.4 Subgroup analysis by intervention dose

Figure S3.27.5 Subgroup analysis by administration frequency

Figure S3.27.6 Subgroup analysis by supplement form

Figure S3.27.7 Subgroup analysis by trimester of supplementation initiation

Figure S3.27.8 Subgroup analysis by maternal population mean 25(OH)D concentration (30 nmol/L)

Figure S3.27.9 Subgroup analysis by maternal population mean 25(OH)D concentration (50 nmol/L)

**28. Asthma or recurrent/persistent wheeze by 3 y/o**

Figure S3.28.1 Primary analysis

Figure S3.28.2 Sensitivity analysis

**29. Infant body weight at 1 year old (g)**

Figure S3.29.1 Primary analysis

Figure S3.29.2 Subgroup analysis by intervention type

Figure S3.29.3 Subgroup analysis by population type

Figure S3.29.4 Subgroup analysis by intervention dose

Figure S3.29.5 Subgroup analysis by administration frequency

Figure S3.29.6 Subgroup analysis by supplement form

Figure S3.29.7 Subgroup analysis by trimester of supplementation initiation

Figure S3.29.8 Subgroup analysis by maternal population mean 25(OH)D concentration (30 nmol/L)

Figure S3.29.9 Subgroup analysis by maternal population mean 25(OH)D concentration (50 nmol/L)

**30. Infant body length at 1 year old (cm)**

Figure S3.30.1 Primary analysis

Figure S3.30.2 Subgroup analysis by intervention type

Figure S3.30.3 Subgroup analysis by population type

Figure S3.30.4 Subgroup analysis by intervention dose

Figure S3.30.5 Subgroup analysis by administration frequency

Figure S3.30.6 Subgroup analysis by supplement form

Figure S3.30.7 Subgroup analysis by trimester of supplementation initiation

Figure S3.30.8 Subgroup analysis by maternal population mean 25(OH)D concentration (30 nmol/L)

Figure S3.30.9 Subgroup analysis by maternal population mean 25(OH)D concentration (50 nmol/L)

**31. Infant head circumference at 1 year old (cm)**

Figure S3.31.1 Primary analysis

**32. Weight-for-age z score at 1 year old**

Figure S3.32.1 Primary analysis

Figure S3.32.2 Subgroup analysis by intervention type

Figure S3.32.3 Subgroup analysis by population type

Figure S3.32.4 Subgroup analysis by intervention dose

Figure S3.32.5 Subgroup analysis by administration frequency

Figure S3.32.6 Subgroup analysis by supplement form

Figure S3.32.7 Subgroup analysis by trimester of supplementation initiation

Figure S3.32.8 Subgroup analysis by maternal population mean 25(OH)D concentration (30 nmol/L)

Figure S3.32.9 Subgroup analysis by maternal population mean 25(OH)D concentration (50 nmol/L)

**33. Length-for-age z score at 1 year old**

Figure S3.33.1 Primary analysis

Figure S3.33.2 Subgroup analysis by intervention type

Figure S3.33.3 Subgroup analysis by population type

Figure S3.33.4 Subgroup analysis by intervention dose

Figure S3.33.5 Subgroup analysis by administration frequency

Figure S3.33.6 Subgroup analysis by supplement form

Figure S3.33.7 Subgroup analysis by trimester of supplementation initiation

Figure S3.33.8 Subgroup analysis by maternal population mean 25(OH)D concentration (30 nmol/L)

Figure S3.33.9 Subgroup analysis by maternal population mean 25(OH)D concentration (50 nmol/L)

**34. Head circumference-for-age z score at 1 year old**

Figure S3.34.1 Primary analysis

**35. Neonatal bone mineral content (g)**

Figure S3.35.1 Primary analysis

**36. Neonatal bone mineral density (g/cm^2^)**

Figure S3.36.1 Primary analysis

**37. Infant bone mineral content (g)**

Figure S3.37.1 Primary analysis

**38. Infant bone mineral density (g/cm^2^)**

Figure S3.38.1 Primary analysis

**Definitions of primary analysis, sensitivity analysis, and subgroup analysis**

1. Primary analysis includes trials that reported outcomes in ways that meet outcome definitions.
2. Sensitivity analysis includes trials that reported outcomes regardless of meeting outcome definitions.
3. Subgroup analysis:
4. Intervention type:
   - Type 1: vitamin D alone *versus* control or no intervention
   - Type 2: vitamin D + calcium + other vitamins + minerals *versus* no vitamin D + calcium + other vitamins and minerals
   - Type 3: vitamin D *versus* active control of up to 600 IU/day
   - Type 4: vitamin D and/or calcium and/or other vitamins and/or minerals *versus* active control of up to 600 IU/day and/or calcium and/or other vitamins and/or minerals
5. Population type:
   - General population
   - Populations identified as high-risk groups for outcomes of interest
6. Vitamin D dosage of intervention arm:

- ≤600 IU/day
- 600 to ≤ 2000 IU/day
- >2000 IU/day

1. Administration frequency:

- Regular (daily, weekly, every two weeks, or monthly)
- Bolus

1. Trimester of intervention initiation:

- 1st trimester
- 2nd trimester
- 3rd trimester

1. Maternal baseline 25(OH)D: The Subgroup of maternal baseline vitamin D status was determined by the population mean 25(OH)D concentration of the control group. Two Subgroup analyses were performed using different thresholds.
   - ≥50 nmol/L or <50 nmol/L
   - ≥30 nmol/L or <30 nmol/L

**1. Preeclampsia**

**Figure S3.1.1** Primary analysis for the risk ratio of preeclampsia for women who were supplemented with vitamin D in pregnancy versus who were not

**
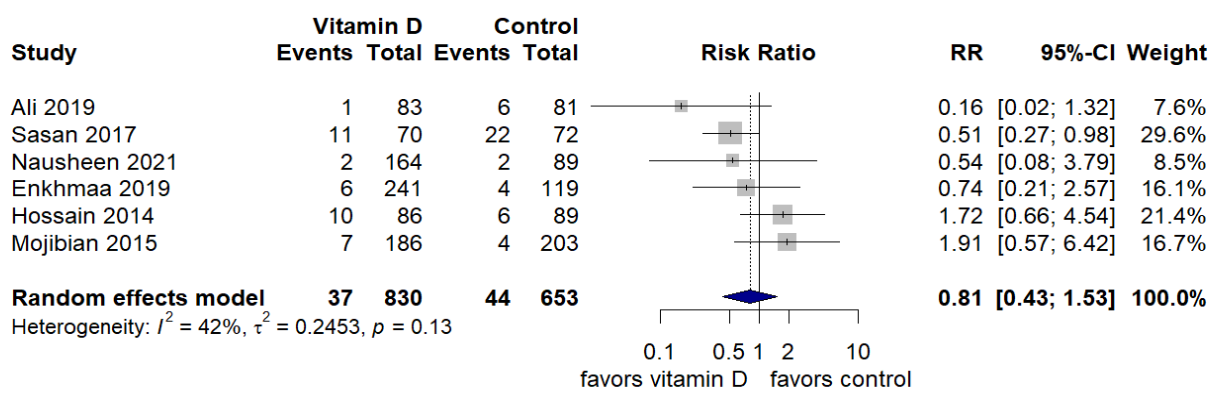
**

**Figure S3.1.2** Sensitivity analysis for the risk ratio of preeclampsia for women who were supplemented with vitamin D in pregnancy versus who were not


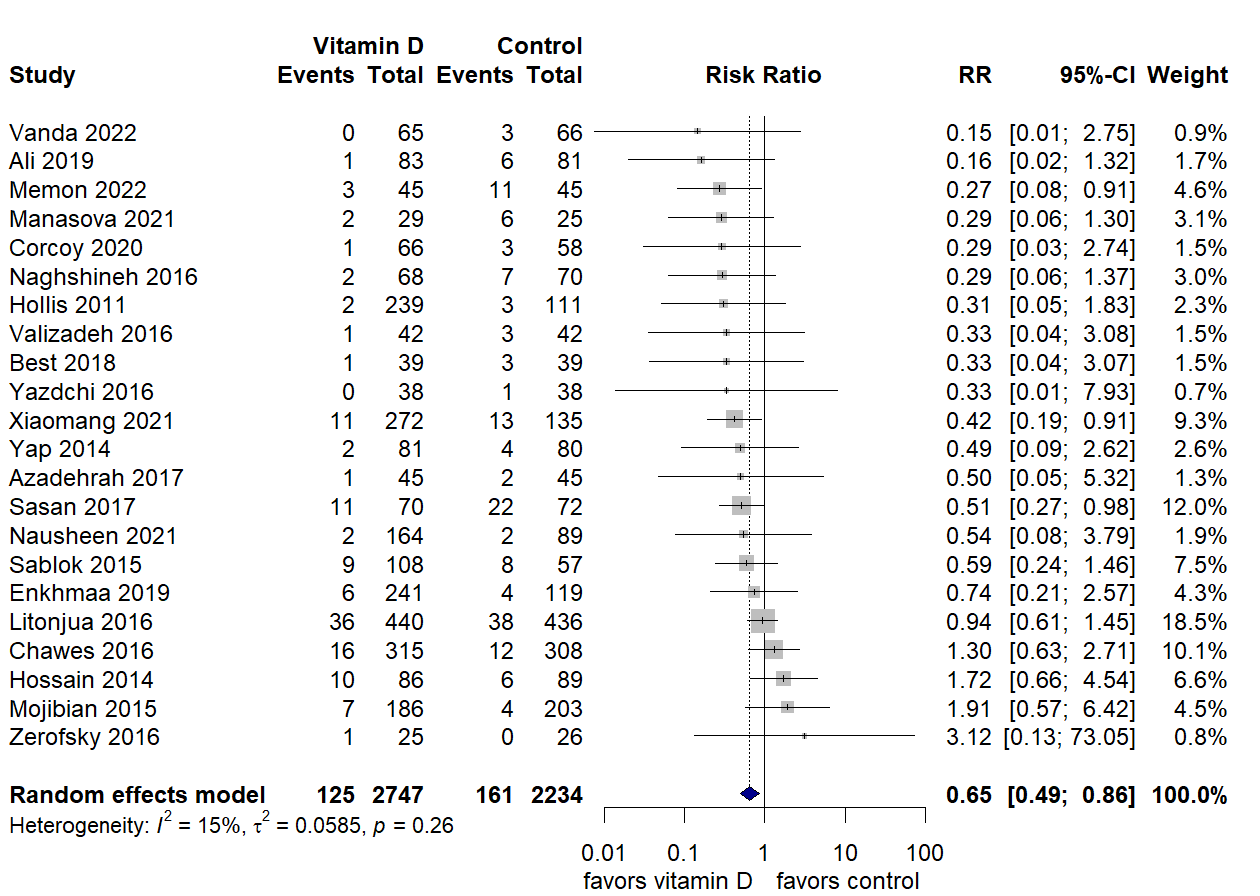


**Figure S3.1.3** Subgroup analysis by intervention type for the risk ratio of preeclampsia for women who were supplemented with vitamin D in pregnancy versus who were not


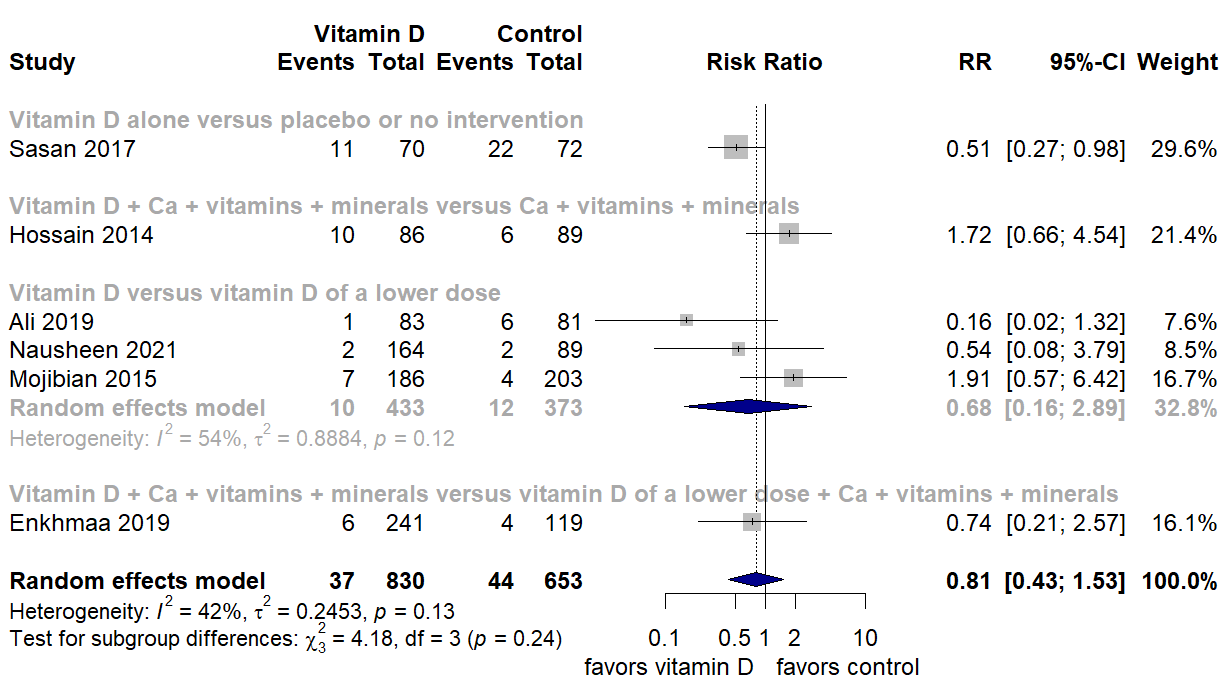


**Figure S3.1.4** Subgroup analysis by population type for the risk ratio of preeclampsia for women who were supplemented with vitamin D in pregnancy versus who were not


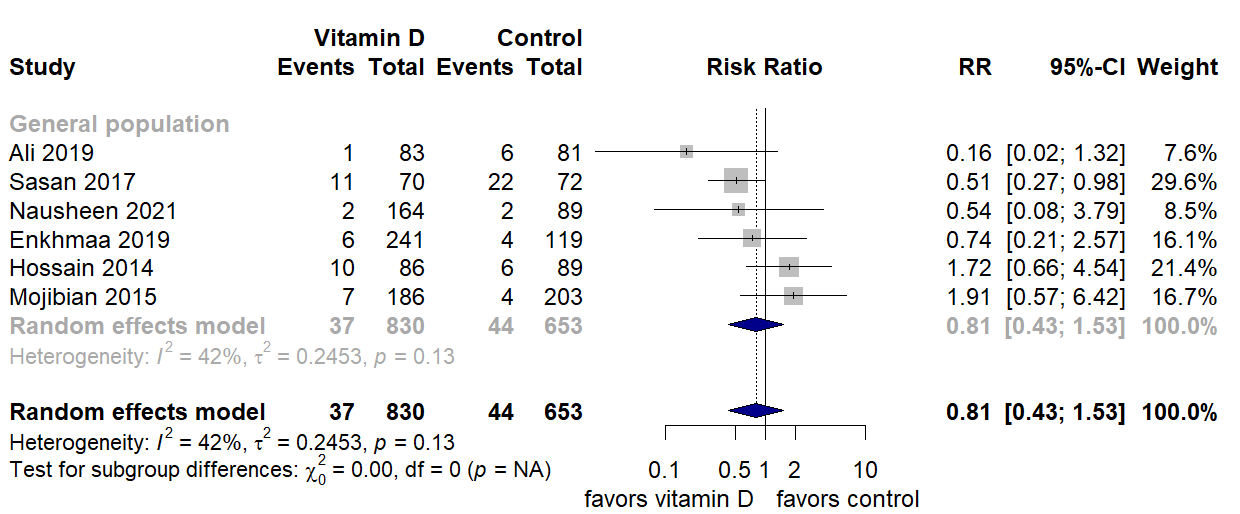


**Figure S3.1.5** Subgroup analysis by intervention dose for the risk ratio of preeclampsia for women who were supplemented with vitamin D in pregnancy versus who were not

**
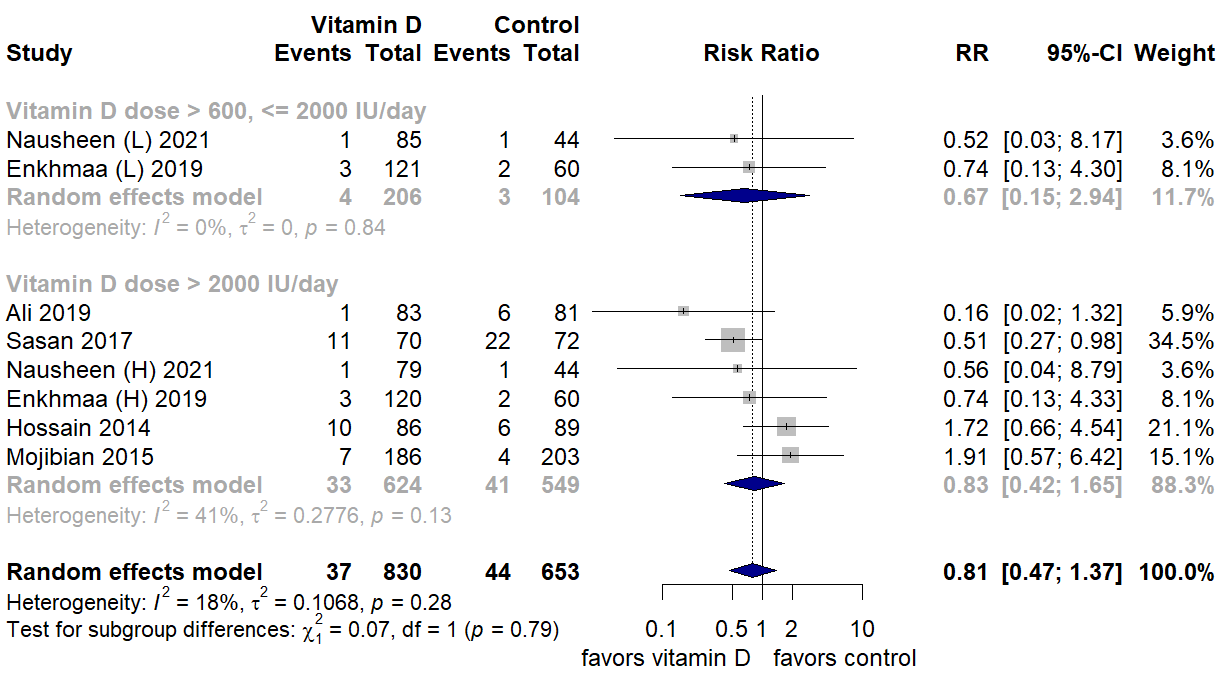
**

**Figure S3.1.6** Subgroup analysis by administration frequency for the risk ratio of preeclampsia for women who were supplemented with vitamin D in pregnancy versus who were not


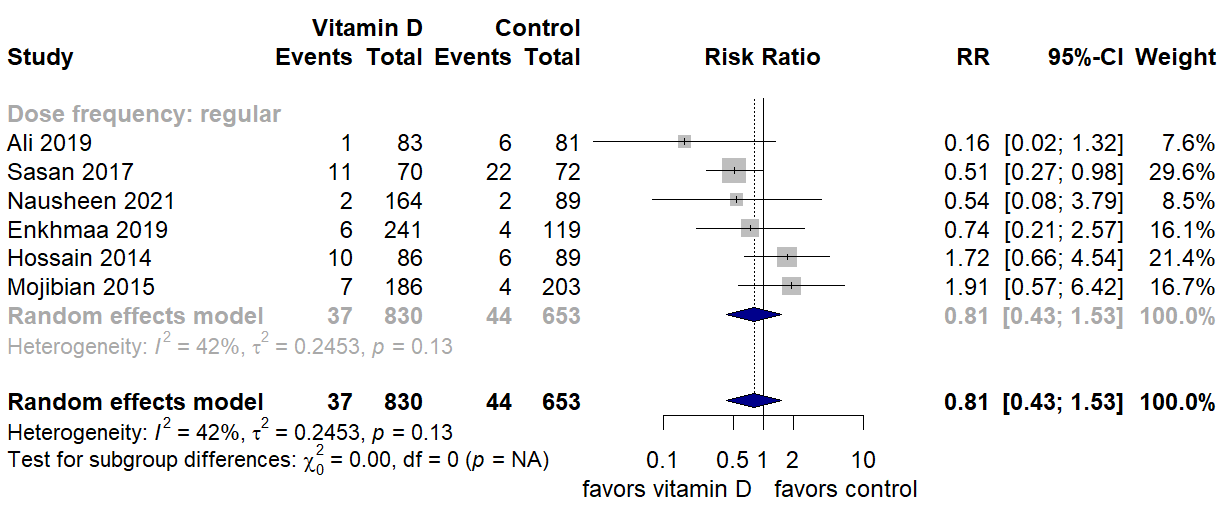


**Figure S3.1.7** Subgroup analysis by supplement form for the risk ratio of preeclampsia for women who were supplemented with vitamin D in pregnancy versus who were not


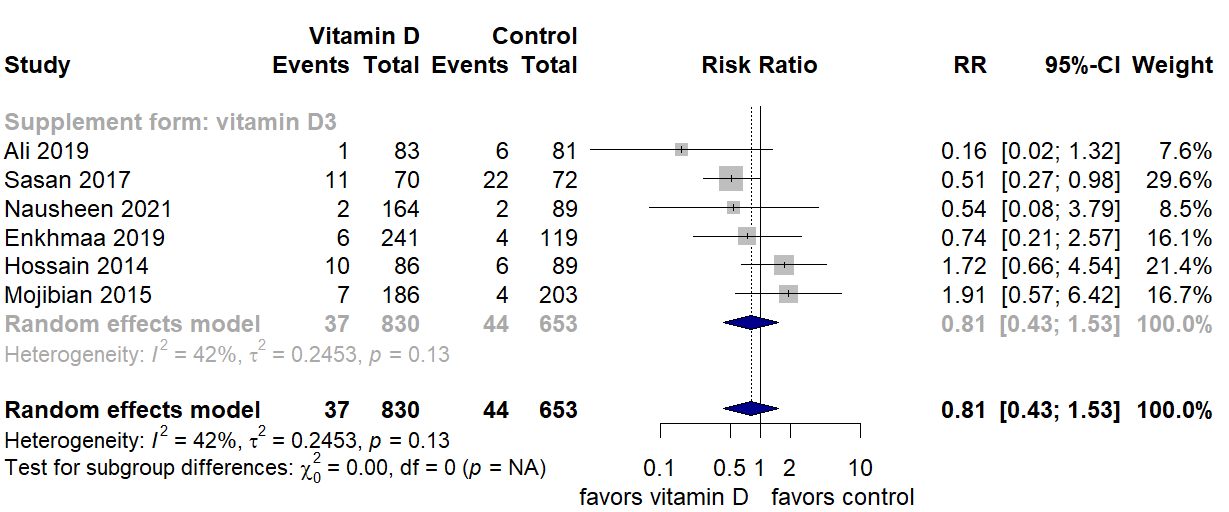


**Figure S3.1.8** Subgroup analysis by trimester of supplementation initiation for the risk ratio of preeclampsia for women who were supplemented with vitamin D in pregnancy versus who were not

**
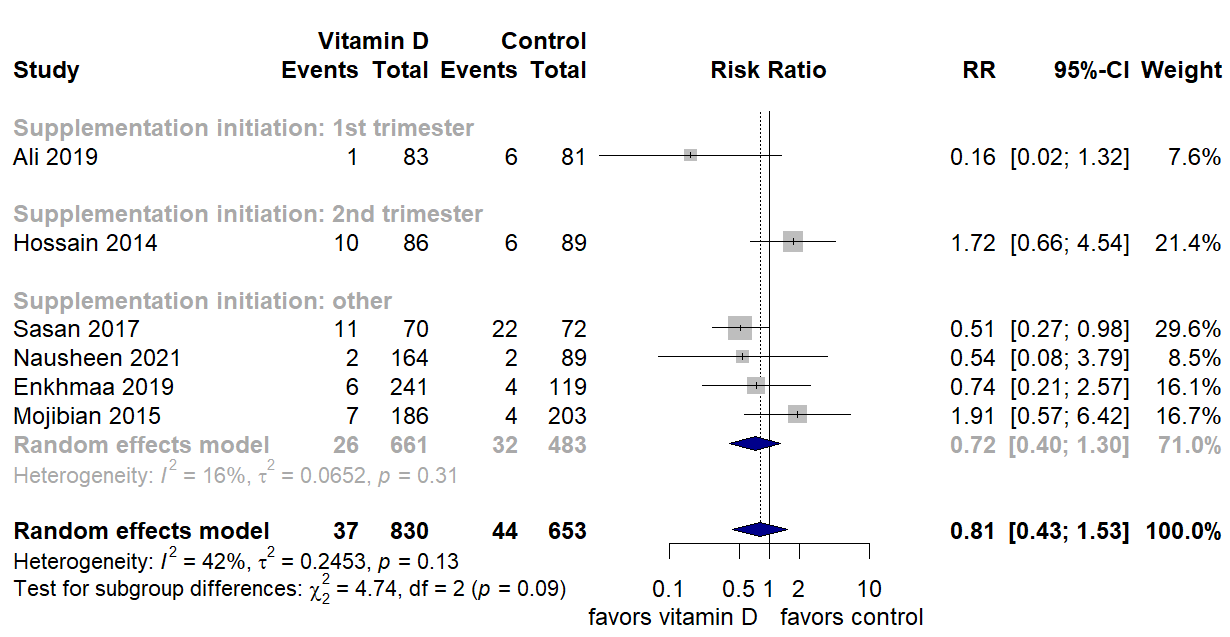
**

**Figure S3.1.9** Subgroup analysis by maternal population mean 25(OH)D concentration (30 nmol/L)for the risk ratio of preeclampsia for women who were supplemented with vitamin D in pregnancy versus who were not.

**
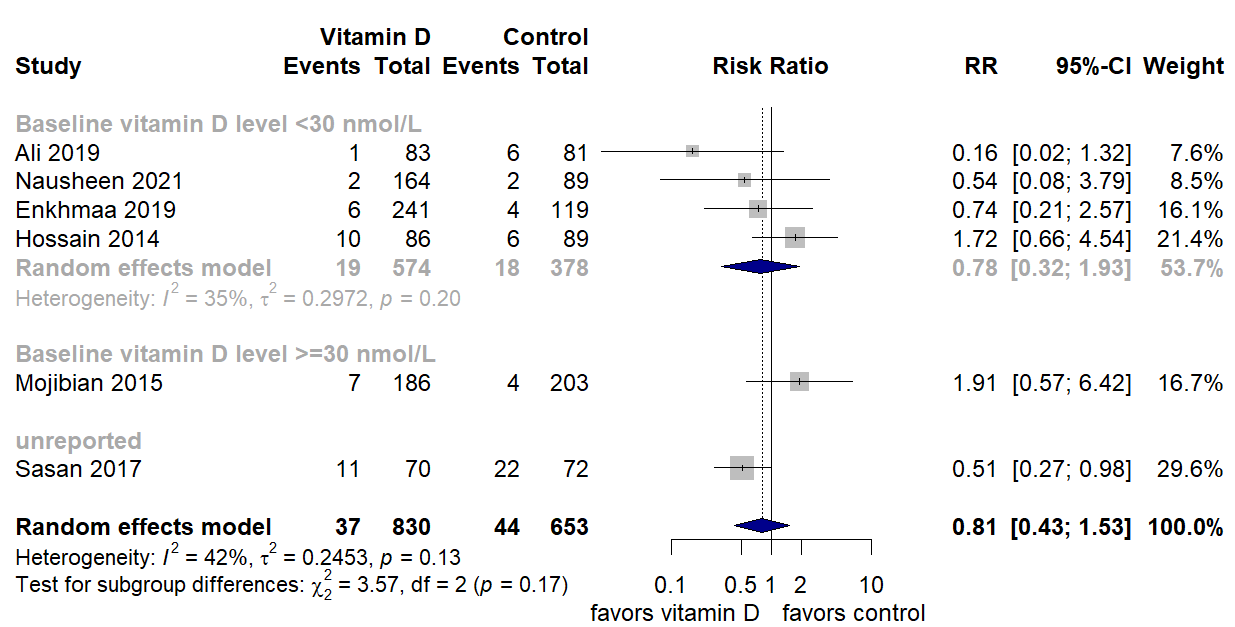
**

**Figure S3.1.10** Subgroup analysis by maternal population mean 25(OH)D concentration (50 nmol/L) for the risk ratio of preeclampsia for women who were supplemented with vitamin D in pregnancy versus who were not

**
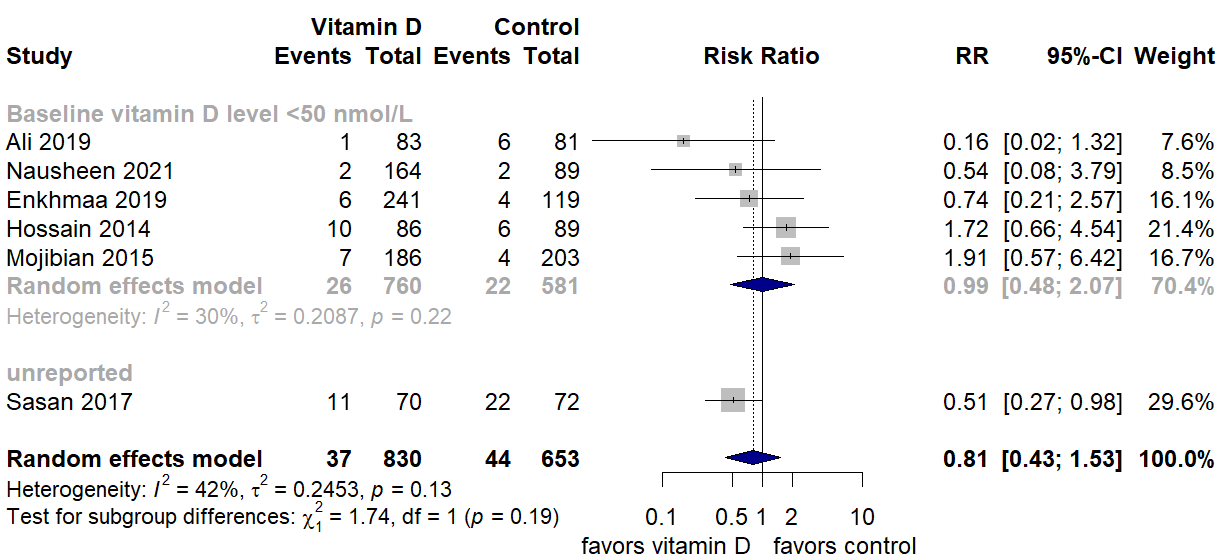
**

**2. Gestational hypertension**

**Figure S3.2.1** Primary analysis for the risk ratio of gestational hypertension for women who were supplemented with vitamin D in pregnancy versus who were not


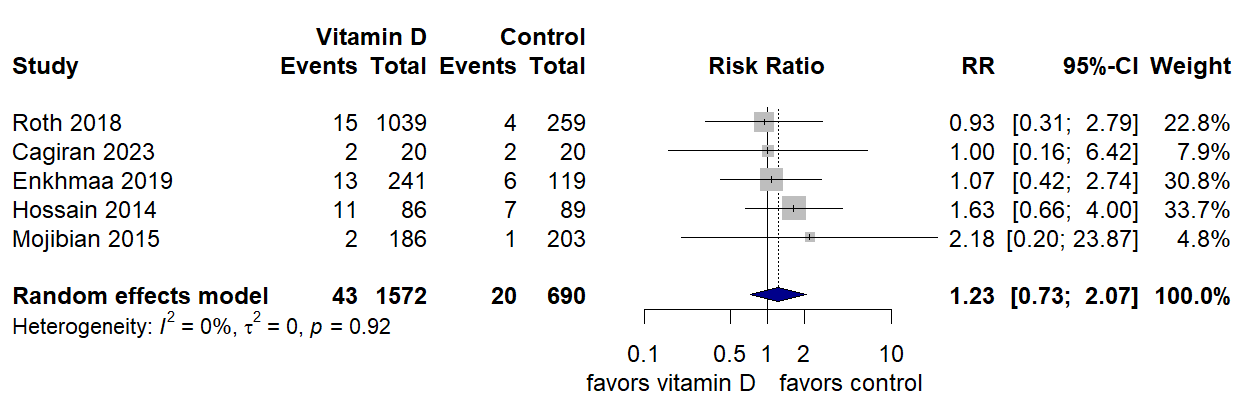


**Figure S3.2.2** Sensitivity analysis for the risk ratio of gestational hypertension for women who were supplemented with vitamin D in pregnancy versus who were not


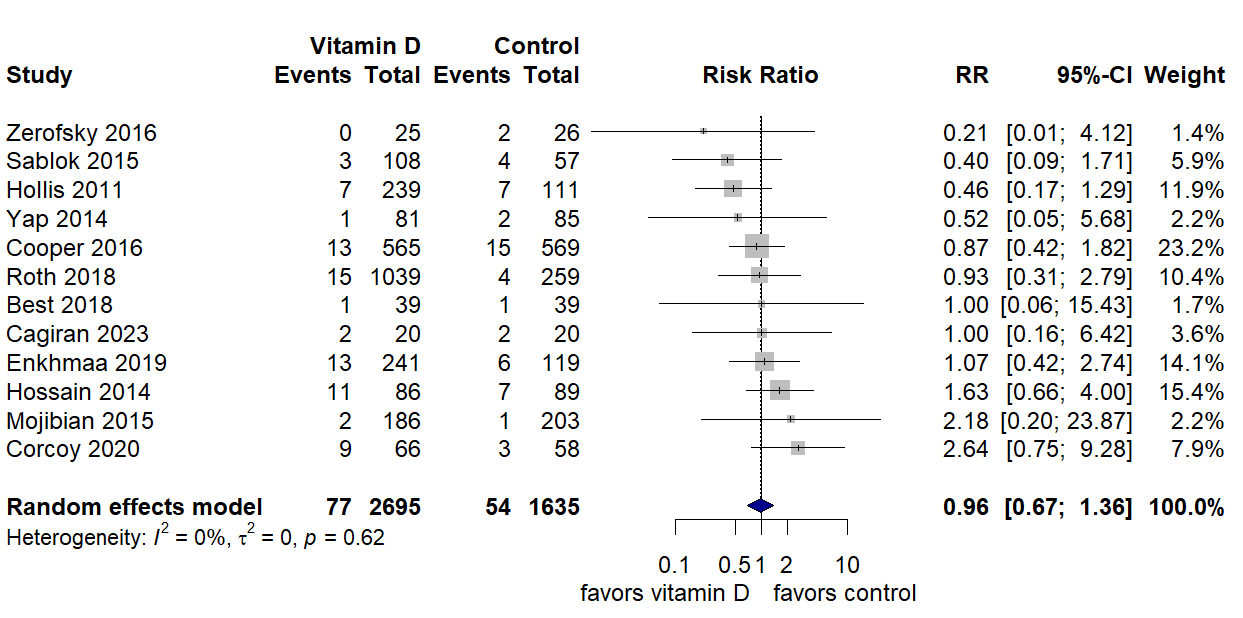


**Figure S3.2.3** Subgroup analysis by intervention type for the risk ratio of gestational hypertension for women who were supplemented with vitamin D in pregnancy versus who were not


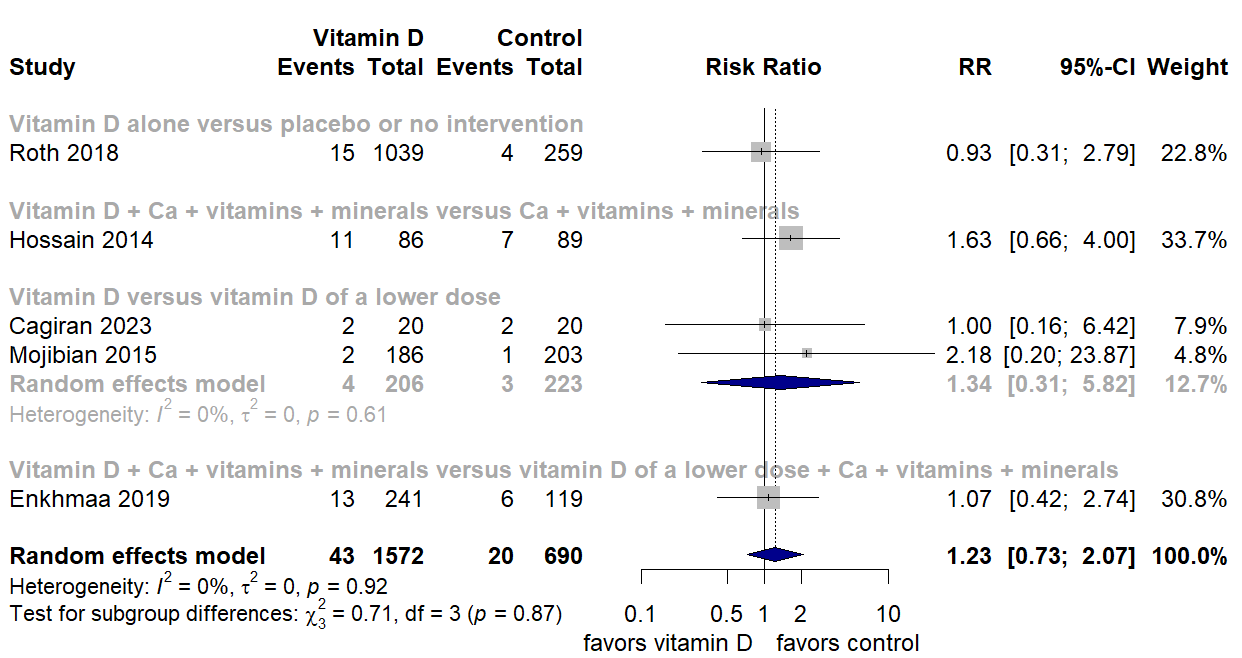


**Figure S3.2.4** Subgroup analysis by population type for the risk ratio of gestational hypertension for women who were supplemented with vitamin D in pregnancy versus who were not


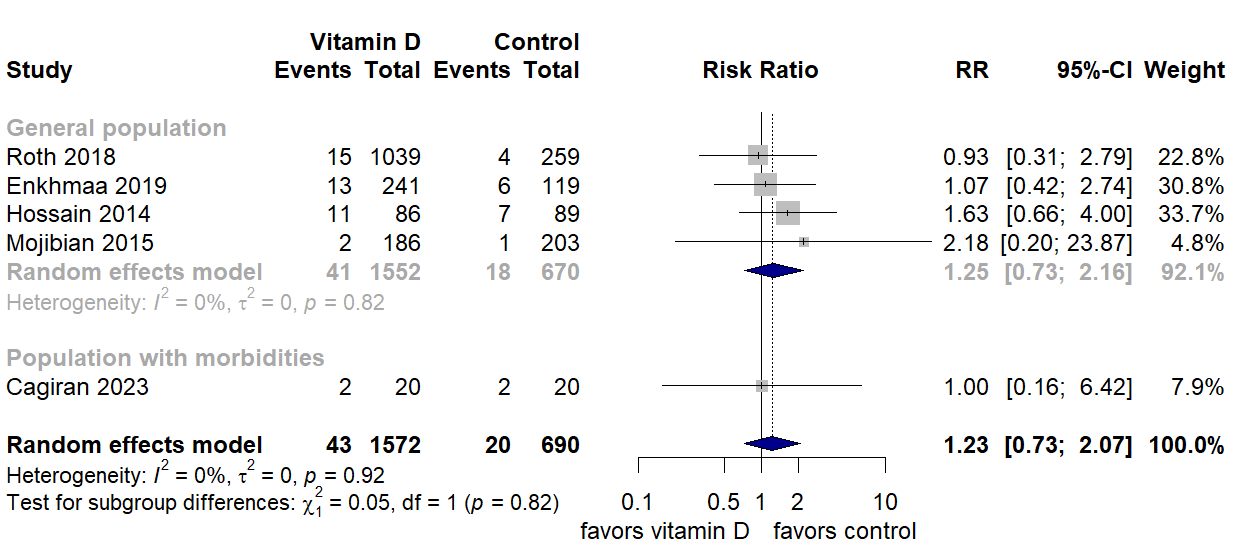


**Figure S3.2.5** Subgroup analysis by intervention dose for the risk ratio of gestational hypertension for women who were supplemented with vitamin D in pregnancy versus who were not


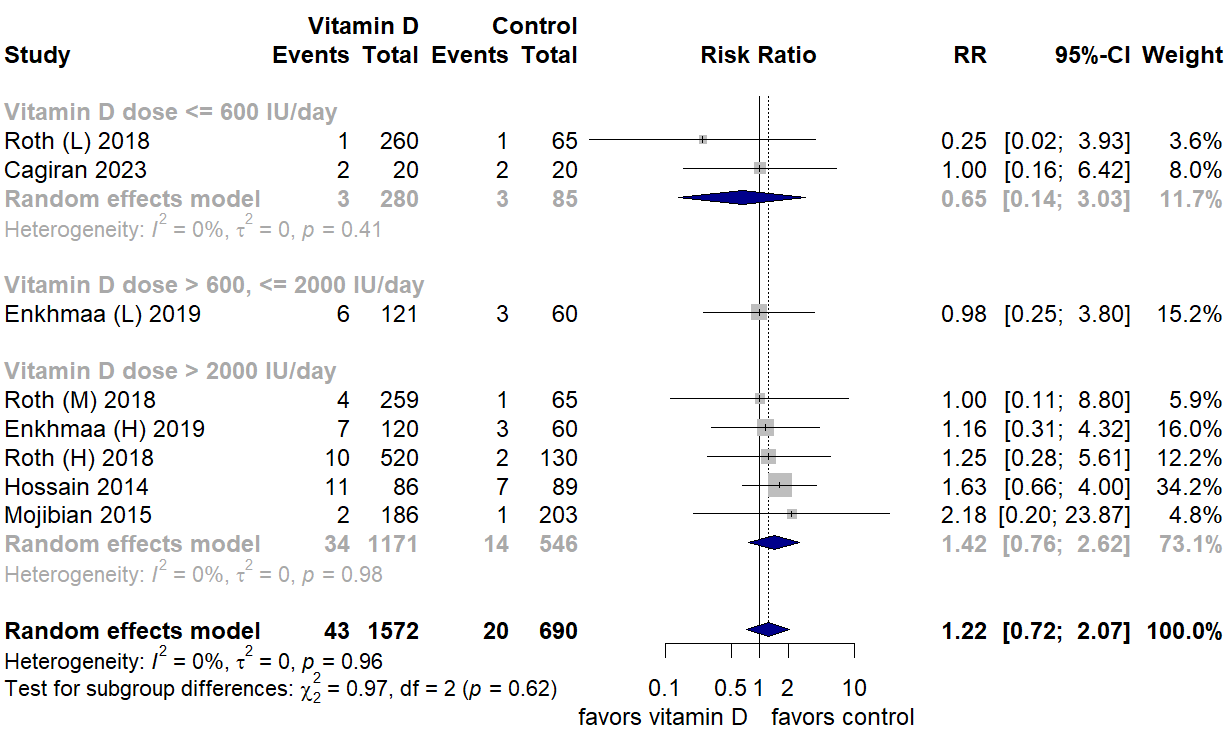


**Figure S3.2.6** Subgroup analysis by administration frequency for the risk ratio of gestational hypertension for women who were supplemented with vitamin D in pregnancy versus who were not


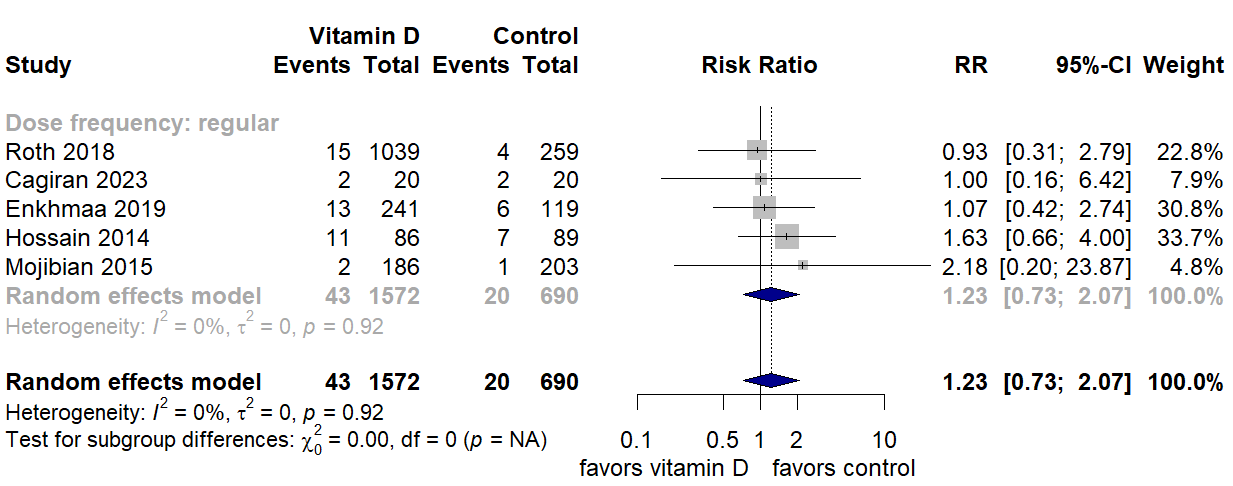


**Figure S3.2.7** Subgroup analysis by supplement form for the risk ratio of gestational hypertension for women who were supplemented with vitamin D in pregnancy versus who were not


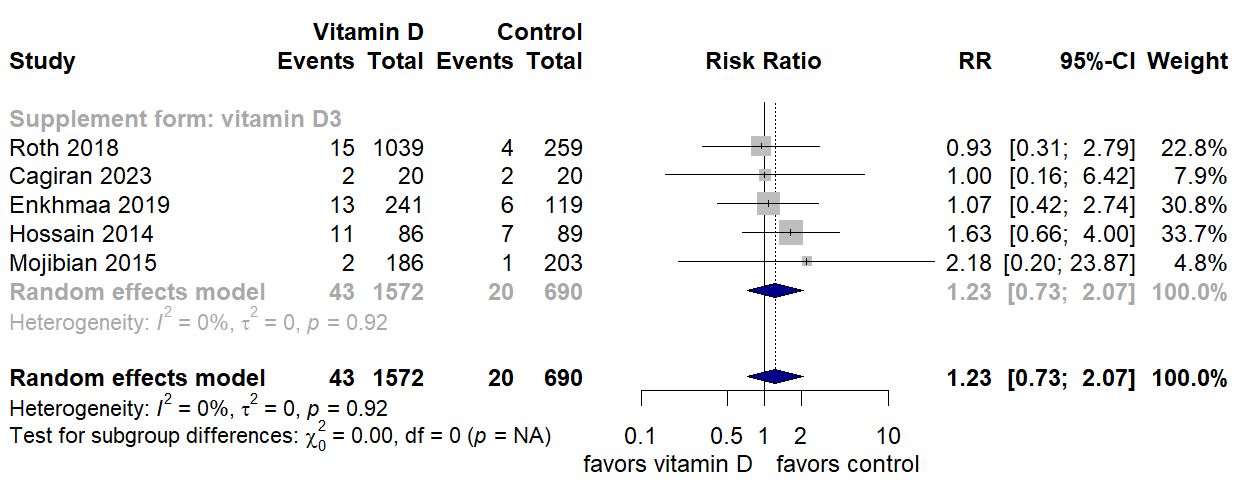


**Figure S3.2.8** Subgroup analysis by initiation of trimester of supplementation initiation for the risk ratio of gestational hypertension for women who were supplemented with vitamin D in pregnancy versus who were not

**
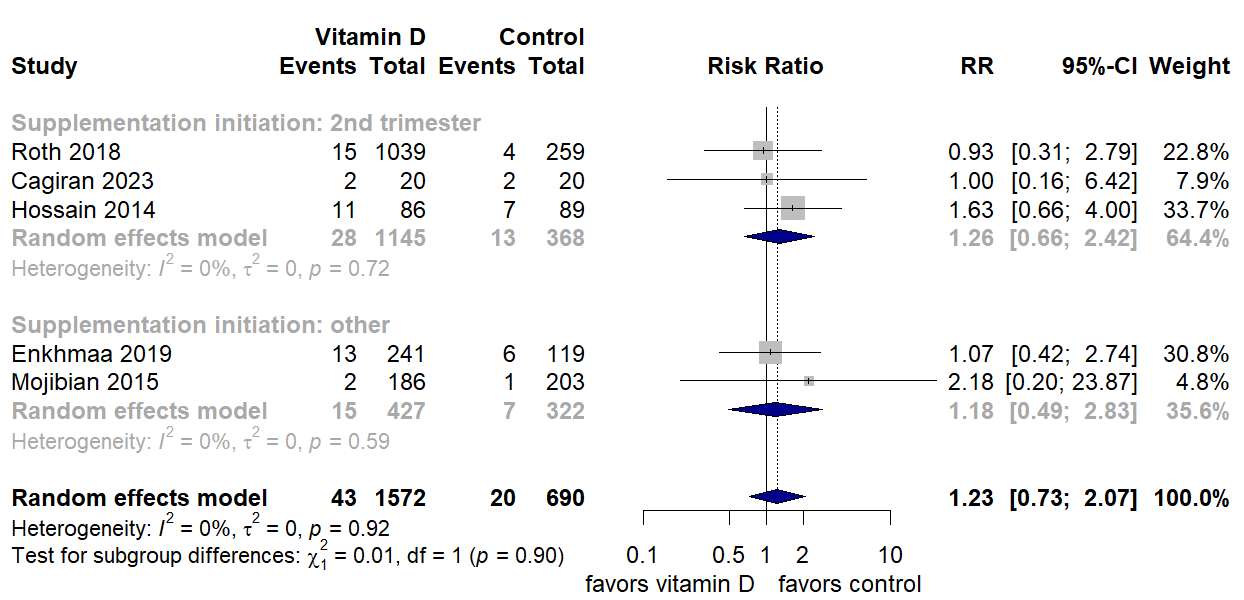
**

**Figure S3.2.9** Subgroup analysis by maternal population mean 25(OH)D concentration (30 nmol/L) for the risk ratio of gestational hypertension for women who were supplemented with vitamin D in pregnancy versus who were not

**
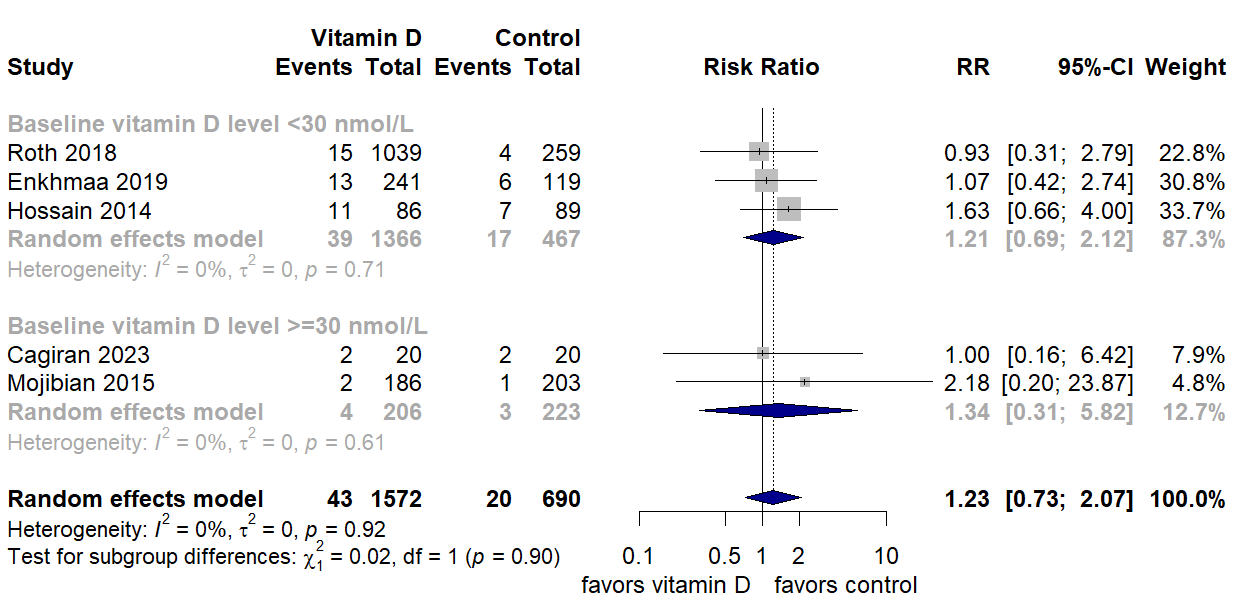
**

**Figure S3.2.10** Subgroup analysis by maternal population mean 25(OH)D concentration (50 nmol/L) for the risk ratio of gestational hypertension for women who were supplemented with vitamin D in pregnancy versus who were not


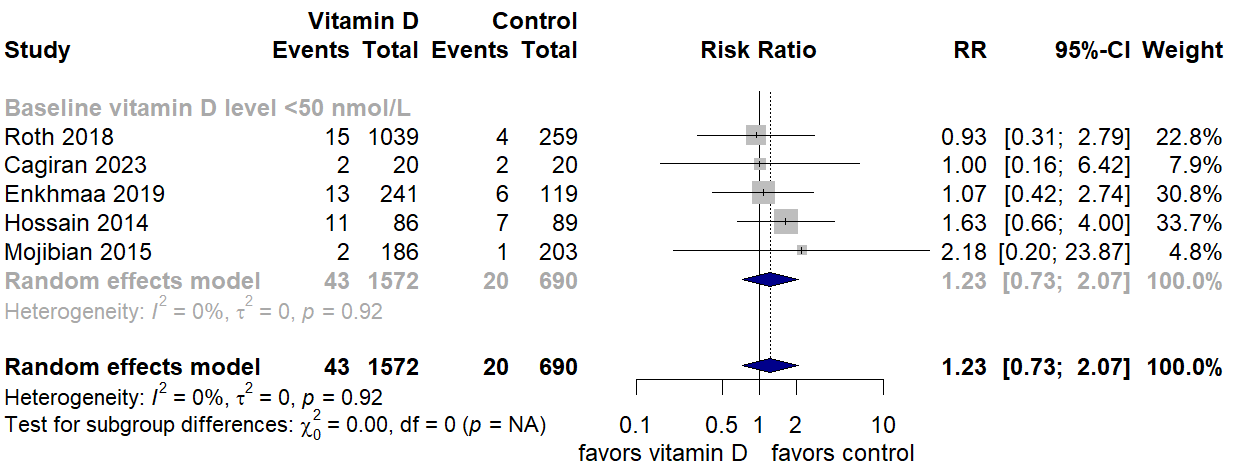


**3. Gestational diabetes**

**Figure S3.3.1** Primary analysis for the risk ratio of gestational diabetes for women who were supplemented with vitamin D in pregnancy versus who were not

**
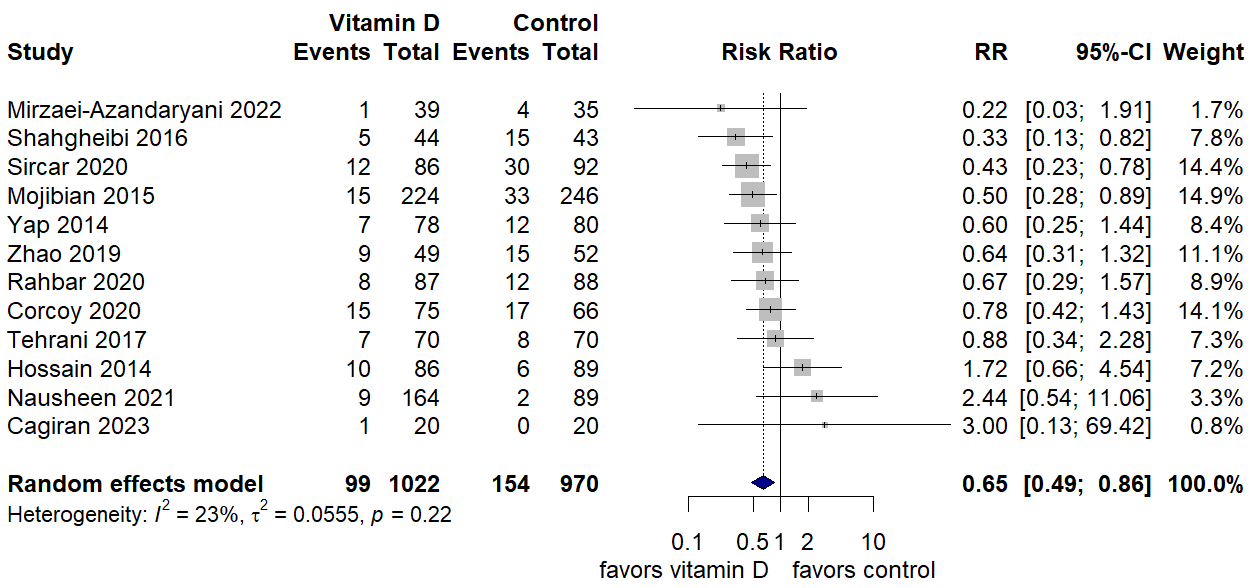
**

**Figure S3.3.2** Sensitivity analysis for the risk ratio of gestational diabetes for women who were supplemented with vitamin D in pregnancy versus who were not


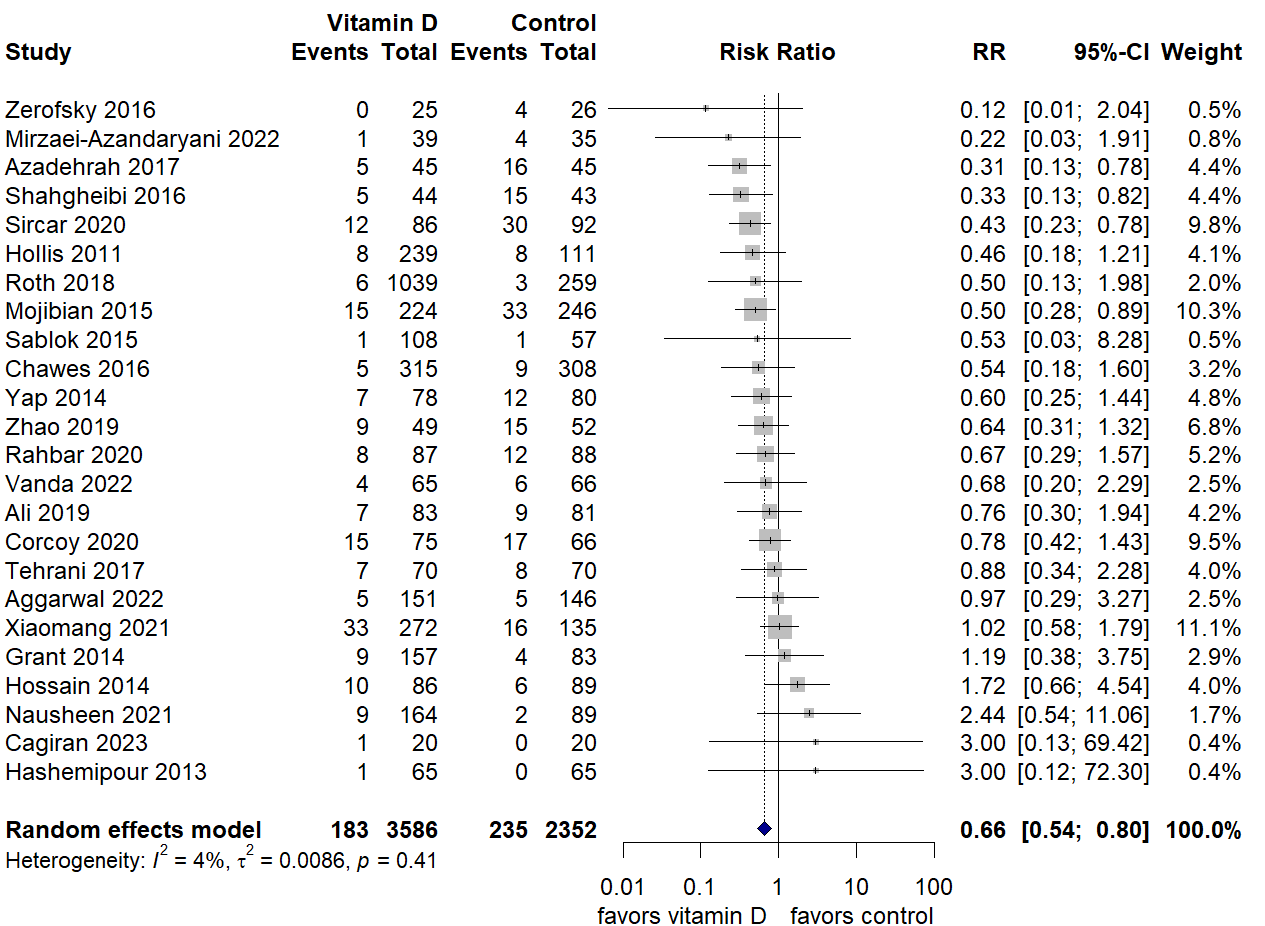


**Figure S3.3.3** Subgroup analysis by intervention type for the risk ratio of gestational diabetes for women who were supplemented with vitamin D in pregnancy versus who were not


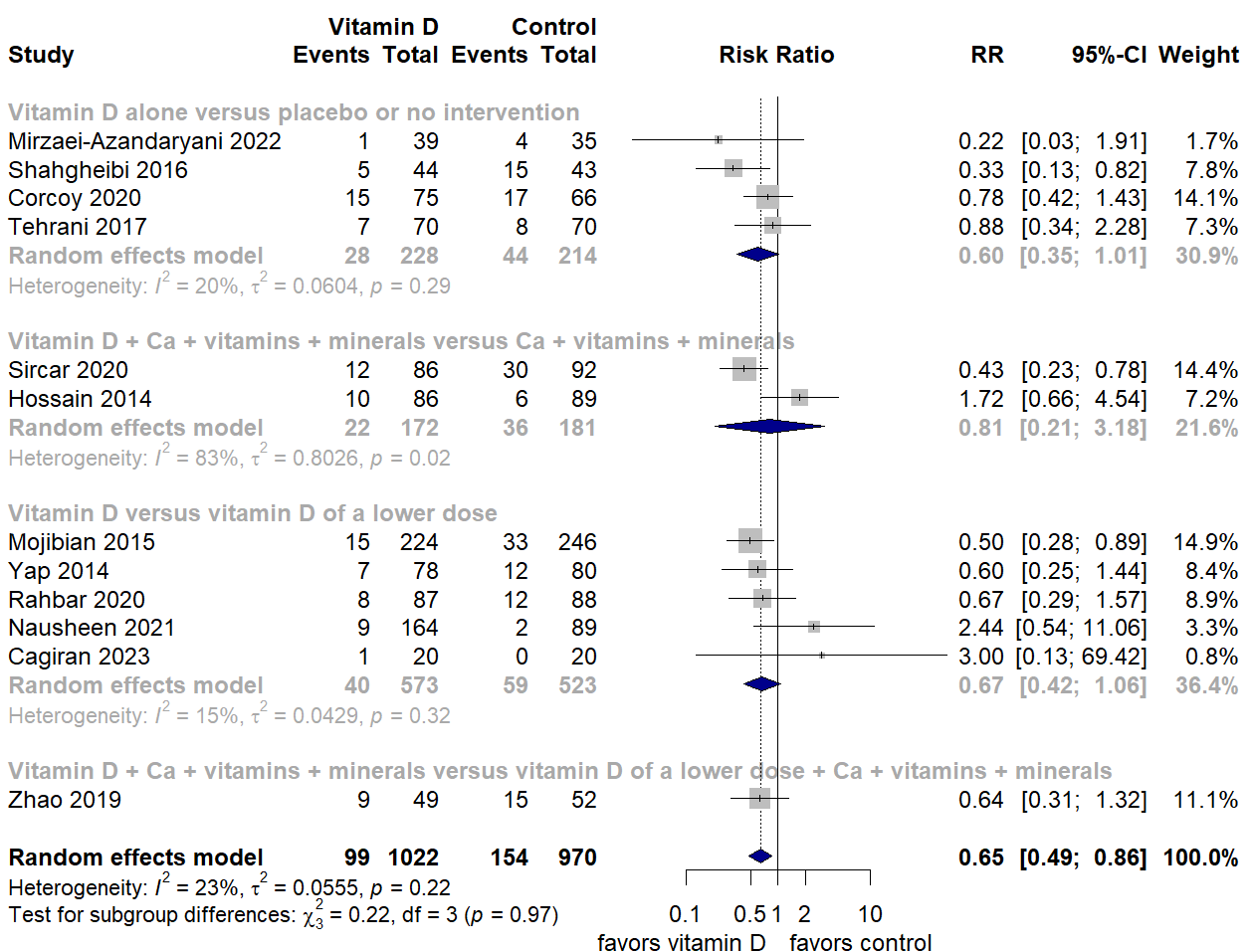


**Figure S3.3.4** Subgroup analysis by population type for the risk ratio of gestational diabetes for women who were supplemented with vitamin D in pregnancy versus who were not


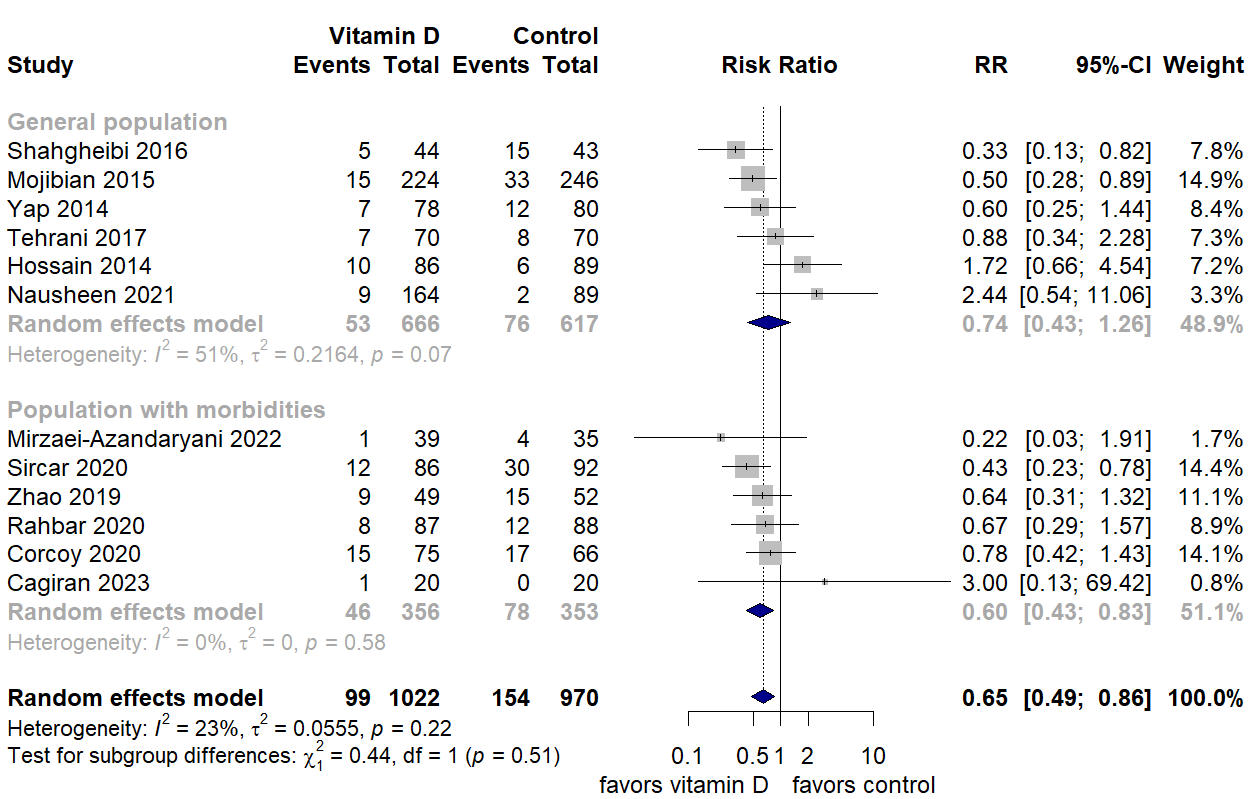


**Figure S3.3.5** Subgroup analysis by population health conditions for the risk ratio of gestational diabetes for women who were supplemented with vitamin D in pregnancy versus who were not

**
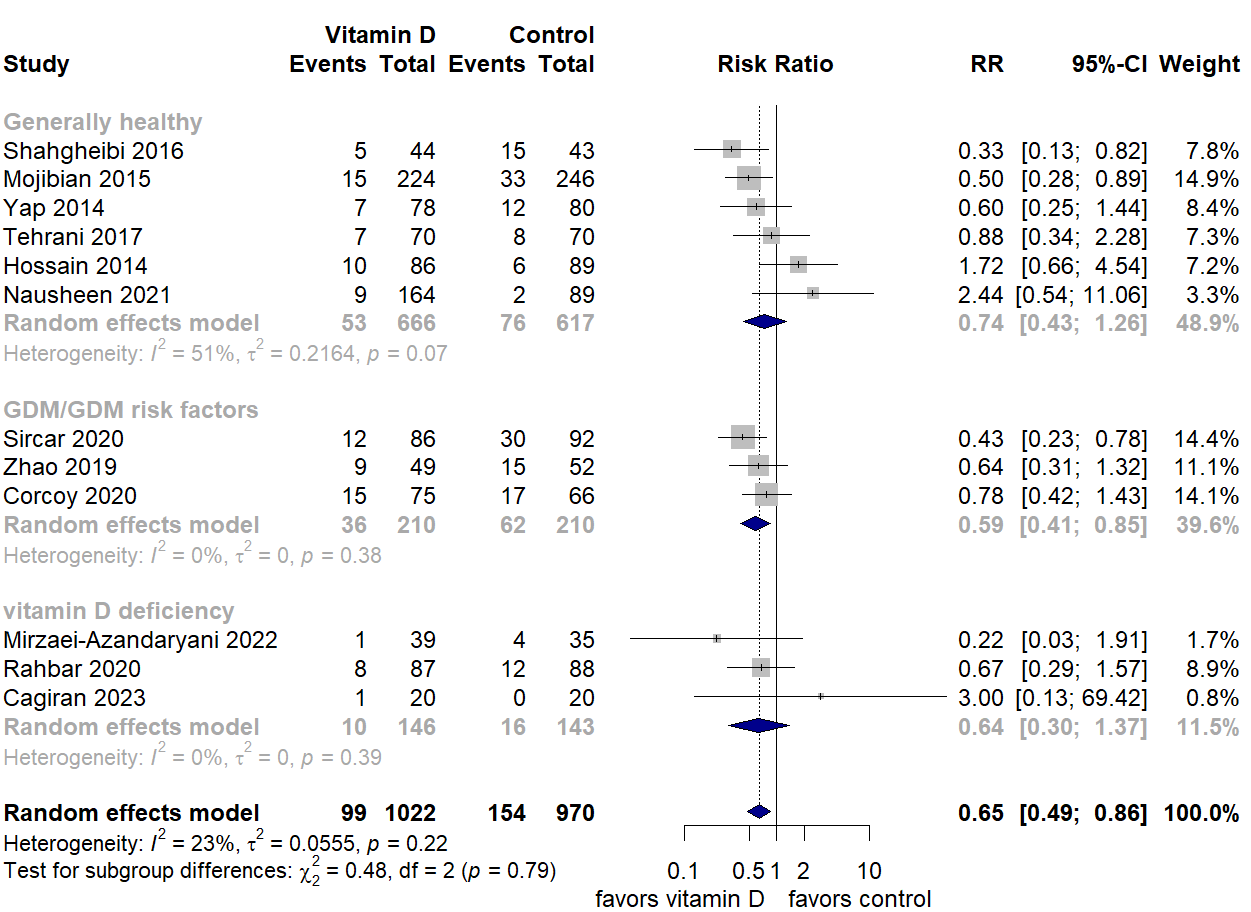
**

Note: Sircar 2020 was done among pregnant women with vitamin D deficiency and GDM risk factors.

**Figure S3.3.6** Subgroup analysis by intervention dose for the risk ratio of gestational diabetes for women who were supplemented with vitamin D in pregnancy versus who were not


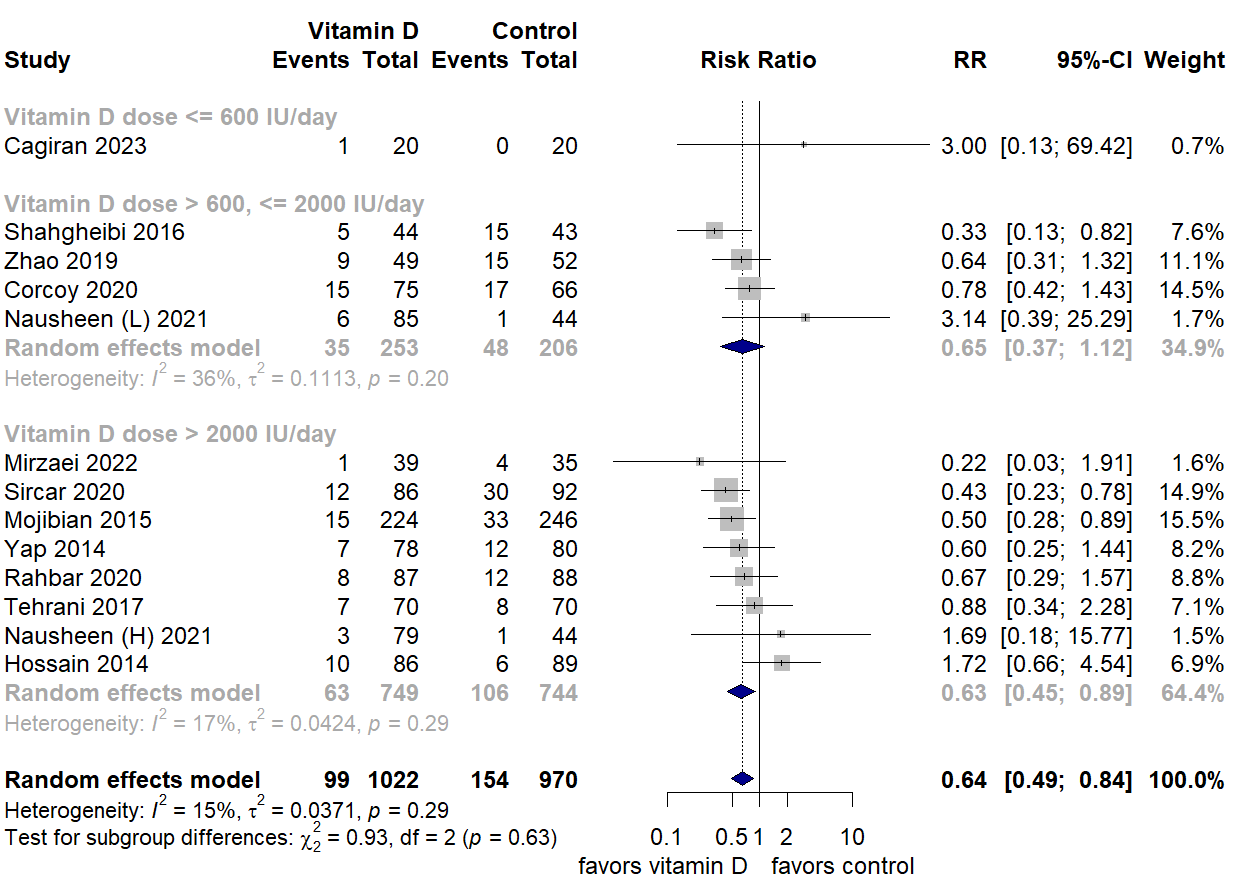


**Figure S3.3.7** Subgroup analysis by administration frequency for the risk ratio of gestational diabetes for women who were supplemented with vitamin D in pregnancy versus who were not


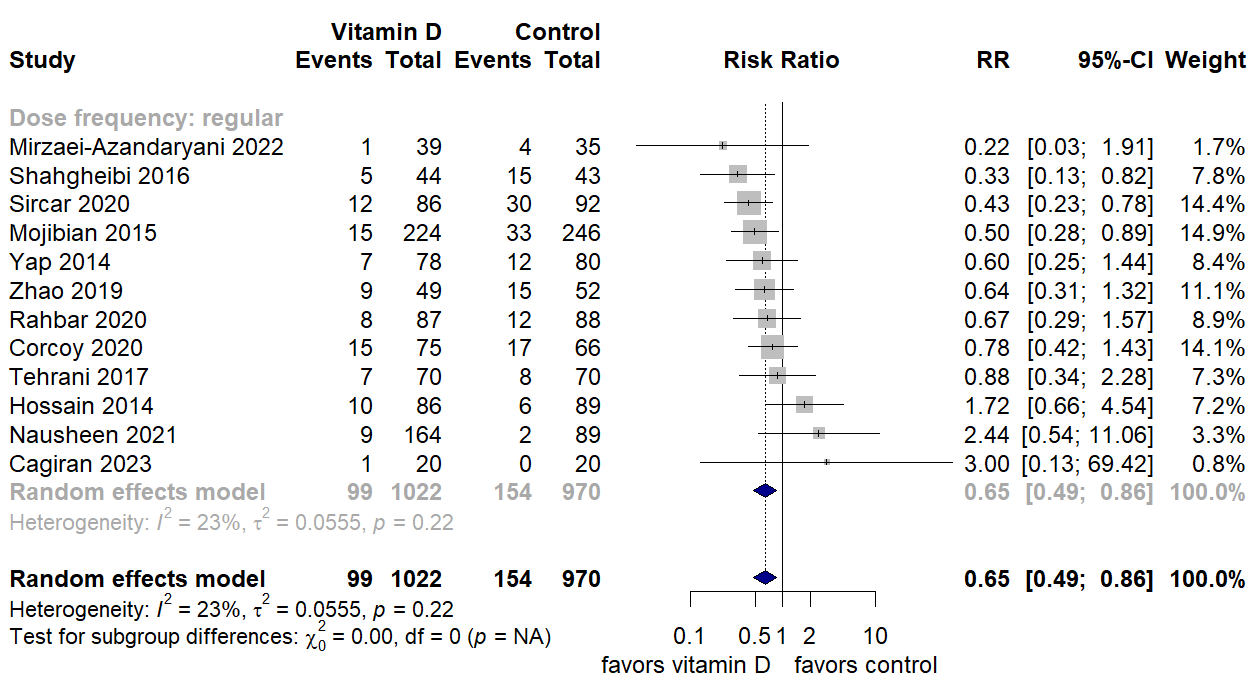


**Figure S3.3.8** Subgroup analysis by supplement form for the risk ratio of gestational diabetes for women who were supplemented with vitamin D in pregnancy versus who were not


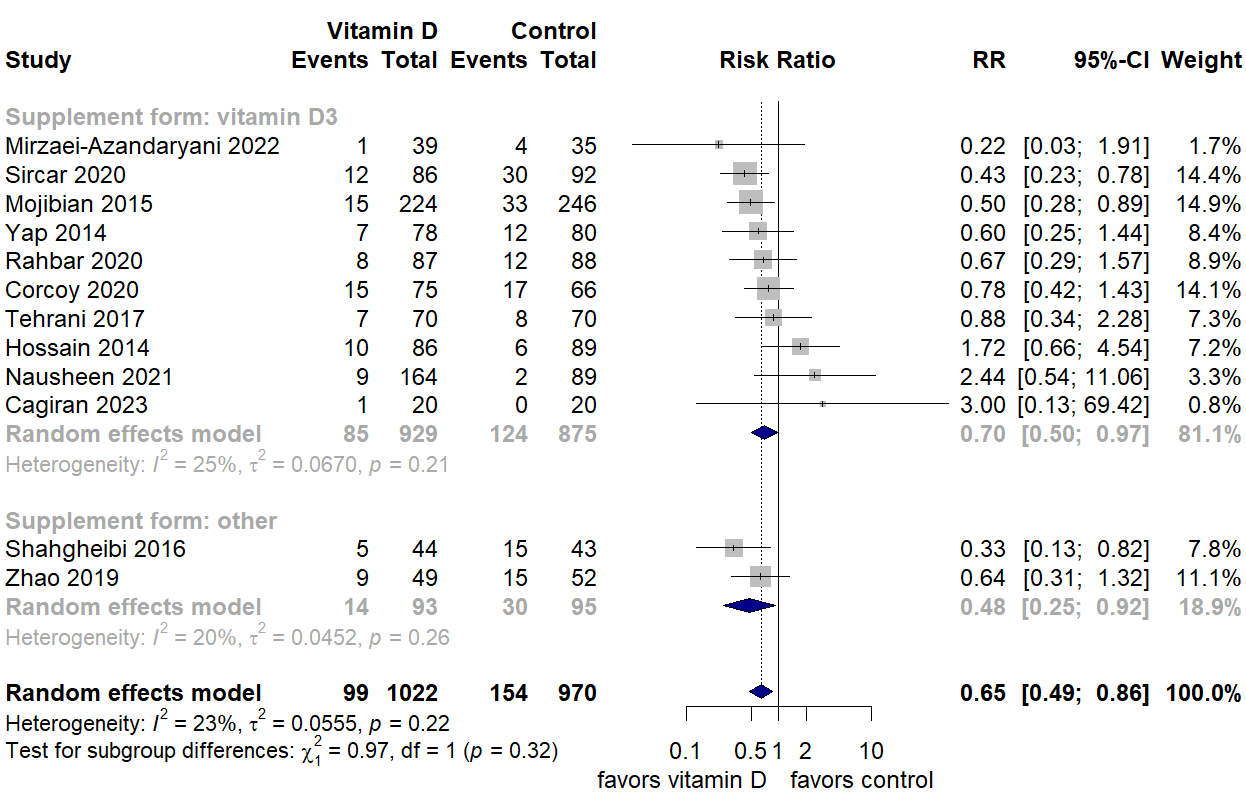


**Figure S3.3.9** Subgroup analysis by trimester of supplementation initiation for the risk ratio of gestational diabetes for women who were supplemented with vitamin D in pregnancy versus who were not

**
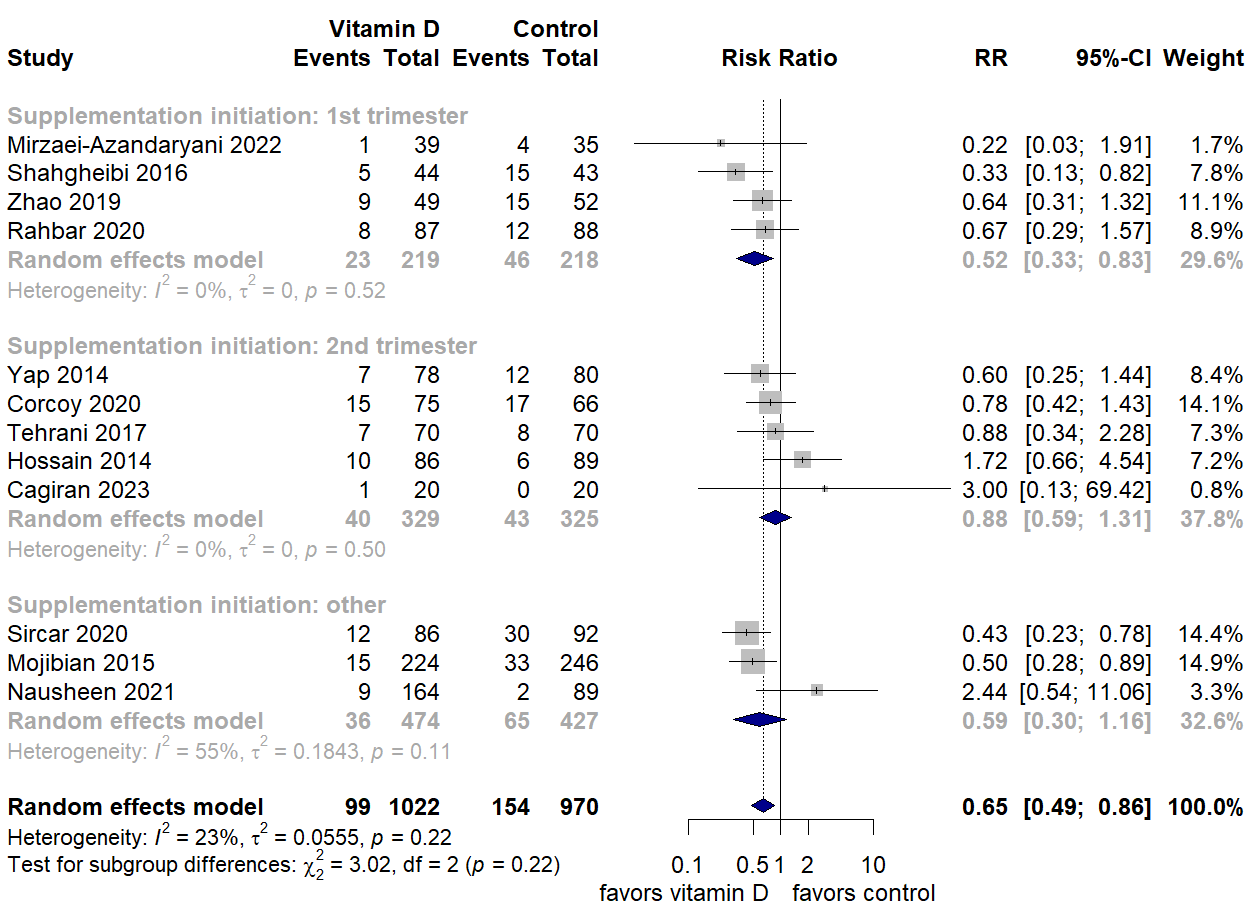
**

**Figure S3.3.10** Subgroup analysis by maternal population mean 25(OH)D concentration (30 nmol/L) for the risk ratio of gestational diabetes for women who were supplemented with vitamin D in pregnancy versus who were not

**
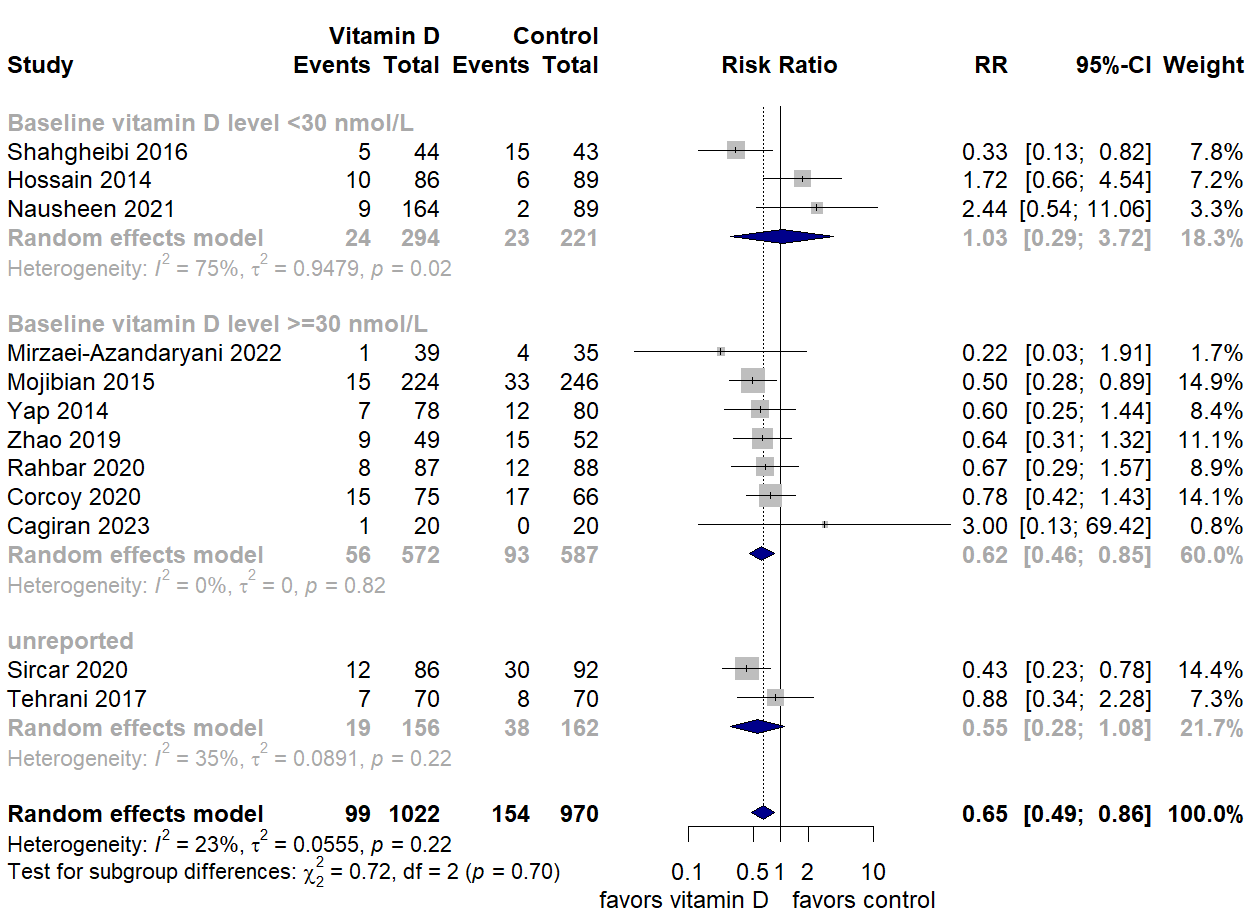
**

**Figure S3.3.11** Subgroup analysis by maternal population mean 25(OH)D concentration (50 nmol/L) for the risk ratio of gestational diabetes for women who were supplemented with vitamin D in pregnancy versus who were not


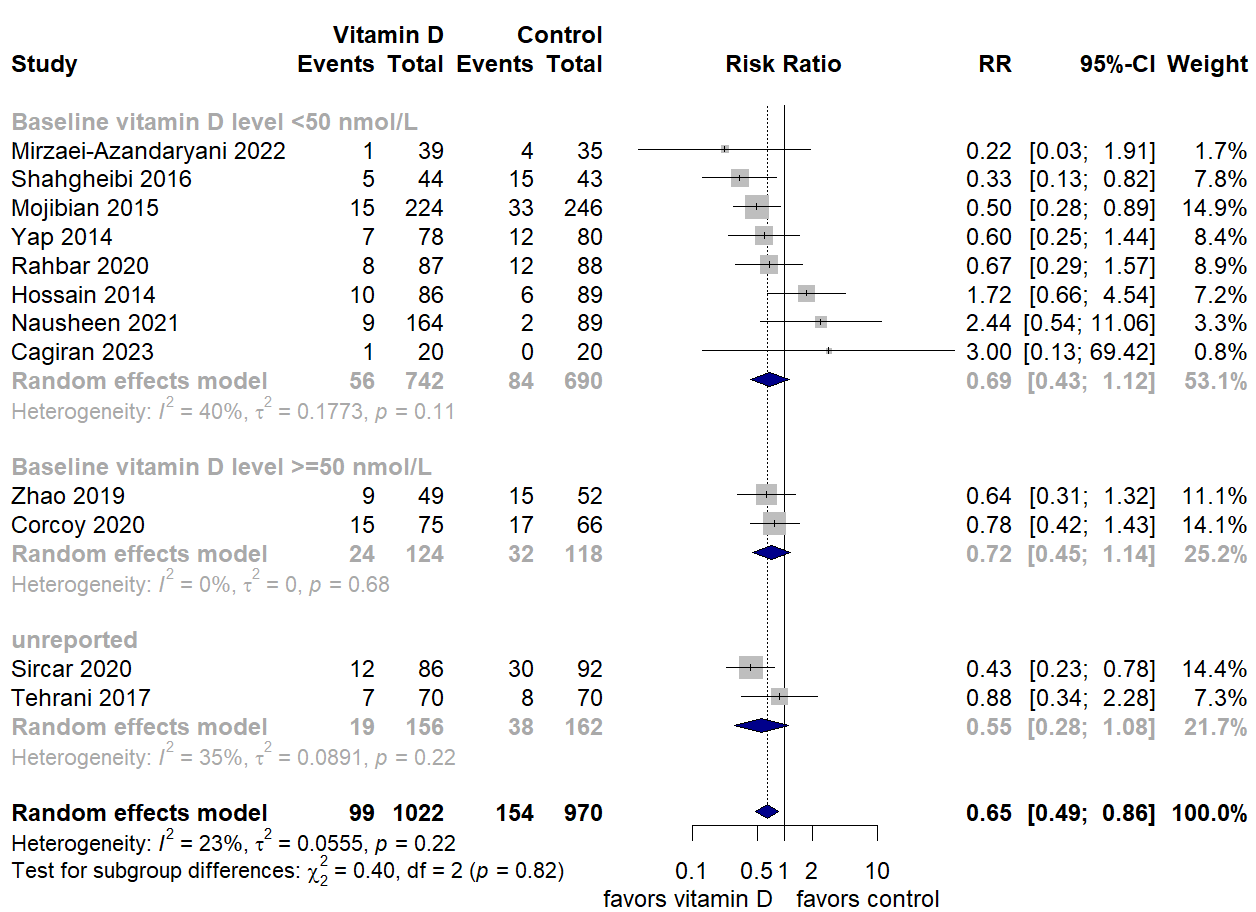


**4. Preterm labor**

**Figure S3.4.1.** Primary analysis for the risk ratio of preterm labor for women who were supplemented with vitamin D in pregnancy versus who were not

**
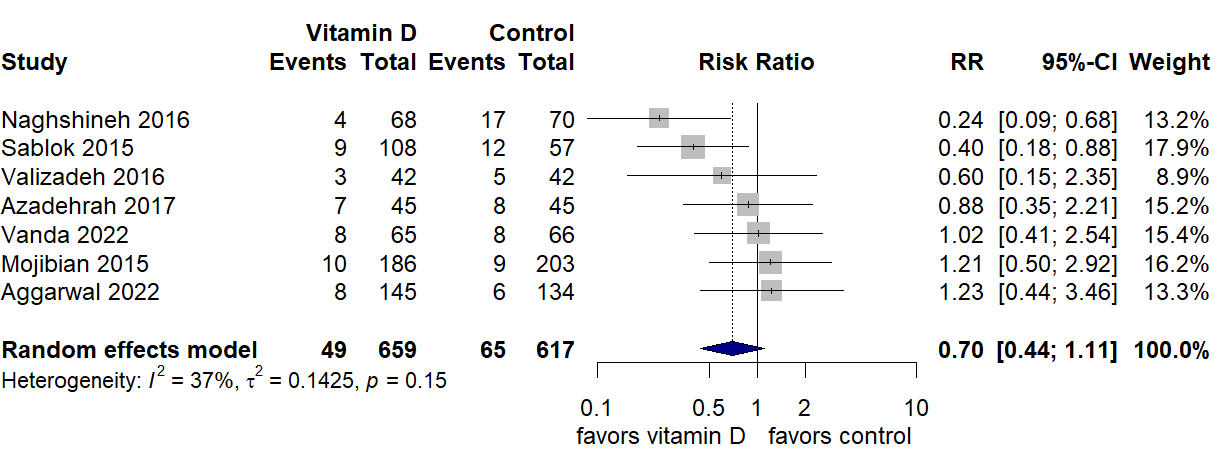
**

**Figure S3.4.2** Subgroup analysis by intervention type for the risk ratio of preterm labor for women who were supplemented with vitamin D in pregnancy versus who were not


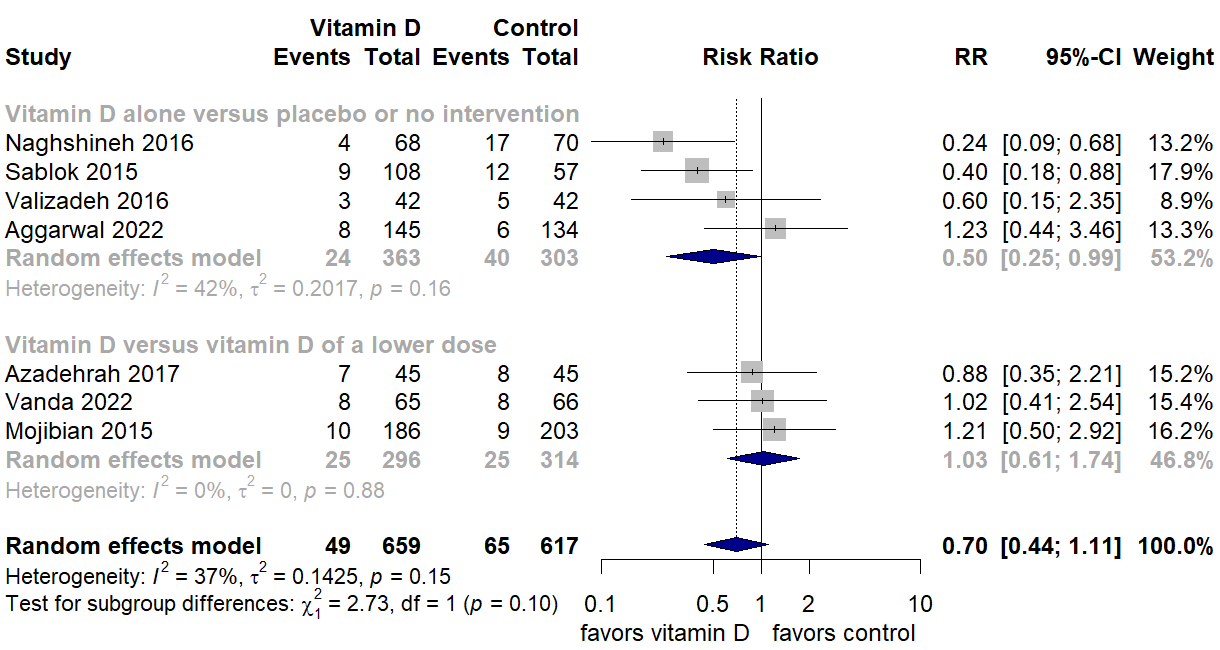


**Figure S3.4.3** Subgroup analysis by population type for the risk ratio of preterm labor for women who were supplemented with vitamin D in pregnancy versus who were not


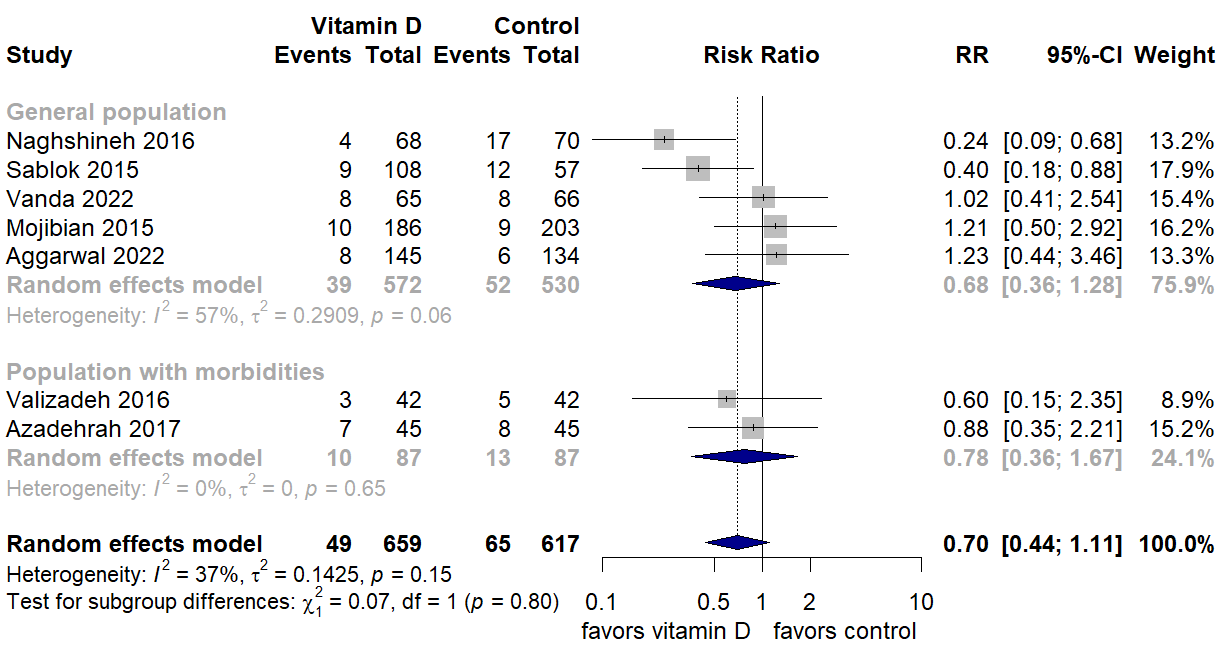


**Figure S3.4.4** Subgroup analysis by intervention dose for the risk ratio of preterm labor for women who were supplemented with vitamin D in pregnancy versus who were not


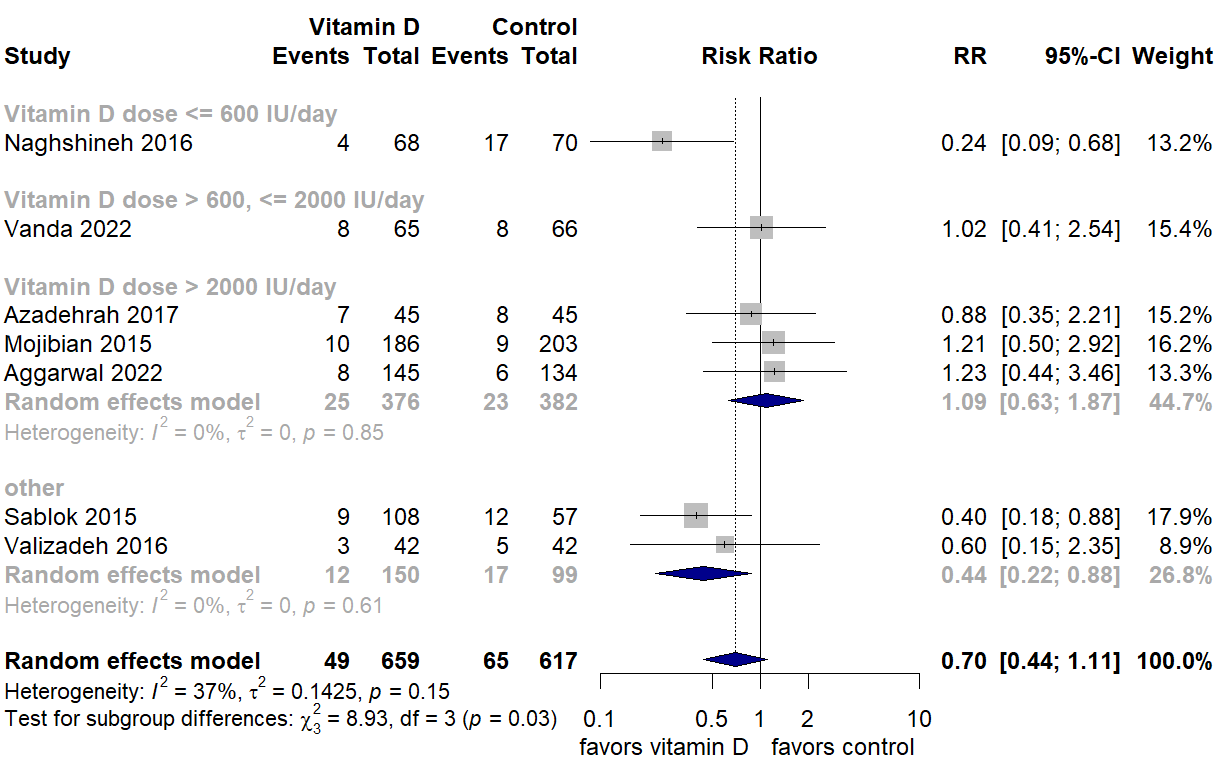


**Figure S3.4.5** Subgroup analysis by administration frequency for the risk ratio of preterm labor for women who were supplemented with vitamin D in pregnancy versus who were not


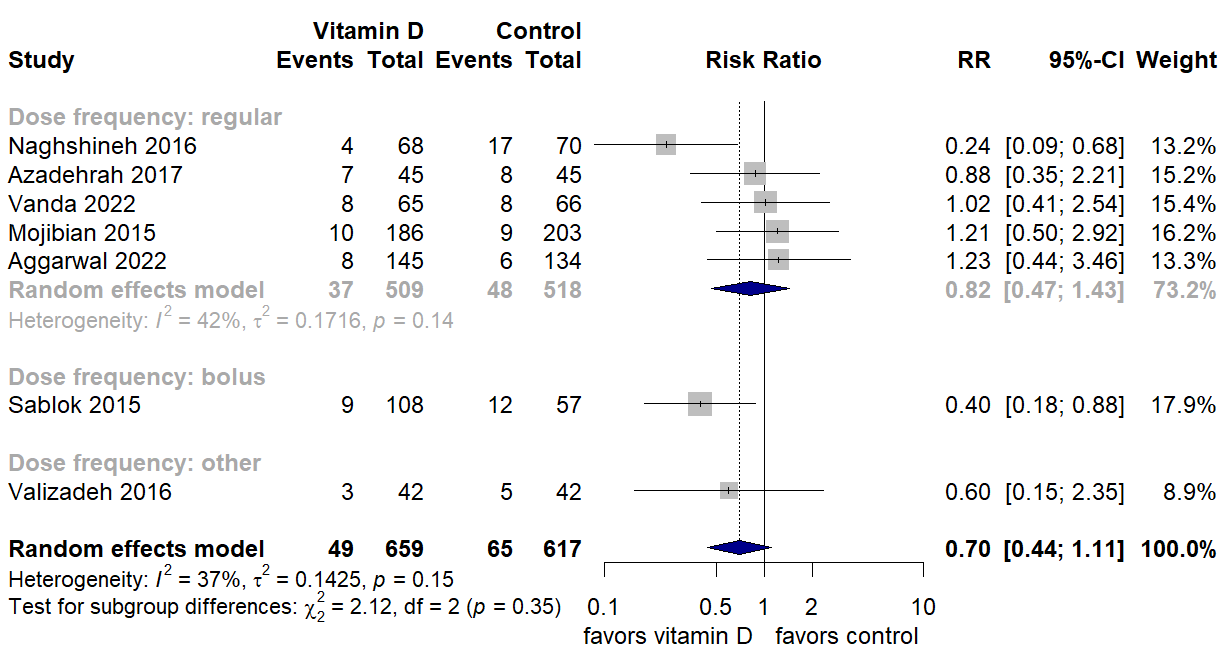


**Figure S3.4.6** Subgroup analysis by supplement form for the risk ratio of preterm labor for women who were supplemented with vitamin D in pregnancy versus who were not


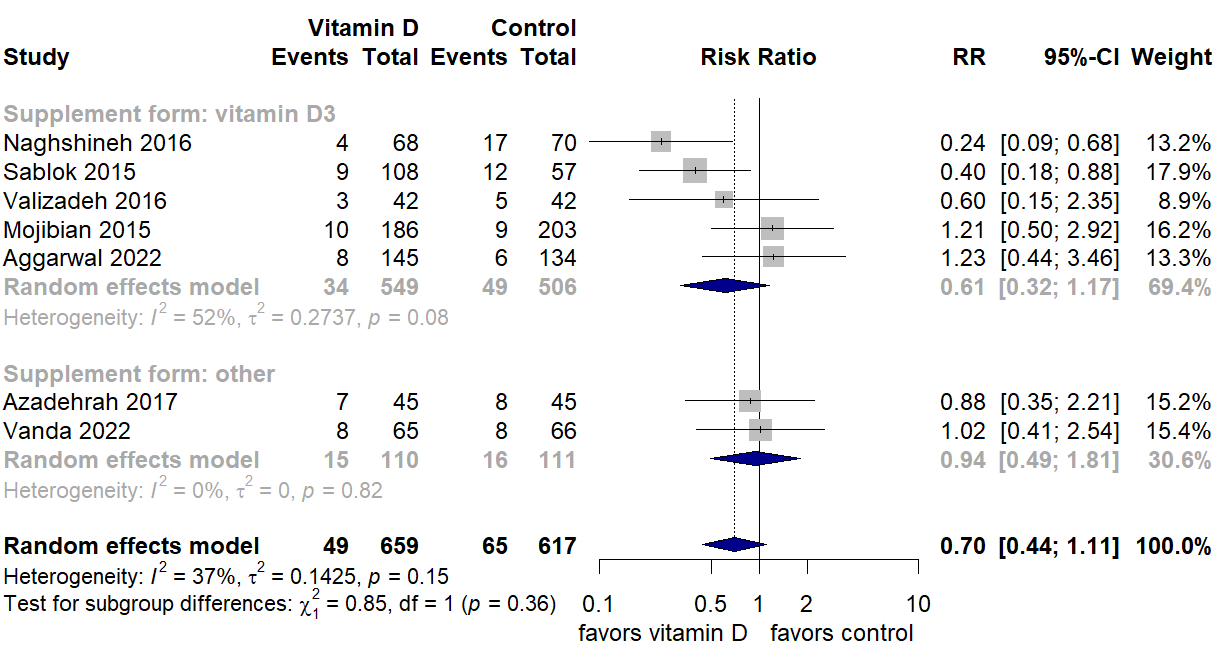


**Figure S3.4.7** Subgroup analysis by trimester of supplementation initiation for the risk ratio of preterm labor for women who were supplemented with vitamin D in pregnancy versus who were not

**
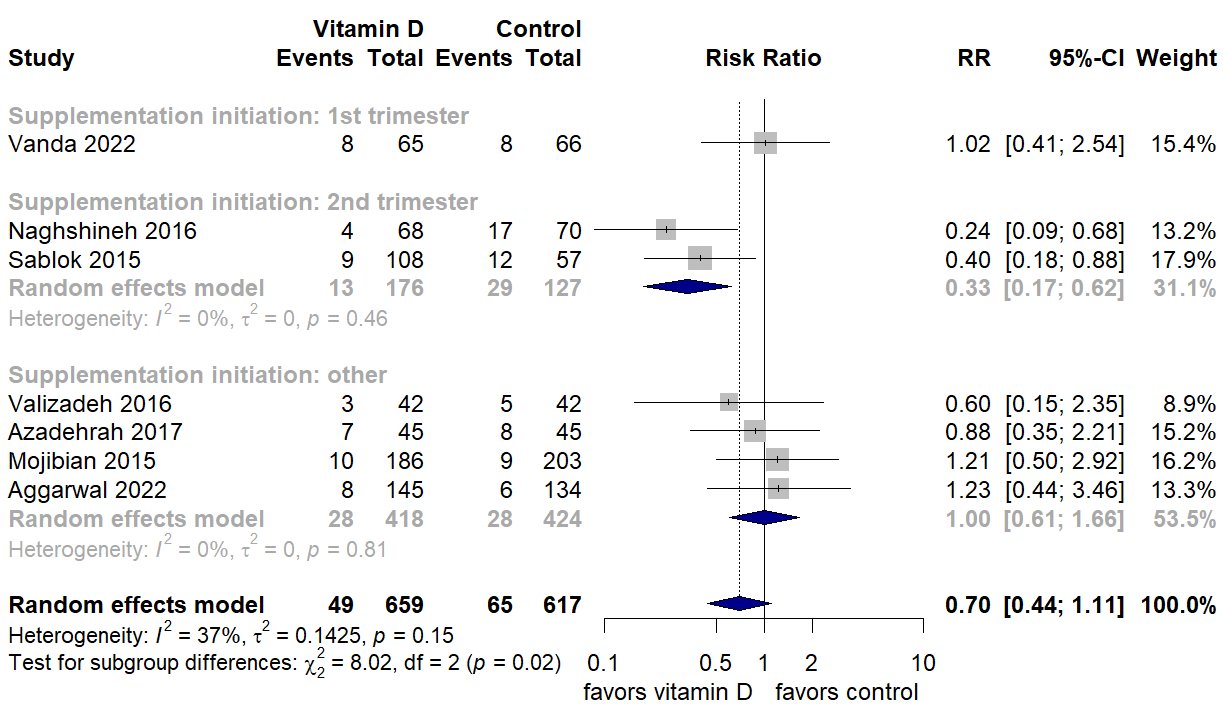
**

**Figure S3.4.8** Subgroup analysis by maternal population mean 25(OH)D concentration (30 nmol/L)for the risk ratio of preterm labor for women who were supplemented with vitamin D in pregnancy versus who were not

**
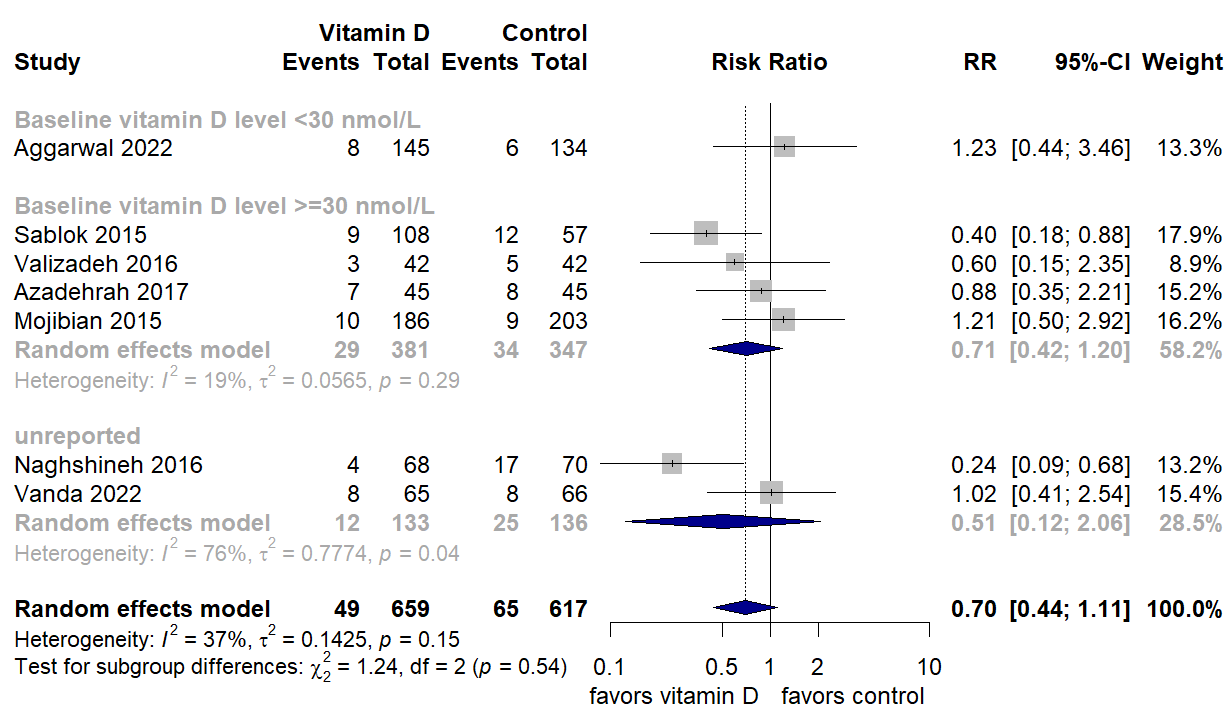
**

**Figure S3.4.9** Subgroup analysis by maternal population mean 25(OH)D concentration (50 nmol/L) for the risk ratio of preterm labor for women who were supplemented with vitamin D in pregnancy versus who were not

**
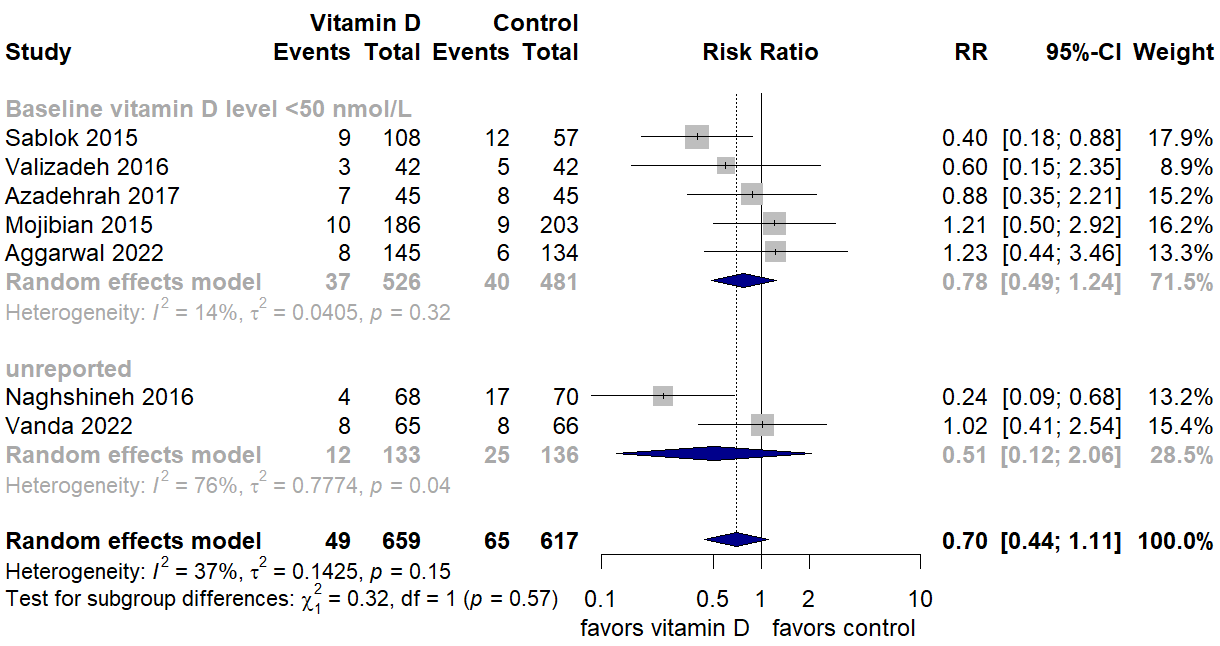
**

**5. Cesarean delivery**

**Figure S3.5.1** Primary analysis for the risk ratio of Cesarean delivery for women who were supplemented with vitamin D in pregnancy versus who were not

**
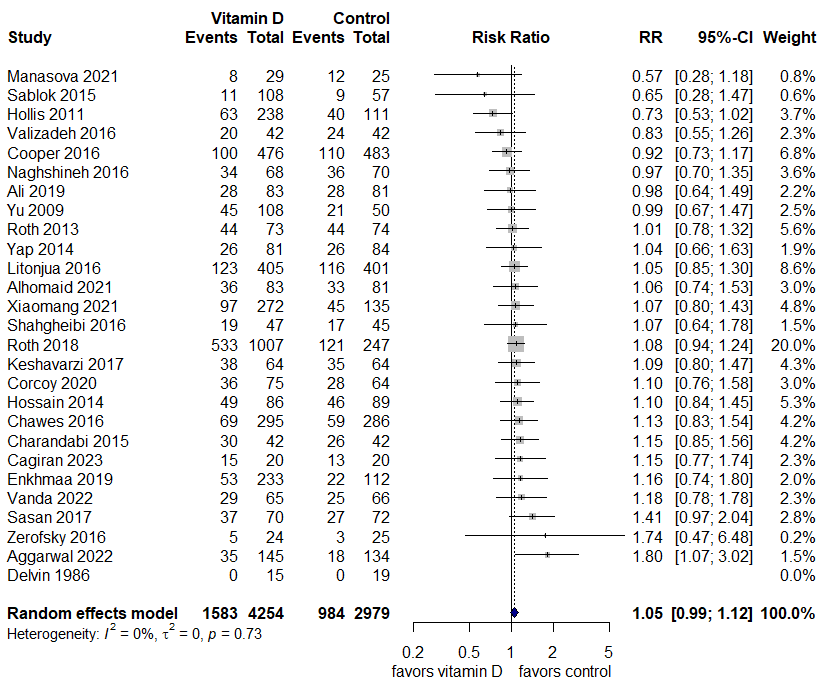
**

**Figure S3.5.2** Subgroup analysis by intervention type for the risk ratio of Cesarean delivery for women who were supplemented with vitamin D in pregnancy versus who were not


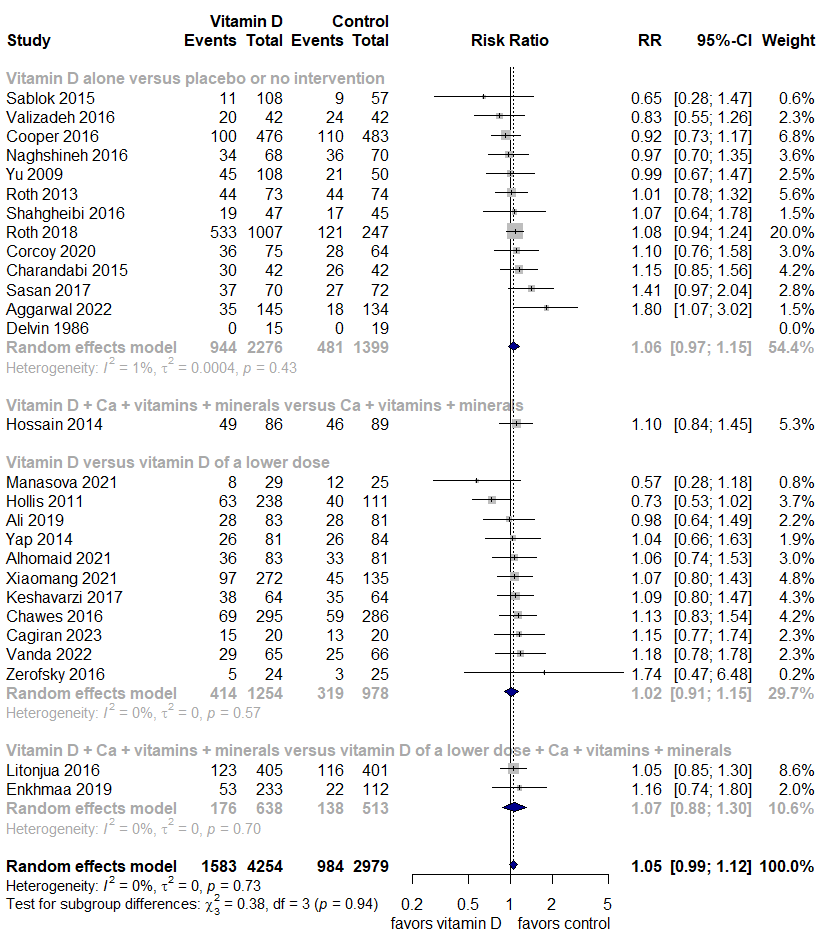


**Figure S3.5.3** Subgroup analysis by population type for the risk ratio of Cesarean delivery for women who were supplemented with vitamin D in pregnancy versus who were not


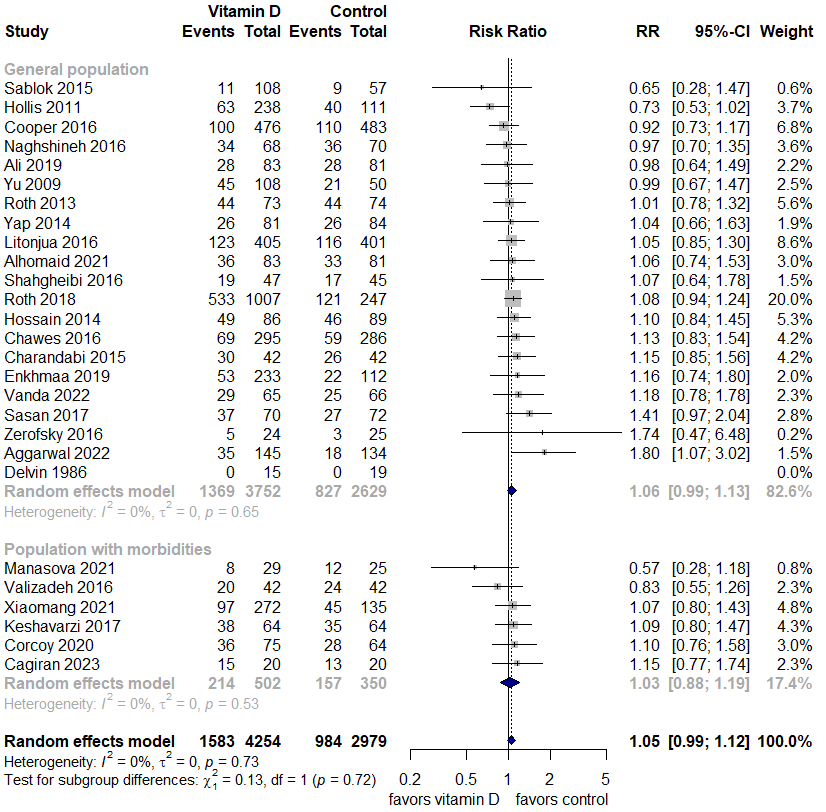


**Figure S3.5.4** Subgroup analysis by intervention dose for the risk ratio of Cesarean delivery for women who were supplemented with vitamin D in pregnancy versus who were not


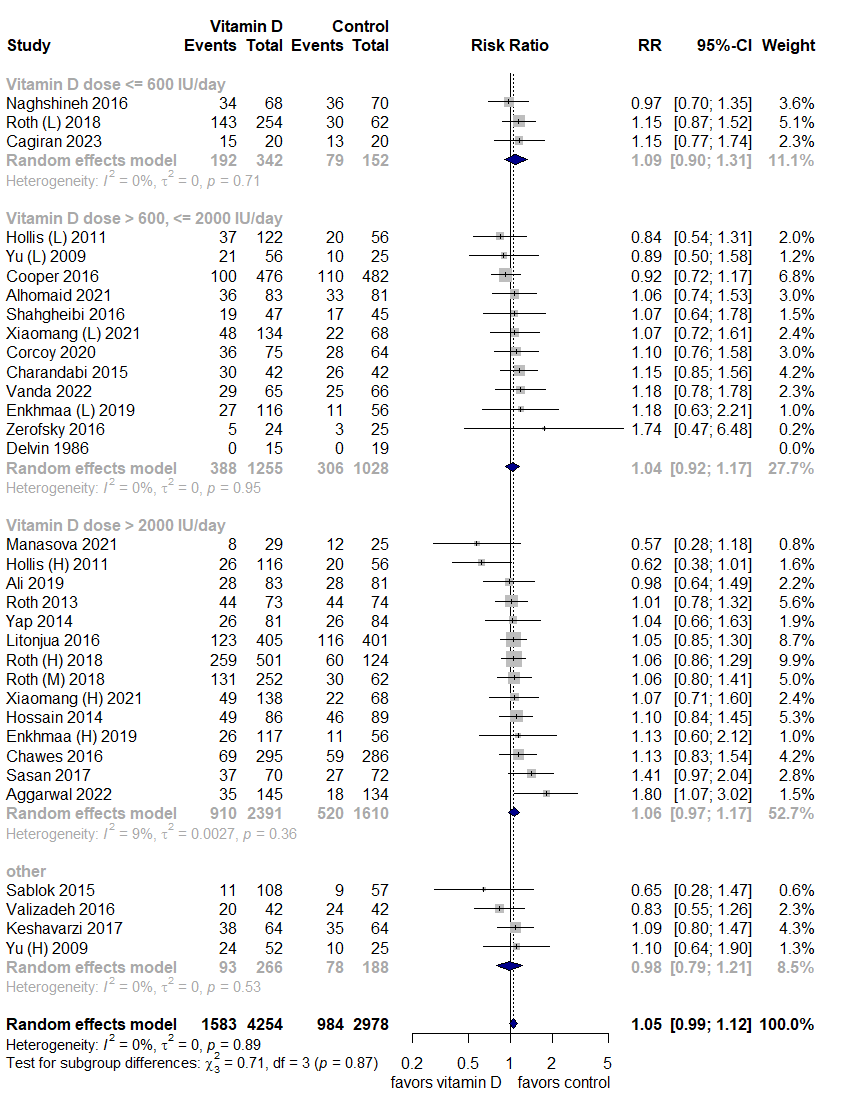


**Figure S3.5.5** Subgroup analysis by administration frequency for the risk ratio of Cesarean delivery for women who were supplemented with vitamin D in pregnancy versus who were not


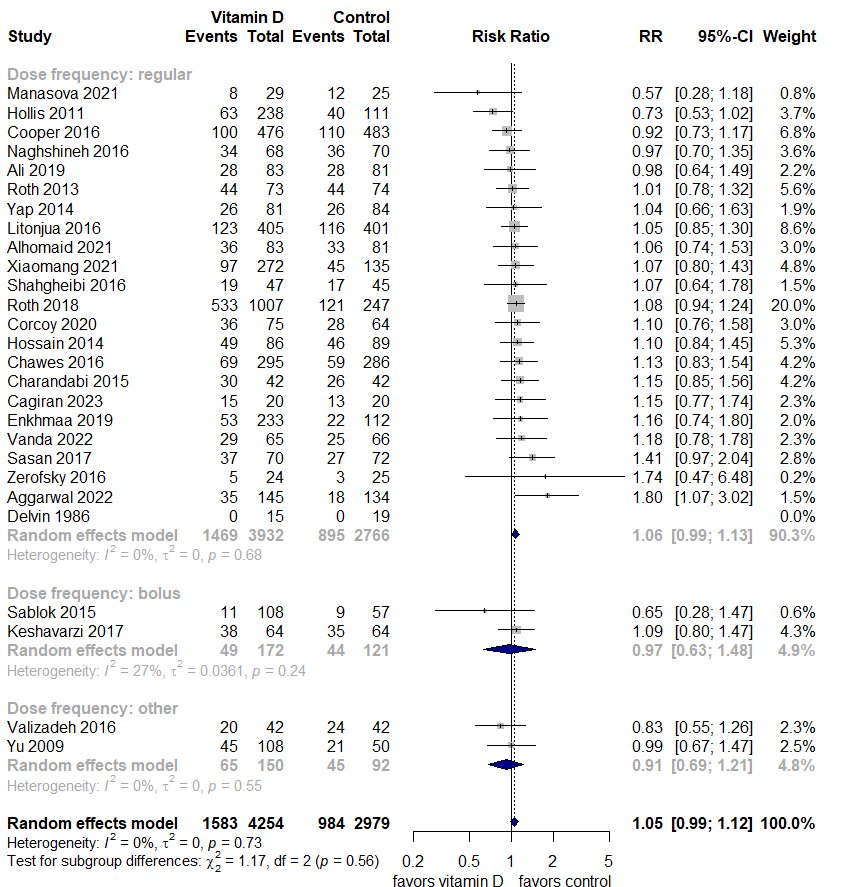


**Figure S3.5.6** Subgroup analysis by supplement form for the risk ratio of Cesarean delivery for women who were supplemented with vitamin D in pregnancy versus who were not

**
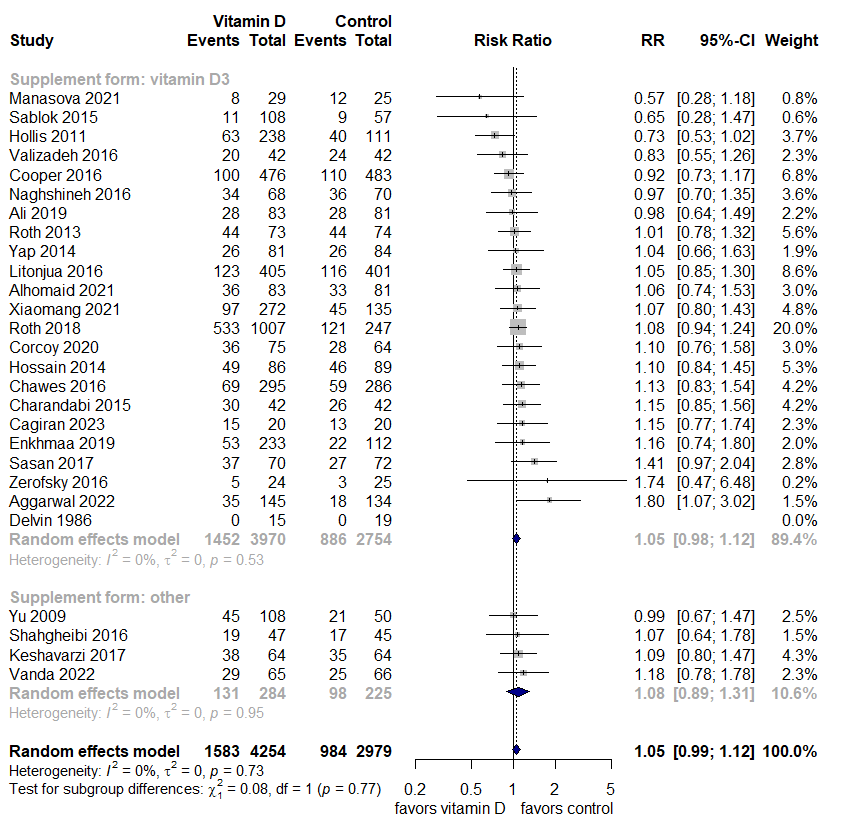
**

**Figure S3.5.7** Subgroup analysis by trimester of supplementation initiation for the risk ratio of cesarean delivery for women who were supplemented with vitamin D in pregnancy versus who were not

**
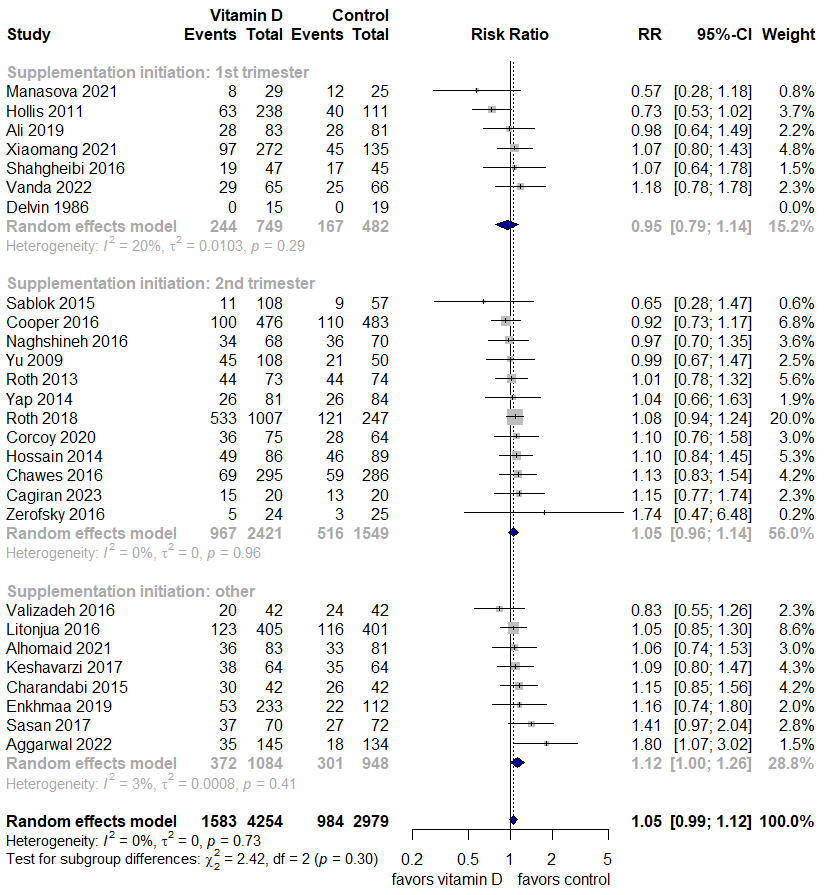
**

**Figure S3.5.8** Subgroup analysis by maternal population mean 25(OH)D concentration (30 nmol/L)for the risk ratio of Cesarean delivery for women who were supplemented with vitamin D in pregnancy versus who were not

**
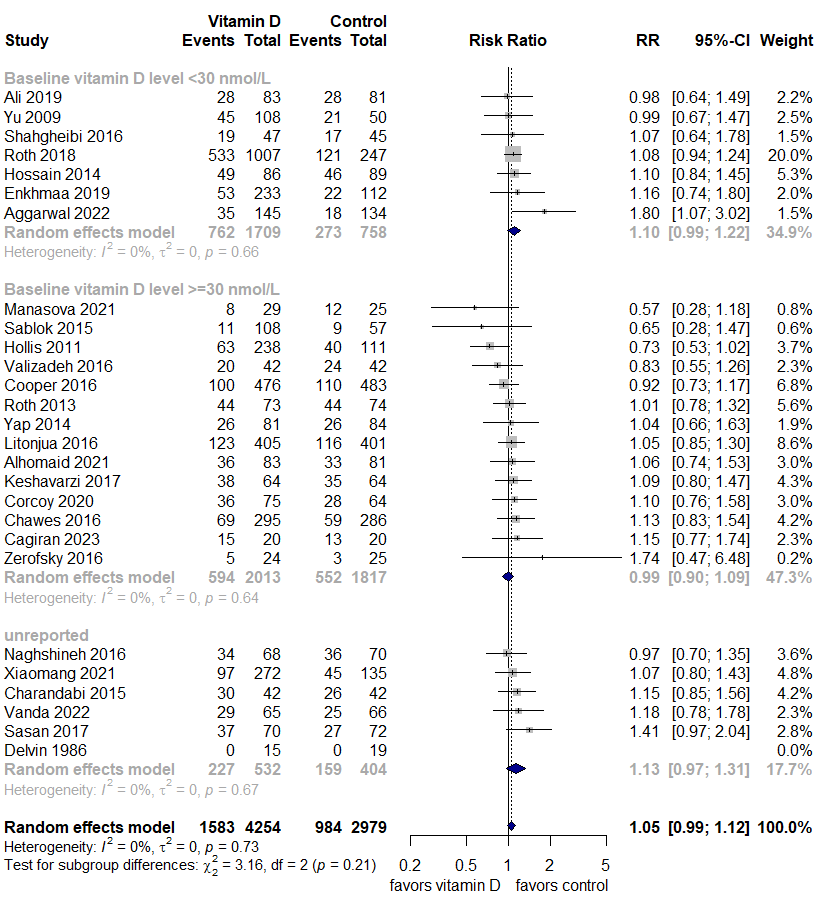
**

**Figure S3.5.9** Subgroup analysis by maternal population mean 25(OH)D concentration (50 nmol/L)of the risk ratio of Cesarean delivery for women who were supplemented with vitamin D in pregnancy versus who were not

**
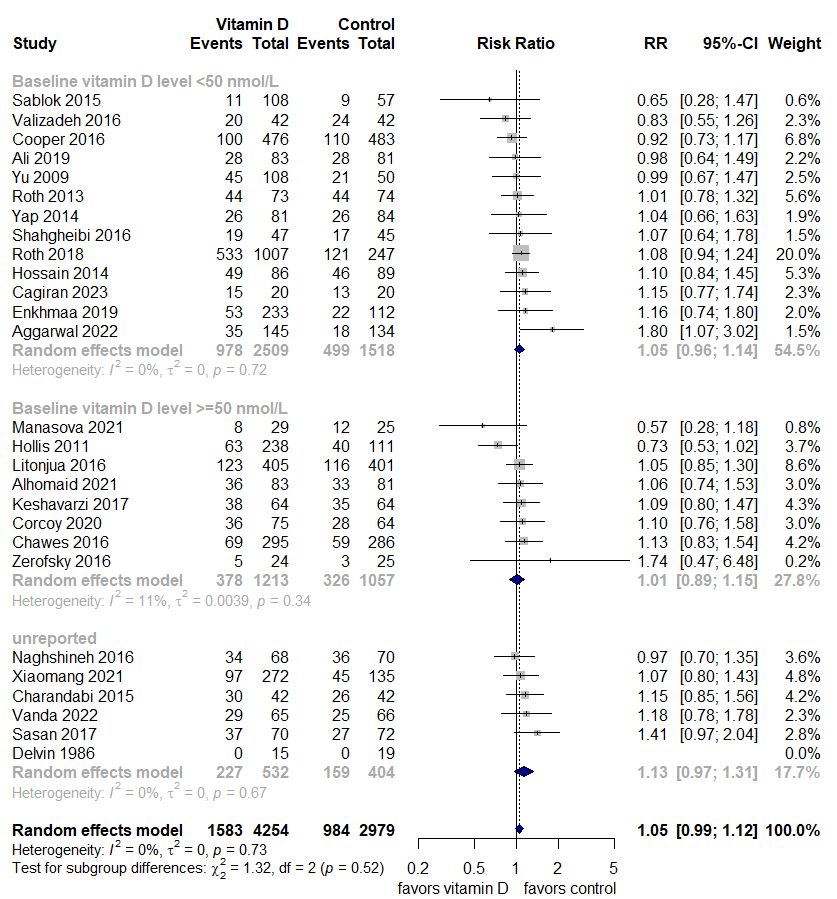
**

**6. Maternal hospitalization**

**Figure S3.6.1** Primary analysis for the risk ratio of maternal hospitalization for women who were supplemented with vitamin D in pregnancy versus who were not

**
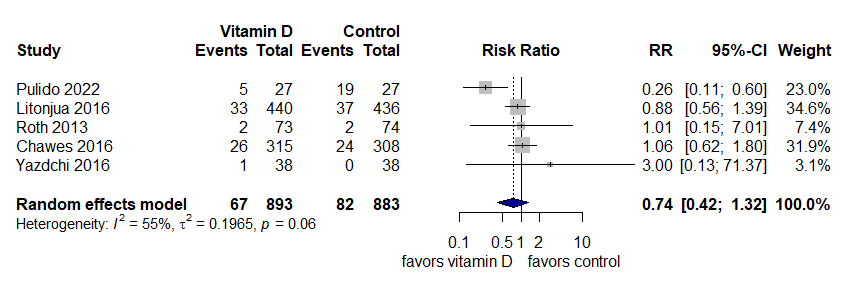
**

**Figure S3.6.2** Subgroup analysis by intervention type for the risk ratio of maternal hospitalization for women who were supplemented with vitamin D in pregnancy versus who were not


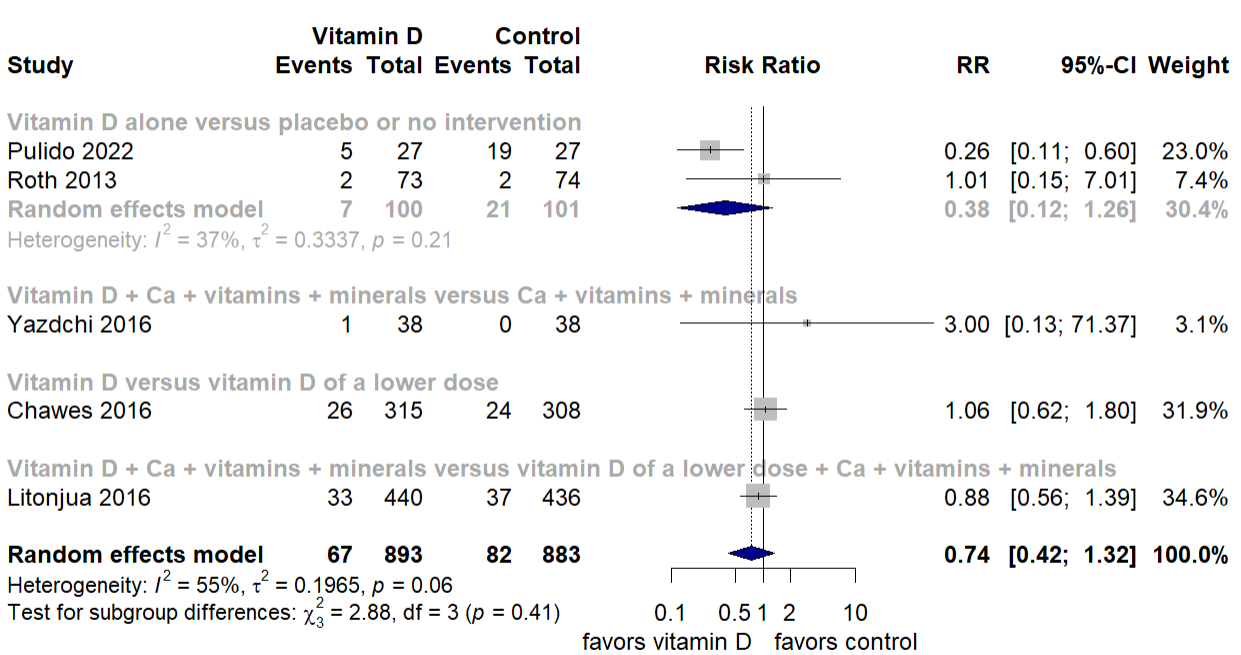


**Figure S3.6.3** Subgroup analysis by population type for the risk ratio of maternal hospitalization for women who were supplemented with vitamin D in pregnancy versus who were not


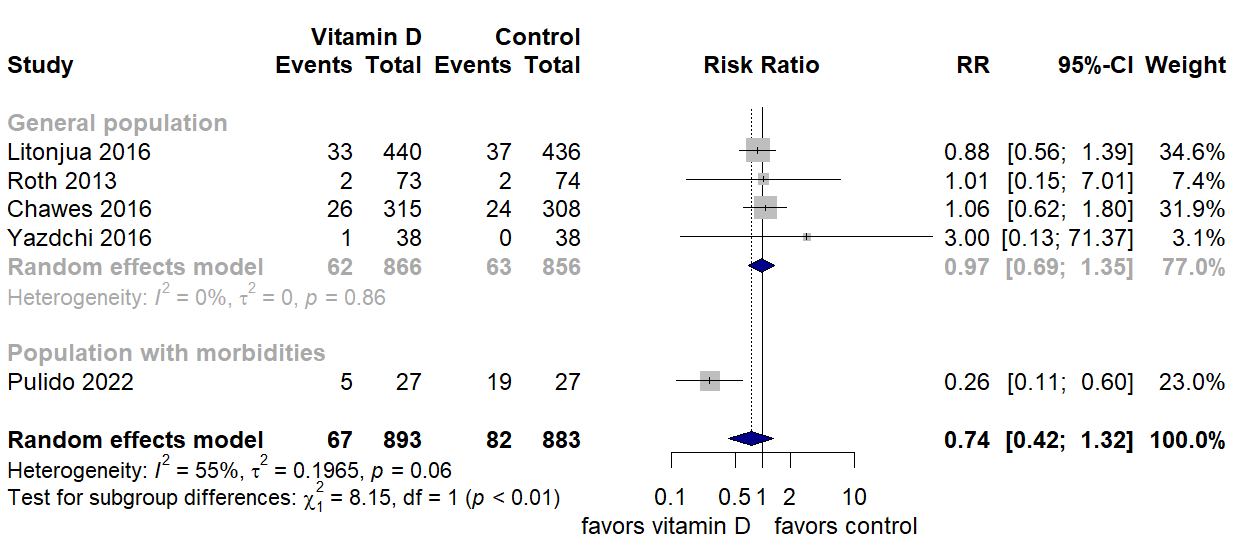


**Figure S3.6.4** Subgroup analysis by intervention dose for the risk ratio of maternal hospitalization for women who were supplemented with vitamin D in pregnancy versus who were not


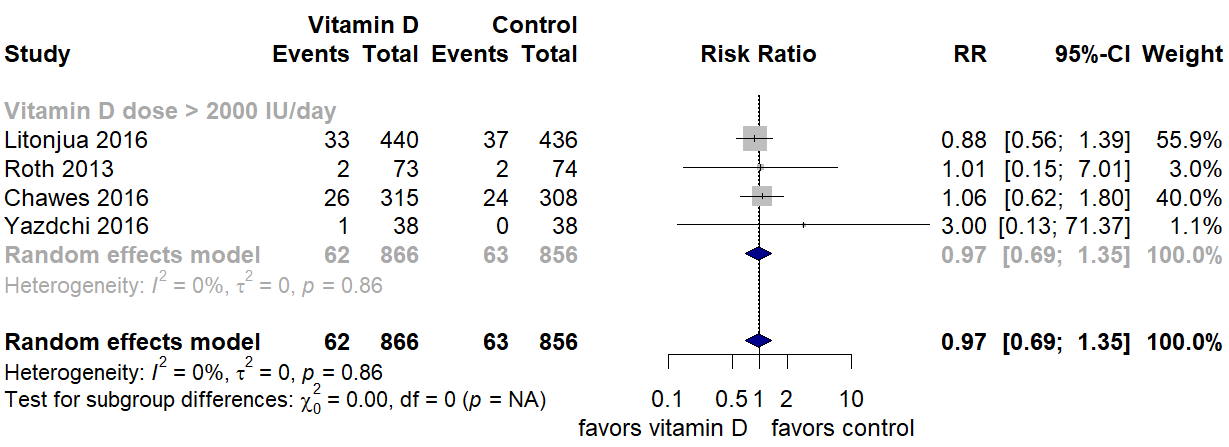


**Figure S3.6.5** Subgroup analysis by administration frequency for the risk ratio of maternal hospitalization for women who were supplemented with vitamin D in pregnancy versus who were not


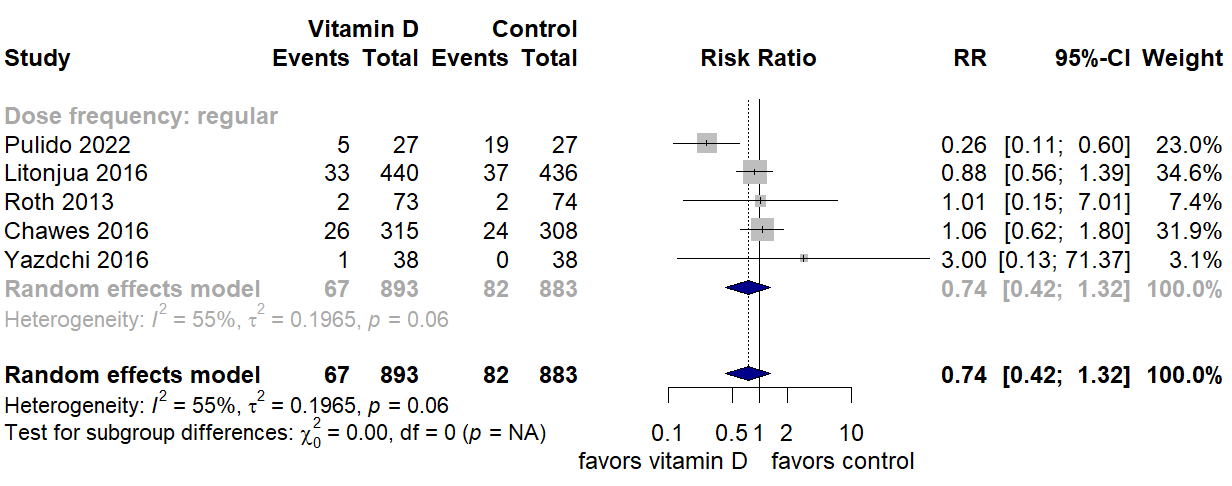


**Figure S3.6.6** Subgroup analysis by supplement form for the risk ratio of maternal hospitalization for women who were supplemented with vitamin D in pregnancy versus who were not

**
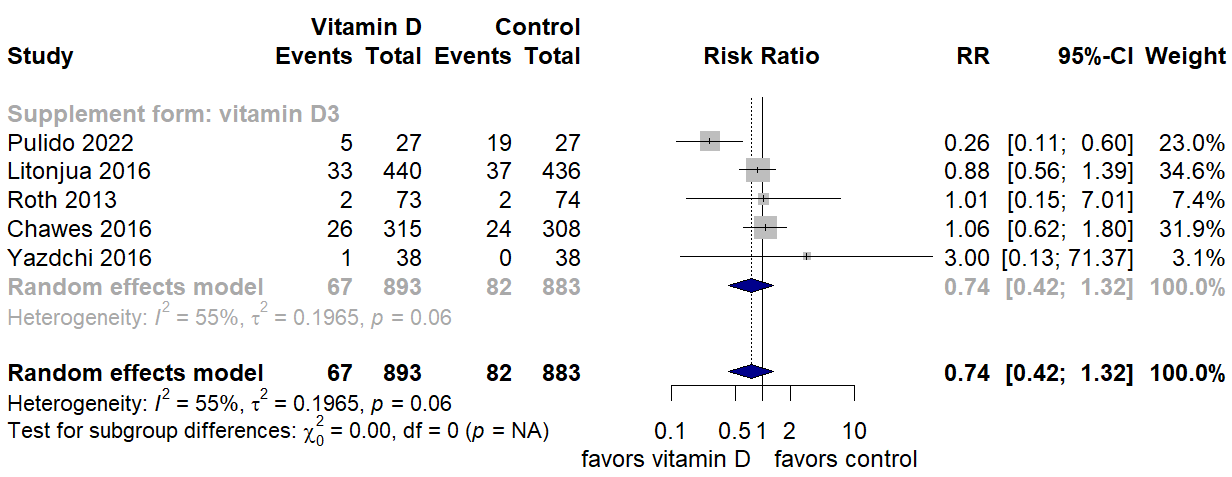
**

**Figure S3.6.7** Subgroup analysis by trimester of supplementation initiation for the risk ratio of maternal hospitalization for women who were supplemented with vitamin D in pregnancy versus who were not

**
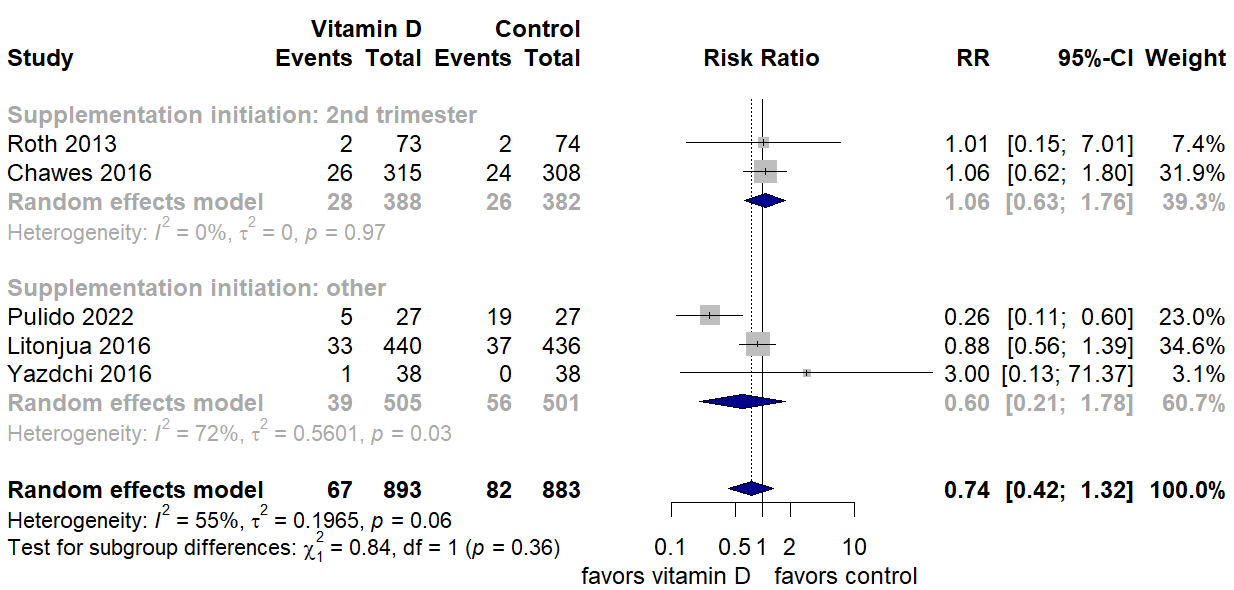
**

**Figure S3.6.8** Subgroup analysis by maternal population mean 25(OH)D concentration (30 nmol/L) for the risk ratio of maternal hospitalization for women who were supplemented with vitamin D in pregnancy versus who were not

**
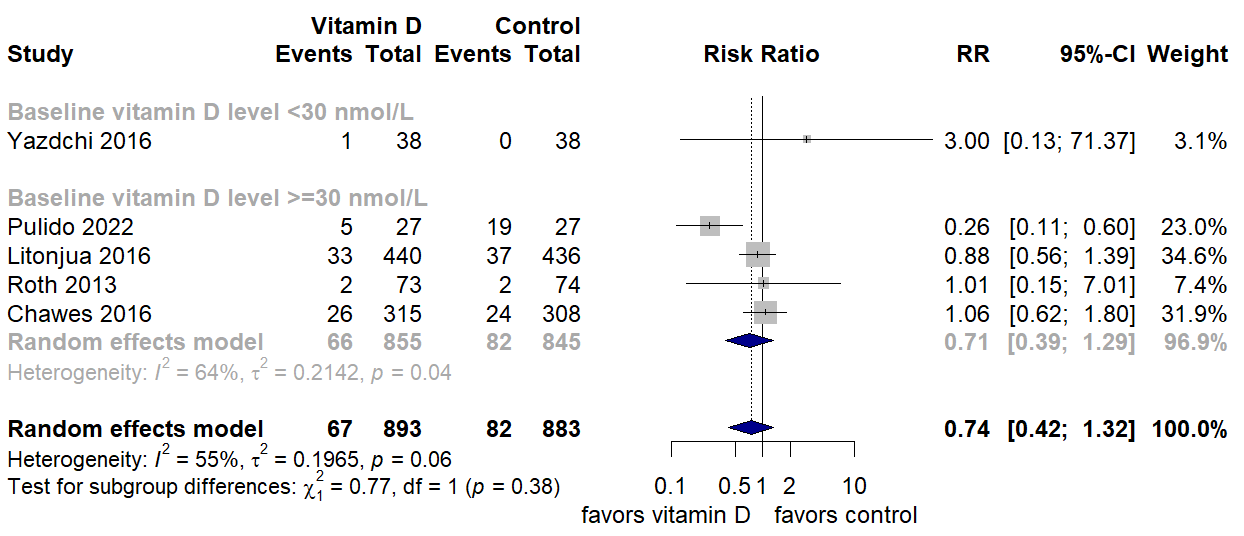
**

**Figure S3.6.9** Subgroup analysis by maternal population mean 25(OH)D concentration (50 nmol/L) of the risk ratio of maternal hospitalization for women who were supplemented with vitamin D in pregnancy versus who were not

**
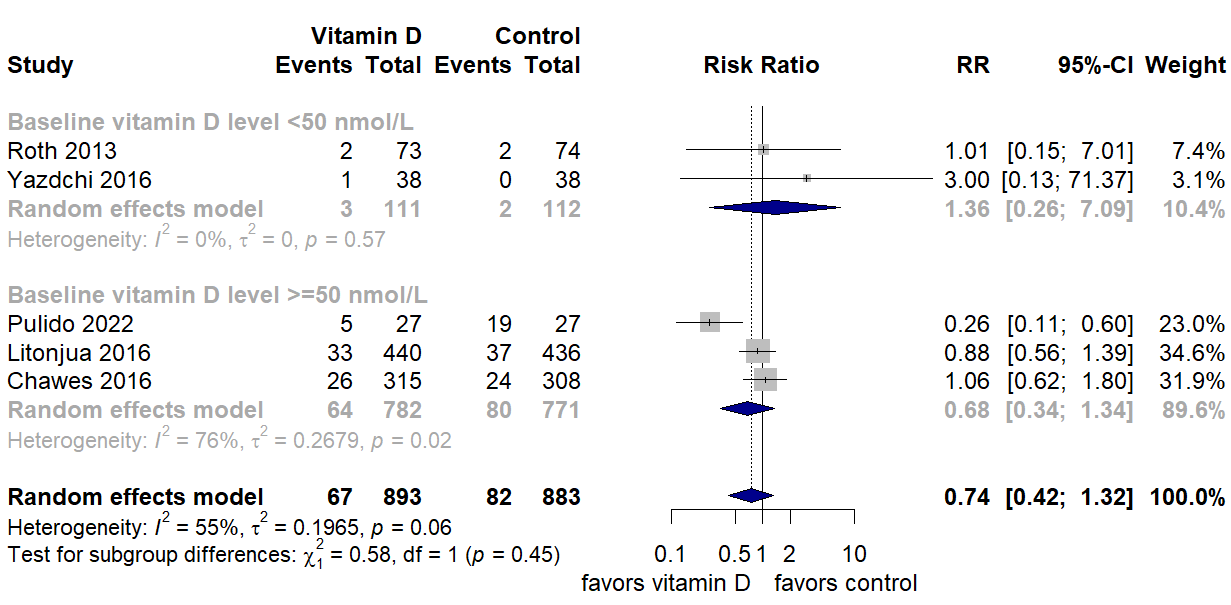
**

**7. Maternal hypercalcemia**

**Figure S3.7.1** Primary analysis for the risk ratio of maternal hypercalcemia for women who were supplemented with vitamin D in pregnancy versus who were not who were not


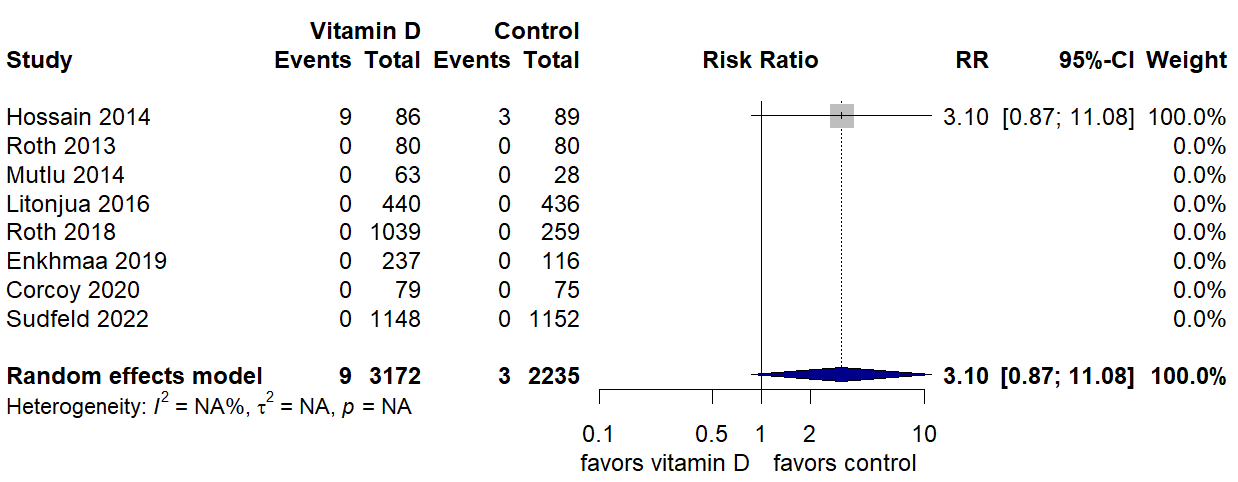


**Figure S3.7.2** Sensitivity analysis for the risk ratio of maternal hypercalcemia for women who were supplemented with vitamin D in pregnancy versus who were not

**
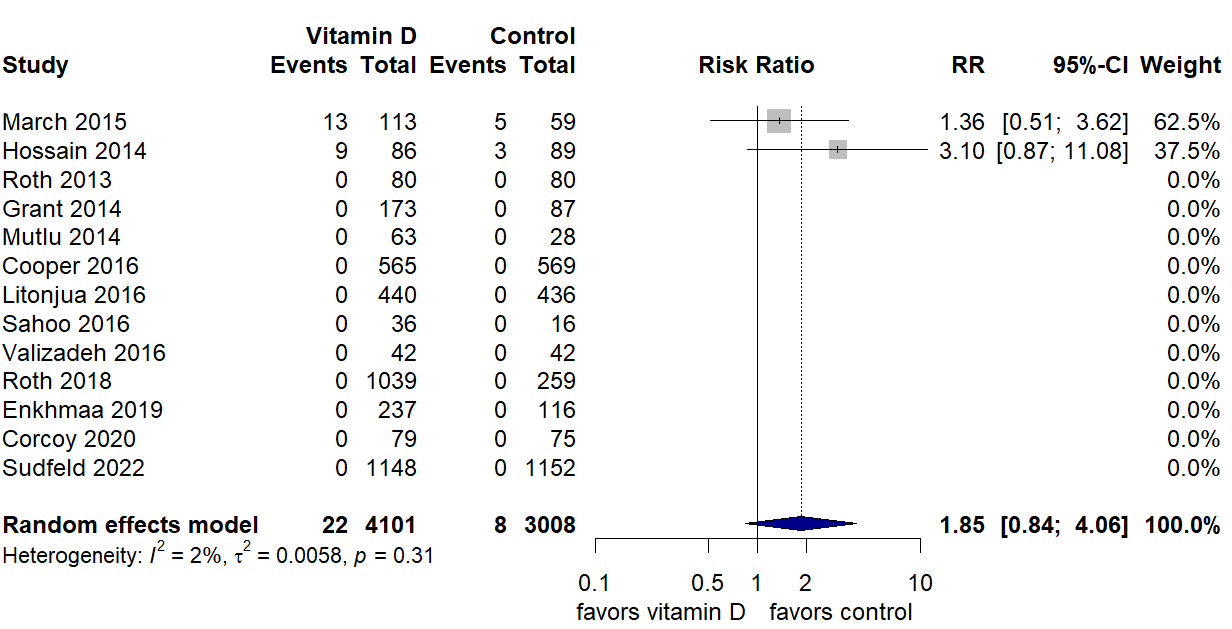
**

**8. Maternal hypocalcemia**

**Figure S3.8.1** Primary analysis for the risk ratio of maternal hypocalcemia for women who were supplemented with vitamin D in pregnancy versus who were not

**
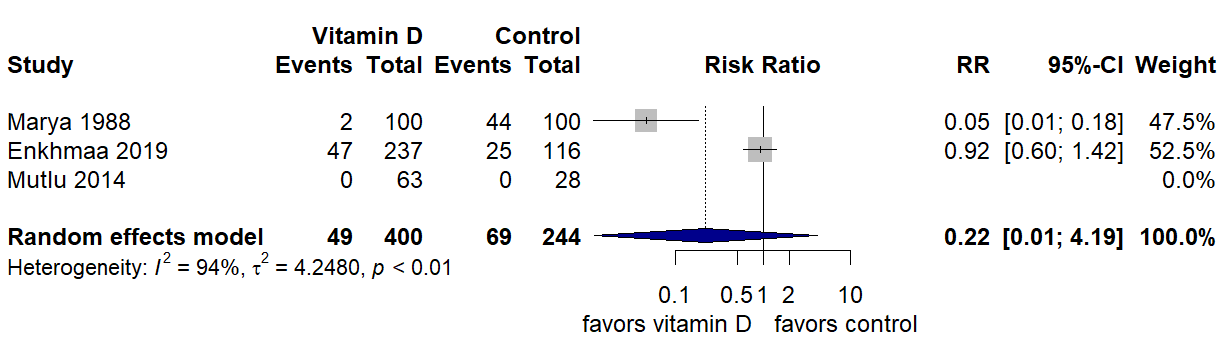
**

**Figure S3.8.2** Sensitivity analysis for the risk ratio of maternal hypocalcemia for women who were supplemented with vitamin D in pregnancy versus who were not


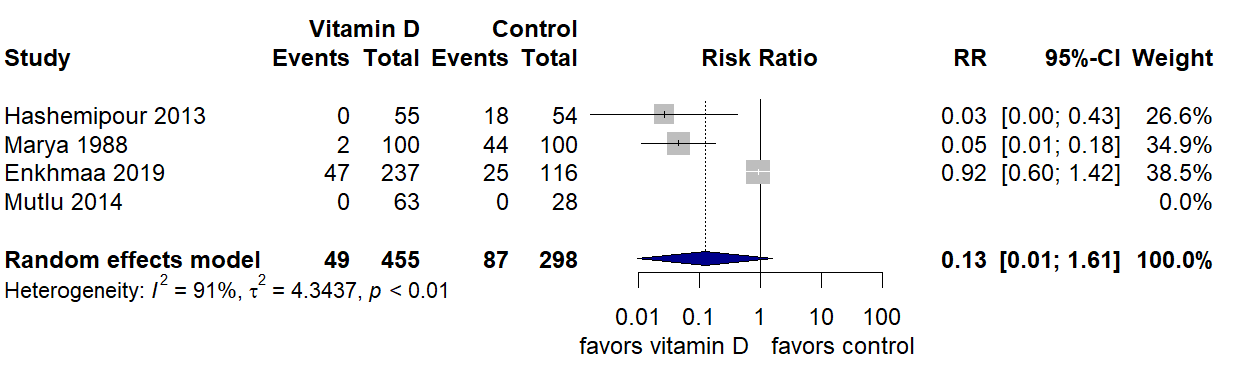


**9. Maternal hypercalciuria**

**Figure S3.9.1** Primary analysis for the risk ratio of maternal hypercalciuria for women who were supplemented with vitamin D in pregnancy versus who were not

**
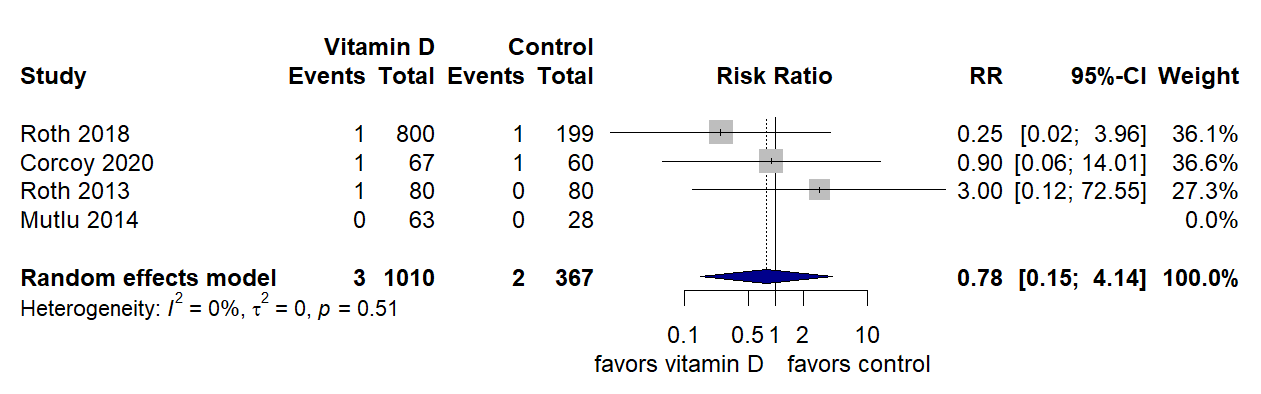
**

**Figure S3.9.2** Sensitivity analysis for the risk ratio of maternal hypercalciuria for women who were supplemented with vitamin D in pregnancy versus who were not

**
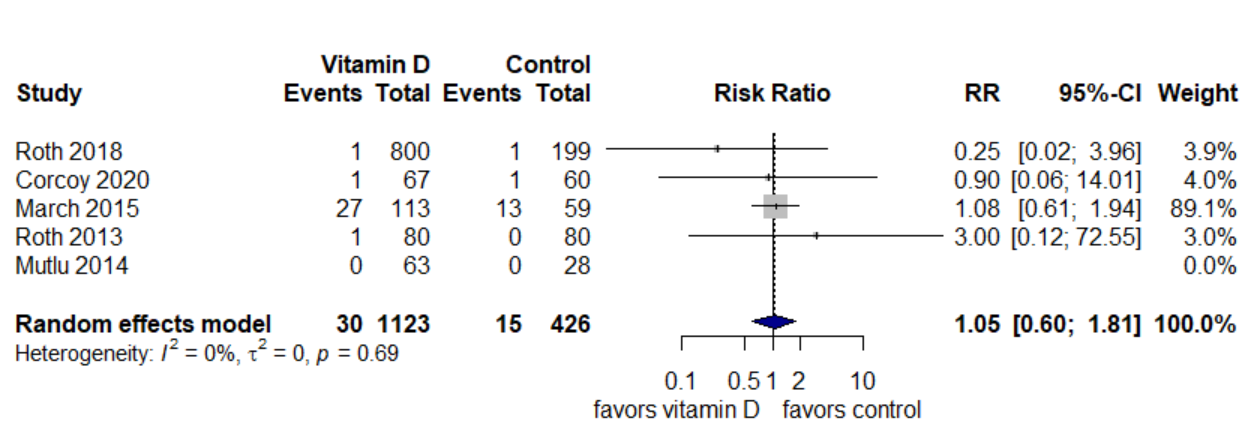
**

**10. Maternal 25(OH)D concentration at or near delivery (nmol/L)**

**Figure S3.10.1** Primary analysis for the mean difference of the mean difference of maternal 25(OH)D concentration at or near delivery among women who were supplemented with vitamin D in pregnancy versus who were not

**
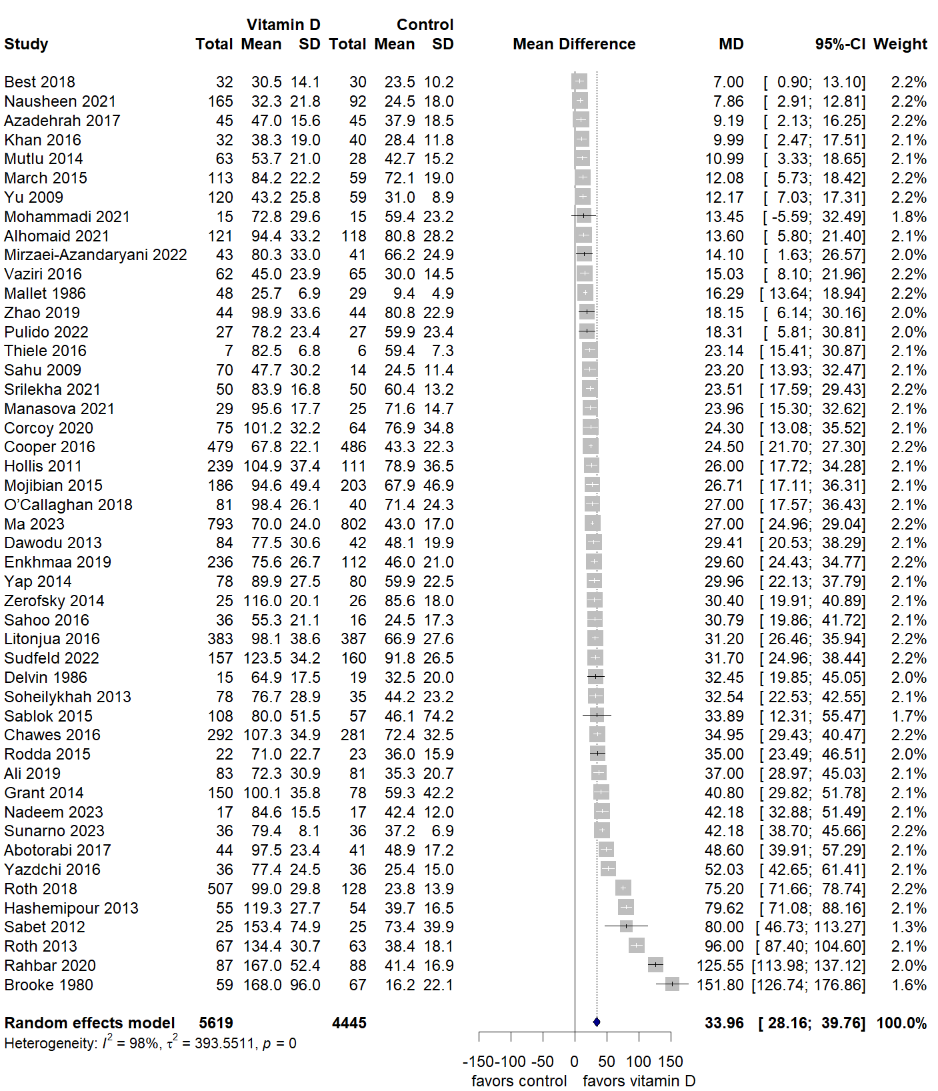
**

**Figure S3.10.2** Subgroup analysis by intervention type for the mean difference of maternal 25(OH)D concentration at or near delivery among women who were supplemented with vitamin D in pregnancy versus who were not


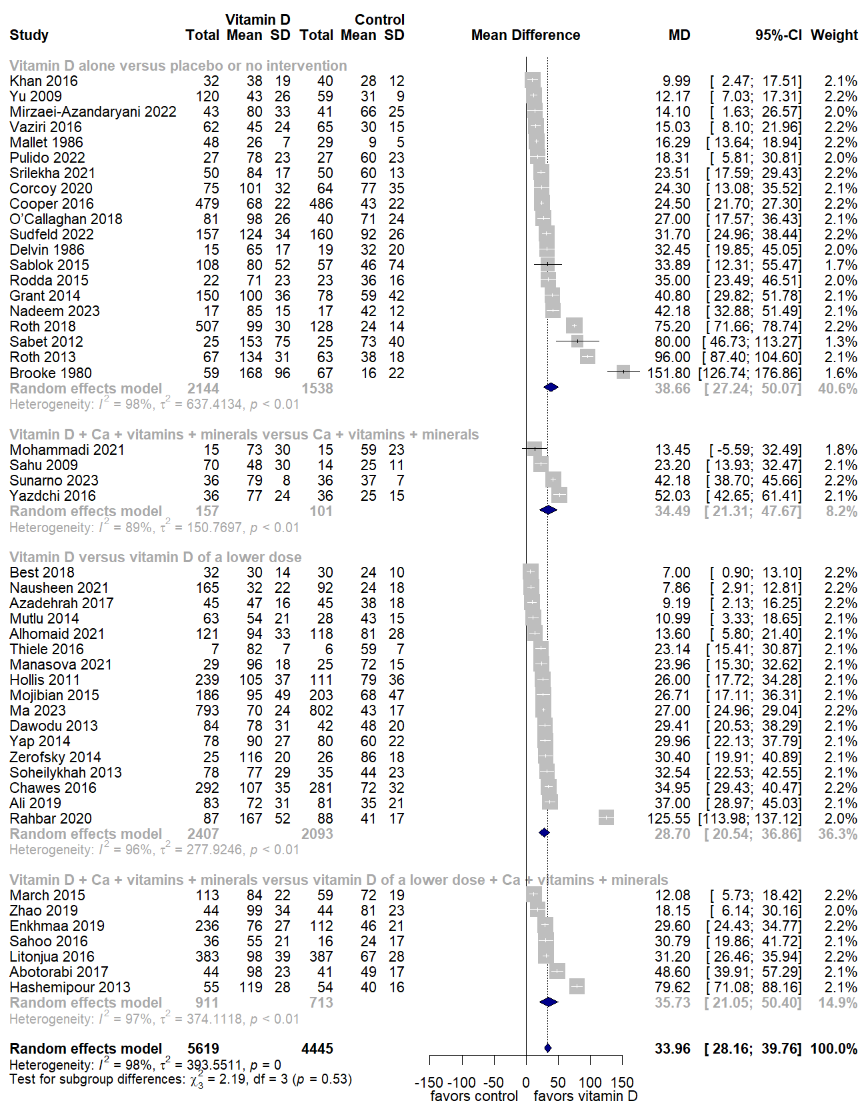


**Figure S3.10.3** Subgroup analysis by population type for the mean difference of maternal 25(OH)D concentration at or near delivery among women who were supplemented with vitamin D in pregnancy versus who were not


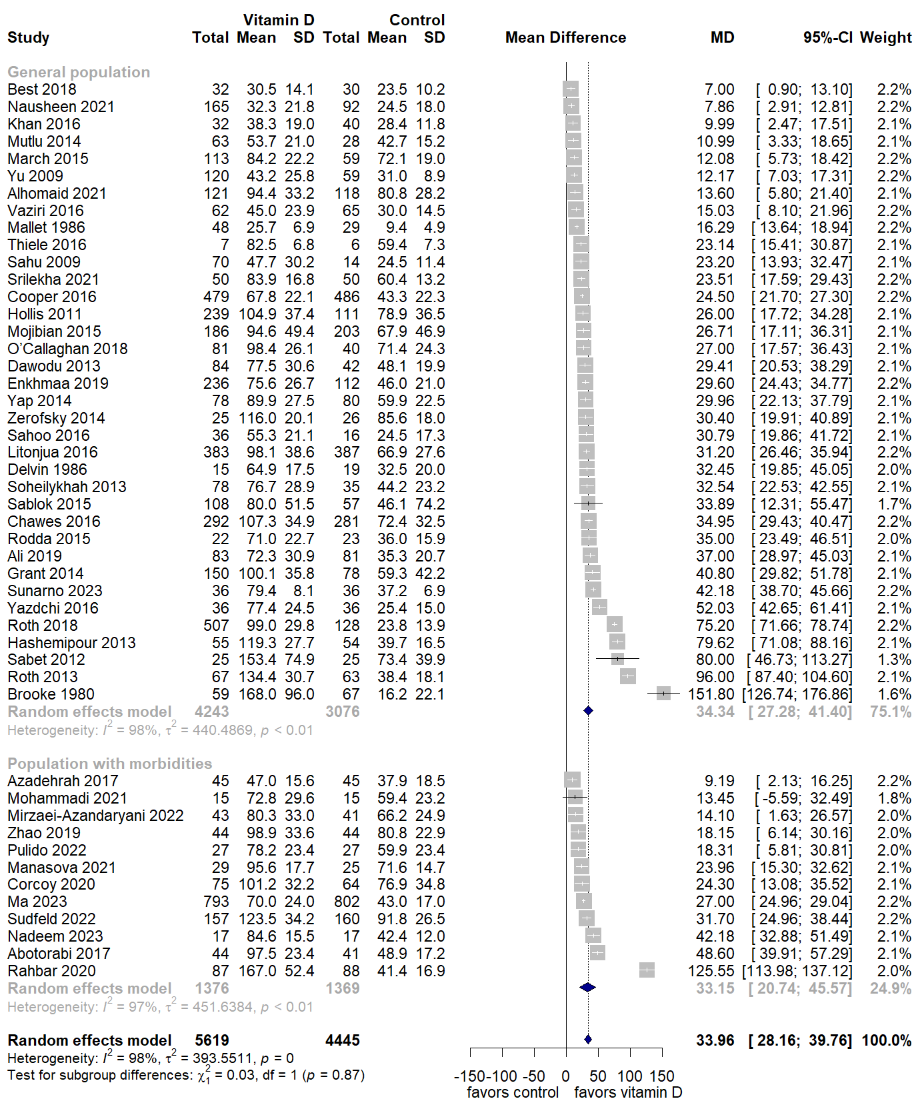


**Figure S3.10.4** Subgroup analysis by intervention dose for the mean difference of maternal 25(OH)D concentration at or near delivery among women who were supplemented with vitamin D in pregnancy versus who were not


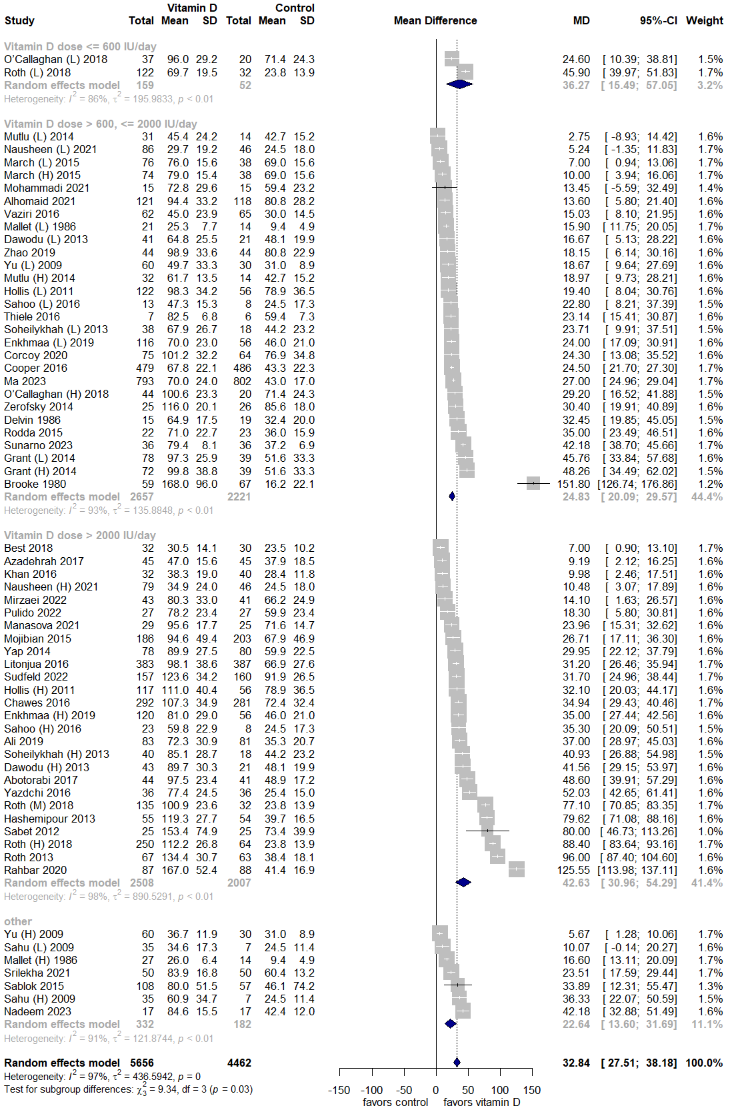


**Figure S3.10.5** Subgroup analysis by administration frequency for the mean difference of maternal 25(OH)D concentration at or near delivery among women who were supplemented with vitamin D in pregnancy versus who were not
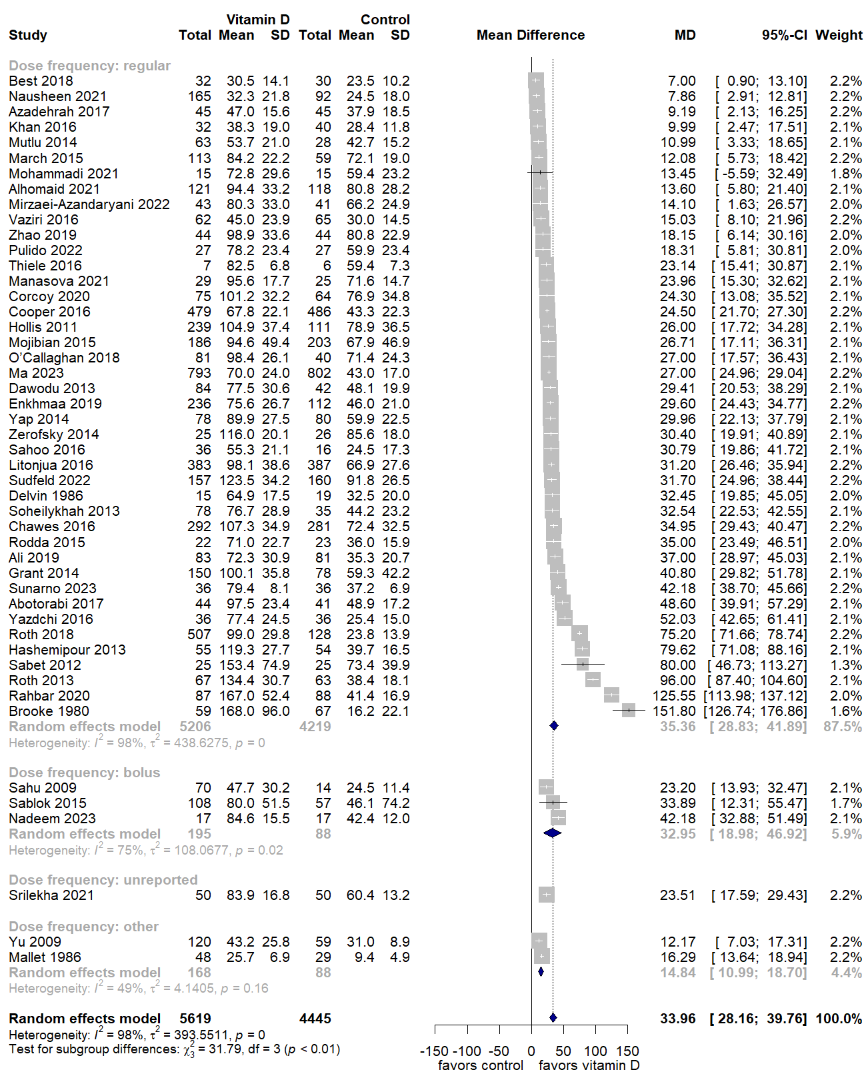


**Figure S3.10.6** Subgroup analysis by supplement form for the mean difference of maternal 25(OH)D concentration at or near delivery among women who were supplemented with vitamin D in pregnancy versus who were not

**
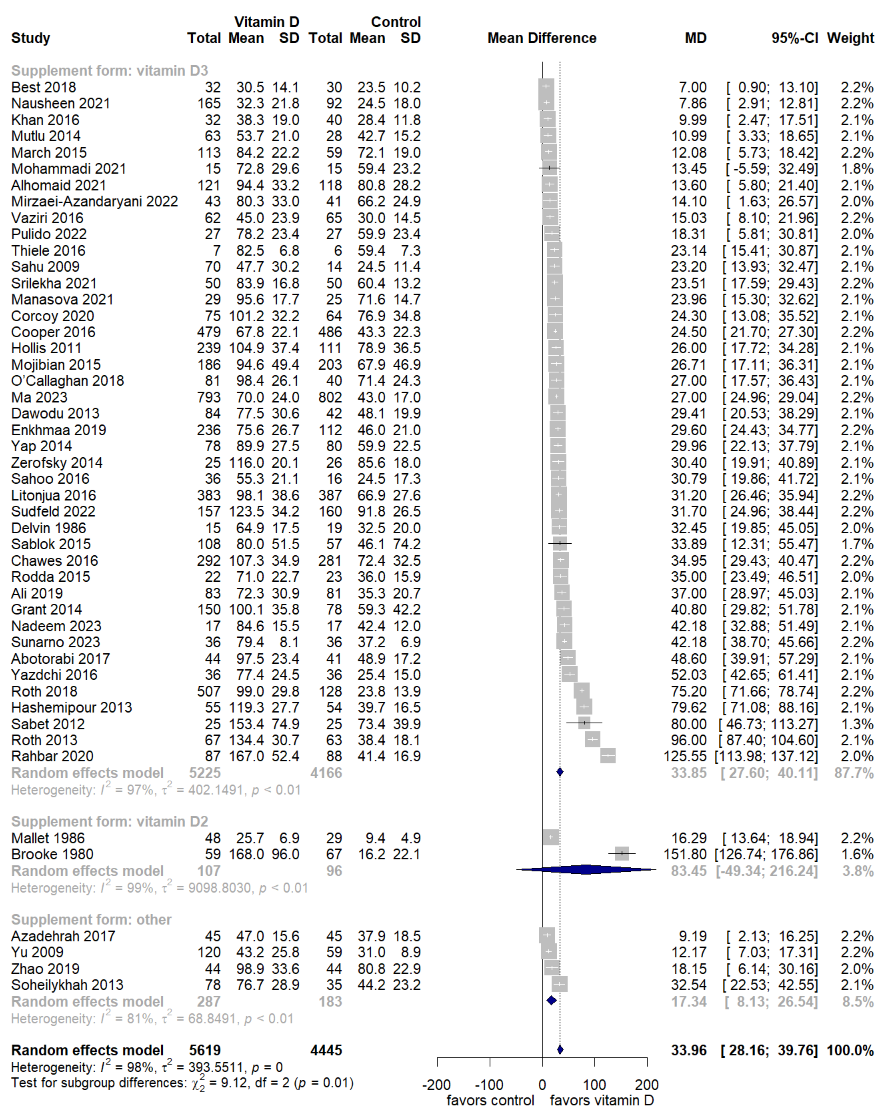
**

**Figure S3.10.7** Subgroup analysis by trimester of supplementation initiation for the mean difference of maternal 25(OH)D concentration at or near delivery among women who were supplemented with vitamin D in pregnancy versus who were not


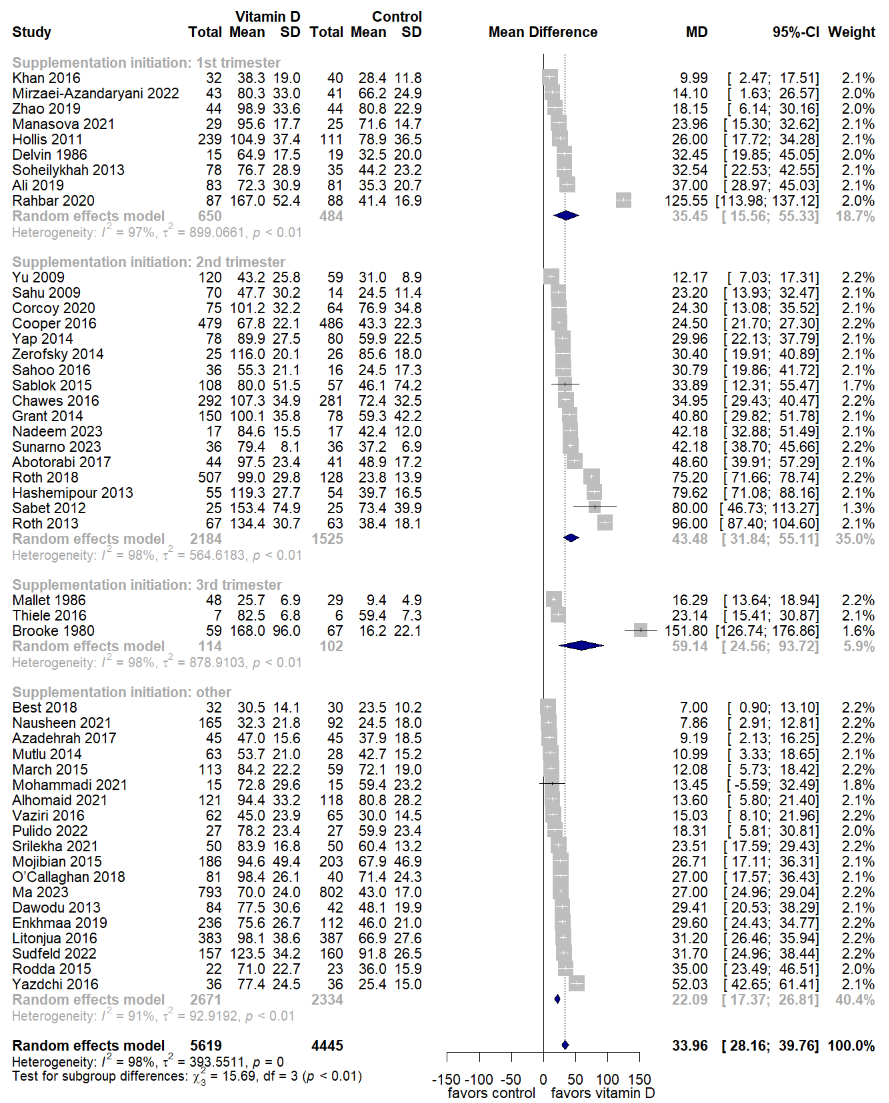


**Figure S3.10.8** Subgroup analysis by maternal population mean 25(OH)D concentration (30 nmol/L) for the mean difference of maternal 25(OH)D concentration at or near delivery among women who were supplemented with vitamin D in pregnancy versus who were not

**
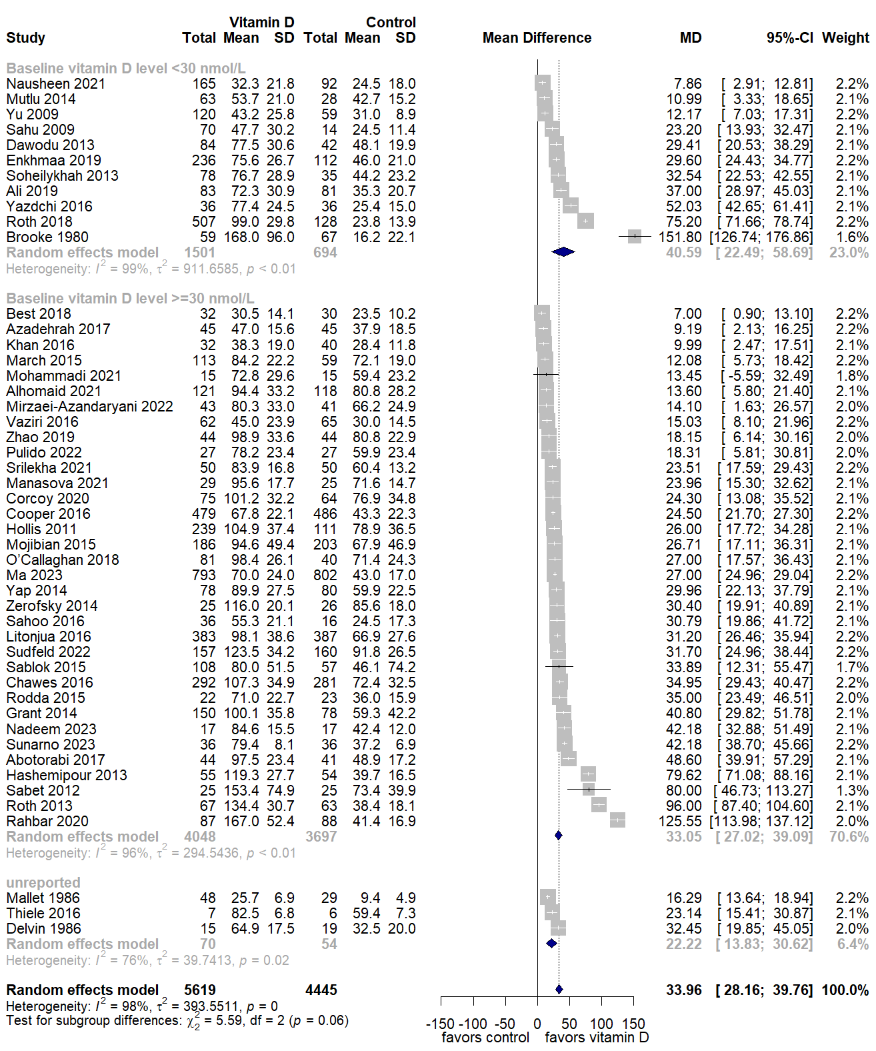
**

**Figure S3.10.9** Subgroup analysis by maternal population mean 25(OH)D concentration (50 nmol/L) for the mean difference of maternal 25(OH)D concentration at or near delivery among women who were supplemented with vitamin D in pregnancy versus who were not


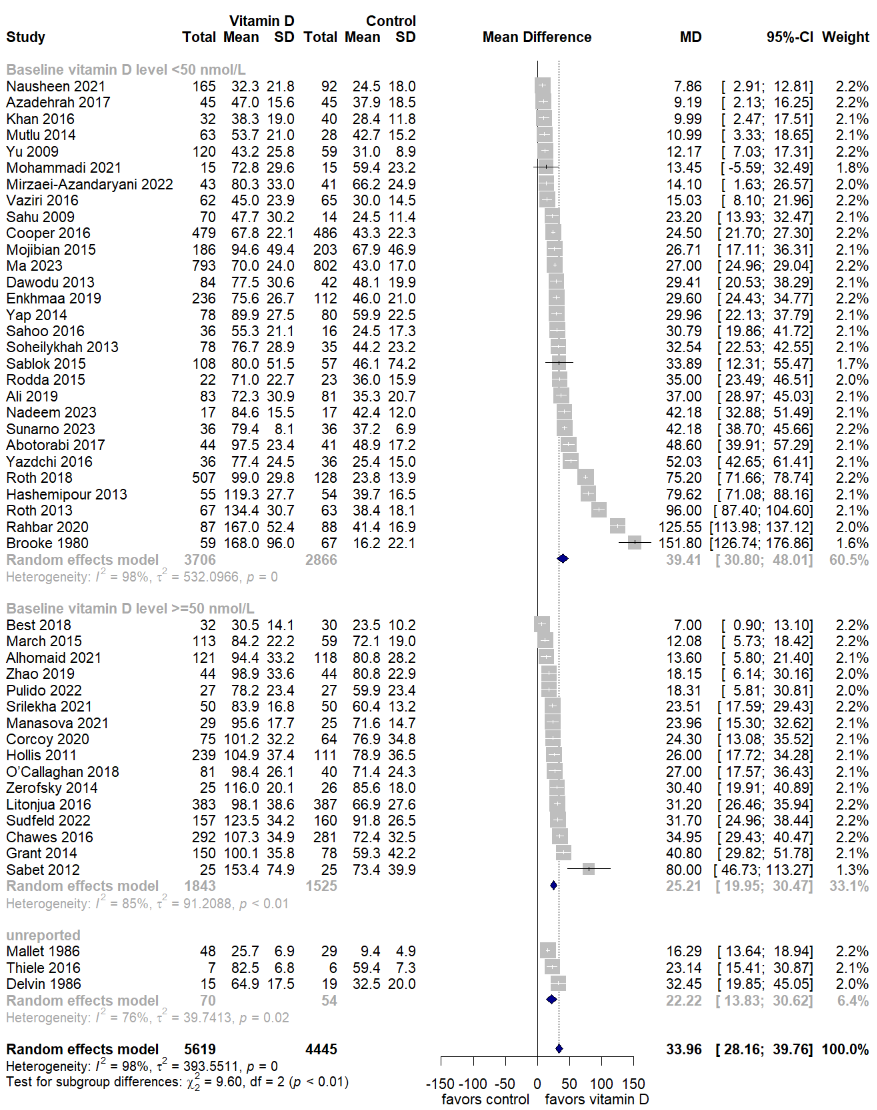


**11. Stillbirth or intrauterine death**

**Figure S3.11.1** Primary analysis for the risk ratio of stillbirth or intrauterine death among women who were supplemented with vitamin D in pregnancy versus who were not

**
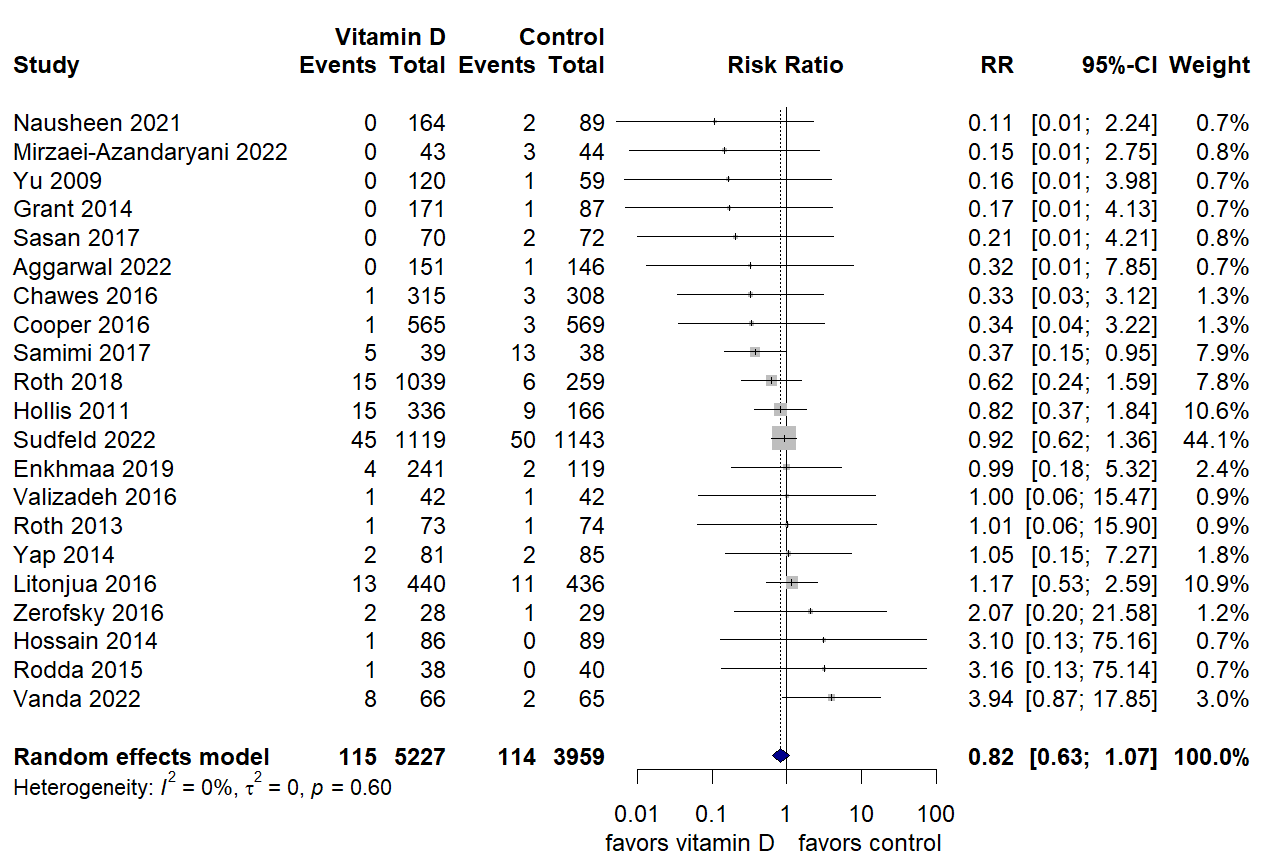
**

**Figure S3.11.2** Subgroup analysis by intervention type for the risk ratio of stillbirth or intrauterine death among women who were supplemented with vitamin D in pregnancy versus who were not


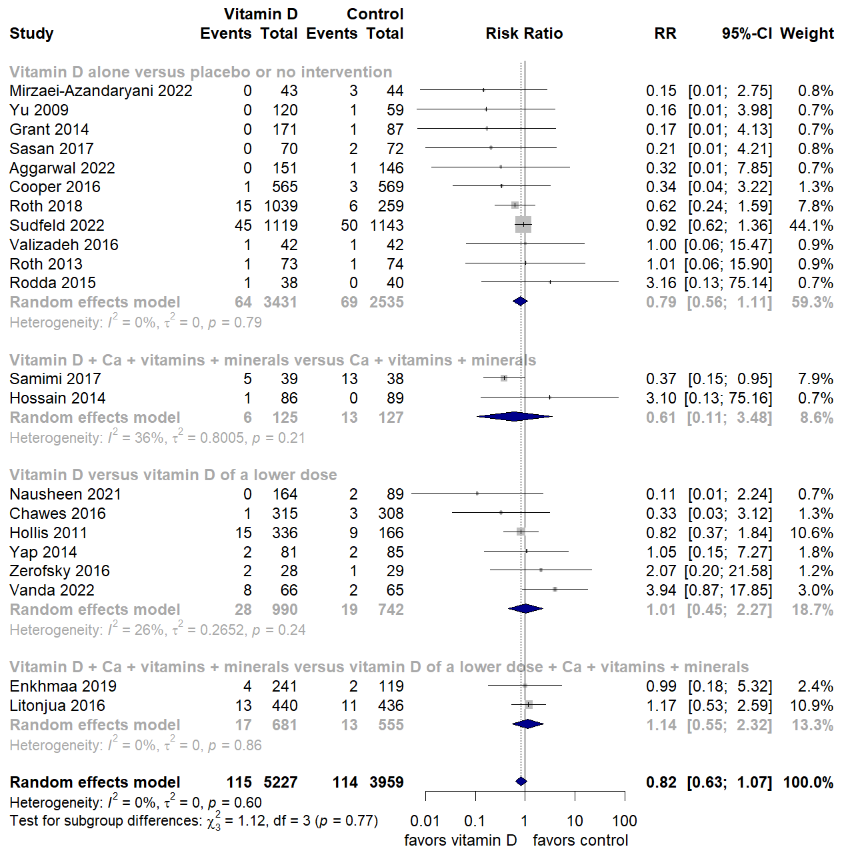


**Figure S3.11.3** Subgroup analysis by population type for the risk ratio of stillbirth or intrauterine death among women who were supplemented with vitamin D in pregnancy versus who were not


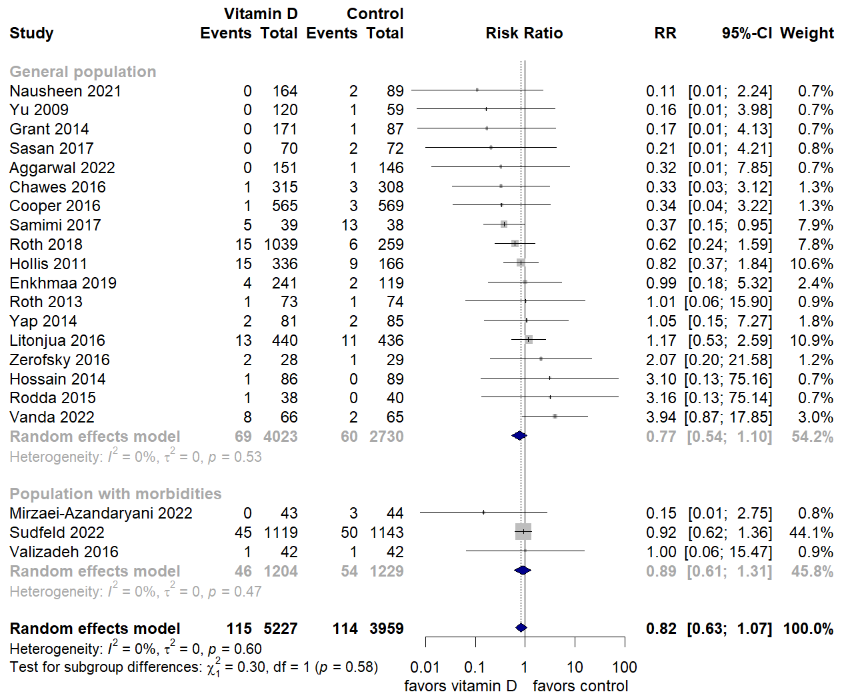


**Figure S3.11.4** Subgroup analysis by intervention dose for the risk ratio of stillbirth or intrauterine death among women who were supplemented with vitamin D in pregnancy versus who were not


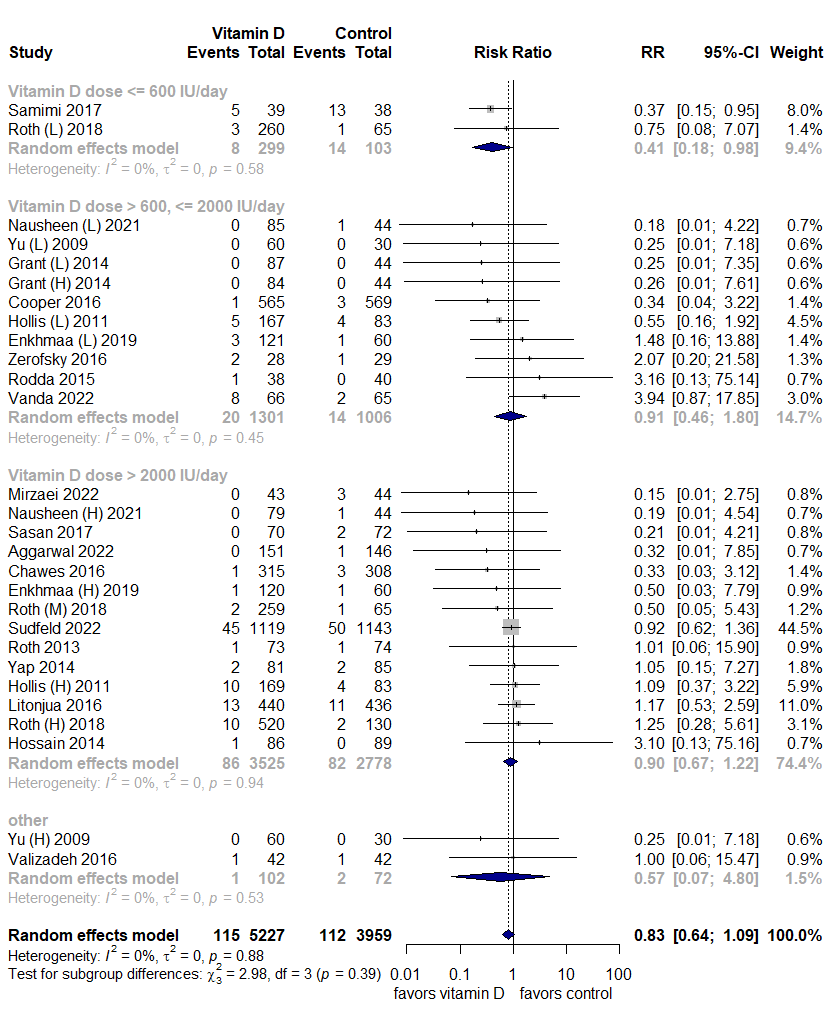


**Figure S3.11.5** Subgroup analysis by administration frequency for the risk ratio of stillbirth or intrauterine death among women who were supplemented with vitamin D in pregnancy versus who were not


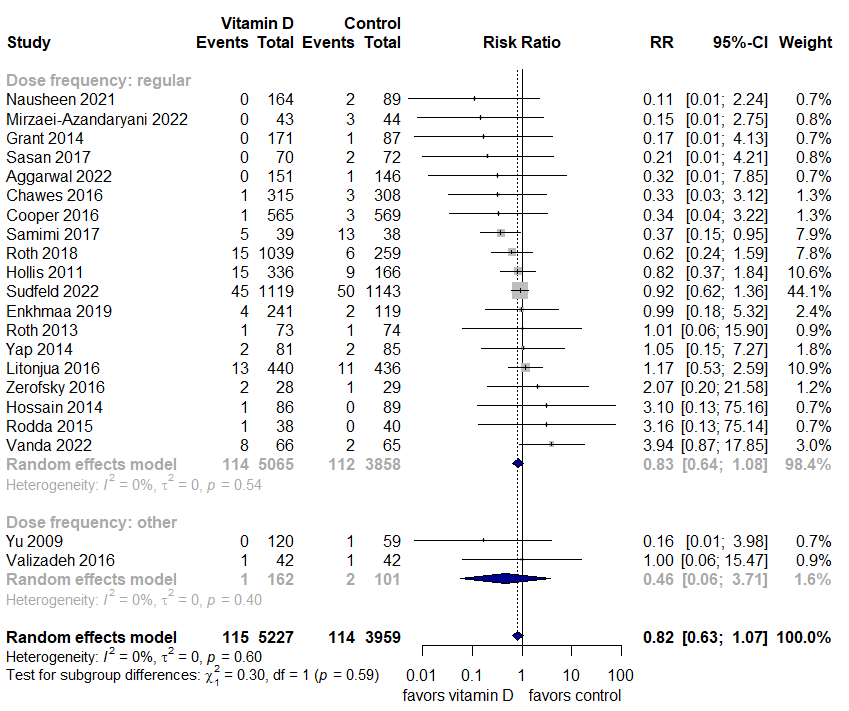


**Figure S3.11.6** Subgroup analysis by supplement form for the risk ratio of stillbirth or intrauterine death among women who were supplemented with vitamin D in pregnancy versus who were not


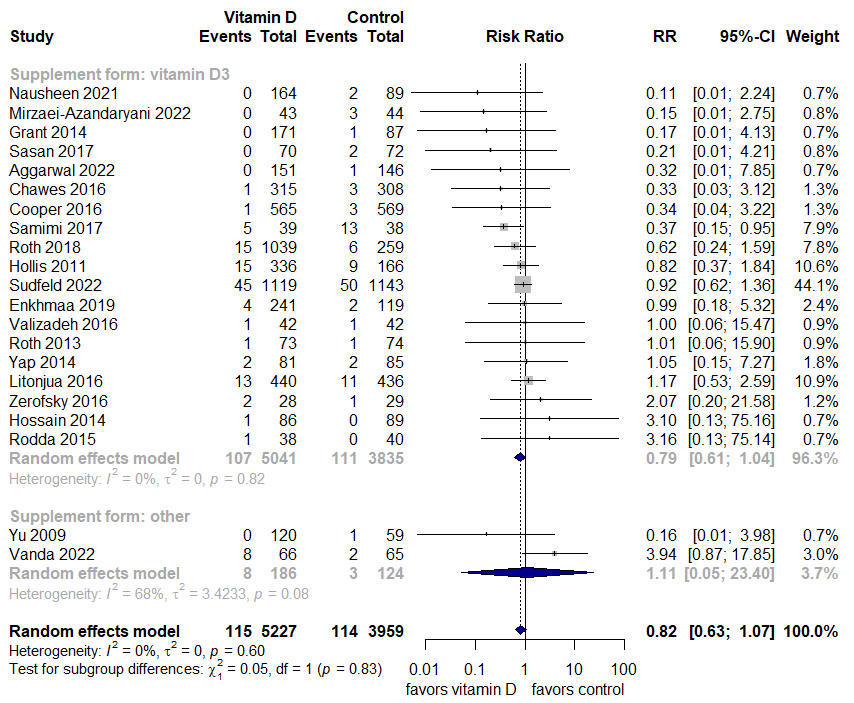


**Figure S3.11.7** Subgroup analysis by trimester of supplementation initiation for the risk ratio of stillbirth or intrauterine death among women who were supplemented with vitamin D in pregnancy versus who were not

**
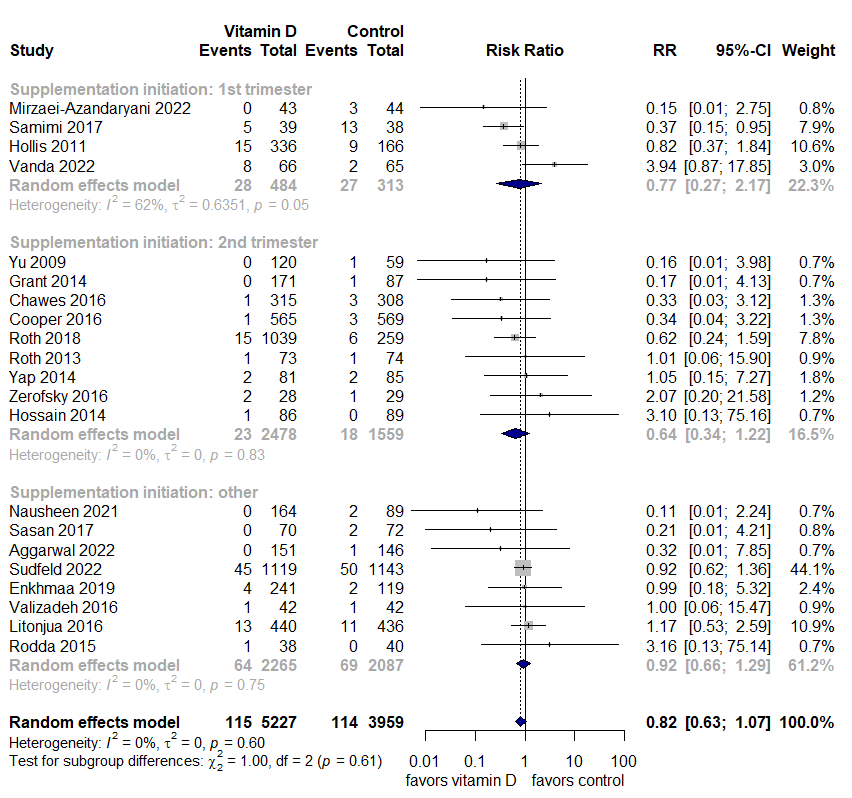
**

**Figure S3.11.8** Subgroup analysis by maternal population mean 25(OH)D concentration (30 nmol/L) of the risk ratio of stillbirth or intrauterine death among women who were supplemented with vitamin D in pregnancy versus who were not


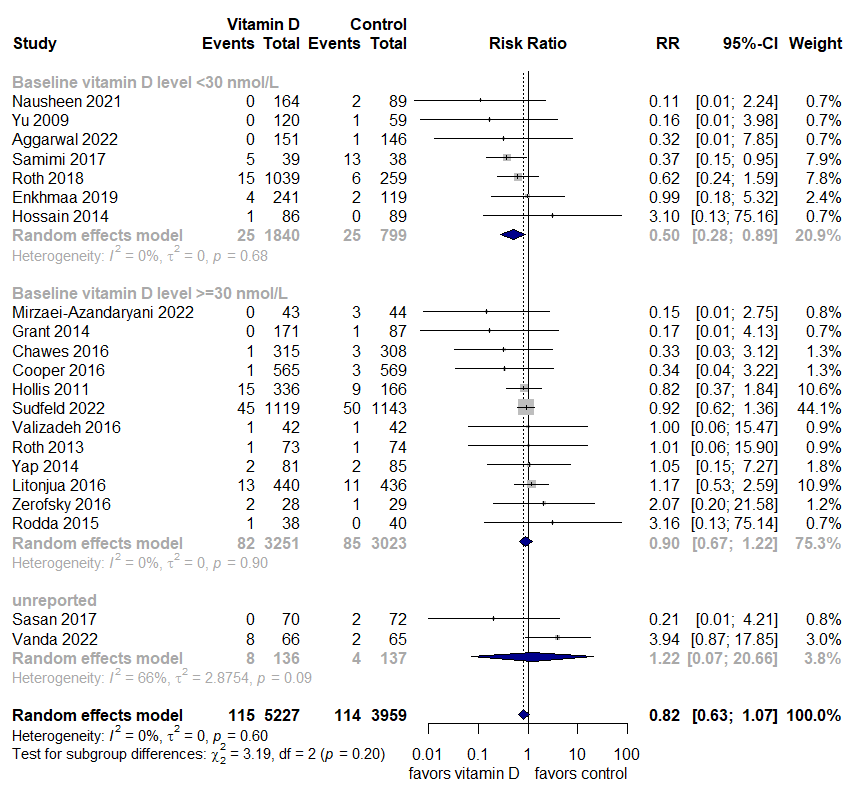


**Figure S3.11.9** Subgroup analysis by maternal population mean 25(OH)D concentration (50 nmol/L) of the risk ratio of stillbirth or intrauterine death among women who were supplemented with vitamin D in pregnancy versus who were not


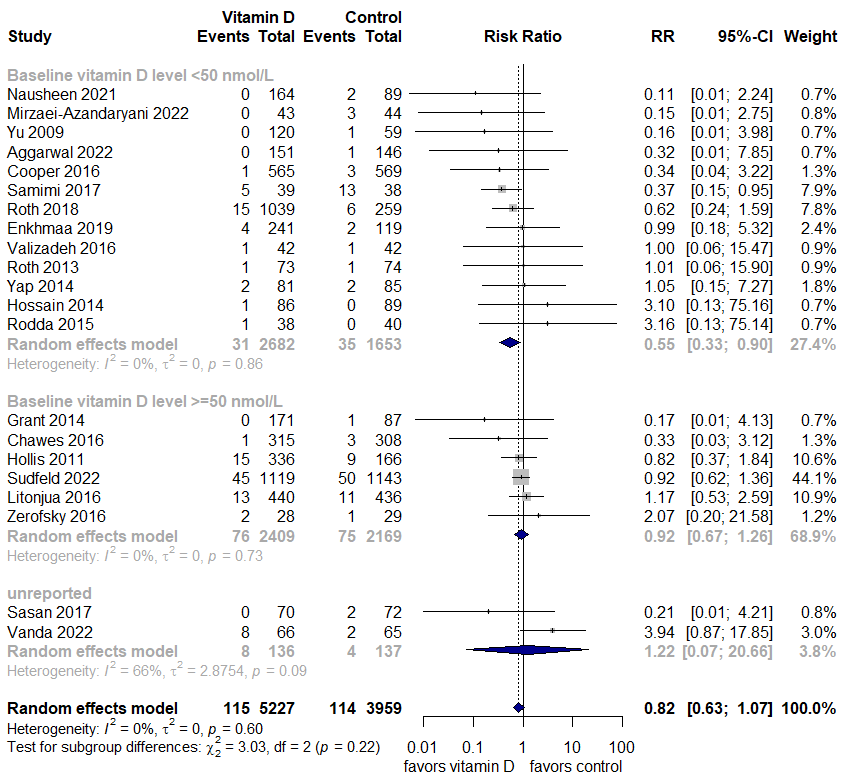


**12. Low birthweight infant (birthweight < 2500g)**

**Figure S3.12.1** Primary analysis for the risk ratio of low birthweight infants among women who were supplemented with vitamin D in pregnancy versus who were not

**
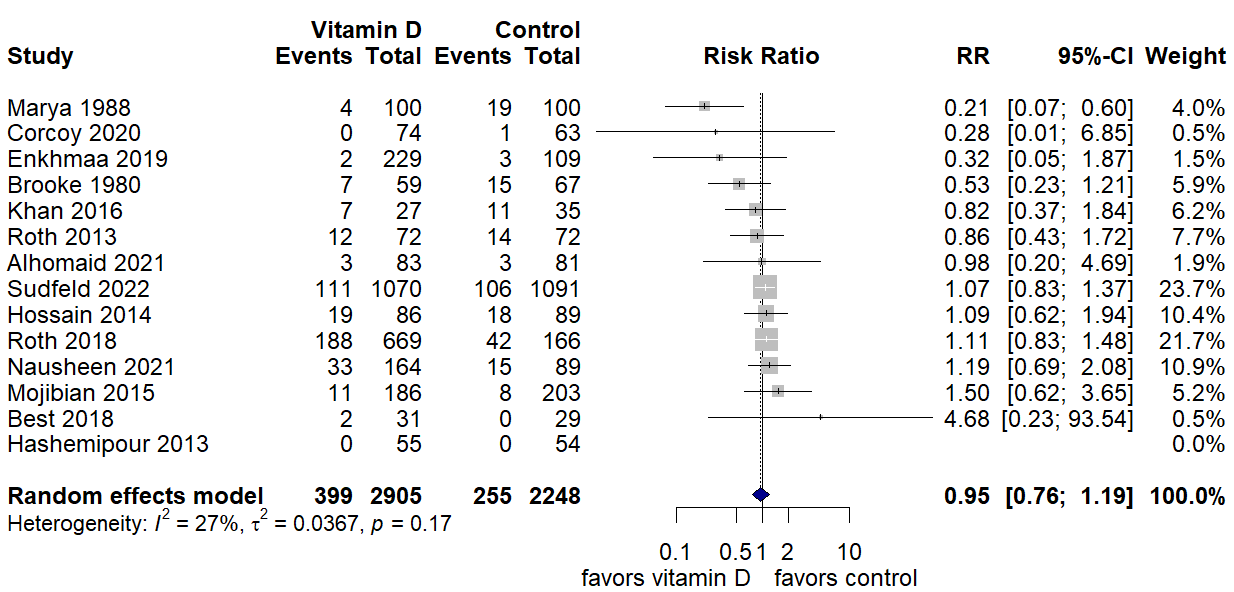
**

**Figure S3.12.2** Subgroup analysis by intervention type for the risk ratio of low birthweight infants among women who were supplemented with vitamin D in pregnancy versus who were not


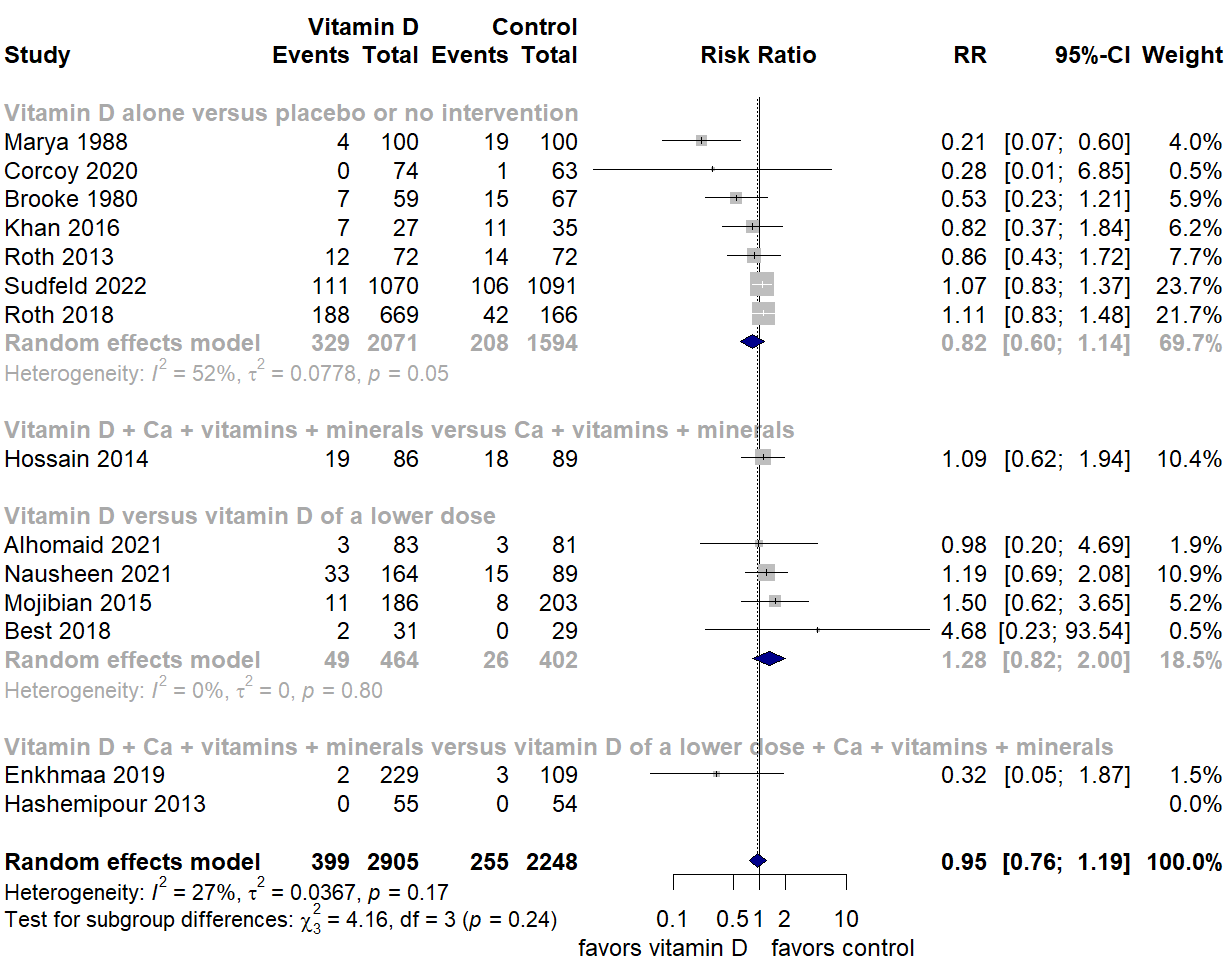


**Figure S3.12.3** Subgroup analysis by population type for the risk ratio of low birthweight infants among women who were supplemented with vitamin D in pregnancy versus who were not


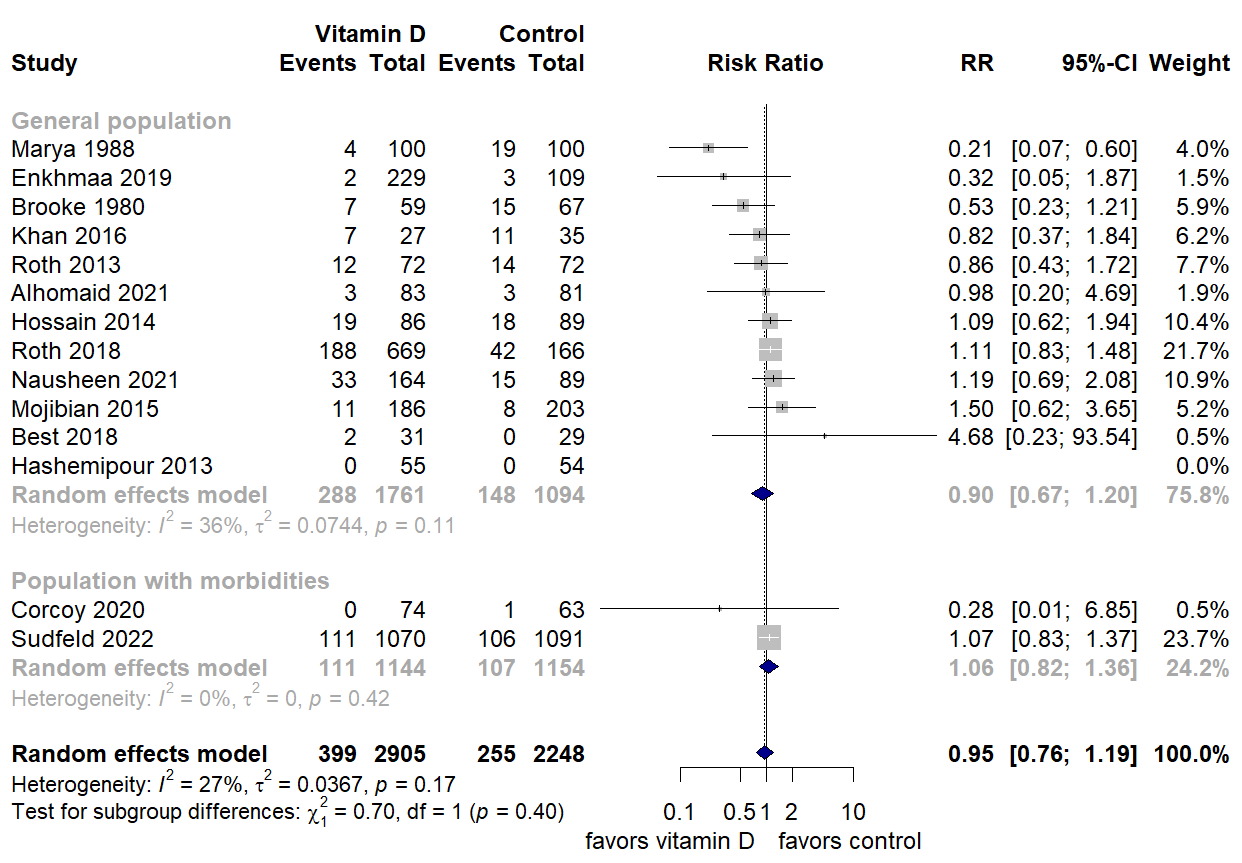


**Figure S3.12.4** Subgroup analysis by intervention dose for the risk ratio of low birthweight infants among women who were supplemented with vitamin D in pregnancy versus who were not


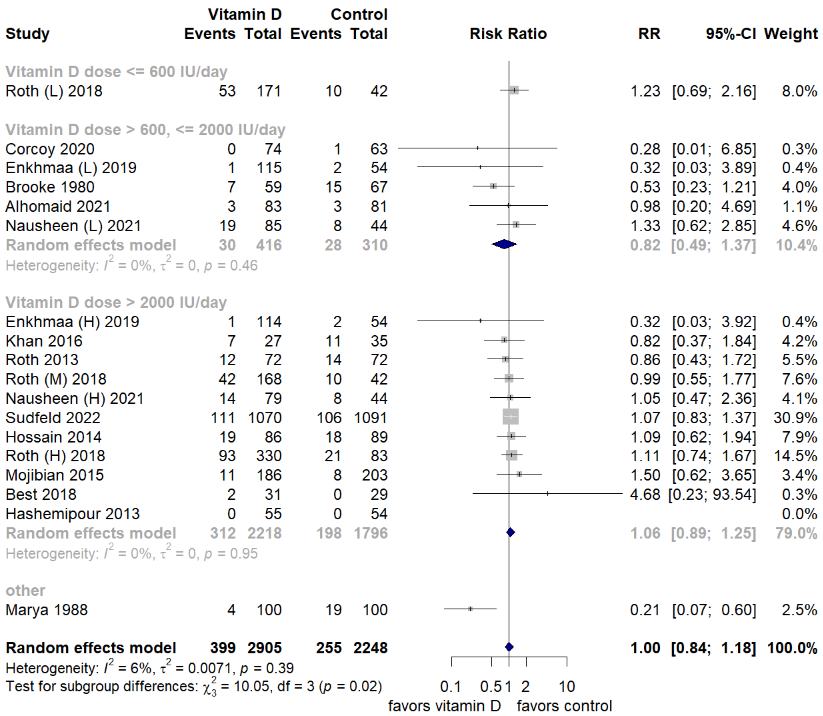


**Figure S3.12.5** Subgroup analysis by administration frequency for the risk ratio of low birthweight infants among women who were supplemented with vitamin D in pregnancy versus who were not


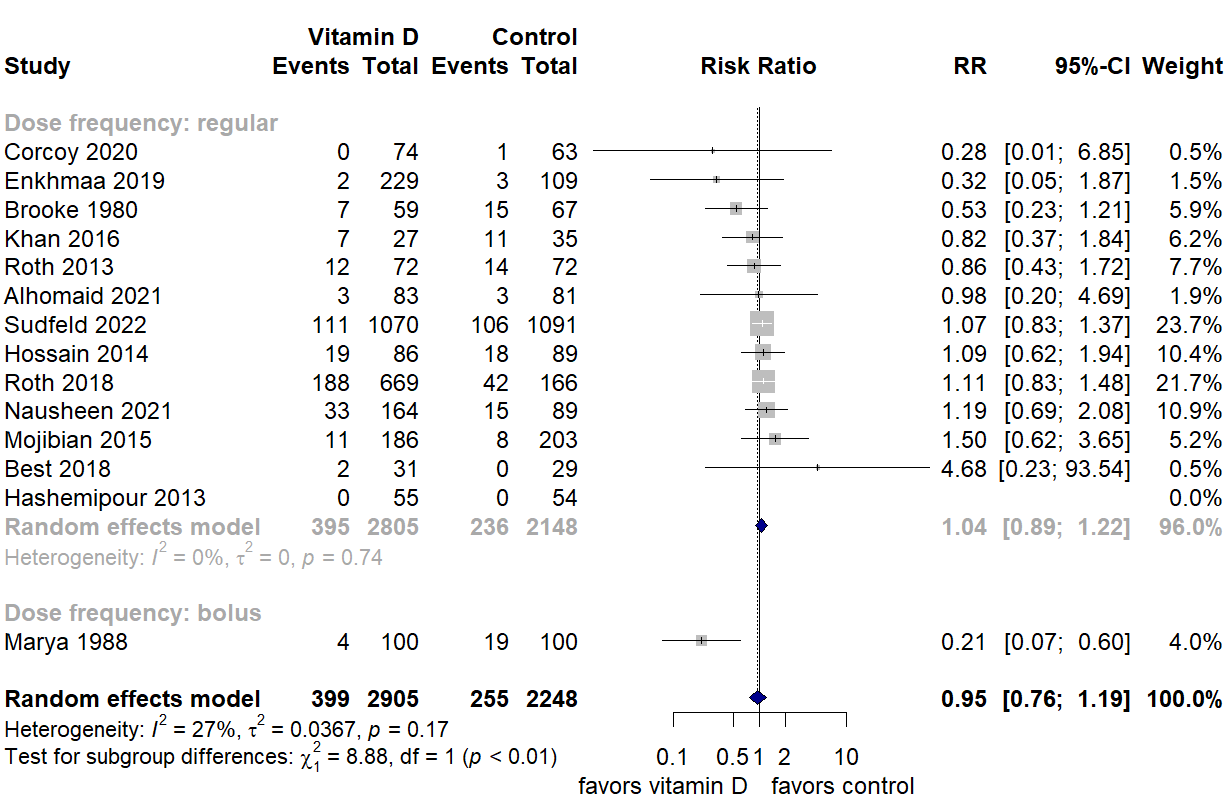
s

**Figure S3.12.6** Subgroup analysis by supplement form for the risk ratio of low birthweight infants among women who were supplemented with vitamin D in pregnancy versus who were not


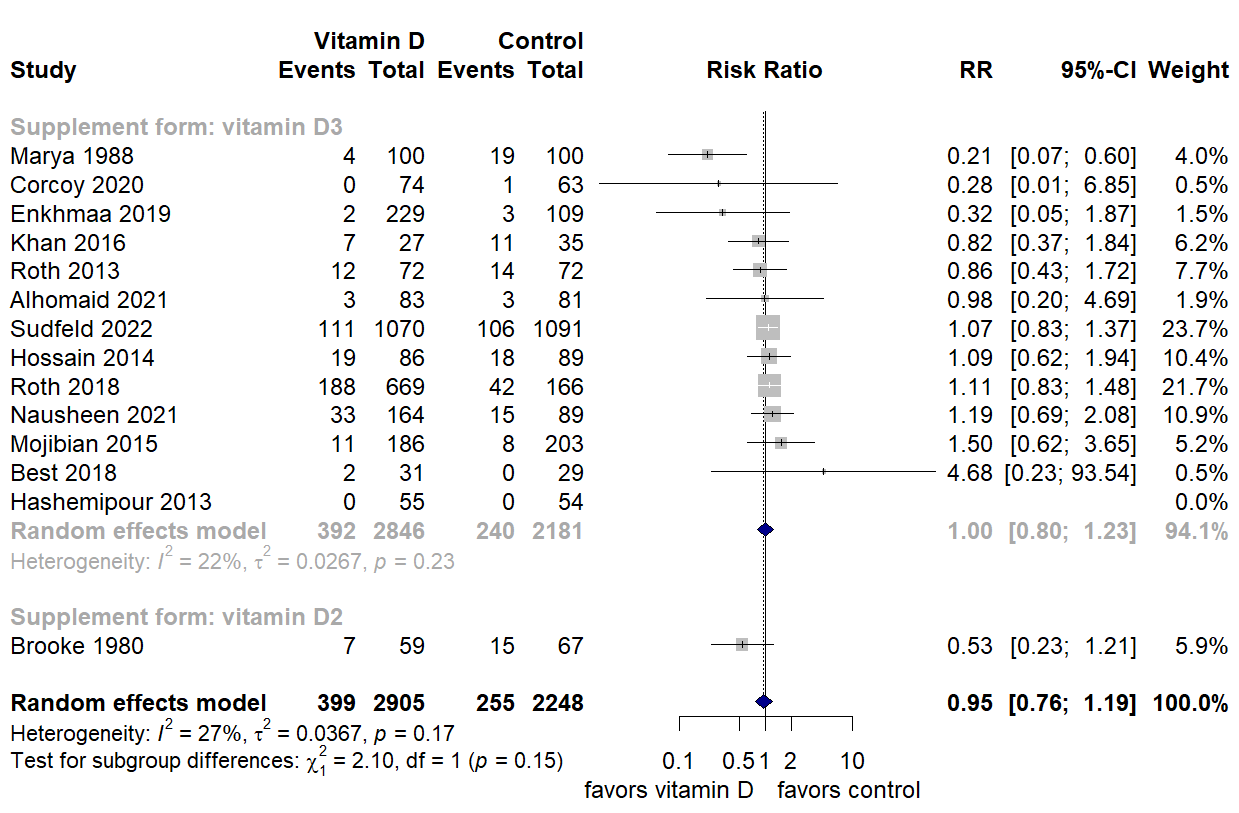


**Figure S3.12.7** Subgroup analysis by trimester of supplementation initiation form for the risk ratio of low birthweight infants among women who were supplemented with vitamin D in pregnancy versus who were not

**Figure S3.12.8** Subgroup analysis by maternal population mean 25(OH)D concentration (30 nmol/L) for the risk ratio of low birthweight infants among women who were supplemented with vitamin D in pregnancy versus who were not

**Figure S3.12.9** Subgroup analysis by maternal population mean 25(OH)D concentration (50 nmol/L) of the risk ratio of low birthweight infants among women who were supplemented with vitamin D in pregnancy versus who were not

**13. Preterm birth (<37 weeks)**

**Figure S3.13.1** Primary analysis for the risk ratio of preterm birth among women who were supplemented with vitamin D in pregnancy versus who were not

**Figure S3.13.2** Subgroup analysis by intervention type for the risk ratio of preterm birth among women who were supplemented with vitamin D in pregnancy versus who were not

**Figure S3.13.3** Subgroup analysis by population type for the risk ratio of preterm birth among women who were supplemented with vitamin D in pregnancy versus who were not

**Figure S3.13.4** Subgroup analysis by intervention dose for the risk ratio of preterm birth among women who were supplemented with vitamin D in pregnancy versus who were not

**Figure S3.13.5** Subgroup analysis by administration frequency for the risk ratio of preterm birth among women who were supplemented with vitamin D in pregnancy versus who were not

**Figure S3.13.6** Subgroup analysis by supplement form for the risk ratio of preterm birth among women who were supplemented with vitamin D in pregnancy versus who were not

**Figure S3.13.7** Subgroup analysis by trimester of supplementation initiation for the risk ratio of preterm birth among women who were supplemented with vitamin D in pregnancy versus who were not

**Figure S3.13.8** Subgroup analysis by maternal population mean 25(OH)D concentration (30 nmol/L) for the risk ratio of preterm birth among women who were supplemented with vitamin D in pregnancy versus who were not

**Figure S3.13.9** Subgroup analysis by maternal population mean 25(OH)D concentration (50 nmol/L) of the risk ratio of preterm birth among women who were supplemented with vitamin D in pregnancy versus who were not

**14. Small for gestational age (birthweight <10th percentile)**

**Figure S3.14.1** Primary analysis for the risk ratio of small for gestational age infants among women who were supplemented with vitamin D in pregnancy versus who were not

**Figure S3.14.2** Subgroup analysis by intervention type for the risk ratio of small for gestational age infants among women who were supplemented with vitamin D in pregnancy versus who were not

**Figure S3.14.3** Subgroup analysis by population type for the risk ratio of small for gestational age infants among women who were supplemented with vitamin D in pregnancy versus who were not

**Figure S3.14.4** Subgroup analysis by intervention dose for the risk ratio of small for gestational age infants among women who were supplemented with vitamin D in pregnancy versus who were not

**Figure S3.14.5** Subgroup analysis by administration frequency for the risk ratio of small for gestational age infants among women who were supplemented with vitamin D in pregnancy versus who were not

**Figure S3.14.6** Subgroup analysis by supplement form for the risk ratio of small for gestational age infants among women who were supplemented with vitamin D in pregnancy versus who were not

**Figure S3.14.7** Subgroup analysis by trimester of supplementation initiation for the risk ratio of small for gestational age infants among women who were supplemented with vitamin D in pregnancy versus who were not

**Figure S3.14.8** Subgroup analysis by maternal population mean 25(OH)D concentration (30 nmol/L) for the risk ratio of small for gestational age infants among women who were supplemented with vitamin D in pregnancy versus who were not

**Figure S3.14.9** Subgroup analysis by maternal population mean 25(OH)D concentration (50 nmol/L) of the risk ratio of small for gestational age infants among women who were supplemented with vitamin D in pregnancy versus who were not

**15. Congenital malformations**

**Figure S3.15.1** Primary analysis for the risk ratio of infants with congenital malformations among women who were supplemented with vitamin D in pregnancy versus who were not

**Figure S3.15.2** Subgroup analysis by intervention type for the risk ratio of infants with congenital malformations among women who were supplemented with vitamin D in pregnancy versus who were not

**Figure S3.15.3** Subgroup analysis by population type for the risk ratio of infants with congenital malformations among women who were supplemented with vitamin D in pregnancy versus who were not

**Figure S3.15.4** Subgroup analysis by intervention dose for the risk ratio of infants with congenital malformations among women who were supplemented with vitamin D in pregnancy versus who were not

**Figure S3.15.5** Subgroup analysis by administration frequency for the risk ratio of infants with congenital malformations among women who were supplemented with vitamin D in pregnancy versus who were not

**Figure S3.15.6** Subgroup analysis by supplement form for the risk ratio of infants with congenital malformations among women who were supplemented with vitamin D in pregnancy versus who were not

**Figure S3.15.7** Subgroup analysis by trimester of supplementation initiation for the risk ratio of infants with congenital malformations among women who were supplemented with vitamin D in pregnancy versus who were not

**Figure S3.15.8** Subgroup analysis by maternal population mean 25(OH)D concentration (30 nmol/L) for the risk ratio of infants with congenital malformations among women who were supplemented with vitamin D in pregnancy versus who were not

**Figure S3.15.9** Subgroup analysis by maternal population mean 25(OH)D concentration (50 nmol/L) of the risk ratio of infants with congenital malformation among women who were supplemented with vitamin D in pregnancy versus who were not

**16. Admission to neonatal intensive care unit (NICU)**

**Figure S3.16.1** Primary analysis for the risk ratio of admission to NICU for infants born to women who were supplemented with vitamin D in pregnancy versus who were not

**Figure S3.16.2** Subgroup analysis by intervention type for the risk ratio of admission to NICU for infants born to women who were supplemented with vitamin D in pregnancy versus who were not

**Figure S3.16.3** Subgroup analysis by population type for the risk ratio of admission to NICU for infants born to women who were supplemented with vitamin D in pregnancy versus who were not

**Figure S3.16.4** Subgroup analysis by intervention dose for the risk ratio of admission to NICU for infants born to women who were supplemented with vitamin D in pregnancy versus who were not

**Figure S3.16.5** Subgroup analysis by administration frequency for the risk ratio of admission to NICU for infants born to women who were supplemented with vitamin D in pregnancy versus who were not

**Figure S3.16.6** Subgroup analysis by supplement form for the risk ratio of admission to NICU for infants born to women who were supplemented with vitamin D in pregnancy versus who were not

**Figure S3.16.7** Subgroup analysis by trimester of supplementation initiation for the risk ratio of admission to NICU for infants born to women who were supplemented with vitamin D in pregnancy versus who were not

**Figure S3.16.8** Subgroup analysis by maternal population mean 25(OH)D concentration (30 nmol/L) for the risk ratio of admission to NICU for infants born to women who were supplemented with vitamin D in pregnancy versus who were not

**Figure S3.16.9** Subgroup analysis by maternal population mean 25(OH)D concentration (50 nmol/L) of the risk ratio of admission to NICU for infants born to women who were supplemented with vitamin D in pregnancy versus who were not

**17. Gestational age (weeks)**

**Figure S3.17.1** Primary analysis for the mean difference of gestational age for infants born to women who were supplemented with vitamin D in pregnancy versus who were not

**Figure S3.17.2** Subgroup analysis by intervention type for the mean difference of gestational age for infants born to women who were supplemented with vitamin D in pregnancy versus who were not

**Figure S3.17.3** Subgroup analysis by population type for the mean difference of gestational age for infants born to women who were supplemented with vitamin D in pregnancy versus who were not

**Figure S3.17.4** Subgroup analysis by intervention dose for the mean difference of gestational age for infants born to women who were supplemented with vitamin D in pregnancy versus who were not

**Figure S3.17.5** Subgroup analysis by administration frequency for the mean difference of gestational age for infants born to women who were supplemented with vitamin D in pregnancy versus who were not

**Figure S3.17.6** Subgroup analysis by supplement form for the mean difference of gestational age for infants born to women who were supplemented with vitamin D in pregnancy versus who were not

**Figure S3.17.7** Subgroup analysis by trimester of supplementation initiation for the mean difference of gestational age for infants born to women who were supplemented with vitamin D in pregnancy versus who were not

**Figure S3.17.8** Subgroup analysis by maternal population mean 25(OH)D concentration (30 nmol/L) for the mean difference of gestational age for infants born to women who were supplemented with vitamin D in pregnancy versus who were not

**Figure S3.17.9** Subgroup analysis by maternal population mean 25(OH)D concentration (50 nmol/L) for the mean difference of gestational age for infants born to women who were supplemented with vitamin D in pregnancy versus who were not

**18. Birthweight (g)**

**Figure S3.18.1** Primary analysis for the mean difference of birthweight for infants born to women who were supplemented with vitamin D in pregnancy versus who were not

**Figure S3.18.2** Subgroup analysis by intervention type for the mean difference of birthweight for infants born to women who were supplemented with vitamin D in pregnancy versus who were not

**Figure S3.18.3** Subgroup analysis by population type for the mean difference of birthweight for infants born to women who were supplemented with vitamin D in pregnancy versus who were not

**Figure S3.18.4** Subgroup analysis by intervention dose for the mean difference of birthweight for infants born to women who were supplemented with vitamin D in pregnancy versus who were not

**Figure S3.18.5** Subgroup analysis by administration frequency for the mean difference of birthweight for infants born to women who were supplemented with vitamin D in pregnancy versus who were not

**Figure S3.18.6** Subgroup analysis by supplement form for the mean difference of birthweight for infants born to women who were supplemented with vitamin D in pregnancy versus who were not

**Figure S3.18.7** Subgroup analysis by trimester of supplementation initiation for the mean difference of birthweight for infants born to women who were supplemented with vitamin D in pregnancy versus who were not

**Figure S3.18.8** Subgroup analysis by maternal population mean 25(OH)D concentration (30 nmol/L) for the mean difference of birthweight for infants born to women who were supplemented with vitamin D in pregnancy versus who were not

**Figure S3.18.9** Subgroup analysis by maternal population mean 25(OH)D concentration (50 nmol/L) for the mean difference of birthweight for infants born to women who were supplemented with vitamin D in pregnancy versus who were not

**19. Birth body length (cm)**

**Figure S3.19.1** Primary analysis for the mean difference of birth body length for infants born to women who were supplemented with vitamin D in pregnancy versus who were not

**Figure S3.19.2** Subgroup analysis by intervention type for the mean difference of birth body length for infants born to women who were supplemented with vitamin D in pregnancy versus who were not

**Figure S3.19.3** Subgroup analysis by population type for the mean difference of birth body length for infants born to women who were supplemented with vitamin D in pregnancy versus who were not

**Figure S3.19.4** Subgroup analysis by intervention dose for the mean difference of birth body length for infants born to women who were supplemented with vitamin D in pregnancy versus who were not

**Figure S3.19.5** Subgroup analysis by administration frequency for the mean difference of birth body length for infants born to women who were supplemented with vitamin D in pregnancy versus who were not

**Figure S3.19.6** Subgroup analysis by supplement form for the mean difference of birth body length for infants born to women who were supplemented with vitamin D in pregnancy versus who were not

**Figure S3.19.7** Subgroup analysis by trimester of supplementation initiation for the mean difference of birth body length for infants born to women who were supplemented with vitamin D in pregnancy versus who were not

**Figure S3.19.8** Subgroup analysis by maternal population mean 25(OH)D concentration (30 nmol/L) for the mean difference of birth body length for infants born to women who were supplemented with vitamin D in pregnancy versus who were not

**Figure S3.19.9** Subgroup analysis by maternal population mean 25(OH)D concentration (50 nmol/L) for the mean difference of birth body length for infants born to women who were supplemented with vitamin D in pregnancy versus who were not

**20. Birth head circumference (cm)**

**Figure S3.20.1** Primary analysis for the mean difference of birth head circumference for infants born to women who were supplemented with vitamin D in pregnancy versus who were not

**Figure S3.20.2** Subgroup analysis by intervention type for the mean difference of birth head circumference for infants born to women who were supplemented with vitamin D in pregnancy versus who were not

**Figure S3.20.3** Subgroup analysis by population type for the mean difference of birth head circumference for infants born to women who were supplemented with vitamin D in pregnancy versus who were not

**Figure S3.20.4** Subgroup analysis by intervention dose for the mean difference of birth head circumference for infants born to women who were supplemented with vitamin D in pregnancy versus who were not

**Figure S3.20.5** Subgroup analysis by administration frequency for the mean difference of birth head circumference for infants born to women who were supplemented with vitamin D in pregnancy versus who were not

**Figure S3.20.6** Subgroup analysis by supplement form for the mean difference of birth head circumference for infants born to women who were supplemented with vitamin D in pregnancy versus who were not

**Figure S3.20.7** Subgroup analysis by trimester of supplementation initiation for the mean difference of birth head circumference for infants born to women who were supplemented with vitamin D in pregnancy versus who were not

**Figure S3.20.8** Subgroup analysis by maternal population mean 25(OH)D concentration (30 nmol/L) for the mean difference of birth head circumference for infants born to women who were supplemented with vitamin D in pregnancy versus who were not

**Figure S3.20.9** Subgroup analysis by maternal population mean 25(OH)D concentration (50 nmol/L) for the mean difference of birth head circumference for infants born to women who were supplemented with vitamin D in pregnancy versus who were not

**21. Cord 25(OH)D concentration (nmol/L)**

**Figure S3.21.1** Primary analysis for the mean difference of cord 25(OH)D concentration for infants born to women who were supplemented with vitamin D in pregnancy versus who were not

**Figure S3.21.2** Subgroup analysis by intervention type for the mean difference of cord 25(OH)D concentration for infants born to women who were supplemented with vitamin D in pregnancy versus who were not

**Figure S3.21.3** Subgroup analysis by population type for the mean difference of cord 25(OH)D concentration for infants born to women who were supplemented with vitamin D in pregnancy versus who were not

**Figure S3.21.4** Subgroup analysis by intervention dose for the mean difference of cord 25(OH)D concentration for infants born to women who were supplemented with vitamin D in pregnancy versus who were not

**Figure S3.21.5** Subgroup analysis by administration frequency for the mean difference of cord 25(OH)D concentration for infants born to women who were supplemented with vitamin D in pregnancy versus who were not

**Figure S3.21.6** Subgroup analysis by supplement form for the mean difference of cord 25(OH)D concentration for infants born to women who were supplemented with vitamin D in pregnancy versus who were not

**Figure S3.21.7** Subgroup analysis by trimester of supplementation initiation for the mean difference of cord 25(OH)D concentration for infants born to women who were supplemented with vitamin D in pregnancy versus who were not

**Figure S3.21.8** Subgroup analysis by maternal population mean 25(OH)D concentration (30 nmol/L) for the mean difference of cord 25(OH)D concentration for infants born to women who were supplemented with vitamin D in pregnancy versus who were not

**Figure S3.21.9** Subgroup analysis by maternal population mean 25(OH)D concentration (50 nmol/L) for the mean difference of cord 25(OH)D concentration for infants born to women who were supplemented with vitamin D in pregnancy versus who were not

**22. Neonatal death**

**Figure S3.22.1** Primary analysis for the risk ratio of neonatal deaths for infants born to women who were supplemented with vitamin D in pregnancy versus who were not

**Figure S3.22.2** Subgroup analysis by intervention type for the risk ratio of neonatal deaths for infants born to women who were supplemented with vitamin D in pregnancy versus who were not

**Figure S3.22.3** Subgroup analysis by population type for the risk ratio of neonatal deaths for infants born to women who were supplemented with vitamin D in pregnancy versus who were not

**Figure S3.22.4** Subgroup analysis by intervention dose for the risk ratio of neonatal deaths for infants born to women who were supplemented with vitamin D in pregnancy versus who were not

**Figure S3.22.5** Subgroup analysis by administration frequency for the risk ratio of neonatal deaths for infants born to women who were supplemented with vitamin D in pregnancy versus who were not

**Figure S3.22.6** Subgroup analysis by supplement form for the risk ratio of neonatal deaths for infants born to women who were supplemented with vitamin D in pregnancy versus who were not

**Figure S3.22.7** Subgroup analysis by trimester of supplementation initiation for the risk ratio of neonatal deaths for infants born to women who were supplemented with vitamin D in pregnancy versus who were not

**Figure S3.22.8** Subgroup analysis by maternal population mean 25(OH)D concentration (30 nmol/L)for the risk ratio of neonatal deaths for infants born to women who were supplemented with vitamin D in pregnancy versus who were not

**Figure S3.22.9** Subgroup analysis by maternal population mean 25(OH)D concentration (50 nmol/L) for the risk ratio of neonatal deaths for infants born to women who were supplemented with vitamin D in pregnancy versus who were not

**23. Neonatal hypercalcemia**

**Figure S3.23.1** Primary analysis for the risk ratio of neonatal hypercalcemia for infants born to mothers who were versus who were not supplemented with vitamin D in pregnancy

**Figure S3.23.2** Sensitivity analysis for the risk ratio of neonatal hypercalcemia for infants born to mothers who were versus who were not supplemented with vitamin D in pregnancy

**24. Neonatal hypocalcemia**

**Figure S3.24.1** Primary analysis for the risk ratio of neonatal hypocalcemia for infants born to women who were supplemented with vitamin D in pregnancy versus who were not

**Figure S3.24.2** Sensitivity analysis for the risk ratio of neonatal hypocalcemia for infants born to mothers who were versus who were not supplemented with vitamin D in pregnancy

**25. Respiratory infection**

**Figure S25.1** Primary analysis for the risk ratio of respiratory infections for infants born to women who were supplemented with vitamin D in pregnancy versus who were not

**26. Upper respiratory tract infection**

**Figure S26.1** Primary analysis for the risk ratio of upper respiratory tract infections for infants born to women who were supplemented with vitamin D in pregnancy versus who were not

**27. Lower respiratory tract infection**

**Figure S3.27.1** Primary analysis for the risk ratio of lower respiratory tract infections for infants born to women who were supplemented with vitamin D in pregnancy versus who were not

**Figure S3.27.2** Subgroup analysis by intervention type for the risk ratio of lower respiratory tract infections for infants born to women who were supplemented with vitamin D in pregnancy versus who were not

**Figure S3.27.3** Subgroup analysis by population type for the risk ratio of lower respiratory tract infections for infants born to women who were supplemented with vitamin D in pregnancy versus who were not

**Figure S3.27.4** Subgroup analysis by intervention dose for the risk ratio of lower respiratory tract infections for infants born to women who were supplemented with vitamin D in pregnancy versus who were not

**Figure S3.27.5** Subgroup analysis by administration frequency for the risk ratio of lower respiratory tract infections for infants born to women who were supplemented with vitamin D in pregnancy versus who were not

**Figure S3.27.6** Subgroup analysis by supplement form for the risk ratio of lower respiratory tract infections for infants born to women who were supplemented with vitamin D in pregnancy versus who were not

**Figure S3.27.7** Subgroup analysis by trimester of supplementation initiation for the risk ratio of lower respiratory tract infections for infants born to women who were supplemented with vitamin D in pregnancy versus who were not

**Figure S3.27.8** Subgroup analysis by maternal population mean 25(OH)D concentration (30 nmol/L) for the risk ratio of lower respiratory tract infections for infants born to women who were supplemented with vitamin D in pregnancy versus who were not

**Figure S3.27.9** Subgroup analysis by maternal population mean 25(OH)D concentration (50 nmol/L) for the risk ratio of lower respiratory tract infections for infants born to women who were supplemented with vitamin D in pregnancy versus who were not

**28. Asthma or recurrent/persistent wheeze by 3 y/o**

**Figure S3.28.1** Primary analysis for the risk ratio of asthma for infants and children by 3 y/o born to women who were supplemented with vitamin D in pregnancy versus who were not

**Figure S3.28.2** Sensitivity analysis for the risk ratio of asthma for infants and children by 3 y/o born to women who were supplemented with vitamin D in pregnancy versus who were not

**29. Infant body weight at 1 year (g)**

**Figure S3.29.1** Primary analysis for the mean difference of weight at 1 year old for infants born to women who were supplemented with vitamin D in pregnancy versus who were not

**Figure S3.29.2** Subgroup analysis by intervention type for the mean difference of weight at 1 year old for infants born to women who were supplemented with vitamin D in pregnancy versus who were not

**Figure S3.29.3** Subgroup analysis by population type for the mean difference of weight at 1 year old for infants born to women who were supplemented with vitamin D in pregnancy versus who were not

**Figure S3.29.4** Subgroup analysis by intervention dose for the mean difference of weight at 1 year old for infants born to women who were supplemented with vitamin D in pregnancy versus who were not

**Figure S3.29.5** Subgroup analysis by administration frequency for the mean difference of weight at 1 year old for infants born to women who were supplemented with vitamin D in pregnancy versus who were not

**Figure S3.29.6** Subgroup analysis by supplement form for the mean difference of weight at 1 year old for infants born to women who were supplemented with vitamin D in pregnancy versus who were not

**Figure S3.29.7** Subgroup analysis by trimester of supplementation initiation the mean difference of weight at 1 year old for infants born to mothers who were versus who were not supplemented with vitamin D in pregnancy

**Figure S3.29.8** Subgroup analysis by maternal population mean 25(OH)D concentration (30 nmol/L) for the mean difference of weight at 1 year old for infants born to women who were supplemented with vitamin D in pregnancy versus who were not

**Figure S3.29.9** Subgroup analysis by maternal population mean 25(OH)D concentration (50 nmol/L) for the mean difference of weight at 1 year old for infants born to women who were supplemented with vitamin D in pregnancy versus who were not

**30. Infant body length at 1 year (cm)**

**Figure S3.30.1** Primary analysis for the mean difference of body length at 1 year old for infants born to women who were supplemented with vitamin D in pregnancy versus who were not

**Figure S3.30.2** Subgroup analysis by intervention type for the mean difference of body length at 1 year old for infants born to women who were supplemented with vitamin D in pregnancy versus who were not

**Figure S3.30.3** Subgroup analysis by population type for the mean difference of body length at 1 year old for infants born to women who were supplemented with vitamin D in pregnancy versus who were not

**Figure S3.30.4** Subgroup analysis by intervention dose for the mean difference of body length at 1 year old for infants born to women who were supplemented with vitamin D in pregnancy versus who were not

**Figure S3.30.5** Subgroup analysis by administration frequency for the mean difference of body length at 1 year old for infants born to women who were supplemented with vitamin D in pregnancy versus who were not

**Figure S3.30.6** Subgroup analysis by supplement form for the mean difference of body length at 1 year old for infants born to women who were supplemented with vitamin D in pregnancy versus who were not

**Figure S3.30.7** Subgroup analysis by trimester of supplementation initiation the mean difference of body length at 1 year old for infants born to women who were supplemented with vitamin D in pregnancy versus who were not

**Figure S3.30.8** Subgroup analysis by maternal population mean 25(OH)D concentration (30 nmol/L) for the mean difference of body length at 1 year old for infants born to women who were supplemented with vitamin D in pregnancy versus who were not

**Figure S3.30.9** Subgroup analysis by maternal population mean 25(OH)D concentration (50 nmol/L) for the mean difference of length at 1 year old for infants born to women who were supplemented with vitamin D in pregnancy versus who were not

**31. Infant head circumference at 1 year (cm)**

**Figure S3.31.1** Primary analysis for the mean difference of head circumference at 1 year old for infants born to mothers who were versus who were not supplemented with vitamin D in pregnancy

**32. Weight-for-age z score at 1 year old**

**Figure S3.32.1** Primary analysis for the mean difference of weight-for-age z score at 1 year old for infants born to women who were supplemented with vitamin D in pregnancy versus who were not

**Figure S3.32.2** Subgroup analysis by intervention type for the mean difference of weight-for-age z score at 1 year old for infants born to women who were supplemented with vitamin D in pregnancy versus who were not

**Figure S3.32.3** Subgroup analysis by population type for the mean difference of weight-for-age z score at 1 year old for infants born to women who were supplemented with vitamin D in pregnancy versus who were not

**Figure S3.32.4** Subgroup analysis by intervention dose for the mean difference of weight-for-age z score at 1 year old for infants born to women who were supplemented with vitamin D in pregnancy versus who were not

**Figure S3.32.5** Subgroup analysis by administration frequency for the mean difference of weight-for-age z score at 1 year old for infants born to women who were supplemented with vitamin D in pregnancy versus who were not

**Figure S3.32.6** Subgroup analysis by supplement form for the mean difference of weight-for-age z score at 1 year old for infants born to women who were supplemented with vitamin D in pregnancy versus who were not

**Figure S3.32.7** Subgroup analysis by trimester of supplementation initiation the mean difference of weight-for-age z score at 1 year old for infants born to women who were supplemented with vitamin D in pregnancy versus who were not

**Figure S3.32.8** Subgroup analysis by maternal population mean 25(OH)D concentration (30 nmol/L) for the mean difference of weight-for-age z score at 1 year old for infants born to women who were supplemented with vitamin D in pregnancy versus who were not

**Figure S3.32.9** Subgroup analysis by maternal population mean 25(OH)D concentration (50 nmol/L) for the mean difference of weight-for-age z score at 1 year old for infants born to mothers who were supplemented with vitamin D in pregnancy versus who were not

**33. Length-for-age z score at 1 year**

**Figure S3.33.1** Primary analysis for the mean difference of length-for-age z score at 1 year old for infants born to mothers who were supplemented with vitamin D in pregnancy versus who were not

**Figure S3.33.2** Subgroup analysis by intervention type for the mean difference of length-for-age z score at 1 year old for infants born to women who were supplemented with vitamin D in pregnancy versus who were not

**Figure S3.33.3** Subgroup analysis by population type for the mean difference of length-for-age z score at 1 year old for infants born to women who were supplemented with vitamin D in pregnancy versus who were not

**Figure S3.33.4** Subgroup analysis by intervention dose for the mean difference of length-for-age z score at 1 year old for infants born to mothers who were versus who were not supplemented with vitamin D in pregnancy

**Figure S3.33.5** Subgroup analysis by administration frequency for the mean difference of length-for-age z score at 1 year old for infants born to women who were supplemented with vitamin D in pregnancy versus who were not

**Figure S3.33.6** Subgroup analysis by supplement form for the mean difference of length-for-age z score at 1 year old for infants born to women who were supplemented with vitamin D in pregnancy versus who were not

**Figure S3.33.7** Subgroup analysis by trimester of supplementation initiation for the mean difference of length-for-age z score at 1 year old for infants born to women who were supplemented with vitamin D in pregnancy versus who were not

**Figure S3.33.8** Subgroup analysis by maternal population mean 25(OH)D concentration (30 nmol/L) for the mean difference of length-for-age z score at 1 year old for infants born to women who were supplemented with vitamin D in pregnancy versus who were not

**Figure S3.33.9** Subgroup analysis by maternal population mean 25(OH)D concentration (50 nmol/L) for the mean difference of length-for-age z score at 1 year old for infants born to women who were supplemented with vitamin D in pregnancy versus who were not

**34. Head circumference-for-age z score at 1 year**

**Figure S3.34.1** Primary analysis for the mean difference of head circumference-for-age z score at 1 year old for infants born to women who were supplemented with vitamin D in pregnancy versus who were not

**35. Neonatal bone mineral content (g)**

**Figure S3.35.1** Primary analysis for the mean difference of neonatal bone mineral content for infants born to women who were supplemented with vitamin D in pregnancy versus who were not

**36. Neonatal bone mineral density (g/cm^2^)**

**Figure S3.36.1** Primary analysis for the mean difference of infant bone mineral content for infants born to women who were versus who were not supplemented with vitamin D in pregnancy

**37. Infant bone mineral content (g)**

**Figure S3.37.1** Primary analysis for the mean difference of infant bone mineral content for those born to women who were supplemented with vitamin D in pregnancy versus who were not

**38. Infant bone mineral density (g/cm^2^)**

**Figure S3.38.1** Primary analysis for the mean difference of infant bone mineral density for infants born to women who were supplemented with vitamin D in pregnancy versus who were not

**Figure S4. The contribution of trials to primary and sensitivity analyses**
